# Revealing the Hidden Complexity and Reactivity of Palladacyclic Precatalysts: The P(*o*-tolyl)_3_ Ligand Enables a Cocktail of Active Species Utilizing the Pd(II)/Pd(IV) and Pd(0)/Pd(II) Pathways for Efficient Catalysis

**DOI:** 10.1021/acscatal.4c02585

**Published:** 2024-08-09

**Authors:** David R. Husbands, Theo Tanner, Adrian C. Whitwood, Neil S. Hodnett, Katherine M. P. Wheelhouse, Ian J. S. Fairlamb

**Affiliations:** † Department of Chemistry, University of York, York, Heslington YO10 5DD, United Kingdom; ‡ Medicine Development & Supply, GSK Medicines Research Centre, Gunnels Wood Road, Stevenage, Hertfordshire SG1 2NY, U.K.

**Keywords:** palladium, cross-coupling, palladacycle, mechanism, Suzuki−Miyaura, Heck, kinetics

## Abstract

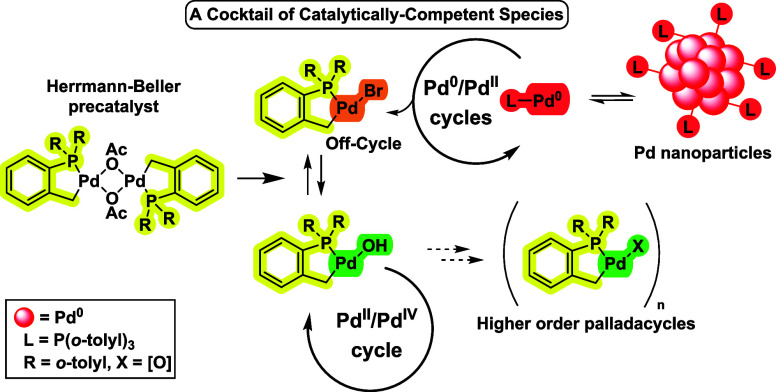

The ligand, P(*o*-tolyl)_3_, is ubiquitous in applied synthetic chemistry and catalysis, particularly in Pd-catalyzed processes, which typically include Pd(OAc)_2_ (most commonly used as Pd_3_(OAc)_6_) as a precatalyst. The Herrmann–Beller palladacycle [Pd(C^P)(μ_2_-OAc)]_2_ (where C^P = monocyclopalladated P(*o*-tolyl)_3_) is easily formed from reaction of Pd(OAc)_2_ with P(*o*-tolyl)_3_. The mechanisms by which this precatalyst system operates are inherently complex, with studies previously implicating Pd nanoparticles (PdNPs) as reservoirs for active Pd^(0)^ species in arylative cross-coupling reactions. In this study, we reveal the fascinating, complex, and nontrivial behavior of the palladacyclic group. First, in the presence of hydroxide base, [Pd(C^P)(μ_2_-OAc)]_2_ is readily converted into an activated form, [Pd(C^P)(μ_2_-OH)]_2_, which serves as a conduit for activation to catalytically relevant species. Second, palladacyclization imparts unique stability for catalytic species under reaction conditions, bringing into play a Pd^(II)^/Pd^(IV)^ cross-coupling mechanism. For a benchmark Suzuki–Miyaura cross-coupling (SMCC) reaction, there is a shift from a mononuclear Pd catalytic pathway to a PdNP-controlled catalytic pathway during the reaction. The activation pathway of [Pd(C^P)(μ_2_-OH)]_2_ has been studied using an arylphosphine-stabilized boronic acid and low-temperature NMR spectroscopic analysis, which sheds light on the preactivation step, with water and/or acid being critical for the formation of active Pd^(0)^ and Pd^(II)^ species. *In situ* reaction monitoring has demonstrated that there is a sensitivity to the structure of the arylboron species in the presence of pinacol. This work, taken together, highlights the mechanistic complexity accompanying the use of palladacyclic precatalyst systems. It builds on recent findings involving related Pd(OAc)_2_/PPh_3_ precatalyst systems which readily form higher order Pd_n_ clusters and PdNPs under cross-coupling reaction conditions. Thus, generally, one needs to be cautious with the assumption that Pd(OAc)_2_/tertiary phosphine mixtures cleanly deliver mononuclear “Pd(0)L_n_” species and that any assessment of individual phosphine ligands may need to be taken on a case-by-case basis.

## Introduction

### Catalytic Applications of the Herrmann–Beller Palladacycle

**Figure 1:**
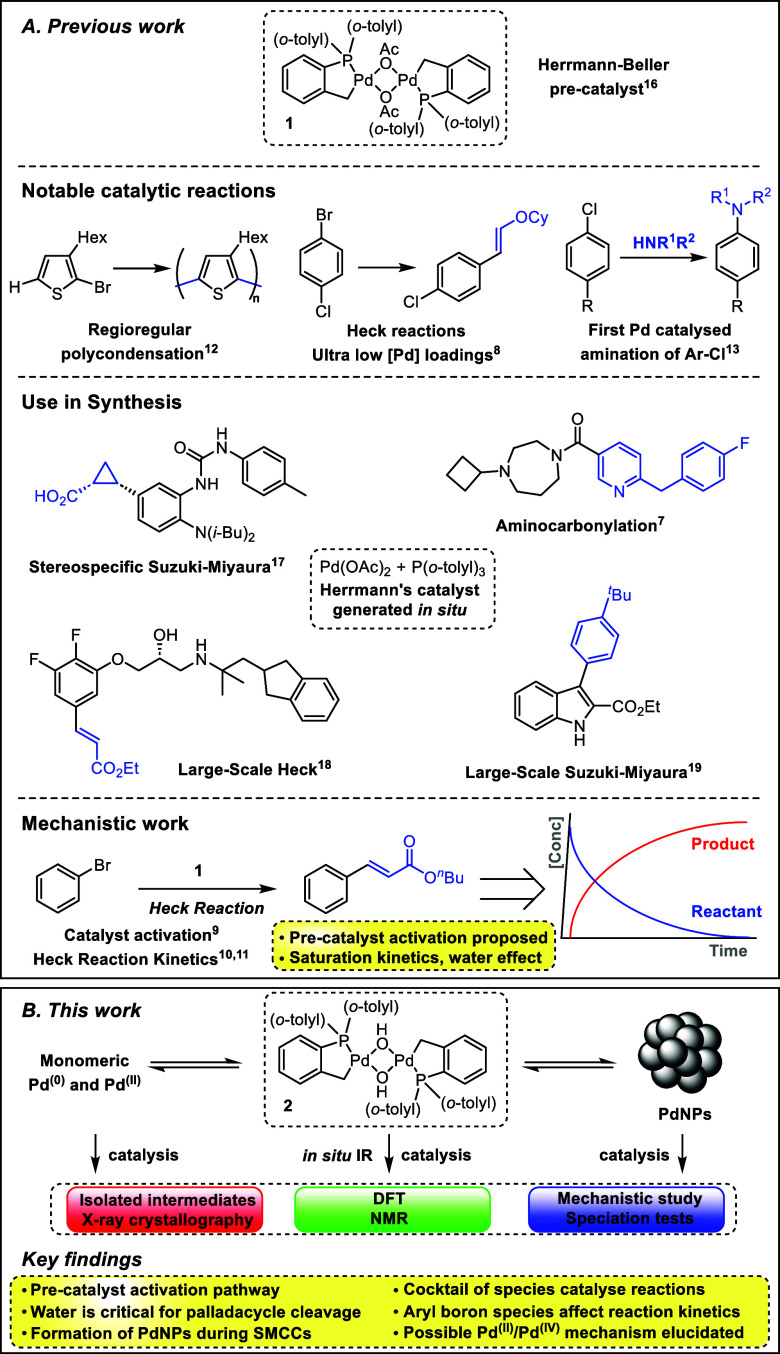
Notable chemical transformations utilizing either the Herrmann–Beller palladacycle (Herrmann’s catalyst) **1** or Pd(OAc)_2_/P(*o*-tolyl)_3_, which generates Herrmann’s catalyst *in situ*, and the focus of this work to probe the [Pd(C^P)(μ_2_-OH)]_2_**2** palladacycle, generated from **1** under basic aqueous conditions.

As one of the most ubiquitous ligands for Pd-catalyzed cross-coupling reactions, P(*o*-tolyl)_3_ has long enjoyed a privileged position. In comparison to other simple phosphines, it is bench-stable, resistant to oxidation, and can be used to perform an eclectic array of challenging cross-coupling reactions under ambient conditions.^1^ A large steric bulk (one of the largest cone angles of all simple phosphines)^2^ enables P(*o*-tolyl)_3_ to form reactive monophosphine-ligated Pd^(0)^ species. High-throughput experimentation and analysis of reaction outcomes for catalytic processes involving P(*o*-tolyl)_3_ show that it is competitive with specialist phosphines in terms of both reactivity and product selectivity.^3^ For example, Burke et al. showed that deuteration of the methyl substituents of P(*o*-tolyl)_3_ can influence branched/linear isomer product ratios in the Suzuki–Miyaura cross-couplings (SMCCs) of deactivated Csp^3^-boronic acids with aryl halides.^4^ The origin of these effects remains largely unclear. The behavior of P(*o*-tolyl)_3_ can be credited to its unusual steric bulk or its penchant to form palladacycles.^5,6^ The discovery of the reaction between Pd(OAc)_2_ with P(*o*-tolyl)_3_ to form stable palladacycles led to one of the first highly active cross-coupling Pd precatalysts for cross-coupling reactions, namely, Herrmann–Beller palladacycle **1**, which is particularly effective for arylative Heck reactions (Figure 1A).^7−13^ While typically employed at high reaction temperatures, ultralow Pd-catalyst loadings can be employed using **1** (with TONs up to 1 × 10^6^).^8,14−16^ Often researchers will employ Pd(OAc)_2_/P(*o*-tolyl)_3_, which could potentially form palladacycle **1***in situ*, for which several transformations have been reported.^17−19^ Mechanistically, the activation of the Herrmann–Beller palladacycle was studied by Jutand et al. at 80 °C in DMF.^9^ It was proposed that precatalyst activation occurs via migration of the acetate anion to phosphorus via a mononuclear Pd^(II)^ complex, involving palladacyclic cleavage.^9^ Prior to this, Blackmond et al. reported a detailed mechanistic study examining the behavior of palladacycles in Heck reactions including **1**.^10,11^ All of this work has been performed under the assumption that **1** activates to an active Pd^(0)^ species, but it was originally speculated by Herrmann and Beller,^16^ and later by Shaw,^20,21^ that palladacycles could remain intact during catalysis; thus, the feasibility of a Pd^(II)^/Pd^(IV)^ redox process was suggested for Heck alkenylation reactions. That said, the higher reaction temperatures of these reactions have led many in the wider catalysis field to conclude that Pd^(0)^ species and associated higher order species dominate in these systems.^22^

By way of further background, Blackmond et al. elegantly demonstrated that water was important for the activation of precatalyst **1**, but the origin of the “water effect” at Pd was not explored further (Figure 1A).^10,11^ On the other hand, Kapdi et al. showed that stabilized Pd colloids formed from **1** are the active catalyst species for the arylation of aldehydes, as well as homocoupling and cross-coupling of aryl boronic acids.^23^

Over the past few years, we have been intrigued by the critical role played by water in the activation of Pd precatalysts. For example, we demonstrated in an agrochemical aryl cyanation reaction that the activation process and Pd-catalyst speciation (clusters formed by aggregation) were dependent on the amount of water present in the reaction system, a finding which was underpinned by kinetic analyses.^24^

A role for water in other cross-coupling chemistries has been reported but rarely explained.^25−27^ Separately, we wished to collect kinetic data on Heck and SMCC reactions to gather valuable information concerning reaction sensitivities, while understanding the formation of both byproducts and side products. Indeed, obtaining reliable kinetic data on SMCC reactions, which are often run under liquid–liquid biphasic conditions (with one liquid phase, typically an aqueous solution of an inorganic base), is often difficult, particularly using *in operando* spectroscopic techniques.^28,29^

Recently, we identified conditions that allow us to operate SMCC reactions in a single liquid phase (the use of THF/H_2_O with ^n^Bu_4_NOH). This enabled the behavior of Pd precatalyst and Pd speciation to be investigated (involving Pd_n_ clusters derived from Pd(OAc)_2_/nPPh_3_).^30−32^ Other solvent/cosolvent/base combinations could therefore enable access to single liquid phases, which is critical for kinetic analysis of SMCCs. With these pillars in place enabling us to reliably probe the kinetic behavior of SMCC reactions, we have focused on uncovering the mechanistic details of Herrmann–Beller palladacycle **1** under working reaction conditions (Figure 1B). Moreover, we anticipated that the knowledge gained from our benchmark reaction system could be translated to a deeper understanding of the mechanism of the Heck reaction and related Pd-catalyzed arylative transformations.

The goals of our study were as follows:

1 Understand the behavior of the Herrmann–Beller palladacycle **1** under aqueous basic conditions.
2) Assess the stability and reactivity of [Pd(C^P)(μ_2_-OH)]_2_**2** as a palladacycle.
3) Assess whether **2** is a (pre)catalyst for SMCC reactions under mild conditions.^33^
4) Develop reaction conditions operating in a single liquid phase enabling SMCC kinetic analyses.
5) Delineation of the activation pathways of **1** and **2** under SMCC working reaction conditions.
6) Gain evidence for competing reaction pathways involving Pd^(0)^/Pd^(II)^ (homogeneous and heterogeneous catalysis) and potential Pd^(II)^/Pd^(IV)^ species.
7) Show that the P(*o*-tolyl)_3_ ligand is more than a simple phosphorus donor ligand for Pd.
8) Bring together a broader picture for the action of P(*o*-tolyl)_3_ ligands involving Pd catalyst species.

## Results and Discussion

### Optimization of a Monophasic Suzuki–Miyaura Reaction and the Kinetic Effects of Precatalyst Concentration

**Scheme 1:**
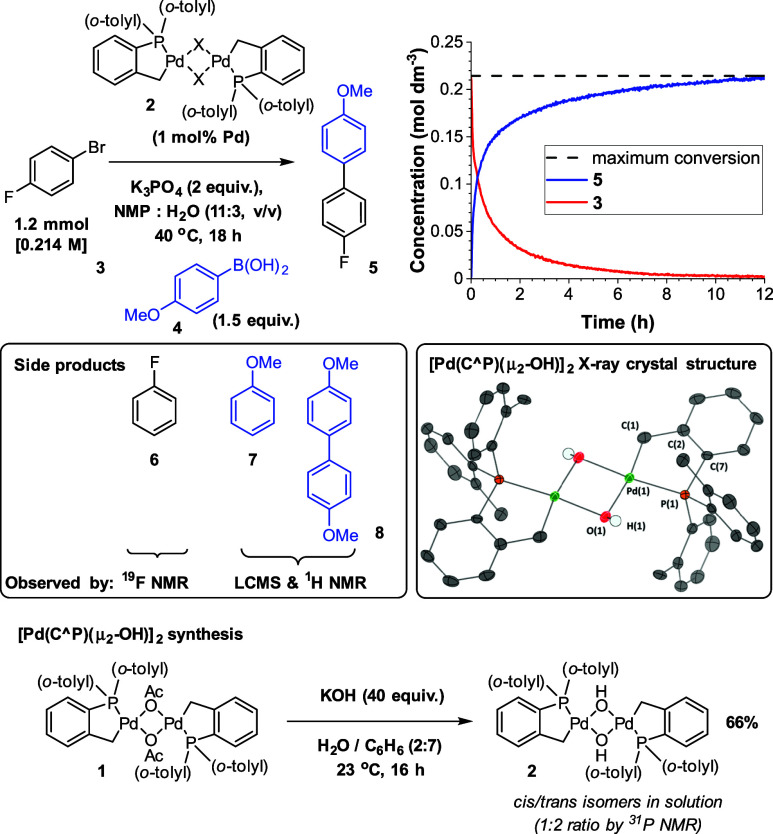
Optimized SMCC Conditions, with Common Side Products Detected by NMR Spectroscopy and LC-MS and the Synthesis of **2** X = OH, Br, I, and OAc, % conv. > 96. *In situ* IR kinetic profile of the SMCC reaction, for palladacycle precatalysts where X = OH (see SI Section 4.2 for X = OAc, Br). Reaction side products detected by NMR spectroscopy and LC-MS. XRD crystal structure of [Pd(C^P)(μ_2_-OH)]_2_**2**, thermal ellipsoids set at 50% probability, and selected H-atoms omitted for clarity.

A benchmark SMCC reaction involving the reaction of 4-fluoro-bromobenene **3** with *p*-anisyl boronic acid **4** to give biaryl product **5** was developed (Scheme 1); reaction conditions were optimized to function in a single liquid phase. Ensuring reagent solubility and homogeneity required a 11:3 (*N*-methylpyrrolidinone (NMP):H_2_O, v/v) solvent ratio (found by experimentation with varying ratios of solvent/cosolvent combinations). Online reaction monitoring was feasible using a Mettler-Toledo ReactIR system (with flexible Ag-halide diamond probe); quantification of starting materials, cross-coupled product, and side-product concentrations was conducted by direct cross-referencing with ^19^F NMR spectroscopic analysis. Under these conditions, the Herrmann–Beller palladacycle **1** gave product **5** with 97% conversion, after being stirred at 40 °C for 18 h under an inert atmosphere (N_2_ or argon). We acknowledge that NMP, being a polar aprotic solvent, could stabilize higher order Pd aggregates.

The related halide derivative of **1** (μ_2_-X = Br) was synthesized and studied alongside the independently synthesized hydroxo-Pd species, [Pd(C^P)(μ_2_-OH)]_2_**2**, by reaction of the Herrmann–Beller palladacycle **1** with KOH in a benzene/water mixture at ambient temperature (66% yield), demonstrating that the bridging acetate anionic ligands are readily exchanged with bridging hydroxide under aqueous conditions.

The rates of product formation for the three precatalysts (Scheme 1, X = OH, OAc, and Br) in SMCCs are almost identical, suggesting a common catalytic pathway. Additionally, ^31^P NMR analysis of the reaction end points determined that two species were present: the bridging Br analogue of **1** (X = Br, ^31^P δ 42 ppm) and an unknown species at ^31^P δ 26.5 ppm (ratio 2.4:1 by ^31^P NMR, see SI Section 7.2). A recharge experiment (see SI Section 4.3) confirmed that these observed species can be brought back online; that is, they are not moribund catalyst species. In keeping with the known behavior of **1** in arylative Heck reactions, [Pd(C^P)(μ_2_-OH)]_2_**2** exhibits an inverse correlation of the reaction rate with catalyst concentration (see SI Section 4.6). That is to say, the overall product conversion at fixed reaction time decreases as catalyst loading increases (up to 10 mol % in **2**). Such a phenomenon has been ascribed to the known aggregation of Pd at higher concentrations, where the aggregated species are inactive.^34^

### Precatalyst Activation by Aryl Boronic Acids Studied Using Low-Temperature NMR: Water Is Found Critical for the Generation of Pd^(0)^ Species

Three commonly observed side products were detected under the reaction conditions, with **7** and **8** (Scheme 1) being the most significant. Stoichiometric studies indicated that [Pd(C^P)(μ_2_-OH)]_2_**2** activates to bicyclic [Pd^(II)^(C^P)_2_] complex **11** and nonphosphine-ligated “Pd^(0)^” species **12** upon exposure to 2 equiv. *p*-anisyl boronic acid **4** (from TGA, 21.8 mol % boroxine in the sample, based on water loss). The presence of Pd^(II)^ complex **11** was determined by ^31^P NMR (single peak at 26.5 ppm, 88% yield) and LIFDI mass spectrometry, which detected a radical cation consistent with the mass of **11** (no other Pd species were observed by LIFDI or ESI mass spectrometry). Pd aggregation, derived from soluble Pd^(0)^ species, was observed in the reaction mixtures (rapid changes from colorless to dark brown/black). When the same reaction was repeated with excess P(*o*-tolyl)_3_, [Pd^(0)^(P(*o*-tolyl)_3_)_2_] was observed (authenticated by comparison to a commercial sample). The ratio of **11** to [Pd^(0)^(P(*o*-tolyl)_3_)_2_] peaks was found to be 1:0.93.^35^ Trapping of Pd^(0)^ by excess P(*o*-tolyl)_3_ provides evidence for a mechanism of activation that generates both bicyclic [Pd^(II)^(C^P)_2_] **11** and nonphosphine-ligated “Pd^(0)^” species **12** in an approximate equal quantity. Further experiments confirmed that **11** was inert to oxidative addition using **3**. By contrast, a reaction of [Pd^(0)^(P(*o*-tolyl)_3_)_2_] with **3** in THF afforded [Pd^(II)^(Br)(C_6_H_4_-*p*-F)(P(*o*-tolyl)_3_)]_2_ (see SI Section 7.6).^35^

**Scheme 2:**
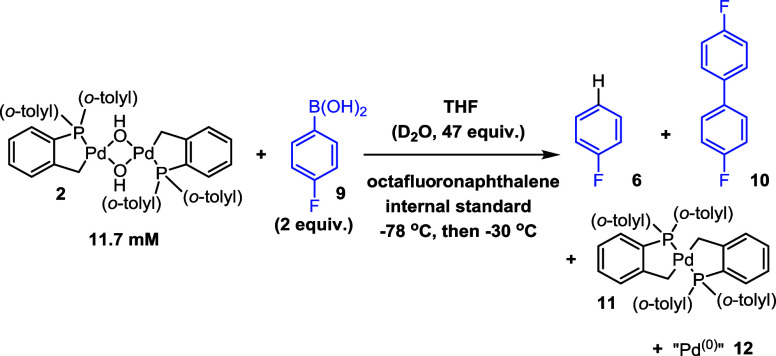
Activation of [Pd(C^P)(μ_2_-OH)]_2_**2** by Aryl Boronic Acid **9** The reactions were carried out in a J. Young NMR tube, prepared at −78 °C. A solution of **9** in THF was added to a solution of **2** and octafluoronaphthalene (internal standard) in THF. The mixture was homogenized by vortexing at −78 °C and then transferred to a precooled NMR spectrometer

**Figure 2:**
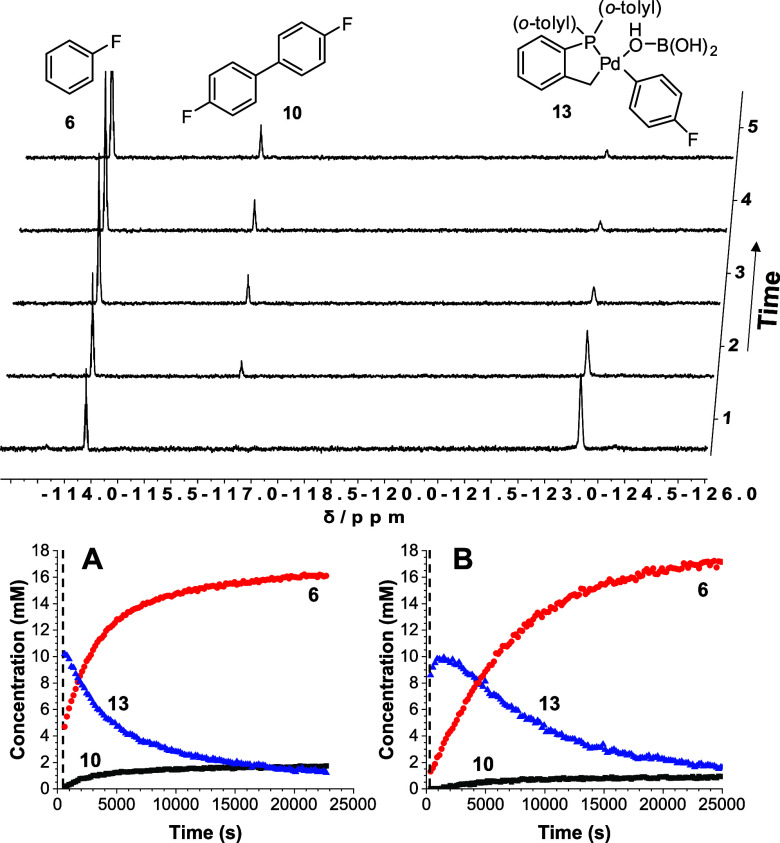
Reaction progression for activation of **2** by arylboronic acid **9** (2 equiv) as shown in Scheme 2 at −30 °C, monitored by ^19^F NMR (470 MHz, THF unlocked, 243 K). NMR time course showing compound identities. (A) reaction according to Scheme 2; (B) reaction according to Scheme 2 with 4 equiv of boric acid additive. Black triangle, Pd transmetalation complex **13**; black circle, fluorobenzene **6**; black square, 4,4′-difluorobiphenyl **10**. Eight scans, relaxation delay = 5 s, and one data point collected at 3 min intervals. The concentration of fluorine-containing species was determined by integration to the internal standard, octafluoronapthalene. First-order rate constants were fitted to *y* = *A*_1_*exp(*x*/*t*_1_) (see SI Section 6.2 and Section 6.3).

Building on the work of Denmark et al.,^36,37^ the mode of activation of [Pd(C^P)(μ_2_-OH)]_2_**2** in generating the active species was investigated (Scheme 2). A reaction of **2** with *p*-fluorophenyl boronic acid **9** was monitored by variable temperature NMR (VT-^19^F NMR, 500 MHz, −70 to 25 °C) (Figure 2). At −55 °C, one major ^19^F peak was recorded at δ ^19^F −123 ppm and one ^31^P peak at δ ^31^P 17.4 ppm, ascribed to mononuclear transmetalation product **13**, which is generated by the arylboronic acid coordinating through the μ_2_–OH group on **2**, then cleaving the dimer species, and undergoing rapid transmetalation (note: ^19^F data comparable to Denmark et al., intermediate **13** has been tentatively assigned due to its similarity in ^19^F chemical shift). It is possible that other coordinating ligands (THF, H_2_O) are present in the place of B(OH)_3_ (modeled computationally *vide infra*). Compounds described later (**16a** and **25**) imply that only the isomer with the aryl group *trans* to the phosphine ligand forms as the more stable species. VT-NMR ^31^P and ^19^F experiments showing the formation and degradation of **13** can be found in SI Section 7.3.^36^ At −30 °C, the formation of fluorobenzene and 4,4′-difluorobiphenyl (δ ^19^F −114.6 and −116.8 ppm, respectively) was confirmed, concomitant with loss of mononuclear post-transmetalation Pd^(II)^ adduct **13** (Figure 2). Reaction of **2** with excess *p*-fluorophenyl boronic acid **9** led to a reaction rate enhancement, with negligible 4,4′-difluorobiphenyl **10** formed (fluorobenzene **6** was the major product, generated in a 9:1 ratio cf., **10**). At lower temperatures (^31^P NMR at the end of reactions), a broad peak was observed in the palladacycle region (δ ^31^P 32–40 ppm), indicating the formation of higher order Pd species in this reaction, possibly as a stepping stone to aggregation, forming PdNPs.

To determine whether residual water from *p*-fluorophenyl boronic acid **9** was responsible for the generation of fluorobenzene **6**, including playing a role in the precatalyst activation step, D_2_O (10 μL, 47 eq w.r.t. [Pd], 9.5 × 10^5^ ppm, added from stock solutions of **2** and **9** in THF at −78 °C) was spiked into an otherwise equivalent reaction. Only **6** was formed under these reaction conditions (rate constant *k*_obs_ = 1.31 × 10^–4^ ± 0.01 × 10^–4^ s^–1^, cf., 2.61 × 10^–4^ ± 0.04 × 10^–4^ s^–1^ for the unchanged reaction). An uncharacterized species at δ ^19^F −112 ppm was observed to degrade over 2.5 h. ^2^H NMR analysis showed significant incorporation of deuterium into the aromatic and aliphatic regions within the ligand framework. Therefore, D_2_O provides protons for fluorobenzene formation and palladacycle Pd–C bond cleavage/protonation.

**Scheme 3:**
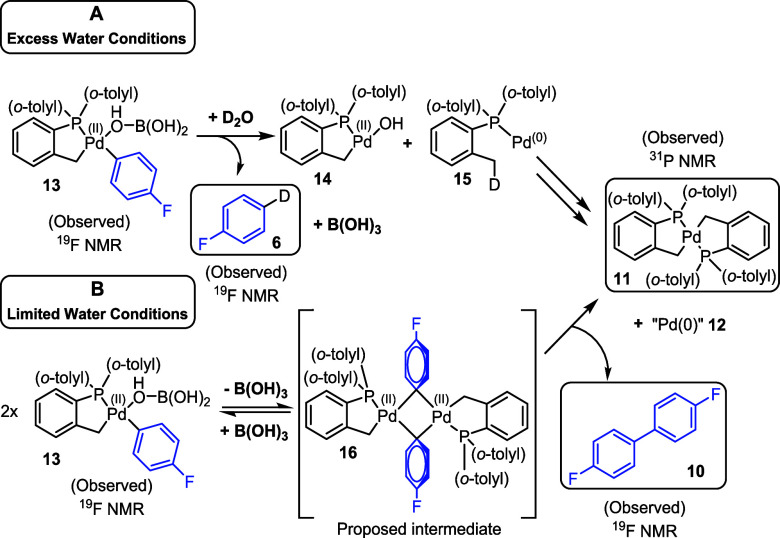
Two Proposed Pathways for the Activation of [Pd(C^P)(μ_2_-OH)]_2_**2** to Pd^(0)^ Complex **12** and Pd^(II)^ Complex **11** with Boronic Acid **9** under (A) Excess Water Conditions and (B) Limited Water Conditions

From our data, two activation pathways can be described (Scheme 3). Under water-limiting conditions (<38 ppm in THF solvent), formation of biaryl **10** is favored, implying that two Pd complexes react after the transmetalation step, potentially via unstable bridging aryl intermediate **16**, although we are unable to exclude other possibilities including Pd^(I)^ intermediates. Homocoupling of the aryl group can then occur, generating bicyclic Pd^(II)^ complex **11** andnonphosphine-ligated “Pd^(0)^” species **12**, with residual water cleaving the palladacycle. Under excess water conditions, the activation process changes, with the water first protonating the aryl group to generate unstable Pd^(II)^ complex **14** (the monomeric species of dimer **2**), alongside fluorobenzene **6**, in a redox–neutral protodeboronation process. Formation of Pd^(0)^ species **15** is tentatively suggested based on the ^31^P NMR spectroscopic data. The precise route to the formation of stable Pd^(II)^ bipalladacycle **11**, observed by ^31^P NMR in several experiments, is unclear.

Additional boric acid (1 equiv w.r.t. [Pd]) was found to retard the reaction rate, cf., the standard reaction similar to D_2_O (fluorobenzene **6** generation rate *k*_obs_ = 1.36 × 10^–4^ ± 0.01 × 10^–4^ s^–1^, cf., 2.61 × 10^–4^ ± 0.04 × 10^–4^ s^–1^), suggesting a stabilizing equilibrium with the boric acid coordinating Pd intermediates, or boric acid acting as a buffer through coordination of bridging OH groups as a Lewis acid.

**Figure 3:**
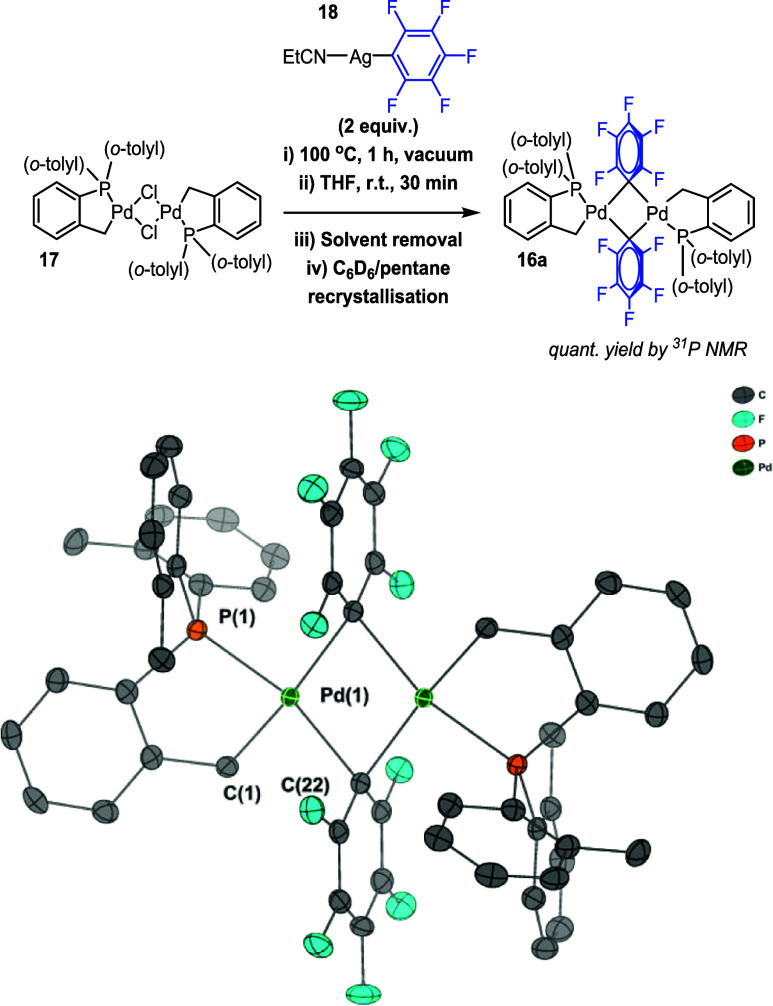
Independent synthesis of [Pd(C^P)(μ_2_-C_6_F_5_)]_2_**16a**. The single-crystal XRD structure is shown, with H-atoms omitted for clarity and thermal ellipsoids set to 50%.

The structure of transmetalated intermediate **16** is supported by literature examples of bridging aryl–Pd complexes.^38^ Stabilized C_6_F_5_ analogue **16a** of this proposed intermediate was successfully synthesized from chloropalladacyle **17** by reaction with **18**, providing evidence that bridging aryl–Pd species could be accessed for this Herrmann–Beller-type palladacyclic framework (Figure 3). **16a** is unstable in water, rapidly decomposing into Pd black and pentafluorobenzene. Note that **16a** exists as a mixture of monomers and dimers in coordinating solvents such as THF (see SI Section 7.8).

### Precatalyst Activation by Arylphosphine Boron Reagents

**Figure 4:**
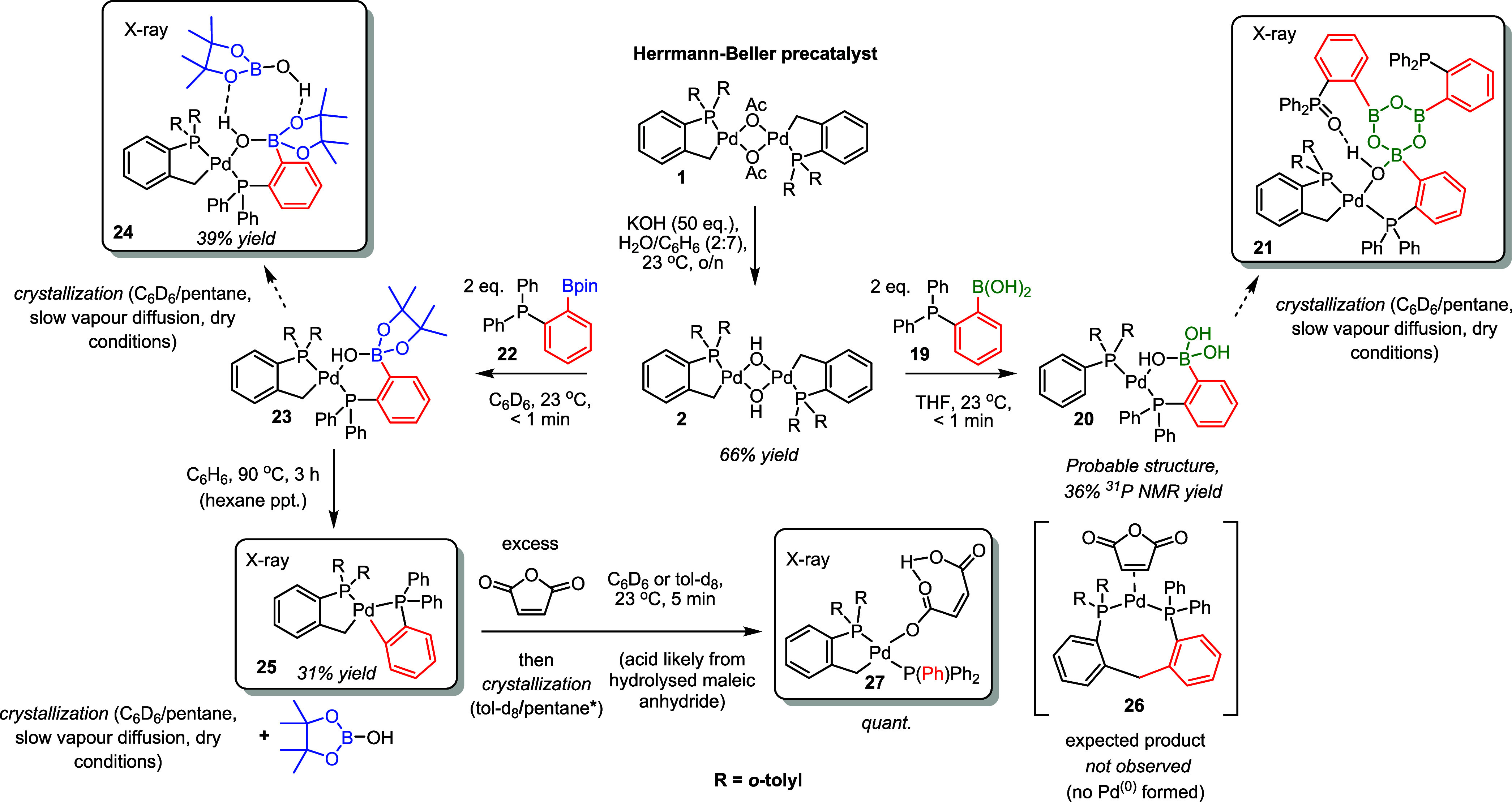
Reaction pathways for [Pd(C^P)(μ_2_-OH)]_2_ palladacycle **2** with phosphine-stabilized boron species.

We next examined the precatalyst activation step and focused on the reaction of [Pd(C^P)(μ_2_-OH)]_2_**2** with aryl boronic acids and pinacol esters (Figure 4). Reported Ph_2_P(C_6_H_4_-*o*-B(OH)_2_) derivative^39^**19** and BPin derivative **22** were selected as aryl boronic tethered phosphines to enhance Pd stabilization and characterization of any downstream intermediates and products.

At room temperature, two equivalents of BPin derivative **22** react rapidly with one equivalent of [Pd(C^P)(μ_2_-OH)]_2_**2** in C_6_D_6_ at 23 °C. Pre-transmetalation complex **23**, possessing a Pd–OH···B interaction, was formed, shown by ^31^P NMR as a change from a single peak at 35 ppm to a roofed pair of AB doublets (^2^*J*_P,P_ = 374 Hz, indicating a *trans* P–P spin–spin coupling). ^1^H NMR spectroscopic analysis shows complete loss of the bridging OH group (δ ^1^H −1.26 ppm). Slow vapor diffusion of a benzene/pentane mixture of **23** led to the crystallization of an adduct possessing one molecule of HO-Bpin. The origin of HO-Bpin can be explained by the subsequent observation, where upon heating, we see formation of highly unusual spiro-*bis*-palladacyclic compound **25** possessing 4-membered and 5-membered palladacycles, connected by a shared Pd atom. It is surprising that the *cis*-disposed Pd carbons do not rapidly reductively eliminate, especially as they have a bond angle of significantly less than 90°. Additionally, the rearrangement of *trans***23** to *cis***25** demonstrates the stability of having aryl groups *trans* to phosphines across a Pd center (even considering a significant steric penalty). However, a high barrier to phosphine dissociation might explain the apparent stability of compound **25**. Nevertheless, we hypothesized that an exogenous π-acidic olefin such as maleic anhydride or fumaronitrile would encourage reductive elimination, generating a thermodynamically stable phosphine-ligated Pd^(0)^ complex.^40^ This was not observed in either case. There was no reaction with fumaronitrile, whereas residual water in the maleic anhydride (forming maleic acid) caused spiro-*bis*-palladacycle **25** to cleave, bringing about the formation of stable Pd^(II)^ complex **27**. The unexpected stability of spiro-*bis*-palladacycle **25** and its resistance to reductive elimination, even at high temperatures (up to 90 °C for 5 h), implies that the activation of these *o*-tolyl palladacycles by reductive elimination onto the ligand backbone possesses a high activation barrier. Hartwig and Louie showed that **1** activates to Pd^(0)^ upon heating with Me_3_SnPh (via Csp^3^–Csp^2^ reductive elimination onto the P(*o*-tolyl)_3_ ligand).^41^ The elimination of **25** to **26** could be expected to follow in a similar fashion but does not. There is instead a requirement for protonolysis for precatalyst activation via cleavage of the transmetalated aryl group. We recognize that the phosphino group could be hindering Csp^2^–Csp^3^ reductive elimination.

Of particular note is that when impure Ph_2_P(C_6_H_4_-*o*-B(OH)_2_) boronic acid variant **19** (known to form boroxines)^39^ was used, the XRD crystal structure obtained of the product was of boroxinate complex **21**, indicating that boroxines can coordinate to the precatalyst directly; they do not necessarily need to be hydrolyzed to the aryl boronic acid. From these studies, we show that pinacol esters can readily undergo direct transmetalation without the need for hydrolysis of the free acid, in agreement with Denmark’s findings.^36^

### Pinacol Effects in SMCC Reactions: Role of Pd Nanoparticles

**Figure 5:**
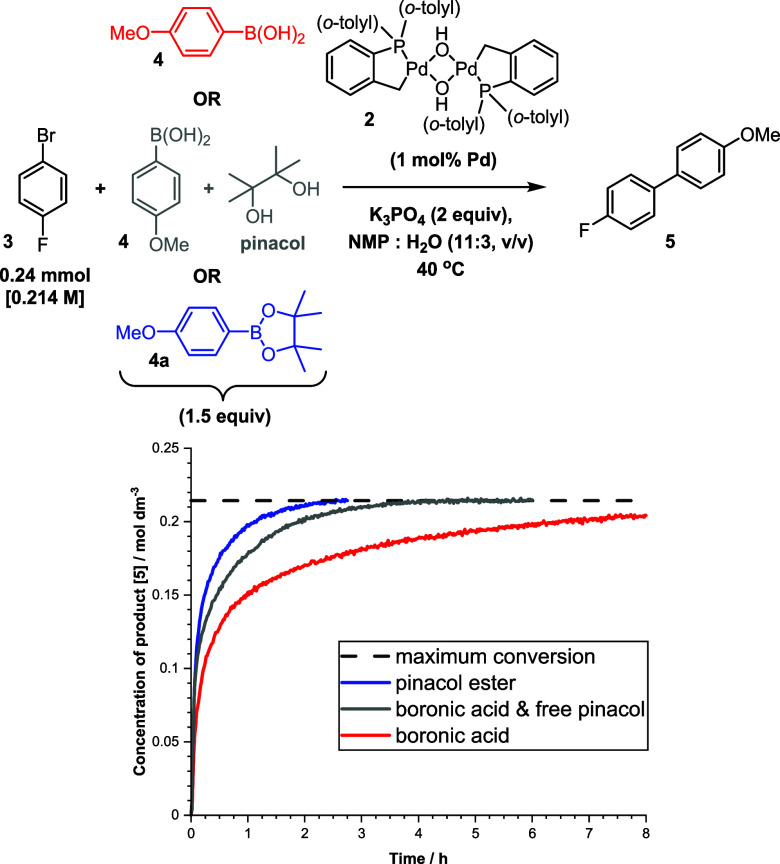
Kinetic profiles for formation of **5** under standard conditions with [Pd(C^P)(μ_2_-OH)]_2_**2** (red, boronic acid **4**, described in Scheme 1, and for the same reaction with the corresponding pinacol ester **4a** replacing the boronic acid (blue), and added boronic acid **4** with pinacol (1 equiv) (gray)). Reactions were monitored with *in situ* IR (diamond probe).

Returning to the catalytic reactions, we wished to assess any difference in reactivity between aryl boronic acid **4** and aryl pinacol ester **4a** in reaction with **3** (Figure 5). Transmetalation reactions of pinacol esters are typically slower than those of boronic acids, which are delivered by hydrolysis. First, aryl pinacol ester **4a** led to a reaction rate enhancement compared to **4**.^36,42,43^ Addition of free pinacol to a reaction involving **4** also resulted in an increase in the reaction rate, but to a less significant extent than with **4a** alone. This could be due to increased solubility relative to **4** or stabilizing interactions of free pinacol at the Pd center(s).

**Figure 6:**
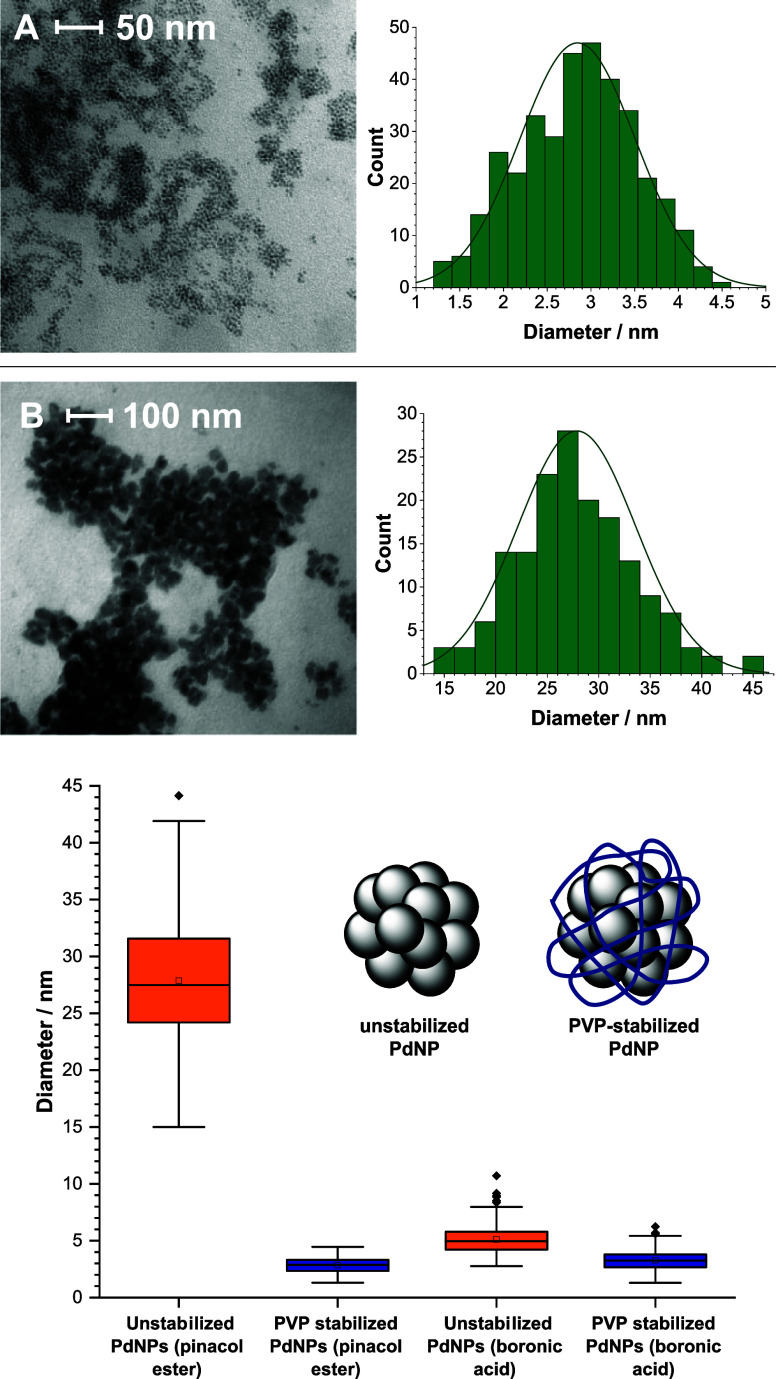
Transmission electron microscopy (TEM) images of PdNPs isolated from a Suzuki reaction employing pinacol ester. The samples were taken at approximately 70% conversion to product. (A) PdNPs stabilized with polyvinylpyrrolidone (PVP) polymer (*M*_W_ = 29000), with distribution of nanoparticles by size; (B) PdNPs from the same reaction without a stabilizing agent. A bBox and whisker plots showing the distribution of PdNP diameters from boronic acid and pinacol ester reactions.

We have considered that the pinacol or arylboron species could influence the Pd catalyst structure, shifting the reaction from an assumed homogeneous catalytic cycle to a heterogeneous Pd nanoparticle (PdNP)-catalyzed reaction. Analysis of an SMCC reaction employing pinacol ester **4a** by TEM, using a method developed by our group,^44^ revealed a significant quantity of PdNPs generated *in situ*. As the analysis involves removing solvent from a reaction sample *in vacuo* under forcing conditions, a stabilizing PVP polymer was added to prevent further Pd aggregation during this step, from which the TEM images of stabilized/nonstabilized PdNPs were then compared. As can be seen in Figure 6, there is a narrow distribution and small size of these PVP–PdNPs, associated with high catalytic activity. However, nonstabilized PdNPs from a reaction employing pinacol ester **4a** were found to be ∼10× larger with a wide size distribution. This indicates that pinacol (either free or in the form of pinacol ester) has a destabilizing effect on the surface of the PdNPs, creating highly active catalyst surfaces. To verify this, further exploration of the standard SMCC reaction (with aryl boronic acid **4**) revealed PdNPs of similar size distribution, with a much smaller variation between PVP-stabilized and nonstabilized PdNPs. This indicates that aryl boronic acids play an active role in stabilizing PdNPs^45^ but generate a less active catalytic system overall. As PdNPs are less prone to aggregation, they can be considered as less catalytically active than those found in the pinacol system.

PdNPs that derive from palladacycle **1** are known and under oxidative (air) conditions are effective at homocoupling aryl boronic acids, which could be another pathway for side-product formation in this system.^23^ The formation of homocoupled side product **8** (typically ∼10% impurity, derived from aryl boronic acid **4**) provides another indication that PdNPs are active in this reaction.

**Figure 7:**
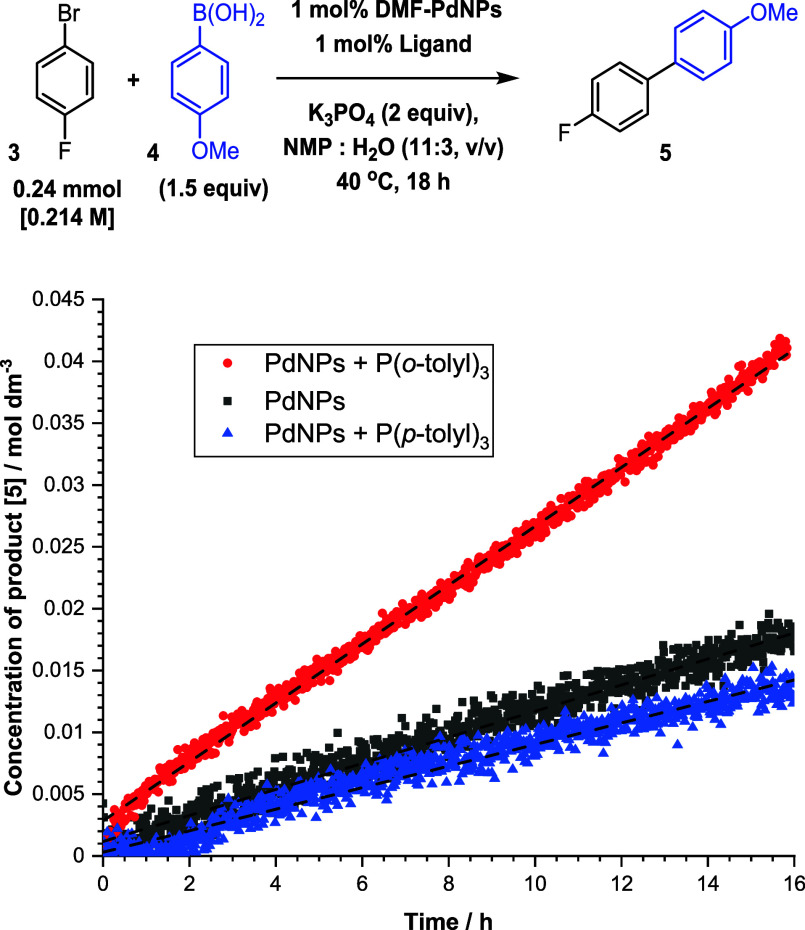
Kinetic profiles for the formation of 4-(4-fluorophenyl)anisole **5** under standard reaction conditions using synthesized DMF-stabilized PdNPs (1 mol %) with and without P(*o*-tolyl)_3_ and P(*p*-tolyl)_3_.

DMF-stabilized PdNPs were independently synthesized according to the literature^46,47^ and added to a standard SMCC reaction at 0.1 mol % catalyst loading, but curiously, no reaction was observed (Figure 7). At 1 mol % loading, 8% conversion to product was achieved after 18 h. This could be improved to 20% by addition of 1 mol % P(*o*-tolyl)_3_. The kinetics for product formation for both reactions were linear (after 0.5 h), suggesting a potential role for a surface-catalyzed SMCC reaction. High-scan ^31^P NMR (57344 scans, concentration of P(*o*-tolyl)_3_ = [4.3 × 10^–4^ M]) revealed the presence of [Pd(C^P)(μ_2_-Br)]_2_**35** and [Pd^(II)^(C^P)_2_] complex **11**, showing that the P(*o*-tolyl)_3_ ligand is capable of detaching Pd or capturing leached Pd from the surface of PdNPs. A control reaction with P(*p*-tolyl)_3_ (unable to form 5-membered palladacycles and showing no substantial improvement over PdNPs alone) demonstrates that the palladacycle forming ability of P(*o*-tolyl)_3_ is crucial to catalytic activity, likely through palladacyclic leaching of Pd from the PdNPs in the system.

**Scheme 4:**
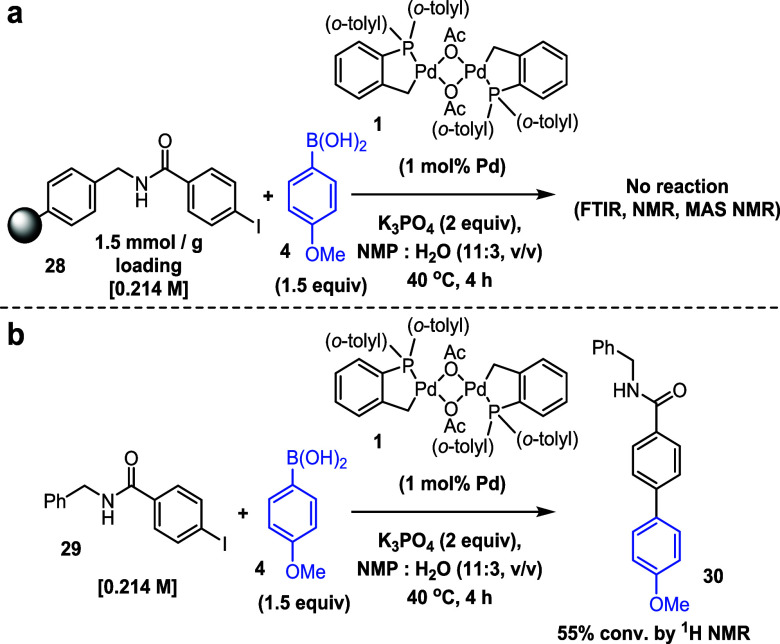
Reaction Scheme for a Three-Phase Test Using an Aryl Iodide Immobilized on Insoluble Resin **28** No reaction was observed by ^1^H NMR spectroscopic analysis (with reference to trimethoxybenzene internal standard), and the recovered resin showed no change in the FTIR spectrum. ^b^ Reaction scheme for the SMCC of related nonresin bound aryl iodide **29** (as a control).

A three-phase test utilizing an aryl iodide immobilized onto polystyrene resin **28** indicated heterogeneous behavior for both aryl pinacol ester and aryl boronic acid (no conversion observed by ^1^H NMR w.r.t. an internal standard; the resin remained unchanged as indicated by FTIR analysis; magic-angle spinning (MAS) ^13^C NMR showed that the immobilized aryl iodide remained unreacted), implying that the reaction proceeds via a path involving stabilized PdNPs (Scheme 4). This result was supported by a control reaction using a related nonresin bound substrate **29**, which showed that [Pd(C^P)(μ_2_-OAc)]_2_**1** was active in the reaction. Although Figure 7 indicates that monomeric Pd leaching from PdNPs occurs under the reaction conditions, it should be noted that this is a slow process with sterically readily available substrates. As such, the use of **28** facilitates the rapid formation of PdNPs due to the availability of **4** (rapidly generating monomeric Pd^(0)^ and inactive **11**, which aggregates due to the lack of a readily available aryl halide substrate).

**Figure 8:**
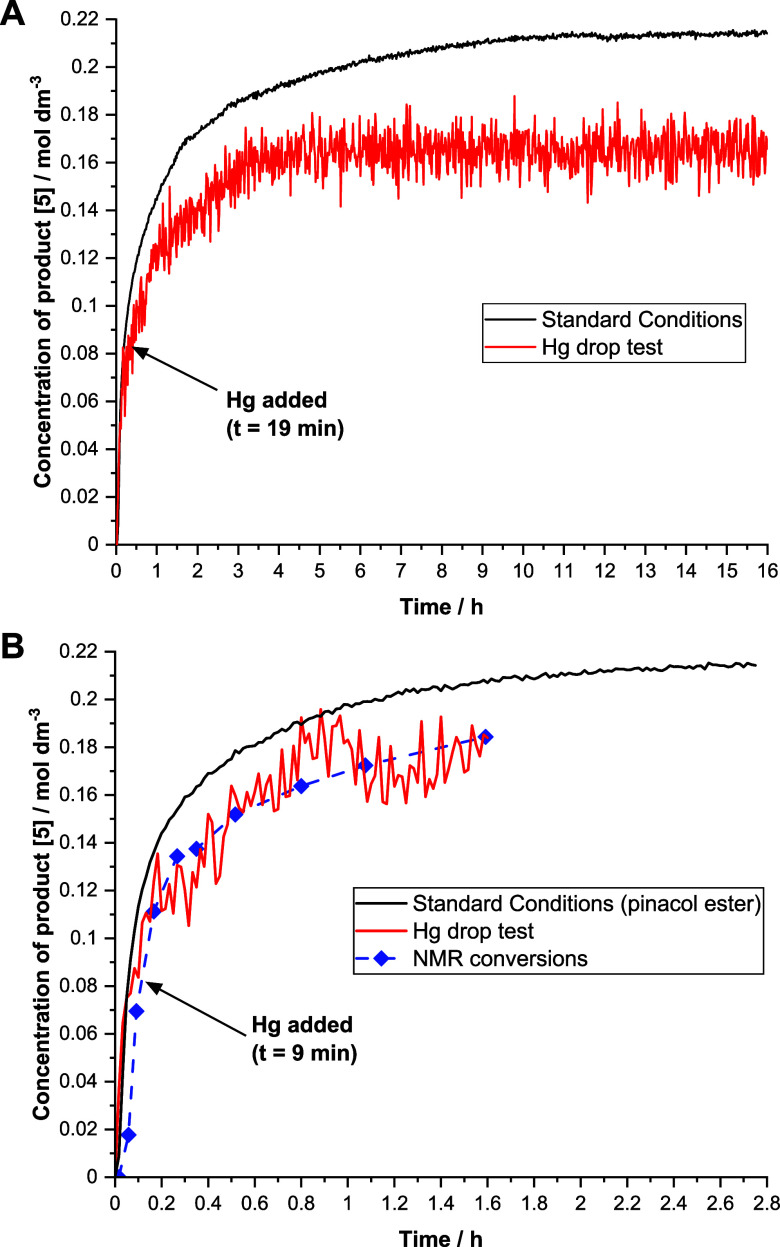
Kinetic profiles for the formation of 4-(4-fluorophenyl)anisole **5** under standard reaction conditions and with 300 equiv of Hg added (relative to catalyst). Kinetic profiles for the formation of 4-(4-fluorophenyl)anisole **5** under standard reaction conditions employing pinacol ester **4a** and with 300 equiv of Hg added. NMR conversions (^19^F) were also taken to provide the complementary data. The Hg drop test data were collected using a fixed Si-probe mirrored conduit (ReactIR) which is more compatible with the reaction conditions employing Hg.

To complement the three-phase test, a Hg drop test was used to test for PdNPs. Previous work has shown that Hg undergoes transmetalation with palladacycles, which can lead to inconsistent results in cross-coupling catalysis.^48,49^ Here, Hg was added at ∼50% conversion or separately prior to the addition of palladacycle **1**. In both cases, the reaction halted at 75% conversion but was initially uninhibited (Figure 8; also see SI Section 4.7). The implication is that lower order Pd is the main catalyst species for the first part of the reaction, but as the reaction nears 75% conversion, conditions change to favor PdNP formation, which acts as a less active catalyst for the remainder of the reaction (either through Pd leaching or directly via Pd-surface catalysis). This fits with the change in the kinetic profile observed through the reaction, taken together with the outcome of the DMF–PdNP-catalyzed control reactions. This test was also performed on the system employing pinacol ester **4a**, with similar results on a shorter time scale. This corroborates other data suggesting that pinacol ester also forms PdNPs at longer reaction times but strongly implies that the pinacol group is influencing catalysis in the homogeneous manifold with mononuclear Pd.

With regard to the previous three-phase test, if the immobilized aryl halide has restricted access to lower order soluble Pd, the reaction conditions mimic those toward the end of the reaction, that is, low [Ar–Br], favoring formation of PdNPs. This evidence supports PdNPs being present and active in this case. We note that our results from these experiments contradict the hypothesis concerning the stoichiometric transmetalative interference of palladacycles with Hg relating to catalytic conditions as reported,^48,49^ which we attribute to different reaction conditions. Nevertheless, we agree that the Hg drop test should be used with caution with adequate controls in place.

### A Pd^(II)^/Pd^(IV)^ Pathway for SMCC Reactions Using Palladacyclic Precatalysts Has Been Proposed Using Stoichiometric Reactions and Computational Chemistry

[Pd(C^P)(μ_2_-C_6_F_5_)]_2_ palladacycle **16a** was shown to be unstable with respect to water, as the addition of excess water to a sample in C_6_D_6_ caused rapid degradation and generation of pentafluorobenzene **33** (observed by ^1^H and ^19^F NMR, respectively). The ^31^P NMR spectrum showed a mixture of [Pd(C^P)(μ_2_-OH)]_2_**2** and what was likely bicyclic [Pd^(II)^(C^P)_2_] complex **11**, showing that water can either displace the bridging Ar^F^ ligand entirely or cause activation to Pd^(0)^ via palladacycle cleavage.

**Scheme 5:**
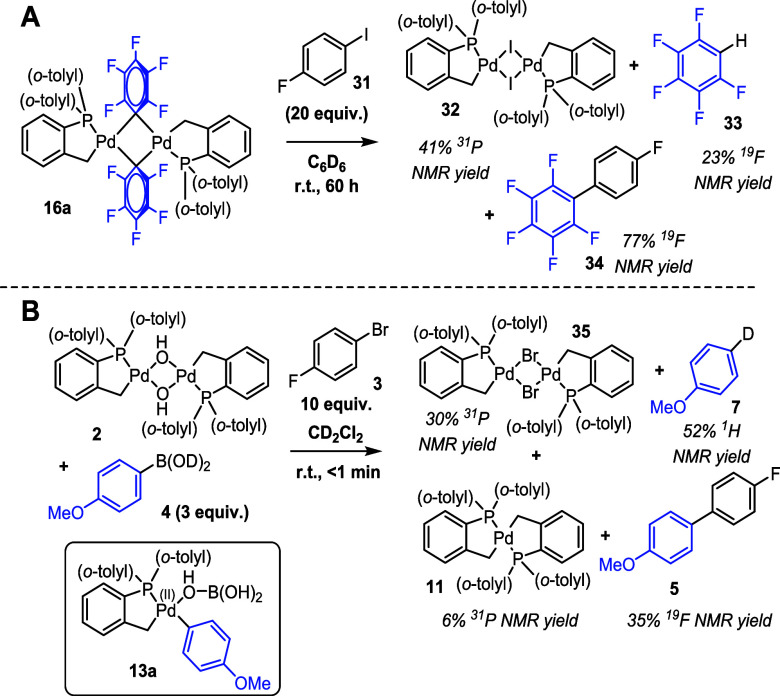
(Reaction Scheme for the Cross-Coupling of Pd_2_(μ_2_-Ar^F^)_2_ Palladacycle **16a** with 1-Iodo-4-Fluorobenzene **31** Uunder Anhydrous Conditions Reaction scheme for cross-coupling utilizing Pd_2_(μ_2_-OH)_2_ palladacycle **2** with 1-bromo-4-fluorobenzene **3** and deuterated arylboronic acid **4** under anhydrous conditions

Interestingly, in the presence of aryl halide **31**, [Pd(C^P)(μ_2_-C_6_F_5_)]_2_**16a** slowly cross-couples, forming [Pd(C^P)(μ_2_-I)]_2_ palladacycle **32**, which was confirmed by LIFDI mass spectrometry and X-ray crystallography (Scheme 5A). A small amount of **33** suggests that trace water was present, facilitating activation to Pd^(0)^. An unknown phosphorus species at δ ^31^*p* = 29 ppm was observed upon addition of aryl halide **31**. Over time, this complex disappeared concurrently with the formation of cross-coupled product **34** (confirmed by reference to an authentic sample) and [Pd(C^P)(μ_2_-I)]_2_**32** (see SI Section 7.9).

For [Pd(C^P)(μ_2_-OH)]_2_**2**, upon activation with arylboronic acid **4** in the presence of aryl halide **3**, ^31^P NMR spectroscopic analysis revealed that stable [Pd(C^P)(μ_2_-Br)]_2_ palladacycle **35** was formed rapidly in appreciable quantities. ^19^F NMR revealed a significant quantity of cross-coupled product **5** and trace amounts of fluorobenzene **6** (likely formed from palladacyclic cleavage and reformation by reductive elimination). Additionally, a high-scan ^2^H NMR spectrum (38912 scans, [deuterated **4**] = 0.032 M) revealed that there was no deuterium incorporation into the palladacycle or fluorobenzene, but there was a significant amount of incorporation into the anisole **7** (deuteriodeboronation) side product. This indicates that either the generated boric acid or residual water is able to cause the protonolysis of complex **13a** (analogue of **13** with C_6_H_4_-*p*-OMe, see Scheme 3 and inset structure of **13a** in Scheme 5B), generating observed [Pd^(II)^(C^P)_2_] bipalladacycle **11** and anisole **7**. Some side products were formed, with the main species being cross-coupled product **5**, as depicted in Scheme 5B.

These results raised the interesting possibility that a Pd^(IV)^ intermediate could be involved in SMCC under the reaction conditions. Rather than activation to Pd^(0)^ species by aryl boronic acid **4**, then oxidative addition by aryl halide **3,** followed by palladacycle reformation by elimination of fluorobenzene **6**, instead oxidative addition could occur directly on Pd species **13a**, generating transient Pd^(IV)^ complex **36** with a square-based pyramidal geometry (with a vacant coordination site, possibly stabilized by solvent or boric acid). Complex **36** is primed to undergo reductive elimination and generate cross-coupled product **5**, generating palladacyclic [Pd(C^P)(Br)] monomer **35a**, which would rapidly dimerize to form [Pd(C^P)(μ_2_-Br)]_2_**35**. This mechanism circumvents the need for water or boric acid to cleave the palladacycle (which under reaction conditions would be a side reaction generating [Pd^(II)^(C^P)_2_] bipalladacycle **11** and nonphosphine-ligated Pd^(0)^**12**, accounting for PdNP formation) and accounts for all the observed species. Alternatively, it is possible that a small quantity of Pd^(0)^ could form and undergo oxidative addition and then transmetalation with **16a**/**13a** in a catalytic manner (as described computationally by Echavarren using simple models in Heck couplings),^50^ which would generate the cross-coupled product. This pathway would be enhanced by the presence of water (shown to degrade **16a**) but would likely generate significant Pd aggregates as a consequence, which were not observed. Previously proposed activation pathways for **1** tend to require ligand modification (via reductive elimination breaking the Pd–C palladacycle),^9,41^ but the products of these pathways are not observed here.

With these results, we recognize that it is likely the same mechanism that occurs for the cross-coupling of [Pd(C^P)(μ_2_-C_6_F_5_)]_2_ palladacycle **16a** with aryl halide **31** (Scheme 5). Stoichiometric experiments showed that [Pd(C^P)(μ_2_-Br)]_2_**35** can undergo rapid ligand exchange with a hydroxide base in an NMP/water mixture to reform [Pd(C^P)(μ_2_-OH)]_2_**2**, allowing for a catalytic process involving Pd^(IV)^ intermediates (where transmetalation occurs first) and accounting for observed [Pd(C^P)(μ_2_-Br)]_2_**35** at the reaction end point.

**Figure 9:**
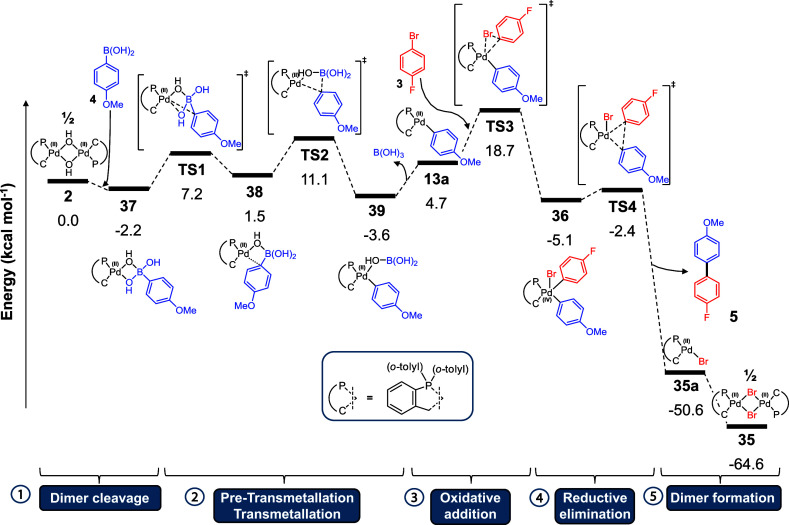
Proposed reaction pathway showing the generation of cross-coupled product **5** from the reaction of 1-bromo-4-fluorobenzene **3** with aryl boronic acid **4**. DFT calculations were performed at the B3LYP/def2svp level of theory with a superfine integration grid (gas-phase) for optimization; then, single-point energy calculations were conducted at the B3LYP/def2tzvpp level of theory, employing an SMD implicit solvent model (using dichloromethane) and Grimme’s third empirical dispersion correction with Becke–Johnson damping.

To rationalize the experimental observations, computational studies using DFT methods were used to model the intermediates and species that are likely involved in the activation of [Pd(C^P)(μ_2_-OH)]_2_**2** (Figure 9). The pathway begins with cleavage of **2** to **37** by aryl boronic acid **4**. **37** then undergoes rearrangement to **38**, which has the C_ipso_ carbon coordinated to the Pd center in a stabilizing interaction. Pre-transmetalation complex **38** then undergoes formal transmetalation (TS2) to afford **39**. These calculations were based on reported structures and transition states on a similar structure using an arylboronic acid employed by Denmark et al.^36^**39** then loses the stabilizing boric acid, which is removed from subsequent calculations. **13a** contains a vacant coordination site due to this loss and is calculated to undergo oxidative addition with aryl halide **3**. Up until this point, the energy span of the calculated structures and transition states is low (<14.7 kcal mol^–1^ between structures) which is consistent with rapid processes even at low temperatures. The thermodynamic resting state of **39** is consistent with experimentally observed transmetalation complex **13** (by low-temperature ^19^F NMR in the absence of aryl halide, see Figure 2). The key oxidative addition step (TS3) has an energy barrier of 14.0 kcal mol^–1^, giving an energetic span δE (the difference between the summit and preceding trough of the energy surface, as defined by Shaik and Kozuch)^51^ of 22.4 kcal mol^–1^, which is consistent with an energetically feasible process at room temperature. Proposed Pd^(IV)^ intermediate **36** undergoes an (effectively barrierless) reductive elimination step (TS4), generating cross-coupled product **5** and palladacyclic [Pd(C^P)(Br)] monomer **35a**. This complex can then dimerize to form [Pd(C^P)(μ_2_-Br)]_2_**35** as a thermodynamic sink.

The pathway calculated using DFT methods is feasible at room temperature and accounts for the observed products of the reaction. To further corroborate the reported calculations, single-point energy calculations were run using different functionals (B3LYP δE = 22.4 kcal mol^–1^, PBE0 δE = 15.5 kcal mol^–1^, B3PW91 δE = 19.6 kcal mol^–1^, and LC-ωPBE δE = 35.9, see SI Section 10 for more details). The LC-ωPBE long-range corrected functional appears to overestimate the energy of Pd^(IV)^ complexes in comparison to the other hybrid functionals, giving an unfeasibly large energetic span. It should be noted that only implicit solvent modeling was used here, and the impact of explicit solvents on polar molecules can be significant, often reducing energy barriers significantly.^52,53^ As the experimental SMCC is carried out in a highly polar mixture of NMP/H_2_O, it is probable that a Pd^(II)^/Pd^(IV)^ pathway is more energetically favorable than calculated.

**Scheme 6:**
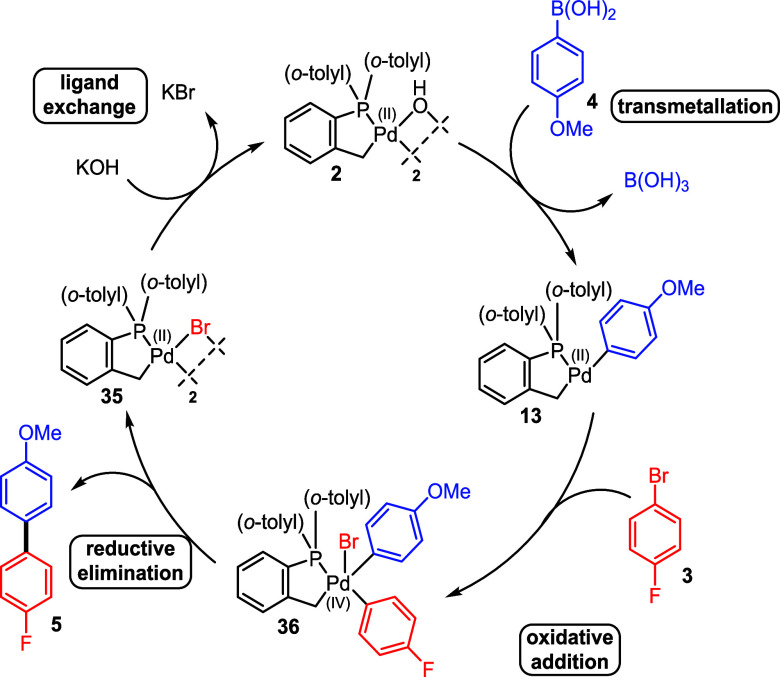
Proposed Catalytic Pd^(II)^/Pd^(IV)^ Cycle Based on Stoichiometric Cross-Coupling Experiments and Calculated Reaction Pathway Energies

Overall, the DFT calculations show that a Pd^(II)^/Pd^(IV)^ process is feasible and can be involved in this reaction, accounting for all of the observed species (Scheme 6). It should be noted that Pd^(0)^/Pd^(II)^ pathways are possible for this particular transformation, which have been extensively computed by Harvey and Yaman under similar conditions.^54^ However, a traditional Pd^(0)^/Pd^(II)^ pathway would require the cleavage and subsequent reformation of the Pd–C palladacycle, leading to species that are not observed experimentally in this case (Scheme 5).

From this collection of stoichiometric and catalytic experiments, it can be concluded that many Pd species are present during an SMCC reaction under these conditions. The idea of a “catalytic cocktail” was first proposed by Ananikov et al., with the evolution of PdNPs during a reaction and the subsequent leaching of mononuclear Pd species proposed for being responsible for sigmoidal kinetics.^55−58^ These dynamic reactions are complex, as all of the considered Pd sources (PdNPs, bulk Pd, that is, Pd black, and molecular Pd species) can be catalytically active, causing issues with selectivity and stability under reaction conditions.

**Scheme 7:**
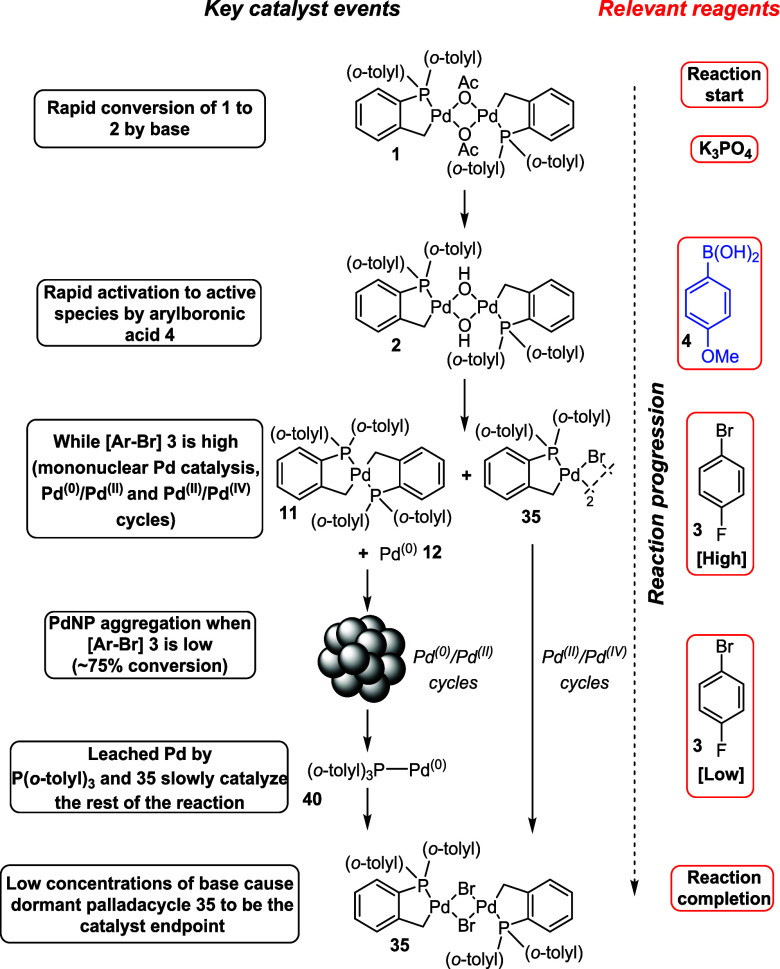
A “Cradle-to-Grave” Representation of PreCcatalyst **1** to Inactive PdNPs and Palladacycle **35**, with the Key Steps and Contributions from Reagents Present in the SMCC

In this case (Scheme 7), the evolution of the Pd species begins with the conversion of Herrmann–Beller palladacycle **1** to [Pd(C^P)(μ_2_-OH)]_2_**2** by a base in the system. This complex is in turn activated by homocoupling (and potentially involving protodeboronation as an associated process) aryl boronic acid **4**, generating catalytically active nonphosphine-ligated Pd^(0)^ species **12**, inactive [Pd^(II)^(C^P)_2_] bipalladacycle **11**, and [Pd(C^P)(C_6_H_4_-*p*-OMe)] **13a** (rapidly reacting to form [Pd(C^P)(μ_2_-Br)]_2_**35)**, which are responsible for cross-coupling reaction involving both Pd^(0)^/Pd^(II)^ and Pd^(II)^/Pd^(IV)^ cycles.

From the experimental evidence using PdNPs as catalysts, it is likely that the mononuclear Pd^(II)^/Pd^(IV)^ cycle (alongside a traditional Pd^(0)^/Pd^(II)^ cycle) dominates the reaction (because PdNP processes are slow). However, as the reaction proceeds and [aryl halide] depletes, PdNPs begin to form through aggregation and catalysis slows. The liberated P(*o*-tolyl)_3_ facilitates leaching of Pd^(0)^[P(*o*-tolyl)_3_] **40** species from these nanoparticles and could continue catalysis slowly, or alternatively, the Pd^(II)^/Pd^(IV)^ cycle continues at a decreasing rate. The depletion of base toward reaction completion results in a buildup of [Pd(C^P)(μ_2_-Br)]_2_**35**, which is spectroscopically observed at the end of the reaction.

Overall, this reaction contains a series of well-defined catalytically active Pd species, which influence the rate of reaction due to different relative activities toward cross-coupling.

## Conclusion

The precatalyst activation of the Herrmann–Beller palladacycle **1** via its derivative [Pd(C^P)(μ_2_-OH)]_2_**2** has been studied by *in situ* reaction monitoring and by crystallization of phosphine-stabilized palladium intermediates. The activation mode has been found to be dependent on arylboronic acid homocoupling (generating Ar–Ar). The results associate protodeboronation with the generation of the active Pd species. The role of water in this process was found to be critical, with deuterium incorporation in both the ligand and byproducts demonstrating that water is at least one requirement for cleavage of the Pd–C bond leading to the generation of the Pd^(0)^ species. [Pd(C^P)(μ_2_-Br)]_2_**35** has been determined to be a key catalytic resting state during the SMCC reaction for an energetically feasible Pd^(II)^/Pd^(IV)^ cycle, which would allow the electron-rich palladacycle functionality to remain intact during the activation step and throughout catalysis. [Pd(C^P)(μ_2_-Br)]_2_**35** has been shown to be able to re-enter the catalytic cycle when the concentration of hydroxide anion is high but becomes dormant at low base concentrations. The presence of PdNPs in the reaction has been explored, and their activity has been verified by heterogeneous catalysis tests (i.e., three-phase test, Hg drop test, and use of PdNPs as catalysts). Experiments have shown that monomeric Pd species can be leached from PdNPs through the action by P(*o*-tolyl)_3_. It is proposed that this mechanism for leaching is at least partly responsible for catalytic activity during the final stages of the reaction. *In situ* reaction monitoring has demonstrated an effect of the arylboron species on the reaction rate, which has implications for the catalyst identity and activity.

The take-home messages from our study are listed as follows:

- Herrmann–Beller palladacycle **1** is readily converted to its derivative [Pd(C^P)(μ_2_-OH)]_2_**2** by reaction with an aqueous base.
- A cocktail of catalytic species (mono- and dinuclear Pd) is generated from Herrmann–Beller palladacycle **1** (presumed to be precatalyst species), under typical SMCC reaction conditions.
- [Pd(C^P)(μ_2_-Br)]_2_**35** is a key catalytic resting state under the conditions examined for the SMCC reaction.
- Under appropriate reaction conditions, Herrmann–Beller palladacycle **1** can in principle access a catalytic cycle involving Pd^(II)^/Pd^(IV)^ species, as originally speculated by Shaw for the Heck reaction.^20,21^ Reactions run under mild conditions (e.g., ∼20–40 °C) enable the palladacyclic structure to be retained under these milder working reaction conditions.
- Water and aryl boronic acid homocoupling play a critical role in the generation of Pd^(0)^ species from palladacycle **1**.
- Exogenous P(*o*-tolyl)_3_ can leach Pd species from PdNPs *via* cyclopalladation, although the catalysis by PdNP species is sluggish under the reaction conditions used.

This study has highlighted that the SMCC reaction mediated by ubiquitous palladacycle **1** is complicated by Pd speciation events, involving Pd^(0)^/Pd^(II)^, potential Pd^(II)^/Pd^(IV)^, and higher order Pd_n_ species. All of these species are catalytically competent. Therefore, the involvement of several catalytic species and cycles has been revealed, particularly under milder reaction conditions employing palladacycle **1**. We recognize that further work is required to experimentally observe and characterize the proposed Pd^(IV)^ species and pathways in this work, but we are confident that such mechanisms are possible (supported by computational calculations and informed by experimental observations that are available). Furthermore, the P(*o*-tolyl)_3_ ligand cannot be considered as a simple 2-electron phosphine donor. As we have shown, it can clearly undergo “molecular gymnastics” at Pd^(II)^. Its behavior could explain observations where this ligand, and indeed related phosphine ligands capable of forming a palladacycle, standout in terms of reaction outcomes and product selectivities.^4^ We are unable to fully rule out a participating role for very small nonphosphine-stabilized Pd_n_ clusters formed under the catalytic reactions used in this study, where simplified models for catalytic cycles are often generalized. We have provided evidence that P(*o*-tolyl)_3_ presents more complex behavior, adding to what we already know about the nontrivial behavior of PPh_3_.^30,31^

## Supporting Information Available

*In situ* IR data, XRD files, NMR time courses, DFT coordinates, details of synthesis, and characterization of compounds (PDF)
(XYZ)
(CIF)

## Supplementary Material

cs4c02585_si_001.pdf

cs4c02585_si_002.xyz

cs4c02585_si_003.cif

## ABBREVIATIONS

SMCC: Suzuki–Miyaura cross-coupling
NMR: nuclear magnetic resonance
IR: infrared spectroscopy
XRD: X-ray diffraction
TEM: transmission electron microscopy
PdNP: palladium nanoparticles
PVP: polyvinylpyrrolidone
Ar–Br: aryl bromide
TGA: thermal gravimetric analysis
ESI: electrospray ionization mass spectrometry
LIFDI: liquid injection field desorption ionization mass spectrometry
