# Supplementary material for: Revealing the Hidden Complexity and Reactivity of Palladacyclic Precatalysts: The P(o-tolyl)3 Ligand Enables a Cocktail of Active Species Utilizing the Pd(II)/Pd(IV) and Pd(0)/Pd(II) Pathways for Efficient Catalysis
Source: ACS Catal. 2024 Aug 9;14(17):12769–82. doi: 10.1021/acscatal.4c02585 (PMC11385352; doi:10.1021/acscatal.4c02585)
Supplement: Supplementary file 1 — cs4c02585_si_001.pdf [file cs4c02585_si_001.pdf]

Supporting Information for

Revealing the hidden complexity and reactivity of palladacyclic pre-catalysts: The P(o-tolyl)$_3$ ligand enables a cocktail of active species utilizing Pd(II)/Pd(IV) and Pd(0)/Pd(II) pathways for efficient catalysis

David R. Husbands,[a] Theo Tanner,[a] Adrian C. Whitwood,[a] Neil S. Hodnett,[b] Katherine M. P. Wheelhouse[b] and Ian J. S. Fairlamb[a,*]

* Corresponding author: ian.fairlamb@york.ac.uk

[a] Department of Chemistry, University of York, Heslington, York, YO10 5DD, United Kingdom.
[b] Medicine Development & Supply, GSK Medicines Research Centre, Gunnels Wood Road, Stevenage, Hertfordshire, SG1 2NY, United Kingdom.

Table of Contents

1. General Information 3
   1.1 Compound Preparative Techniques 3
   1.2 Instrument Details and Methods for Compound Characterization 3
2. Experimental Details 5
   2.1 General Procedure: Heck Alkenylation Reaction Catalyst Screening 5
   2.2 General Procedure: SMCC Reaction for *in situ* IR Monitoring 6
   2.3 Synthesis of Compounds 8
   2.4 Experimental Details for 3-Phase Test 24
   2.5 Synthesis and Characterization of [Pd(P^C)(μ_2-Ar^F)]$_2$ palladacycle 16a 30
4. *In situ* IR Data 35
   4.1 Heck Reaction Profiles 36
   4.2 Effect of Catalyst Identity on SMCC 41
   4.3 Recharge Experiment 47
   4.4 Effect of Pinacol Additive on Kinetics 49
   4.5 Use of PdNPs as Catalysts 51
   4.6 Catalyst Concentration effect on kinetics 55
   4.7 Mercury Drop Test for PdNPs under SMCC Conditions 60
5. Transmission Electron Microscopy (TEM) for PdNP Observation 63

5.1 TEM from Suzuki Reaction Employing Arylboronic pinacol ester 4a....63
5.2 TEM from Suzuki Reaction Employing Arylboronic acid 4....64
6. [Pd(C^P)(μ_2-OH)]$_2$ Palladacycle 2 Activation Data....67
6.1 Test for Arylation and Identification of Inert Pd(C^P)$_2$ Palladacycle 11, and Identification of Generated Species....67
6.2 General Procedure for Arylation....71
6.3 Run 1 – Unchanged General Procedure 6.2....72
6.4 Run 2 – General Procedure 6.2 with D_2O additive....77
6.5 Run 3 – General Procedure 6.2 with Boric Acid Additive (4 equiv.)....83
6.6 Run 4 – General Procedure 6.2 with Excess Arylboronic Acid (10 equiv.)....89
7. Stoichiometric [Pd(P^C)(μ_2-OH)]$_2$ Palladacycle 2 Activation Experiments....94
7.1 Activation of [Pd(P^C)(μ_2-OH)]$_2$ Palladacycle 2 by Polar Solvents....94
7.2 Reaction Endpoint....95
7.3 Trapping Pd$^{(0)}$ with Excess Phosphine....96
7.4 Stoichiometric Cross-Coupling Reaction using [Pd(P^C)(μ_2-OH)]$_2$ Palladacycle 2 with Arylboronic Acid 9....101
7.5 Stoichiometric Cross-Coupling Reaction using [Pd(P^C)(μ_2-OH)]$_2$ Palladacycle 2 using Arylboronic Acid 4....103
7.6 Oxidative Addition to Authentic Pd$^{(0)}$ Complex to form [Pd(μ_2-Br)(C_6H_4F)[P(*o*-tolyl)$_3$]]$_2$ Oxidative Addition Dimer SI45....106
7.7 Exchange of [Pd(P^C)(μ_2-Br)]$_2$ Bridging Ligand with Base in Solution....109
7.8 Activation of [Pd(P^C)(μ_2-Ar^F)]$_2$ Palladacycle 16a with Excess Water....111
7.9 Cross-Coupling using [Pd(P^C)(μ_2- Ar^F)]$_2$ Palladacycle 16a....114
7.10 Incorporation of Deuterium under SMCC Reaction Conditions....116
8. X-Ray Crystallography....118
9. NMR Spectral Data for Organic and Inorganic Compounds....147
10. Density Functional Theory (DFT) Calculations....205
10.1 DFT Methodology and SPE Calculations for Proposed Pd$^{(II)}$/Pd$^{(IV)}$ Cross-Coupling Mechanism....205
10.2 Stabilization of Complex 13a and 36 by different ligands....207
11. References....210

1. General Information

1.1 Compound Preparative Techniques

Reagents were purchased from Merck, Fluorochem, Alfa Aesar, and Fisher and used without further purification. The purity of chemicals was confirmed by NMR spectroscopy prior to use.

1.2 Instrument Details and Methods for Compound Characterization

NMR spectra were obtained in the solvent indicated in the text below, using a Bruker AVIIIHD 600 Widebore instrument (600 MHz [^{1}H], 565 MHz [^{19}F], 243 MHz [^{31}P], 151 MHz [^{13}C]) or a Bruker AVIIIHD 500 instrument (500 MHz [^{1}H], 471 MHz [^{19}F], 203 MHz [^{31}P], 125 MHz [^{13}C]) or JEOL ECX400 or JEOL ECS400 spectrometer (400 MHz [^{1}H], 101 MHz [^{13}C] and 377 MHz [^{19}F]). Chemical shifts (δ) are reported in parts per million (ppm) and were referenced to the residual non-deuterated solvent of the deuterated solvent used; $CDCl_3$: δ ^{1}H = 7.26 (C*H*Cl_3) and ^{13}C = 77.16 (*C*DCl_3); DCM-*d*$_2$: ^{1}H = 5.32 (CD*H*Cl_2) and ^{13}C = 54.0 (*C*D_2Cl_2); THF-*d*$_8$: δ ^{1}H = 3.59 (OC*H*$_2$$CH_2$), ^{13}C = 67.57 (O*C*$H_2CH_2$), ^{1}H = 1.73 ($OCH_2C$*H*$_2$) ^{13}C = 25.37 ($OCH_2$*C*$H_2$); Toluene-*d*$_8$: δ ^{1}H = 2.08 ($C_6D_5CD_2$*H*), ^{13}C = 20.43 (C_6D_5*C*D_3); C_6D_6 : δ ^{1}H = 7.16 (C_6D_5*H*), ^{13}C = 128.06 (*C*$_6D_6$). Spectral data were typically collected at 298 K (25 °C), unless stated otherwise. All ^{1}H NMR signals are reported as they appear in the spectrum, which in some cases may lead to there being more protons reported than there are in the compound (according to HRMS). This is often due to residual solvent peaks (either from the NMR solvent or solvent trapped in the compounds during crystallization) overlapping in the aromatic region.

^{31}P NMR spectral data were collected with proton decoupling, unless otherwise stated. Chemical shifts for ^{31}P resonances were calibrated by externally referencing to 85% H_3PO_4 in H_2O (w/w). This was practically carried out by inserting a sealed, vacuum-dried capillary tube containing 85% H_3PO_4 in H_2O (w/w) into an NMR tube containing the sample of interest, collecting a ^{31}P NMR spectrum and setting the H_3*P*O_4 resonance to 0 ppm. ^{19}F spectral data were referenced in the same manner using α,α,α-trifluorotoluene (-63.72 ppm with respect to $CFCl_3$). All ^{13}C NMR spectra were obtained with ^{1}H decoupling. All NMR spectra were processed using MestReNova (MNova) software (v. 14).

HRMS ESI-MS spectra were measured using a Bruker Daltronics micrOTOF MS, Agilent series 1200LC with electrospray ionization (ESI) or on a Thermo LCQ using electrospray ionization, with <5 ppm error recorded for all HRMS samples. LIFDI (Liquid Injection Field Desorption Ionization) mass spectrometry was carried out using a JEOL AccuTOF GCx-plus instrument (JMS-T200GC), fitted with a probe produced by Linden CMS. The probe was equipped with 13 μm emitters on an AccuTOF. Alternatively, LIFDI-MS was carried out using a Waters GCT Premier MS Agilent 7890A GC instrument. Mass to charge ratios (*m/z*) are reported in Daltons. High resolution mass spectra (HRMS) are reported with <5 ppm error (ESI and LIFDI). For clarity, LIFDI data are reported for ^{106}Pd, the most abundant natural isotope of Pd, which is part of 'exact mass' values. LIFDI ions are reported as the radical cation $M^{+\bullet}$. ESI ions are reported as the $[M+H]^+$ cation,

unless a Na or K is present in the molecular formula, in which case the $[M+Na]^+$ or $[M+K]^+$ ion is being measured.

Infrared spectra were obtained using a Bruker ALPHA-Platinum FTIR Spectrometer with a platinum-diamond ATR sampling module. Far IR spectra were obtained using a Bruker Tensor 37 FTIR Spectrometer with a platinum-diamond ATR sampling module. Melting points were determined using a Stuart® SMP3 Melting Point machine. All Heck reaction kinetic experiments were followed using a Mettler Toledo React-IR spectrometer with a silicon probe and K6 conduit/R4 (mirror arm), or a diamond probe. IR spectra were collected in real-time every 60 seconds between 4000 and 649 cm^{-1}, with a spectral resolution of 4 cm^{-1}. All reaction temperatures were measured independently from the heating bath using a TENMA 72-7715 electronic thermometer and thermocouple to ensure accurate reaction temperature regulation. All data analysis was carried out on raw, uncorrected data.

For single crystal X-ray crystallographic analysis details, please see Section 8.

2. Experimental Details

2.1 General Procedure: Heck Alkenylation Reaction Catalyst Screening

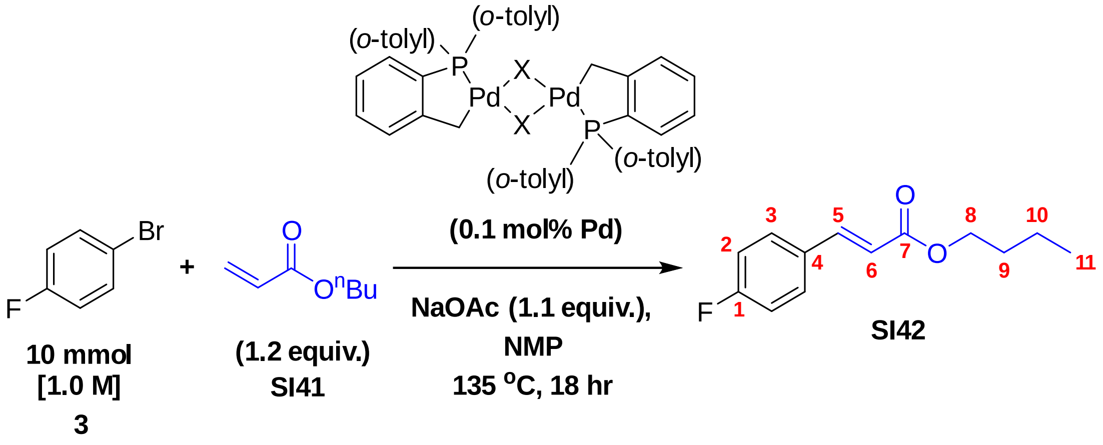

An oven-dried three-necked 100 mL round bottom flask was fitted onto the ReactIR probe, fitted with a condenser, and evacuated / backfilled with N_2 three times. The flask was charged with NMP (10.0 mL, dried, degassed), then heated to 135 °C, at which point a solvent background IR spectrum was recorded. NaOAc (903 mg, 11.0 mmol, 1.1 equiv., anhydrous) was added, followed by 1-bromo-4-fluorobenzene **3** (1.1 mL, 1750 mg, 10.0 mmol, 1.0 equiv., stored over molecular sieves), then catalyst (0.005 mmol, 0.1 mol% Pd) was added. The reaction was initiated by the addition of n-butyl acrylate **SI41** (1539 mg, 1.71 mL, 12.0 mmol, 1.2 equiv., stored over molecular sieves). The reaction progress was monitored by measuring the increase in absorbance at 1509 cm^{-1}, relating to the formation of n-butyl 4-fluorocinnamate **SI42**, and the decrease in absorbances at 1484 cm^{-1}, relating to the consumption of 1-bromo-4-fluorobenzene, and 1190 cm^{-1}, relating to n-butyl acrylate. To deconvolute the peaks and get reasonable quality data, the second derivative of the spectra was used. The reaction was halted when the peaks at these values reached a constant value, indicating completion. At the end of the reaction, a 0.5 mL aliquot of the reaction was analyzed by ^{19}F NMR (20 s relaxation delay) to measure the conversion of each reaction. The final conversion was calculated by measuring the ratio of the diagnostic peaks by (^{19}F δ) -115.9 ppm for 1-bromo-4-fluorobenzene and -109.8 ppm for n-butyl 4-fluorocinnamate. The peak absorption data were imported into Excel after subtracting the NMP reference spectrum. All kinetic traces (conversions) were normalized using the final reaction conversion (as calculated *vide supra*) and the value of an appropriate data point just before the aliquot was taken.

Identity of the product was confirmed by performing a reaction at half-scale in a Schlenk tube following the same procedure. The reaction mixture was extracted into EtOAc (3 x 20 mL), the organic layers were combined and washed with water (5 x 20 mL), dried ($MgSO_4$), filtered and the solvent removed *in vacuo*. The crude was purified by column chromatography (automated Combiflash, 12 g silica cartridge, 5% EtOAc in hexane), R_f = 0.34 (5% EtOAc in hexane)) to give the product **SI42** as a colorless oil (exclusively the

trans isomer, 812 mg, 73% yield). (Note: upon isolation, 2% of bis-arylated by-product co-eluted, and is visible in the ^{19}F NMR spectrum at -111.47 and -113.33 ppm.) ^{1}H NMR (500 MHz, $CDCl_3$, 298 K) δ 7.63 (d, *J* = 16.0 Hz, 1H, H-5), 7.54 – 7.47 (m, 2H, H-3), 7.10 – 7.03 (m, 2H, H-2), 6.36 (dd, *J* = 16.0, 0.6 Hz, 1H, H-6), 4.20 (t, *J* = 6.7 Hz, 2H, H-8), 1.73 – 1.63 (m, 2H, H-9), 1.48 – 1.38 (m, 2H, H-10), 0.96 (t, *J* = 7.4 Hz, 3H, H-11); ^{19}F NMR (470 MHz, $CDCl_3$, 298 K) δ -109.85 (tt, *J* = 8.4, 5.4 Hz); ^{13}C NMR (126 MHz, $CDCl_3$, 298 K) δ 167.1 (C-7), 164.0 (d, *J* = 251.3 Hz, C-1), 143.3 (C-5), 130.9 (d, *J* = 3.4 Hz, C-4), 130.0 (d, *J* = 8.5 Hz, C-3), 118.2 (d, *J* = 2.3 Hz, C-6), 116.1 (d, *J* = 22.0 Hz, C-2), 64.6 (C-8), 30.9 (C-9), 19.3 (C-10), 13.9 (C-11); HRMS (ESI$^+$) $(C_{13}H_{15}FO_2Na)^+$ *m/z* (calculated) 245.0948 (found) 245.0949, mass difference 0.3 ppm; FTIR (ATR): ṽ (cm^{-1}) 2960 (C-H aromatic), 2874 (C-H aliphatic), 1708 (s, C=O acrylate), 1639 (C=C acrylate), 1600 (C=C aromatic), 1509, 1466, 1415, 1384, 1312, 1279, 1257, 1230, 1196, 1158 (vs, C-O stretch) 1095, 1063, 1025, 980, 868, 829, 790, 738, 509, 414. Data matches literature for this compound.[1]

Lab book ref. DRH-03-159 (for characterization data)

2.2 General Procedure: SMCC Reaction for *in situ* IR Monitoring

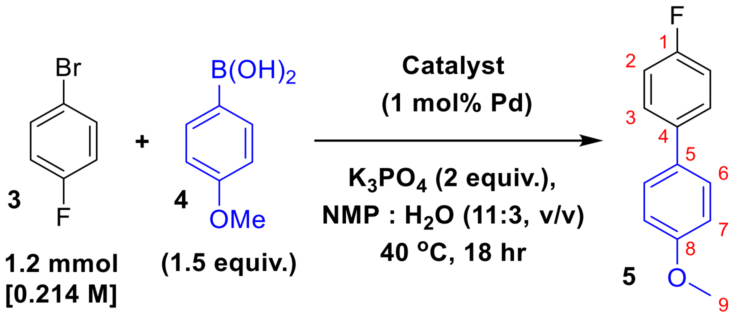

An oven-dried three-necked 100 mL round bottom flask was fitted onto the ReactIR probe, and purged with argon for 15 mins. At all points of the experiment hereafter, a flow of argon to the reaction vessel was maintained. NMP (dry, degassed, 4.4 mL) and H_2O (degassed, 1.2 mL) were added and the hotplate temperature set to 40 °C. When this temperature had been reached and stabilized, a reference spectrum was recorded. To the flask, anhydrous tribasic K_3PO_4 (510 mg, 2.4 mmol, 2 equiv.), 4-methoxyphenylboronic acid **4** (275 mg, 1.8 mmol, 1.5 equiv.) and 1-bromo-4-fluorobenzene **3** (132 µL, 1.2 mmol, 1.0 equiv.) were added sequentially at 5 minute intervals. The reaction was initiated by the addition of the palladium catalyst (1 mol% w.r.t. 1-bromo-4-fluorobenzene **3**). The reaction progress was monitored by measuring the increase in absorbance at 795 cm^{-1}, relating to the formation of 4-(4-fluorophenyl)anisole **5**, and the decrease in absorbance at 1486 cm^{-1}, relating to the consumption of 1-bromo-4-fluorobenzene **3**. To deconvolute the peaks and obtain acceptable signal to noise, the second derivative of the spectra was used. The reaction was halted when the peaks at these wavenumbers reached a constant value, indicating reaction completion. At the end of the reaction, a 0.5 mL aliquot of the reaction was analyzed by ^{19}F NMR (20 s relaxation delay) to measure the conversion of each reaction. The final reaction conversion

was calculated by measuring the ratio of the diagnostic peaks by (^{19}F δ) -115.9 ppm for 1-bromo-4-fluorobenzene **3** and -117.6 ppm for 4-(4-fluorophenyl)anisole **5**. The peak absorption data for 1486 cm^{-1} and 795 cm^{-1} were imported into Excel, after subtracting the NMP reference spectrum. All kinetic traces (conversions) were normalized using the final reaction conversion and the value of an appropriate data point just before the aliquot was taken.

The identity of the product was confirmed by working up the reaction after completion. The reaction mixture was extracted into EtOAc (3 x 20 mL), the organic layers were combined and washed with water (5 x 20 mL), dried ($MgSO_4$), filtered and the solvent removed in vacuo. The crude was purified by column chromatography (manual column chromatography, SiO_2, dry loaded, 5% EtOAc in hexane, R_f = 0.38 (5% EtOAc in hexane)) to give the product as a white solid; ^1H NMR (600 MHz, $CDCl_3$, 298 K) δ 7.53 – 7.45 (m, 4H, H-6, H-3), 7.14 – 7.07 (m, 2H, H-2), 7.00 – 6.95 (m, 2H, H-7), 3.86 (s, 3H, H-9); ^{19}F NMR (565 MHz, $CDCl_3$, 298 K) δ -116. (tt, *J* = 8.6, 5.3 Hz); ^{13}C NMR (151 MHz, $CDCl_3$, 298 K) δ 162.3 (d, *J* = 245.4 Hz, C-1), 159.3 (C-8), 137.1 (d, *J* = 3.2 Hz, C-4), 133.0 (C-5), 128.4 (d, *J* = 7.9 Hz, C-3), 128.2 (C-6), 115.7 (d, *J* = 21.4 Hz, C-2), 114.4 (C-7), 55.5 (C-9); HRMS (EI) ($C_{13}H_{11}FO$)$^{\cdot}$ m/z (calculated) 202.07884, (found) 202.07812, mass difference 3.57 ppm; FTIR (ATR): ṽ (cm^{-1}) 3067 (C-H aromatic), 3016 (C-H aromatic), 2965 (C-H aromatic), 2841 (C-H aliphatic), 1597 (C=C aromatic), 1495 (C=C aromatic), 1466, 1441, 1401, 1326, 1291, 1230 (C-O), 1181, 1134, 1118, 1038 (C-F), 1012, 952, 824, 808, 791, 538, 506, 473, 443. Data matches literature for this compound.[2]

Lab book ref. DRH-03-96-2 (for NMR data), DRH-02-1-3

Note: when pinacol was used as an additive, (213 mg, 1.8 mmol, 1.5 equiv.) was added after the arylboronic acid. When arylboronic pinacol ester **4a** was used, there was an addition of (483 µL, 1.8 mmol, 1.5 equiv.) instead of boronic acid.

2.3 Synthesis of Compounds

Synthesis of $[Pd(P^\wedge C)(\mu_2\text{-}OH)]_2$ Palladacycle 2

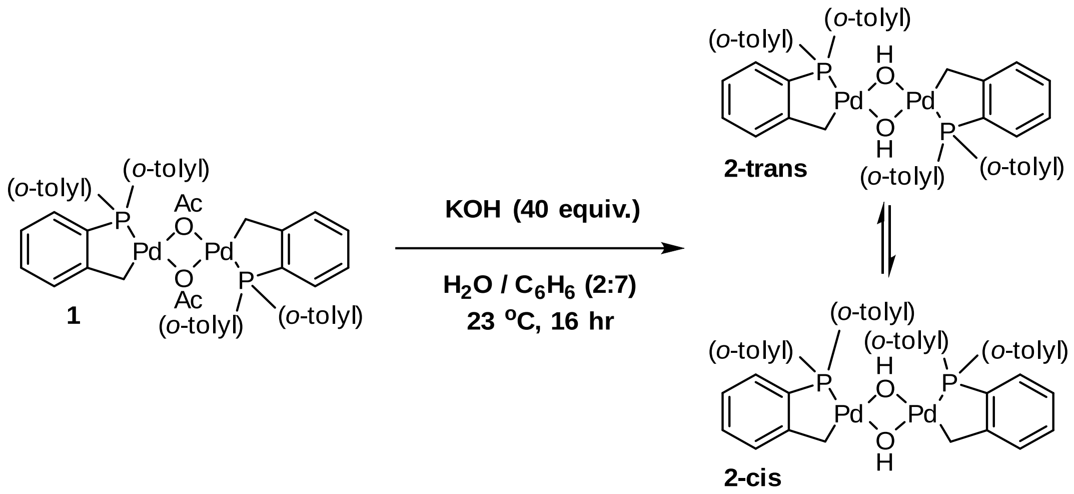

All solvents were degassed with N_2, and all reactions and manipulations were performed under a nitrogen atmosphere. The Herrmann-Beller palladacycle **1** (750 mg, 0.80 mmol, 1 eq.) was added to benzene (15 mL) and stirred to give a yellow suspension. To this, KOH (2290 mg, 40.8 mmol, 51 eq.) dissolved in deionized water (4 mL) was added, and the biphasic mixture stirred vigorously for 18 h. The cream suspension in the benzene layer was decanted, and the aqueous layer extracted with warm benzene (60 °C, 4 × 6 mL). The organic layers were collected, washed with water (2 × 10 mL) and the solvent removed *in vacuo*, giving a cream colored product **2** as *cis*/*trans* isomers in a 1:2 ratio in solution (683 mg, 66 %); ^{1}H NMR (500 MHz, DCM-d_2, 203 K) δ; 7.46 – 7.33 (m, 4H, Ar-*H*), 7.33 – 7.22 (m, 11H, Ar-*H*), 7.10 (dd, *J* = 13.2, 7.8 Hz, 4H, Ar-*H*), 7.08 – 6.98 (m, 2H, Ar-*H*), 6.95 (d, *J* = 11.0 Hz, 1H, Ar-*H*), 6.81 – 6.70 (m, 3H, Ar-*H*), 6.71 – 6.61 (m, 1H, Ar-*H*), 3.07 (d, *J* = 14.2 Hz, 1H, CH_2), 2.99 (s, 5H), 2.99 – 2.90 (m, 3H, CH_2), 2.85 (d, *J* = 6.8 Hz, 2H, CH_2), 2.82 (d, *J* = 3.3 Hz, 1H, CH_2), 2.79 (s, 2H, CH_3), 2.75 (dd, *J* = 14.0, 3.3 Hz, 1H, CH_2), 2.64 (d, *J* = 2.9 Hz, 3H, CH_3), 2.53 (s, 4H, CH_3), 2.36 (s, 1H, CH_3), 2.22 (s, 2H, CH_3), -0.65 (s, μ-OH *cis*), -0.93 (s, μ-OH *cis*), -1.26 (d, *J* = 2.7 Hz, μ-OH *trans*), -1.40 (d, *J* = 2.8 Hz, μ-OH *trans*), -2.31 (s, μ-OH *cis*), -2.44 (s, μ-OH *cis*); ^{13}C NMR (135 MHz, DCM-d_2, 298 K) δ; 158.9 (dd, *J* = 31.5, 8.7 Hz, Ar-*C*), 142.8 (Ar-*C*), 135.8 (d, *J* = 56.3 Hz, Ar-*C*), 132.7 (Ar-*C*), 132.6 (Ar-*C*), 132.1 (Ar-*C*), 132.0 (Ar-*C*), 131.2 (d, *J* = 2.2 Hz, Ar-*C*), 131.2 (d, *J* = 2.5 Hz, Ar-*C*), 131.0 (Ar-*C*), 130.7 (Ar-*C*), 128.3 (dd, *J* = 21.5, 8.1 Hz, Ar-*C*), 126.2 (Ar-*C*), 125.8 (Ar-*C*), 125.7 (Ar-*C*), 125.7 (Ar-*C*), 26.6 (*C*H_2), 24.9 (*C*H_2), 23.0 (*C*H_3), 22.9 (*C*H_3), 22.8 (*C*H_3); ^{31}P NMR (202.5 MHz, DCM-d_2, 203 K) δ; 34.02 (*trans* isomer), 33.56 (*cis* isomer), 33.37 (*cis* isomer), 33.18 (*trans* isomer); HRMS (ESI$^+$) $(C_{42}H_{43}O_2P_2Pd_2)^+$ *m/z* (calculated) 853.0802 (found) 853.0785, mass difference 1.7 ppm, $(C_{21}H_{22}OPPd)^+$ *m/z* (calculated) 427.0438, (found) 427.0453, mass difference 1.6 ppm. FTIR (ATIR): $\tilde{\nu}$ (cm^{-1}) 3646 (O-H), 3051 (C-H aromatic), 2857 (C-H aliphatic), 1582 (C=C aromatic), 1466, 1440 (C-P), 1283, 1069, 750; Far IR (ATIR) $\tilde{\nu}$ (cm^{-1}) 586, 560, 525, 509, 477, 459, 441, 417, 362, 316, 269,

249; mp. decomposed above 188 °C. This is a novel compound. Lab book ref. DRH-01-108 (X-ray diffraction structure ijsf2016, ijsf 21008a, ijsf 21005),

Synthesis of the $[Pd(P^{\wedge}C)(\mu_2\text{-}Br)]_2$ Palladacycle 35

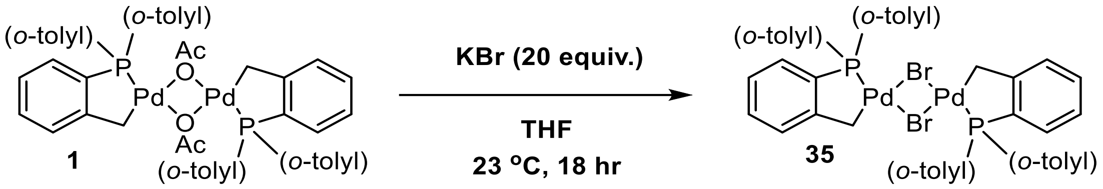

The Herrmann-Beller palladacycle **1** (30 mg, 0.032 mmol, 1 eq.) and anhydrous KBr (76 mg, 0.64 mmol, 20 eq.) were added to THF (2 mL, dry, degassed) under N_2. The resulting mixture was rapidly stirred for 18 hours and stirred to give a vivid yellow suspension. Deionized water (10 mL) was added, and the complex was extracted into DCM (3 × 10 mL). The organic layers were collected, washed (water, 10 mL), dried ($MgSO_4$), filtered and the solvent evaporated *in vacuo* to give the $Pd(P^{\wedge}C)(\mu_2\text{-}Br)]_2$ palladacycle 35 as a bright yellow powder (32 mg, 100%); ^1H NMR (500 MHz, DCM-d_2, 203 K) δ 7.47 – 7.17 (m, 12H, Ar-*H*), 7.17 – 7.00 (m, 8H, Ar-*H*), 6.86 (dd, *J* = 12.0, 7.7 Hz, 1H, Ar-*H*), 6.82 – 6.73 (m, 2H, Ar-*H*), 6.73 – 6.68 (m, 1H, Ar-*H*), 3.77 – 3.64 (m, 2H, CH*H*), 3.43 (t, *J* = 15.6 Hz, 2H, C*H*H), 2.70 (s, 1H, tolyl-*H*), 2.66 – 2.60 (m, 4H, tolyl-*H*), 2.59 (s, 1H, tolyl-*H*), 2.55 (s, 1H, tolyl-*H*), 2.53 (s, 1H, tolyl-*H*), 2.51 (s, 1H, tolyl-*H*), 1.70 (s, 3H, tolyl-*H*); ^{31}P NMR (202 MHz, DCM-d_2, 203 K) δ 41.49 (s), 41.29 (s) (Note: due to extremely low solubility in tested solvents, it was not possible to get a ^{13}C NMR of this complex. This complex exists as two isomers (phosphorus -*cis* and -*trans*) at 203 K); HRMS (LIFDI) $(C_{42}H_{40}P_2Br_2Pd_2)^{+\bullet}$ *m/z* (calculated) 975.90362, (found) 975.90083, mass difference 2.85 ppm; (ATIR): $\tilde{\nu}$ (cm^{-1}) 2970 (C-H aromatic), 2858 (C-H aliphatic), 1583 (C=C aromatic), 1465, 1440 (C-P), 1203, 1028, 753, 617, 580, 558, 520, 469, 459; mp. decomposed above 285 °C.

Lab book ref. DRH-02-8-1, DRH-02-39 (X-ray diffraction structure ijsf21041)

Synthesis of the $[Pd(P^{\wedge}C)(\mu_2\text{-}Cl)]_2$ Palladacycle 17

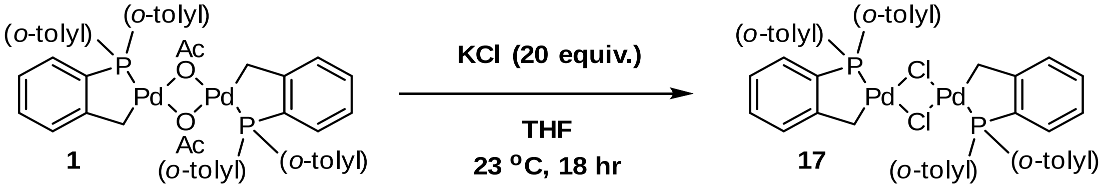

The Herrmann-Beller palladacycle **1** (150 mg, 0.16 mmol, 1 eq.) and anhydrous KCl (239 mg, 3.2 mmol, 20 eq.) were added to THF (10 mL, dry, degassed) under N_2. The resulting mixture was rapidly stirred for 18 hours and stirred to give a yellow suspension. Deionised water (10 mL) then DCM (40 mL) were added (Note: This complex is sparingly soluble in DCM, so larger volumes may be needed). The organic layers

were collected, washed (water, 2 × 20 mL), and the solvent evaporated (without drying) in vacuo to give the [Pd(P^C)(μ_2-Cl)]$_2$ palladacycle **17** as a bright yellow powder (134 mg, 94%); ^{1}H NMR (500 MHz, DCM-d_2, 203 K) δ 7.47 – 7.18 (m, 12H, Ar-*H*), 7.18 – 6.94 (m, 8H, Ar-*H*), 6.86 – 6.65 (m, 4H, Ar-*H*), 3.59 – 3.42 (m, 2H, C*H*H), 3.37 (q, *J* = 13.8, 11.3 Hz, 2H, CH*H*), 2.79 (s, 2H, tolyl-*H*), 2.73 (s, 1H, tolyl-*H*), 2.66 (s, 1H, tolyl-*H*), 2.65 (s, 3H, tolyl-*H*), 2.54 (s, 2H, tolyl-*H*), 2.44 (s, 1H, *tolyl-H*), 2.40 (s, 1H, tolyl-*H*), 1.70 (s, 1H, tolyl-*H*); ^{31}P NMR (202 MHz, DCM-d_2, 203 K) δ 39.06 (s), 38.86 (s), 38.76 (s), 38.64 (s). (Note: due to extremely low solubility in tested solvents, it was not possible to get a ^{13}C NMR of this complex. At 203 K, the broad ^{31}P environment observed at room temperature splits into 4 peaks, each representing a structural isomer of this complex); HRMS (LIFDI) ($C_{42}H_{40}P_2Pd_2Cl_2$)$^{+\bullet}$ m/z (calculated) 888.00465, (found) 888.00508, mass difference 0.49 ppm; (ATIR): ṽ (cm-1) 3053 (C-H aromatic), 2969 (C-H aromatic), 2940 (C-H aliphatic), 2869 (C-H aliphatic), 1586 (C=C aromatic), 1578 (C=C aromatic), 1464, 1440 (C-P), 1283, 1264, 1203, 1162, 1130, 1083, 1052, 805, 757, 714, 579, 559, 518, 479, 469, 461; mp. decomposed above 280 °C.

Lab book ref. DRH-02-130, DRH-03-78

Synthesis of the [Pd(P^C)(μ_2-I)]$_2$ Palladacycle 32

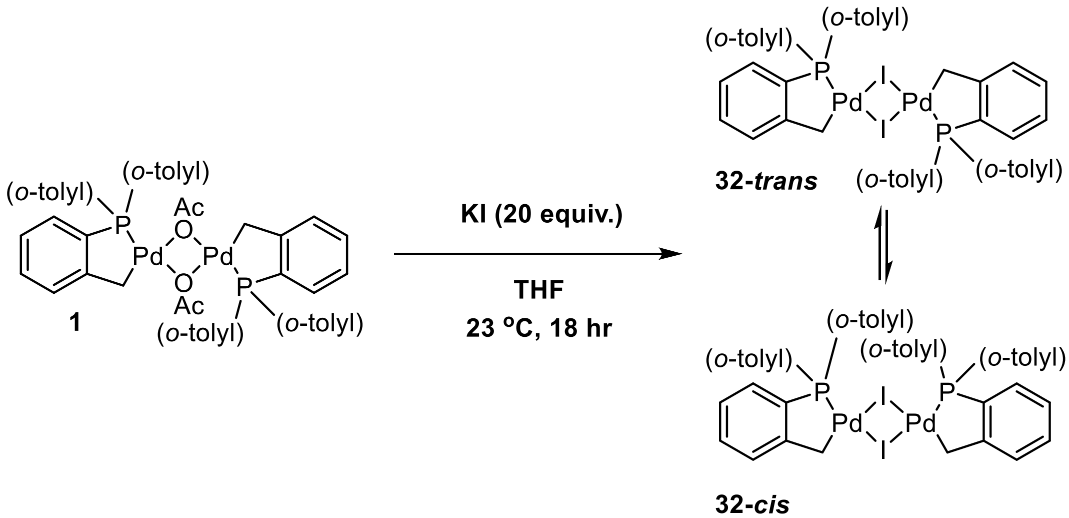

The Herrmann-Beller palladacycle **1** (30 mg, 0.032 mmol, 1 eq.) and anhydrous KI (106 mg, 0.64 mmol, 20 eq.) were added to THF (2 mL, dry, degassed) under N_2. The resulting mixture was rapidly stirred for 18 hours and stirred to give a red suspension. Deionized water (10 mL) was added, and the complex was extracted into DCM (3 x 10 mL). The organic layers were collected, washed (water, 10 mL), dried ($MgSO_4$), filtered and the solvent evaporated in vacuo to give the [Pd(P^C)(μ_2-I)]$_2$ palladacycle **32** as a red-orange powder (27 mg, 79%); ^{1}H NMR (500 MHz, DCM-d_2, 298 K) δ 7.40 (d, *J* = 7.7 Hz, 5H, Ar-*H*), 7.33 – 7.22 (m, 10H, Ar-*H*), 7.17 (s, 5H, Ar-*H*), 7.06 (t, *J* = 7.3 Hz, 2H, Ar-*H*), 6.92 (s, 2H, Ar-*H*), 3.79 (s, 4H, CH_3, CH_2),

2.63 (d, J = 15.8 Hz, 12H, CH_3, CH_2); ^{31}P NMR (203 MHz, DCM-d_2, 298 K) δ; 45.27, 44.45; (Note: due to extremely low solubility in tested solvents, it was not possible to get a ^{13}C NMR of this complex) HRMS (LIFDI$^+$) $(C_{42}H_{40}P_2Pd_2I_2)^{\cdot+}$ m/z (calculated) 1071.87588, (found) 1071.87958, mass difference 3.45 ppm; (ATIR): ṽ (cm-1) 2950 (C-H aromatic), 2878 (C-H aliphatic), 1462 (C=C aromatic), 1440 (C-P), 1263, 1162, 1069, 804, 754, 712, 680, 575, 558, 518, 468; mp. decomposed above 276 °C.

Lab book ref. DRH-02-3

Synthesis of (phenyl-2-boronic acid)diphenyl phosphine 19

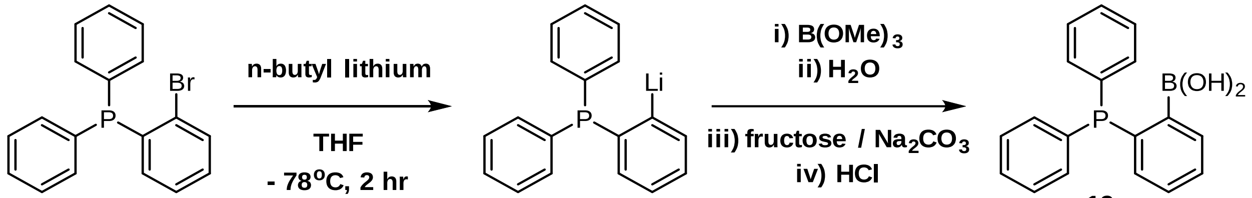

19

(2-Bromophenyl)diphenyl phosphine (500 mg, 1.47 mmol, 1 eq.) was dried *in vacuo* in an oven-dried Schlenk flask for 1 hour. THF (7.5 mL, dry, degassed) was added under N_2, and the solution cooled to -78 °C. n-Butyl lithium (2.5 M in hexane, 0.6 mL, 1.5 mmol, 1.02 eq.) was added dropwise, and the resulting orange solution was stirred at -78 °C for 2 hours. Trimethylborate (0.82 mL, 7.35 mmol, 5 eq.) was added dropwise, and the resulting solution was stirred for 1 hour before being slowly brought to room temperature, where stirring continued for 18 hours. Water (3 mL, deionised) was added dropwise under N_2 to the yellow solution, giving a cloudy white suspension. Diethyl ether (10 mL) was added, the mixture was separated and extracted into diethyl ether (2 × 10 mL). The organic phases were combined, dried ($MgSO_4$), filtered and concentrated *in vacuo* to give the crude product as a gummy cream solid, which foamed under high vacuum. Purification of the crude product was achieved by dissolving the gummy solid in ethyl acetate (15 mL). An equal volume of 1 M Na_2CO_3 and D-fructose solution was added, and the mixture vigorously shaken for 5 mins. The aqueous layer was collected and acidified to pH 2 (6 M HCl, then 1 M HCl), during which a pale yellow solid formed. The aqueous layer was extracted with ethyl acetate (3 × 15 mL), the organic layers were washed with water (2 × 15 mL, deionised), dried ($MgSO_4$) and concentrated *in vacuo*. The resulting oil was dissolved in EtOAc and passed through a short silica plug. The solution was concentrated in vacuo, giving a white foam **19** (67 mg). ^{1}H NMR (600 MHz, CDCl$_3$, 298 K) δ 7.40 – 7.32 (m, 7H), 7.33 – 7.18 (m, 4H), 7.19 (s, 2H), 6.09 (d, J = 7.3 Hz, 2H, B(OH)$_2$); ^{31}P NMR (243 MHz, CDCl$_3$, 298 K) -13.0 ppm, consistent with literature.[3] HRMS (ESI$^+$) $(C_{18}H_{17}BO_2P)^{\cdot+}$ m/z (calculated) 307.1054, (found) 307.1051, mass difference 1.4 ppm.

NMR analysis showed a significant number of impurities (58% pure by ^{31}P NMR integration) – the significant impurities δ (^{31}P) were PPh_3 (-3.96 ppm), and the phosphine oxide (40.79 ppm), with unknown species at 53-54 ppm. Due to the synthetic challenges of making this compound and the assumption that the

phosphine oxide would not bind as strongly as **19**, it was decided to use this impure compound in an excess for future reactions.

Lab book ref. DRH-02-35

Reaction of (phenyl-2-boronic acid)diphenyl phosphine 19 with [Pd(P^C)(μ_2-OH)]$_2$ palladacycle 2

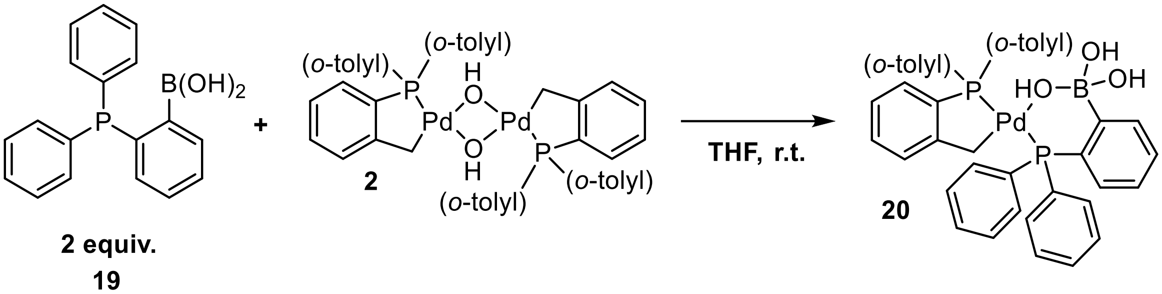

(phenyl-2-boronic acid)diphenyl phosphine **19** (6 mg, 0.0177 mmol, 3 eq.) and [Pd(P^C)(μ_2-OH)]$_2$ palladacycle **2** (5 mg, 0.0059 mmol, 1 eq.) were charged into a J-Young NMR tube and dissolved in THF (0.5 mL, dry, degassed) under an N_2 atmosphere before being analyzed by NMR spectroscopy . (Note: as the starting phosphine was impure (58%), an excess was used).

The NMR spectrum shows a distinctive pair of AB roofed doublets, consistent with the proposed product (^{31}P NMR (243 MHz, THF unlocked, 298 K) δ; 29.10 (d, J = 415 Hz), 26.62 (d, J = 415 Hz)). This was confirmed by ^{31}P COSY experiments, and (HRMS (ESI$^+$) ($C_{39}H_{36}BO_2P_2$ Pd)$^+$ m/z (calculated) 715.1313, (found) 715.1344, mass difference 1.2 ppm) as the cation with loss of OH$^-$.

Single crystals suitable for X-ray diffraction were obtained by slow vapor diffusion (benzene/pentane), and showed a boroxine-containing complex **21**, indicating that the impurities in the starting material contribute to the overall stability.

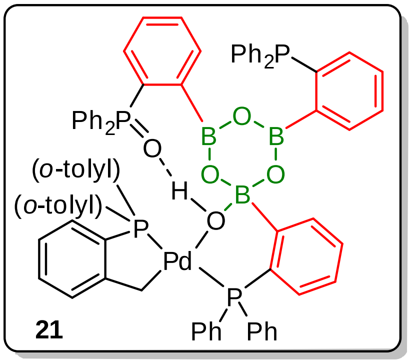

Lab book ref. DRH-02-44 (X-ray diffraction structure ijsf21046a)

Synthesis of (phenyl-2-pinacol ester)diphenyl phosphine 22

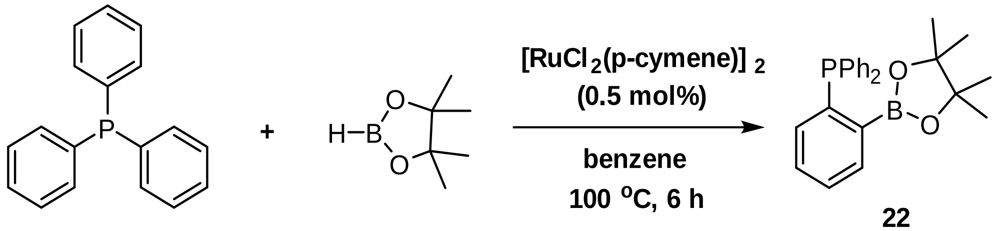

22

(phenyl-2-pinacol ester)diphenyl phosphine **22** was synthesized according to a literature procedure.[4] Triphenylphosphine (131 mg, 0.5 mmol, 1 eq.) and dichloro(p-cymene)ruthenium dimer (1.5 mg, 2.5 μmol, 0.5 mol%) were dissolved in benzene (dry, degassed, 2.5 mL) under a N_2 atmosphere in a large glass ampoule. To this solution, HBpin (80 μL, 0.55 mmol, 1.1 eq.) was added (Note: fresh HBpin is required, as the compound decomposes over time, leading to low reaction yields), then the vessel was sealed under N_2 with a PTFE screw-tap and the reaction refluxed at 100 °C for 6 h. (Safety Note: As H_2 gas is evolved by this reaction, a high-pressure glass ampoule was used with a blast shield). The solvent was removed *in vacuo*, giving the crude product. The product was purified by flash column chromatography (automated Combiflash, 4 g silica cartridge, neat hexane, rising to 5% EtOAc in hexane over 1 column volume, held at 5%), giving the product **22** as a white powder (97 mg, 50%). ^{1}H NMR (500 MHz, C*D*Cl_3, 298K) δ 7.80 – 7.75 (m, 1H), 7.31 – 7.18 (m, 12H), 6.75 (ddd, *J* = 6.5, 4.6, 1.5 Hz, 1H), 1.05 (s, 12H); ^{31}P NMR (202 MHz, C*D*Cl_3, 298K) δ -4.16; ^{13}C NMR (126 MHz, C*D*Cl_3, 298K) δ 143.7 (d, *J* = 17.5 Hz), 138.4 (d, *J* = 11.0 Hz) 135.6 (d, *J* = 8.7 Hz), 134.3 (d, *J* = 20.0 Hz), 132.3, 130.6, 128.6, 128.5 (d, *J* = 7.2 Hz), 127.4, 84.0, 24.6 (d, *J* = 2.8 Hz) (the ^{13}C environment neighbouring the B atom was not observed due to fast relaxation from the B atom); ^{11}B NMR (160 MHz, C*D*Cl_3, 298K) δ 31.82; (ATIR): $\tilde{\nu}$ (cm^{-1}) 3051 (C-H aromatic), 2977 (C-H aliphatic), 1581 (C=C aromatic), 1475, 1432 (C-P), 1343 (s, C-C aliphatic), 1316 (s, B-O), 1141, 1103, 1048, 962, 855, 743, 695, 660, 497; HRMS (ESI^+) $(C_{24}H_{27}BO_2P)^+$ *m/z* (calculated) 389.1836, (found) 389.1841, mass difference 0.7 ppm; mp 113.0 – 113.7 °C. Data matches literature for this compound.[4]

Lab book ref. DRH-02-57

Synthesis of Pd-OH pinacol ester phosphine complex 23

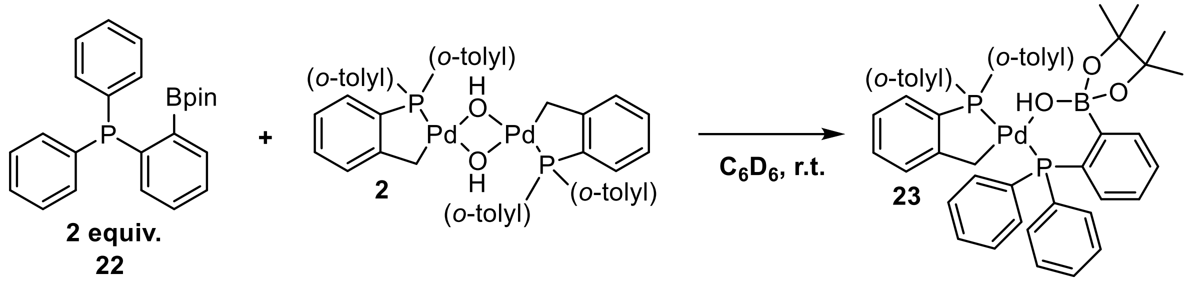

(Phenyl-2-pinacol ester)diphenyl phosphine **22** (50 mg, 0.129 mmol, 2.1 eq.) and the $Pd_2(\mu_2\text{-}OH)_2$ palladacycle **2** (55 mg, 0.064 mmol, 1 eq.) were dissolved in DCM (2 mL, dry, degassed) under nitrogen and stirred rapidly for 30 mins. The yellow solution was filtered to remove an insoluble white precipitate, then concentrated in vacuo to ~ 0.5 mL. Hexane (10 mL, dry, degassed) was added and the mixture was gently shaken and immediately stored at -20 °C for 24 hours. A mixture of colorless crystals and white precipitate was formed and the solvent filtered off. The solid was dried *in vacuo*, giving the product **23** (41 mg, 39%). ^{1}H NMR and single crystal XRD analysis revealed that the product contained stabilising HO-Bpin hydrogen bonding to the complex (integration of free and bound pinacol protons). ^{1}H NMR (500 MHz, DCM-d_2, 193 K) δ 7.76 – 7.65 (m, 3H, Ar-*H*), 7.47 – 7.27 (m, 11H, Ar-*H*), 7.21 (dt, *J* = 9.8, 5.1 Hz, 3H, Ar-*H*), 7.14 (t, *J* = 7.6 Hz, 2H, Ar-*H*), 7.10 – 6.98 (m, 4H, Ar-*H*), 6.99 – 6.91 (m, 1H, Ar-*H*), 6.76 (t, *J* = 8.4 Hz, 1H, Ar-*H*), 2.90 (s, 3H, tolyl-CH_3), 2.58 (s, 2H, CH_2), 2.52 (s, 3H, tolyl-CH_3), 0.61 (d, *J* = 20.2 Hz, 12H, pinacol-CH_3); ^{31}P NMR (243 MHz, DCM-d_2, 298 K) δ 26.75 (d, *J* = 374 Hz), 20.12 (d, *J* = 374 Hz); ^{13}C NMR (151 MHz, DCM-d_2, 298 K) δ 159.0 (d, *J* = 2.2 Hz, Ar-*C*), 158.7 (Ar-*C*), 143.0 (Ar-*C*), 135.0 (Ar-*C*), 133.8 (d, *J* = 6.3 Hz, Ar-*C*), 133.0 (Ar-*C*), 132.9 (Ar-*C*), 132.9 (Ar-*C*), 132.7, 132.6 – 132.5 (m, Ar-*C*), 132.4, 132.2, 131.6 (d, *J* = 2.5 Hz, Ar-*C*), 131.2 (Ar-*C*), 130.1 (d, *J* = 2.4 Hz, Ar-*C*), 129.4 (d, *J* = 2.7 Hz, Ar-*C*), 129.2 (d, *J* = 10.2 Hz, Ar-*C*), 128.4 (d, *J* = 10.2 Hz, Ar-*C*), 128.2 (d, *J* = 20.0 Hz, Ar-*C*), 126.6 (d, *J* = 7.6 Hz, Ar-*C*), 126.1 (d, *J* = 7.0 Hz, Ar-*C*), 125.4 (d, *J* = 7.2 Hz, Ar-*C*), 82.9, 79.0, 25.2 (d, *J* = 1.9 Hz, *C*H_2), 24.7 (*C*H_3), 23.8 (*C*H_3); ^{11}B NMR (193 MHz, DCM-d_2, 298 K) δ 22.66 (HO-Bpin), 5.93 (complex); (ATIR): $\tilde{\nu}$ (cm^{-1}) 3610 (br, O-H), 3392 (br, O-H), 3053 (C-H aromatic), 2965 (C-H aliphatic), 2925 (C-H aliphatic), 1582 (C=C aromatic), 1524, 1475, 1435 (C-P), 1368, 1268, 1201, 1154 (s, C-O), 1097, 1008, 951, 850, 743, 693, 580, 559, 507, 463; HRMS (ESI)$^+$ $(C_{39}H_{35}P_2Pd)^+$ *m/z* (found) 671.1237 (Note: It was not possible to get the mass of the full molecular ion by LIFDI, ESI or APCI – in each case there was loss of the HOBpin group, giving the fragment observed here).

Lab book ref. DRH-02-62, DRH-02-59 (X-ray diffraction structure ijsf21049)

Synthesis of Pd bipalladacycle complex 25

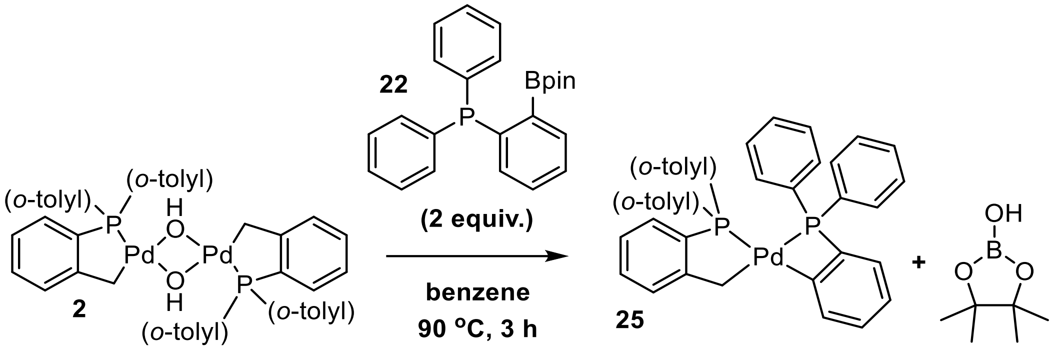

(phenyl-2-pinacol ester)diphenyl phosphine **22** (55 mg, 0.015 mmol, 2.1 eq.) and the $Pd_2(\mu\text{-}OH)_2$ palladacycle **2** (50 mg, 0.007 mmol, 1 eq.) were dissolved in benzene (2 mL, dry, degassed) and sealed under nitrogen in a Schlenk tube. The reaction mixture was heated at 90 °C for 3 hours, before being left to cool to room temperature. The solution was concentrated *in vacuo* to ~ 0.5 mL, then hexane (10 mL, dry, degassed) was added and the mixture was immediately stored at -20 °C for 24 hours. A pale yellow/orange precipitate formed and was isolated by cannula filtration, dried *in vacuo* and stored under argon, (27 mg, 31%). A small amount of this solid was recrystallized (benzene/pentane slow vapor diffusion), giving colorless single crystals of the desired complex **25**. (ATIR): $\tilde{\nu}$ (cm^{-1}) 3053, 1580, 1558, 1463, 1433, 1276, 1157, 1128, 1099, 1060, 1023, 814, 745, 716, 694, 578, 556, 499, 467, 436 (note: mechanically separated single crystals were used for ATIR, and contained oil contamination. This was accounted for by subtracting an oil background from the spectrum, but some C-H stretches may be obscured); ^{1}H NMR (500 MHz, DCM-d_2, 203 K) δ 7.63 (q, *J* = 5.5 Hz, 1H, Ar-*H*), 7.47 (dd, *J* = 7.9, 2.9 Hz, 1H, Ar-*H*), 7.40 (td, *J* = 7.3, 1.4 Hz, 1H, Ar-*H*), 7.35 – 7.29 (m, 5H, Ar-*H*), 7.27 (td, *J* = 7.5, 1.9 Hz, 4H, Ar-*H*), 7.21 – 7.07 (m, 7H, Ar-*H*), 7.05 (t, *J* = 8.1 Hz, 1H, Ar-*H*), 7.00 (t, *J* = 7.4 Hz, 1H, Ar-*H*), 6.98 – 6.93 (m, 1H, Ar-*H*), 6.84 (t, *J* = 7.8 Hz, 1H, Ar-*H*), 3.55 (dd, *J* = 15.0, 10.3 Hz, 1H, C*H*H), 3.37 (q, *J* = 7.1 Hz, 1H, CH*H*), 2.17 (s, 3H, CH_3), 2.01 (s, 3H, CH_3); ^{31}P NMR (202 MHz, DCM-d_2, 203 K) δ 23.25 (d, *J* = 13.1 Hz); ^{13}C NMR (126 MHz, DCM-d_2, 203 K) δ 160.4 (d, *J* = 4.2 Hz, Ar-*C*), 160.1 (d, *J* = 4.1 Hz, Ar-*C*), 159.8, 159.6 (Ar-*C*), 158.8 (d, *J* = 15.7 Hz, Ar-*C*), 150.5 (d, *J* = 4.4 Hz, Ar-*C*), 150.1 (d, *J* = 5.1 Hz, Ar-*C*), 141.4 (d, *J* = 17.4 Hz, Ar-*C*), 141.0 (d, *J* = 13.7 Hz, Ar-*C*), 133.0 (Ar-*C*), 132.8 (d, *J* = 12.8 Hz, Ar-*C*), 132.5 (d, *J* = 6.5 Hz, Ar-*C*), 132.3 (d, *J* = 12.8 Hz, Ar-*C*), 131.7 (Ar-*C*), 131.6 (Ar-*C*), 131.5 (Ar-*C*), 131.3 (Ar-*C*), 131.1 (Ar-*C*), 131.0 (Ar-*C*), 130.8 (Ar-*C*), 130.8 (Ar-*C*), 130.6 (Ar-*C*), 130.1 (Ar-*C*), 129.7 (Ar-*C*), 129.6 (Ar-*C*), 129.5 (Ar-*C*), 129.3 (Ar-*C*), 129.2 (Ar-*C*), 129.1 (Ar-*C*), 128.4 (d, *J* = 9.2 Hz, Ar-*C*), 126.0, 125.9, 125.4 (d, *J* = 5.7 Hz, Ar-*C*), 124.6 (d, *J* = 5.7 Hz, Ar-*C*), 30.5 (CH_2), 22.7 (t, *J* = 8.7 Hz, CH_3), 22.2 (d, *J* = 13.6 Hz, CH_3); HRMS (LIFDI) $(C_{39}H_{34}P_2Pd)^{+\bullet}$ *m/z* (calculated) 670.11651, (found) 670.11943, mass difference 4.35 ppm.

Lab book ref. DRH-02-72, DRH-02-66 (X-ray diffraction structure ijsf21051)

Isolation and Characterization of maleic acid Pd complex 27

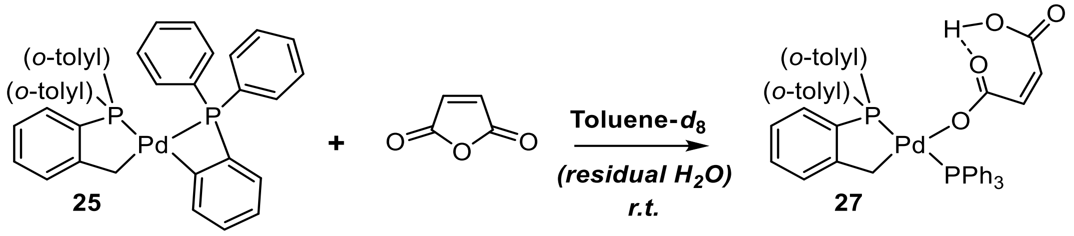

The Pd bipalladacycle **25** (3 mg, 0.0045 mmol, 1 eq.) and maleic anhydride (0.6 mg, 0.0067 mmol, 1.5 eq.) were dissolved in deuterated toluene (0.5 mL, dry, degassed) and sealed under argon. After 15 minutes, the sample was analyzed by NMR spectroscopy. After removal of solvent *in vacuo*, single crystals of **27** suitable for X-ray diffraction were grown by slow vapor diffusion (benzene/pentane); ^{1}H NMR (500 MHz, Toluene-d_8, 203 K) δ 7.65 – 7.57 (m, 5H, Ar-*H*), 7.13 – 7.08 (m, 2H, Ar-*H*), 6.97 – 6.90 (m, 6H, Ar-*H*), 6.84 (t, *J* = 6.9 Hz, 4H, Ar-*H*), 6.75 (dq, *J* = 15.7, 8.4, 7.6 Hz, 2H, Ar-*H*), 6.68 – 6.56 (m, 3H, Ar-*H*), 5.79 (d, *J* = 12.8 Hz, 1H, alkene-C*H*), 5.44 (d, *J* = 12.8 Hz, 1H, alkene-C*H*), 3.41 (d, *J* = 13.0 Hz, 1H, C*H*H), 3.08 (s, 3H, CH_3), 2.56 (m, 4H, (CH_3, CH*H*)); ^{31}P NMR (202 MHz, Toluene-d_8, 203 K) δ 33.58 (d, *J* = 382.7 Hz), 25.47 (d, *J* = 382.7 Hz). (Note: the number of aromatic protons does not match with the expected structure due to integration issues caused by the toluene-d_8 solvent obscuring some peaks) ^{13}C NMR (126 MHz, Toluene-d_8, 298 K) δ 171.3 (O=*C*), 157.8 (d, *J* = 4.8 Hz, Ar-*C*), 157.5 (d, *J* = 4.7 Hz, Ar-*C*), 142.2 (Ar-*C*), 134.1 (dd, *J* = 11.5, 2.1 Hz, Ar-*C*), 133.9 (H*C*=*C*H), 133.1 (Ar-*C*), 132.8 (Ar-*C*), 132.5 (dd, *J* = 5.6, 2.7 Hz, Ar-*C*), 132.0 (Ar-*C*), 131.7 (d, *J* = 9.7 Hz, Ar-*C*), 131.4 (d, *J* = 7.6 Hz, Ar-*C*), 130.7 (d, *J* = 2.5 Hz, Ar-*C*), 130.3(Ar-*C*), 130.2 (d, *J* = 5.3 Hz, Ar-*C*), 130.0 (d, *J* = 2.1 Hz, Ar-*C*), 129.9 (Ar-*C*), 125.5 (d, *J* = 6.4 Hz, Ar-*C*), 23.8 (*C*H_2), 22.0 (*C*H_3). HRMS (LIFDI) $(C_{82}H_{73}O_4P_4Pd_2)^{+\bullet}$ *m/z* (calculated) 1459.25, (found) 1459.27130, mass difference ppm. The suspected structure of the observed mass is shown below.

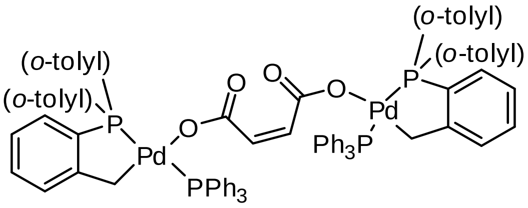

Lab book ref. DRH-02-79 (X-ray diffraction structure ijsf21052)

Synthesis of Structural Analogues of maleic acid Pd complex 27

[Pd(C^P)(OAc)(PPh_3)] SI43

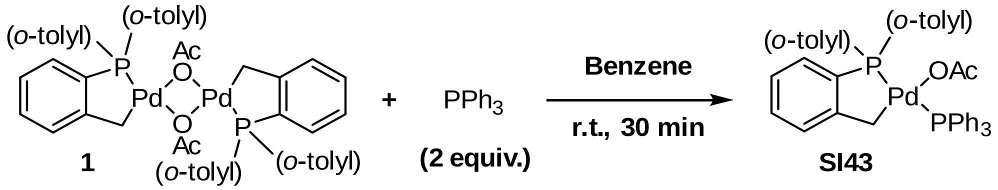

Triphenylphosphine (22.9 mg, 0.0875 mmol, 2.05 eq) was recrystallized from hot methanol before being added to an oven dried Schlenk flask. Herrmann-Beller palladacycle **1** (40 mg, 0.0427 mmol, 1 eq) then dry benzene (4 mL) were added, and the solution was stirred at room temperature for 30 mins. The solvent volume was reduced to ¼ *in vacuo*, then dry hexane (4 mL) at 0 °C was added, resulting in a white precipitate forming. The precipitate was isolated by filtration, washed with hexane (3 ´ 5 mL) then Et_2O (3 mL), giving the product **SI43** as an off-white solid (25 mg, 40%). ^1H NMR (500 MHz, DCM-d_2, 203 K) δ 7.60 – 7.51 (m, 7H, Ar-*H*), 7.49 – 7.34 (m, 12H, Ar-*H*), 7.31 (ddt, *J* = 15.6, 7.5, 3.7 Hz, 2H, Ar-*H*), 7.23 – 7.18 (m, 1H, Ar-*H*), 7.14 (q, *J* = 7.7 Hz, 2H, Ar-*H*), 7.08 (t, *J* = 8.7 Hz, 1H, Ar-*H*), 7.03 (t, *J* = 7.9 Hz, 2H, Ar-*H*), 6.94 – 6.85 (m, 1H, Ar-*H*), 6.78 (t, *J* = 8.2 Hz, 1H, Ar-*H*), 2.92 (s, 4H, CH_3, CH_2), 2.23 (s, 4H, CH_3, CH_2), 1.07 (s, 3H, CO_2CH_3). (Note: the CH_2 palladacycle peaks were obscured by the CH_3 o-tolyl peaks, but HSQC and COSY NMR identified 2 environments for each peak. The number of aromatic protons is higher than expected due to residual benzene solvent); ^{31}P NMR (202 MHz, DCM-d_2, 203 K) δ 30.96 (d, *J* = 391 Hz, *P*Ph_3), 25.27 (d, *J* = 391 Hz, *P*(o-tolyl)$_3$); ^{13}C NMR (126 MHz, DCM-d_2, 203 K) δ 176.2 (*C*O_2CH_3), 158.4 (d, *J* = 33.7 Hz, Ar-*C*), 141.6 (d, *J* = 14.0 Hz, Ar-*C*), 140.8 (d, *J* = 16.8 Hz, Ar-*C*), 134.0 (d, *J* = 12.0 Hz, Ar-*C*), 133.1 (Ar-*C*), 132.5 (Ar-*C*), 132.0 (Ar-*C*), 131.6 (Ar-*C*), 131.4 (Ar-*C*), 130.9 (Ar-*C*), 130.8 (Ar-*C*), 130.4 (Ar-*C*), 130.2 (Ar-*C*), 130.0 (Ar-*C*), 129.8 (Ar-*C*), 129.0 (Ar-*C*), 128.0 (d, *J* = 9.8 Hz, Ar-*C*), 126.9 (Ar-*C*), 125.5 (Ar-*C*), 24.3 (*C*H_2), 23.5 (CO_2*C*H_3), 22.6 (d, *J* = 11.3 Hz, *C*H_3), 20.8 (d, *J* = 8.8 Hz, *C*H_3); HRMS (LIFDI) $(C_{41}H_{38}O_2P_2Pd)^{+\bullet}$ *m/z* (calculated) 730.13764, (found) 730.13677, mass difference -1.19 ppm; (ATIR): $\tilde{\nu}$ (cm^{-1}) 3053 (C-H aromatic), 3004 (C-H aromatic), 2919 (C-H aliphatic), 2857 (C-H aliphatic), 1598 (C=O), 1579 (C=C aromatic), 1467, 1434 (C-P), 1369, 1322, 1282, 1270, 1202, 1185, 1159, 1130, 1095, 1071, 1028, 998, 746, 694, 668, 618, 580, 558, 511, 460, 429; mp. decomposed above 164 °C.

Single crystals suitable for X-ray diffraction were grown from a mixture of Herrmann-Beller palladacycle **1** and triphenylphosphine (1:10 ratio) by slow vapor diffusion (toluene / pentane).

Lab book ref. DRH-03-44, DRH-01-57 (X-ray diffraction structure ijsf2008)

[Pd(C^P)(Cl)(PPh$_3$)] SI44

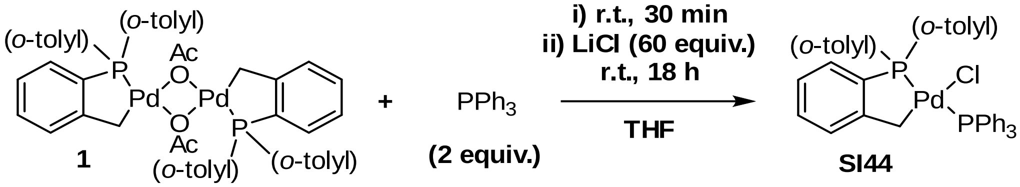

Triphenylphosphine (22.9 mg, 0.0875 mmol, 2.05 eq) was recrystallized from hot methanol before being added to an oven dried Schlenk flask. Herrmann-Beller palladacycle **1** (40 mg, 0.0427 mmol, 1 eq) then toluene (4 mL, dry, degassed) were added, and the solution was stirred at room temperature for 30 mins under N_2. LiCl (72 mg, 1.71 mmol, 40 eq.) was added, followed by water (0.2 mL, degassed), and the reaction was stirred rapidly for a further 1 h. Toluene (10 mL) was added, and the organic layer was washed with water (3 x 10 mL), dried ($MgSO_4$), filtered and the solvent removed in vacuo, giving the product **SI37** as a yellow powder (54 mg, 89%), ^{1}H NMR (500 MHz, DCM-d_2, 203 K) δ 7.57 (t, *J* = 8.5 Hz, 8H, Ar-*H*), 7.41 (dt, *J* = 27.4, 7.5 Hz, 14H, Ar-*H*), 7.31 – 7.18 (m, 1H, Ar-*H*), 7.13 (dt, *J* = 21.6, 7.8 Hz, 4H, Ar-*H*), 6.98 (q, *J* = 8.6, 8.0 Hz, 2H, Ar-*H*), 6.83 (d, *J* = 8.2 Hz, 2H, Ar-*H*), 6.72 (t, *J* = 7.9 Hz, 1H, Ar-*H*), 3.03 (d, *J* = 13.8 Hz, 1H, C*H*H), 2.79 (s, 3H, CH_3), 2.60 (s, 3H, CH_3), 2.20 (t, *J* = 13.4 Hz, 1H, CH*H*) (Note: the number of aromatic protons is higher than expected due to residual toluene solvent peaks); ^{31}P NMR (202 MHz, DCM-d_2, 203 K) δ 32.09 (d, *J* = 415.1 Hz), 30.10 (d, *J* = 415.1 Hz); ^{13}C NMR (126 MHz, DCM-d_2, 203 K) δ 157.3 (dd, *J* = 29.7, 7.1 Hz, Ar-*C*), 142.4 (dd, *J* = 114.9, 12.4 Hz, Ar-*C*), 137.7 (Ar-*C*), 134.3 (d, *J* = 9.3 Hz, Ar-*C*), 132.7 (Ar-*C*), 131.8 (Ar-*C*), 131.7 (Ar-*C*), 131.4 (d, *J* = 9.9 Hz, Ar-*C*), 131.1 (Ar-*C*), 130.9 (d, *J* = 7.4 Hz, Ar-*C*), 130.6 (d, *J* = 30.7 Hz, Ar-*C*), 130.2 (Ar-*C*), 130.1 (Ar-*C*), 129.8 (d, *J* = 8.6 Hz, Ar-*C*), 128.8 (dd, *J* = 30.8, 7.3 Hz, Ar-*C*), 128.6 (Ar-*C*), 128.2 (d, *J* = 11.9 Hz, Ar-*C*), 127.9 (d, *J* = 8.4 Hz, Ar-*C*), 127.8 (Ar-*C*), 127.3 (d, *J* = 19.2 Hz, Ar-*C*), 125.7 (Ar-*C*), 125.4 (d, *J* = 5.9 Hz, Ar-*C*), 124.9 (Ar-*C*), 31.6 (*C*H_3), 23.5 (d, *J* = 7.3 Hz, *C*H_2), 22.5 (d, *J* = 9.8 Hz, *C*H_2); HRMS (LIFDI) $(C_{39}H_{35}P_2ClPd)^{+\bullet}$ *m/z* (calculated) 706.09319, (found) 706.09285, mass difference 0.47 ppm; (ATIR): $\tilde{\nu}$ (cm^{-1}) 3051 (C-H aromatic), 3005 (C-H aromatic), 2953 (C-H aliphatic), 2922 (C-H aliphatic), 2853 (C-H aliphatic, 1580 (C=C aromatic), 1466, 1434 (C-P), 1283, 1265, 1201, 1185, 1118, 1094, 1070, 1028, 998, 908, 805, 747, 727, 692, 589, 508, 459; mp. decomposed above 206 °C.

Lab book ref. DRH-03-46, DRH-01-33 (X-ray diffraction structure ijsf2005)

Synthesis of $Pd_2(Br)_2(C_6H_4F)_2[P(o\text{-tolyl})_3]_2$ oxidative addition dimer (SI45)

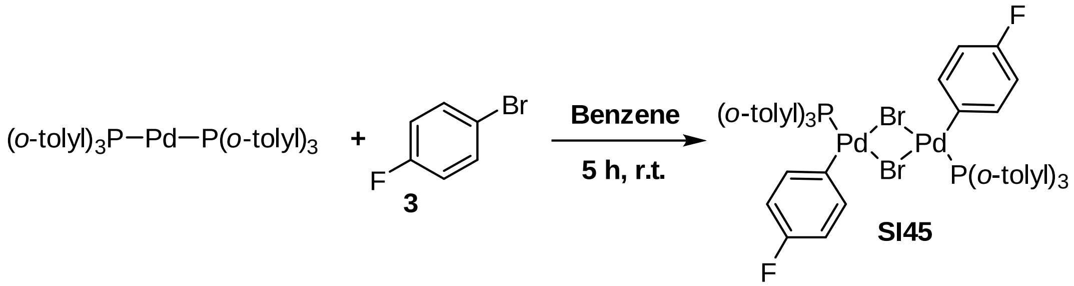

Method adapted from Hartwig *et al.*[5]

Bis(tri(o-tolyl)phosphine)palladium(0) (50 mg, 0.070 mmol, 1 eq.) and 1-bromo-4-fluorobenzene **3** (38 µL, 0.35 mmol, 5 eq.) were added to benzene (2 mL, dry, degassed) under N_2. The resulting mixture was rapidly stirred for 5 hours, after which the solvent was evaporated to ~ 0.5 mL and diethyl ether (10 mL, dry, degassed) was added to precipitate the compound. The flask was stored at -20 ºC for 2 days to allow complete precipitation, after which the solvent was filtered off and the precipitate dried under vacuum to give the product **SI45** as a yellow powder (19 mg, 46 %). Single crystals suitable for X-ray diffraction were obtained by slow vapor diffusion from DCM / pentane; (upon cooling to 203 K, cis and trans isomers of the complex were observed – all relevant peaks are reported here) ^{1}H NMR (500 MHz, DCM-d_2, 203 K) δ 7.73 – 7.58 (m, 2H, Ar-*H*), 7.59 – 7.40 (m, 6H, Ar-*H*), 7.31 – 7.17 (m, 6H, Ar-*H*), 7.17 – 6.90 (m, 12H, Ar-*H*), 6.68 – 6.45 (m, 4H, Ar-*H*), 6.25 – 5.98 (m, 3H, *Ar-H*), 3.73 (s, 2H, tolyl-*H*), 3.64 – 3.56 (m, 1H, tolyl-*H*), 3.54 – 3.43 (m, 2H, tolyl-*H*), 3.25 (s, 1H, tolyl-*H*), 3.18 (s, 1H, tolyl-*H*), 2.30 (s, 1H, tolyl-*H*), 2.12 – 2.00 (m, 3H, tolyl-*H*), 1.50 – 1.33 (m, 7H, tolyl-*H*); ^{31}P NMR (202 MHz, DCM-d_2, 203 K) δ 29.21, 26.64; ^{19}F NMR (470 MHz, DCM-d_2, 203 K) δ -123.06, -123.33; ^{13}C NMR (151 MHz, DCM-d_2, 298 K) δ 160.7 (d, J = 240.4 Hz, *C*-F), 143.5 (*C*-P), 136.5 (*C*-CH_3), 135.0 (Ar-*C*), 133.4 (Ar-*C*), 132.3 (o-tolyl-*C*), 131.3 (o-tolyl-*C*), 126.5 (Ar-*C*), 126.0 (o-tolyl-*C*), 113.1 (d, J = 19.6 Hz, (o-tolyl-*C*)), 23.8 (*C*H_3); (ATIR): $\tilde{\nu}$ (cm^{-1}) 3060 (C-H aromatic), 2973 (C-H aliphatic), 2855 (C-H aliphatic), 1567 (C=C aromatic), 1476, 1447 (C-P), 1274, 1215 (s, C-F), 1118, 1007, 817, 755, 716, 680, 564, 533, 475, 465; Far IR (ATIR) $\tilde{\nu}$ (cm^{-1}) 305, 291 (Pd-Br), 255, 245, 214; mp. decomposed above 175 °C.

Note: HRMS was not possible for this complex as it decomposed under LIFDI conditions. A m/z of 979.91241 (highest peak) corresponding to a complex containing 2 Pd atoms was observed, and it is likely that this is the $Pd_2(\mu_2\text{-}Br)_2$ palladacycle **30**, which could form under LIFDI conditions *via* elimination of two C_6H_5F groups.

Lab book ref. DRH-02-50-1 (X-ray diffraction structure ijsf21047), DRH-02-51-2

Synthesis of aryl iodide immobilised on a polystyrene resin 28 for the 3-phase test

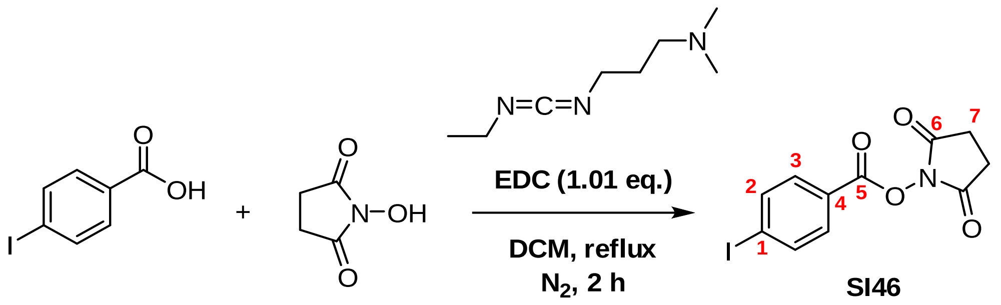

Method adapted from Shell and Mohler.[6]

4-Iodobenzoic acid (1283 mg, 5.18 mmol, 1.01 eq.) and *N*-hydroxysuccinimide (590 mg, 5.12 mmol, 1 eq.) were added to a dry flask and purged with N_2. DCM (100 mL, dry, degassed) was added, followed by EDC (986 mg, 5.18 mmol, 1.01 eq.). The resulting mixture was refluxed at 50 °C for 2 h, then allowed to cool. The mixture was washed with sat. $NaHCO_3$ (3 × 15 mL), water (3 × 15 mL), HCl (1M, 1 × 15 mL), dried ($MgSO_4$), filtered then concentrated under vacuum to give the product **SI46** as an off-white solid (1440 mg, 82%). ^{1}H NMR (500 MHz, C*D*Cl_3, 298 K) δ 7.93 – 7.86 (m, 2H, H-3), 7.86 – 7.79 (m, 2H, H-2), 2.93 – 2.89 (m, 4H, H-7); ^{13}C NMR (126 MHz, C*D*Cl_3, 298 K) δ 169.2 (C-6), 161.7 (C-5), 138.5 (C-3), 131.8 (C-2), 124.7 (C-4), 103.5 (C-1), 25.8 (C-7); HRMS (ESI$^+$) $(C_{11}H_8NO_4Na)^+$ *m/z* (calculated) 367.9387, (found) 367.9390, mass difference 0.5 ppm; (ATIR): $\tilde{\nu}$ (cm^{-1}) 2952 (C-H aromatic), 2923 (C-H aromatic), 2854 (C-H aliphatic), 1768 (s, C=O ester), 1716 (s, C=O amide), 1583 (C=C aromatic), 1478, 1423, 1372, 1203 (C-C aliphatic), 1074, 1049, 1001, 843, 815, 735, 688, 641, 604, 553,486, 460, 426; mp 222 °C (decomposed). Data matches literature for this compound.[6]

Lab book ref. DRH-03-66

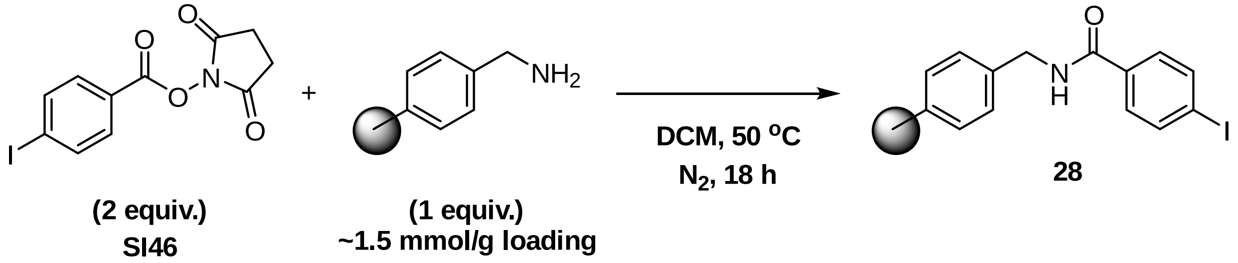

To an oven dried screw-cap vial, aminopolyester resin (200-400 mesh, ~1.5 mmol/g N loading, 333 mg, 0.5 mmol, 1 eq.) and the aryl-succinimide compound **SI46** (345 mg, 1.0 mmol, 2 eq.) were added, and purged with N_2. DCM (3 mL, dry, degassed) was added, and the vial shaken by thermoshaker (500 rpm) at 50 °C for 18 h. The solid was isolated by filtration, washed with DCM (3 × 10 mL) then Et_2O (5 × 5 mL), giving the

product **28** as an insoluble fine powder (411 mg). (ATIR): $\tilde{\nu}$ (cm^{-1}) 1644 (strong, broad), indicating a C=O amide bond; 3306 (br, N-H), 3058 (C-H aromatic), 3024 (C-H aromatic), 2920 (C-H aliphatic), 2849, 1703 (C=O amide), 1586 (C=C aromatic), 1511, 1492, 1476, 1451, 1298, 1234, 1111, 1062, 1006, 989, 839, 750, 750, 607, 537.

Lab book ref. DRH-03-68

Synthesis of 3-phase test substrate (unbound version 29)

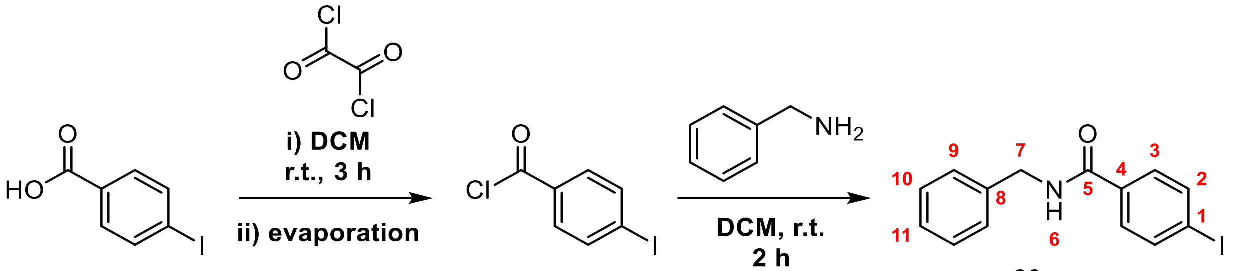

29

4-Iodobenzoic acid (1000 mg, 4.03 mmol, 1 eq.) was suspended in DCM (16 mL) in a flask fitted with a drying tube ($CaCl_2$). Oxyl chloride (0.72 mL, 8.27 mmol, 2.05 eq.) was added dropwise, then one drop of DMF was added. The resulting mixture was stirred at r.t. for 3 h. The solvent and excess oxalyl chloride were removed *in vacuo*, and the residue redissolved in DCM (10 mL). Benzylamine (0.66 mL, 6.05 mmol, 1.5 eq.) then triethylamine (0.75 mL, 5.40 mmol, 1.34 eq.) were added, and the solution stirred at r.t. for 2 h. The solution was poured into EtOAc (100 mL) and washed sequentially with H_2O (50 mL), 10% aqueous HCl (20 mL), sat. aqueous $NaHCO_3$ solution (20 mL) and brine (50 mL). The organic phase was dried ($MgSO_4$), filtered and concentrated *in vacuo*. The residue was recrystallized from hot EtOH to give the product **29** as colorless needles (859 mg, 63%); ^{1}H NMR (600 MHz, C*D*Cl_3, 298 K) δ 7.79 – 7.72 (m, 2H, H-3), 7.53 – 7.47 (m, 2H, H-2), 7.38 – 7.29 (m, 5H, H-9, H-10, H-11), 6.46 (s, 1H, H-6), 4.61 (d, *J* = 5.7 Hz, 2H, H-7); ^{13}C NMR (151 MHz, C*D*Cl_3, 298 K) δ 166.7 (C-5), 138.0 (C-8), 137.9 (C-2), 133.9 (C-4), 129.0 (C-3), 128.7 (C-10), 128.1 (C-9), 127.9 (C-11), 98.6 (C-1), 44.4 (C-7); (ESI$^+$) ($C_{14}H_{13}INO$)$^+$ *m/z* (calculated) 338.0036, (found) 338.0035, mass difference 0.4 ppm; (ATIR): $\tilde{\nu}$ (cm^{-1}) 3310 (br, N-H), 3082 (C-H aromatic), 3056 (C-H aromatic), 3026 (C-H aromatic), 2933 (C-H aliphatic), 1637 (C=O amide), 1582, 1544 (C=C aromatic), 1476, 1449, 1419, 1316, 1302, 1257, 1186, 1150, 1005, 989, 844, 755, 728, 697, 665 (s, C-I), 517, 474, 456, 442; mp 172.0 – 174.2 °C (from ethanol). Data matches literature for this compound.[7]

Lab book ref. DRH-03-90

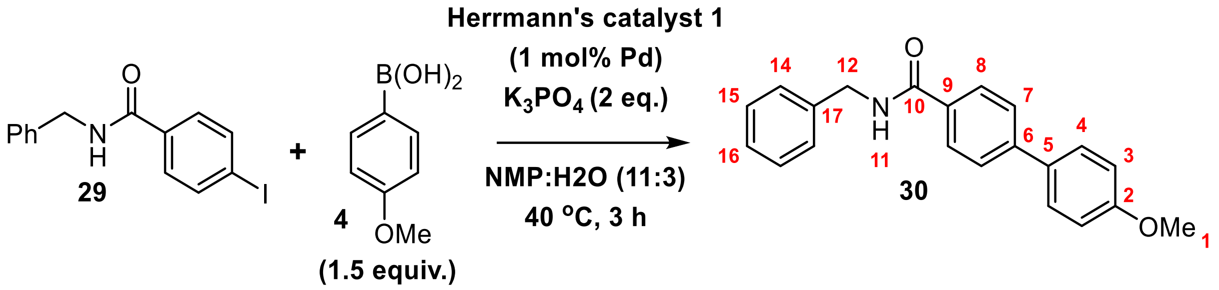

Reaction performed following General Procedure 2.2 in a Schlenk flask (half scale, 0.6 mmol aryl halide **29**, 0.214 M effective concentration). Conversion to product **30** (55%) was calculated from ^{1}H NMR of crude reaction mixture, isolated yield (45 mg, 24%) Note: due to poor solubility in mobile phase selected for manual column chromatography (Rf = 0.12, 40% EtOAc in pet. Ether), solid loading was required.

^{1}H NMR (600 MHz, C*D*Cl_3, 298 K) δ 7.87 – 7.82 (m, 2H, H-8), 7.63 – 7.58 (m, 2H, H-7), 7.58 – 7.52 (m, 2H, H-4), 7.40 – 7.34 (m, 4H, H-14, H-15), 7.36 – 7.28 (m, 1H, H-16), 7.02 – 6.96 (m, 2H, H-3), 6.44 (s, 1H, H-11), 4.67 (d, *J* = 5.6 Hz, 2H, H-12), 3.86 (s, 3H, H-1); ^{13}C NMR (151 MHz, C*D*Cl_3, 298 K) δ 167.2 (C-10), 159.9 (C-2), 144.1 (C-5), 138.4 (C-17), 132.6 (C-9), 132.5 (C-6), 129.0 (C-4), 128.4 (C-15), 128.1 (C-14), 127.8 (C-16), 127.6 (C-8), 126.9 (C-7), 114.5 (C-3), 55.5 (C-1), 44.3 (C-12); (ESI$^+$) $(C_{21}H_{19}NO_2Na)^+$ *m/z* (calculated) 340.1308, (found) 340.1316, mass difference 2.3 ppm; (ATIR): $\tilde{\nu}$ (cm^{-1}) 3325 (br, N-H), 3063 (C-H aromatic), 3032 (C-H aromatic), 3008 (C-H aromatic), 2955 (C-H aromatic), 2922 (C-H aromatic), 2836 (C-H aliphatic), 1633 (C=O amide), 1603, 1535 (C=C aromatic), 1492 (C=C aromatic), 1453, 1291, 1252 (C-O), 1205, 1181, 1079, 1034, 1012, 899, 825, 770, 738, 696, 607, 523, 483; mp 190.0 – 191.5 °C. This is a novel compound.

Lab book ref. DRH-03-95

2.4 Experimental Details for 3-Phase Test

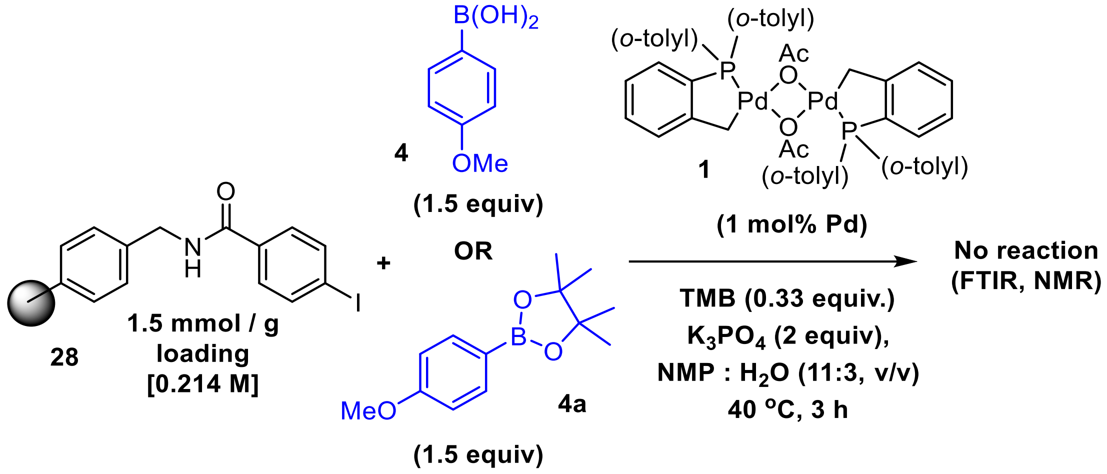

Following the General Procedure 2.2 for SMCC reactions at quarter scale (0.3 mmol aryl halide, 0.214 M effective concentration), the aryl halide was replaced with the resin immobilsed aryl iodide **28**, with 1,3,5-trimethoxybenzene (TMB) used as an internal standard for ^{1}H NMR analysis. After 3 h, a direct aliquot (0.5 mL of filtered reaction mixture) was taken for ^{1}H NMR analysis, and the consumption of aryl boronic acid was calculated w.r.t. the internal standard. ^{1}H COSY NMR was used to determine discrete species, supported by ^{1}H NMR spectra of authentic samples of the potential species. The resin was washed with water and DCM, and the recovered resin was analyzed by FTIR and MAS NMR compared to starting resin.

3-phase Test Data using Arylboronic acid 4

Lab book ref. DRH-03-74

Table 1: ^{1}H NMR integrals (600 MHz, unlocked, 20 s relaxation delay, 4 s acquisition time, 298 K), and calculated mmol of species generated from the boronic acid 4. Lab book ref. DRH-03-74

Species	Shift / ppm	Integral	Integral / H environments	Relative quantity / mmol
TMB	6.11	3.00	1.00	0.0996
Anisole	7.32	3.33	1.665	0.166
arylboronic acid **4**	6.71	0.64	0.32	0.032
arylboronate of **4** ($Ar-B(OH)_3^-$)	7.84	4.07	2.035	0.203
Unknown 1	8.00	0.80	0.40	0.040
Unknown 2	7.43	0.53	0.265	0.026
			**Sum / mmol**	**0.467**
			**mmol boronic acid added**	**0.45**

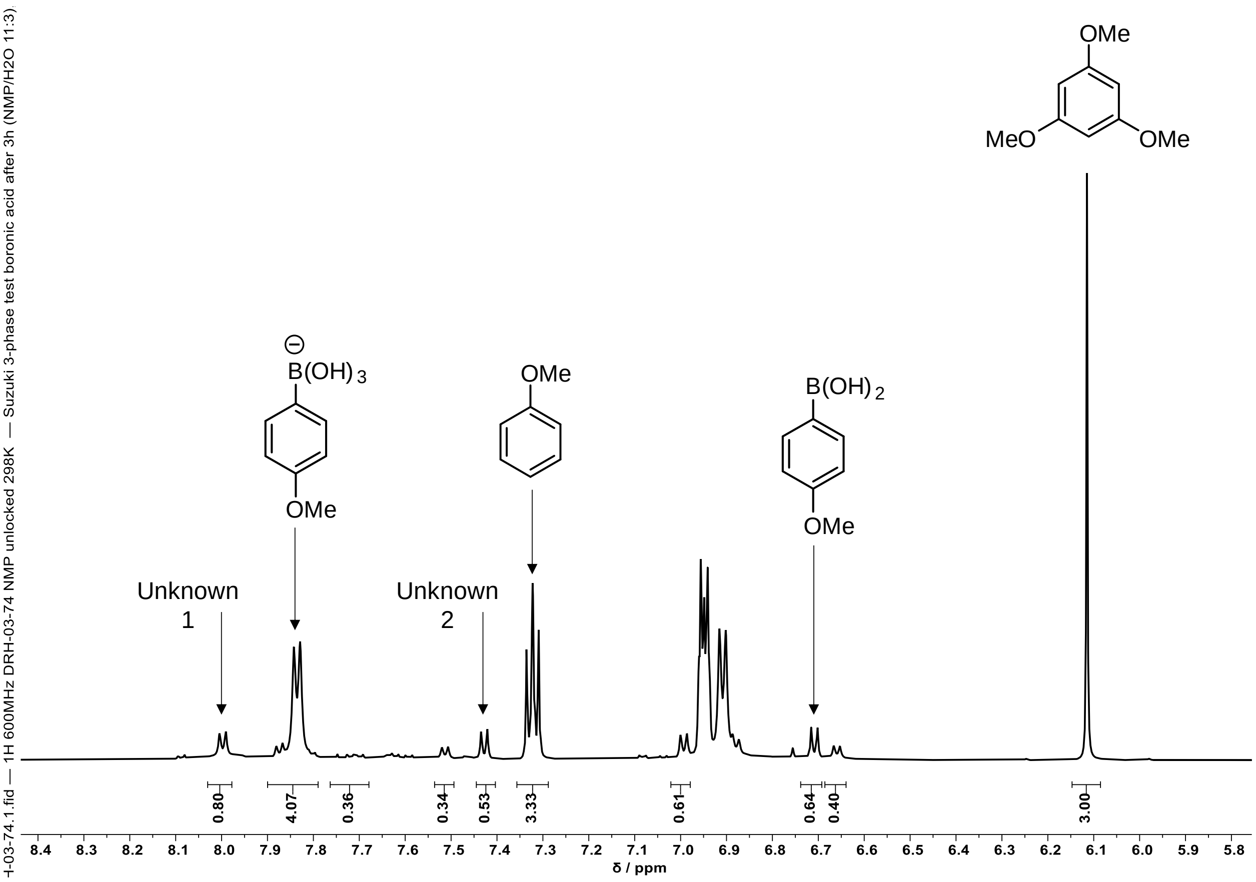

Figure 1: ^{1}H NMR magnified aromatic region (600 MHz, NMP-unlocked, 20 s relaxation delay, 4 s acquisition time, 298 K) of the direct aliquot of the 3-phase test reaction mixture using arylboronic acid 4. The aliquot was taken after 3 h reaction. Lab book ref. DRH-03-74

As there is no change in the recovered resin by FTIR and the calculated mmol quantities of species generated by the arylboronic acid are roughly equivalent to the mmol of arylboronic acid added, there has been no cross-coupling reaction.

3-phase Test Data using Arylboronic pinacol ester 4a

Lab book ref. DRH-03-71

Table 2: ^{1}H NMR integrals and calculated mmol of species generated from the arylboronic pinacol ester 4a. Lab book ref. DRH-03-71

Species	Shift / ppm	Integral	Integral / H environments	Relative quantity / mmol
Trimethoxybenzene	6.11	3.00	1.00	0.937
Anisole	7.32	3.87	1.935	0.181
Arylboronic pinacol ester **4a**	7.83	5.16	2.58	0.242
Arylboronate pinacol ester (Ar-Bpin(OH)$^-$)	7.66	4.26	2.13	0.200
Unknown 1	6.71	0.38	0.19	0.018
Unknown 2	6.62	0.55	0.275	0.026
			**Sum / mmol**	**0.666**
			**mmol pinacol ester added**	**0.45**

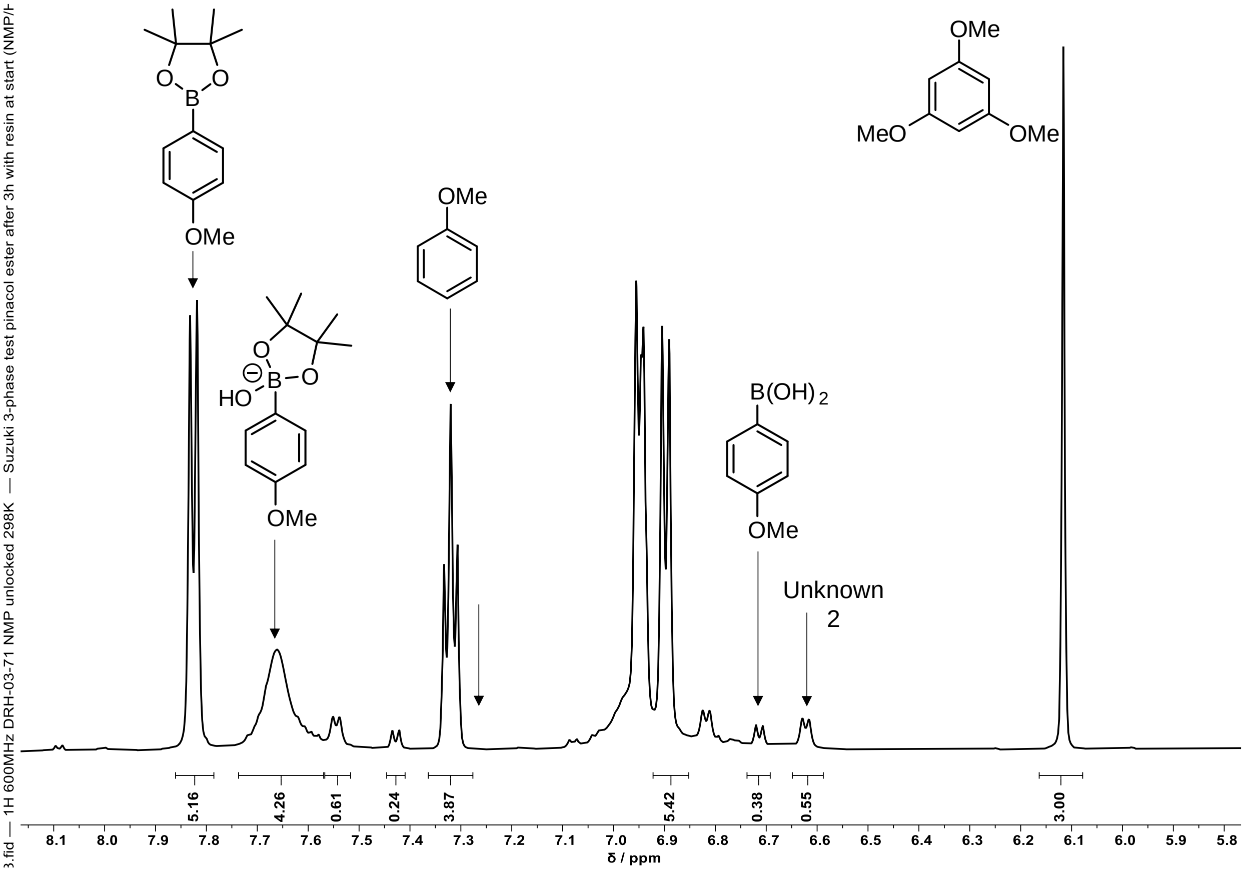

Figure 2: ^{1}H NMR magnified aromatic region (600 MHz, NMP-unlocked, 298 K) of the direct aliquot of the 3-phase test reaction mixture using arylboronic pinacol ester 4a. The aliquot was taken after 3 h reaction. Lab book ref. DRH-03-71

As there was no change in the recovered resin by FTIR (amide stretch at 1650 cm^{-1}) and the calculated mmol quantities of species generated by arylboronic pinacol ester **4a** are higher than the theoretical amount added, it was assumed that there was no cross-coupling reaction. The reason for the higher calculated mmols is likely the signal broadening by the arylboronate pinacol ester species, which led to inflated integral values for other species.

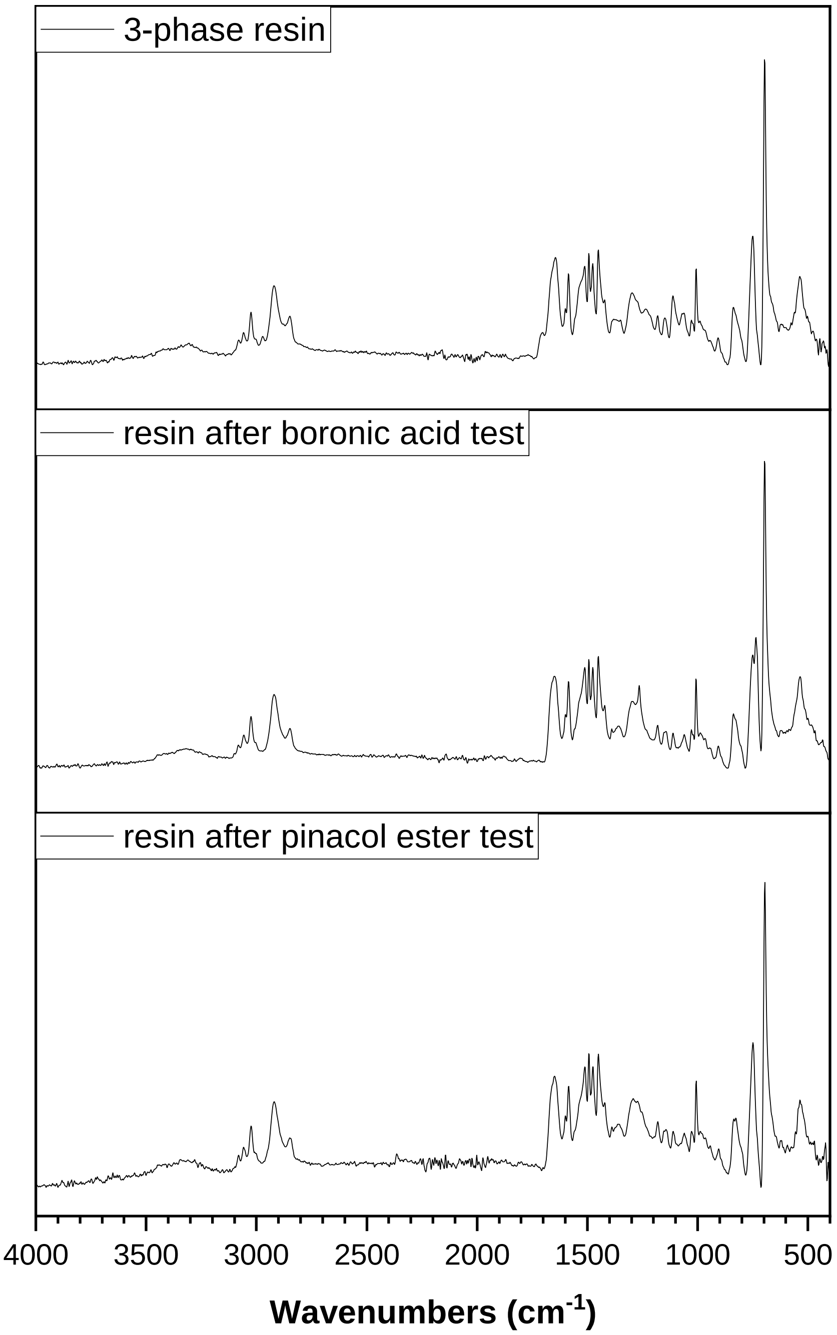

Figure 3: Stacked FTIR data showing the recovered resin from the 3-phase tests in comparison with the starting material 28.

Magic angle spinning (MAS) NMR of the polystyrene-based resins was undertaken to confirm that no reaction had occurred. As can be seen in Figure 4, the incorporation of two new peaks at ^{13}C δ 99 ppm and 167 ppm show that the resin-immobilised substrate **28** was successfully synthesized. Upon reaction with arylboronic acid **4** or arylboronic pinacol ester **4a**, the structure of the resin by MAS NMR remained

unchanged, confirming that no reaction had occurred. For a successful reaction, the C-I carbon signal at 99 ppm would be expected to disappear.

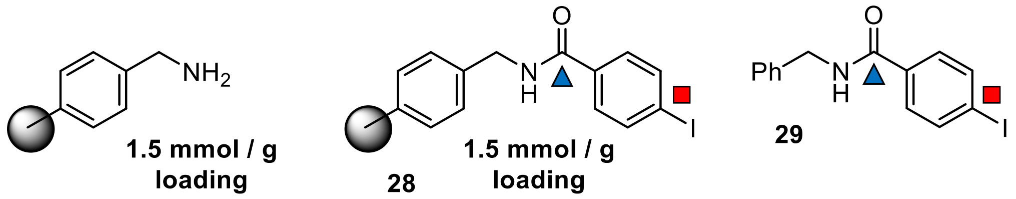

Scheme 1: Structures of the polystyrene resin starting material, substrate immobilised on the same resin 28, and the unbound analogous substrate 29. The diagnostic carbon environments detected by NMR are denoted by a blue triangle (amide *C*=O), and red square (*C*-I).

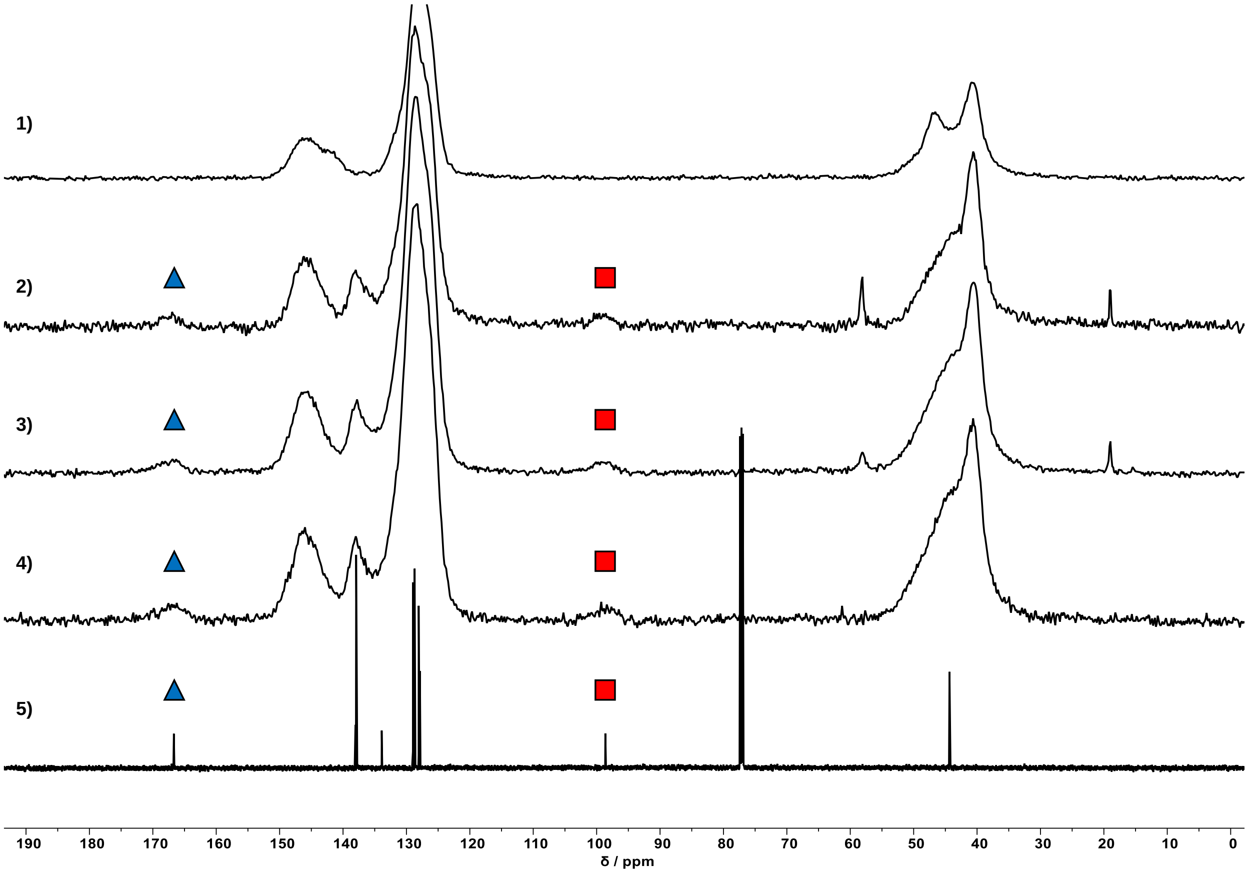

Figure 4: MAS ^{13}C NMR (100 MHz, 20 kHz) of 1) polystyrene resin starting material (lab book ref. DRH-03-68), 2) immobilised substrate 28 (lab book ref. DRH-03-68-2), 3) recovered resin after boronic acid reaction (lab book ref. DRH-03-74), 4) recovered resin after pinacol ester reaction (lab book ref. DRH-03-71), 5) solution phase NMR of unbound resin 29 for confirmation of peak identity (lab book ref. DRH-03-90). Structures of the relevant species are shown in Scheme 1. The diagnostic carbon environments detected by NMR are denoted by a blue triangle (amide *C*=O), and red square (*C*-I).

2.5 Synthesis and Characterization of $[Pd(P^\wedge C)(\mu_2\text{-}Ar^F)]_2$ palladacycle 16a

Synthesis of Ag-C_6F_5 complex 18

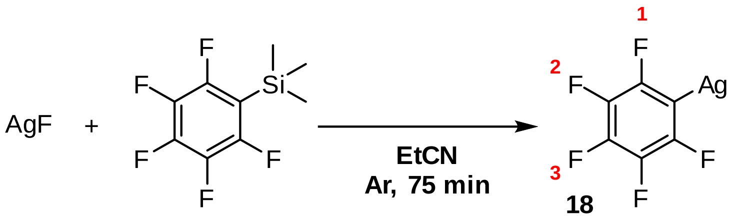

Complex prepared according to a literature procedure by Athavan *et al.*[8]

In an argon glovebox, AgF (302 mg, 2.38 mmol, 1.0 eq.) and dry, degassed EtCN (5 mL) were stirred for 5 min at room temperature under darkness. $Me_3SiC_6F_5$ (450 µL, 2.38 mmol, 1.02 eq.) was added, and the resulting mixture was stirred for a further 75 min. The reaction was removed from the glovebox, and volatiles removed *in vacuo*, giving the product as a pale grey powder (442 mg, 68%). (Note: this complex is unstable in air and sensitive to light and temperature, so was stored in an argon glovebox freezer in a foiled vial) ^{19}F NMR (471 MHz, Acetonitrile-d_3) δ -106.93 (m, 2F, F-1), -159.80 (t, J = 19.2 Hz, 1F, F-3), -163.13 (m, 2F, F-2). The ^{19}F NMR data matches the literature.[8]

Lab book ref. DRH-03-73

Synthesis of $[Pd(P^\wedge C)(\mu_2\text{-}Ar^F)]_2$ palladacycle 16a

Lab book ref. DRH-03-76, DRH-03-79

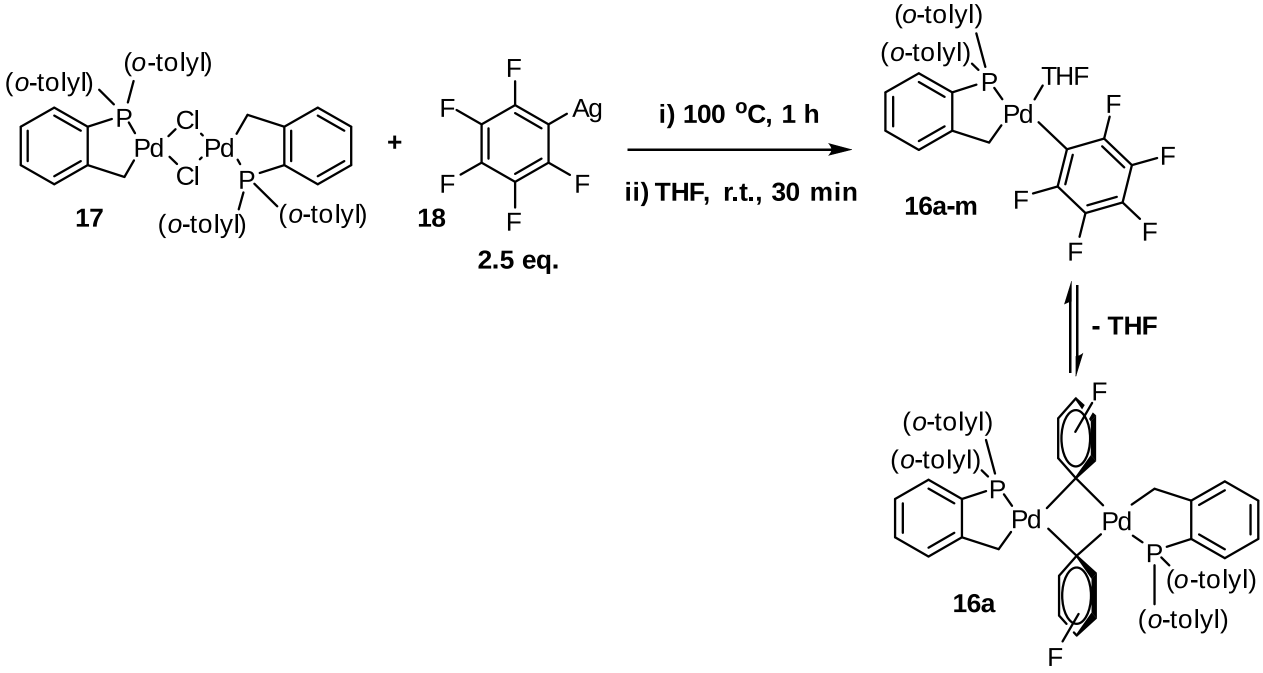

The synthesis of this complex was based on that from Espinet's work, where bridging Pd-Ar-Pd complexes were synthesized and characterised.[9] The complex is highly water sensitive, rapidly decomposing to Pd black and pentafluorobenzene when exposed to water.

In an argon glovebox, Ag-C_6F_5 complex **18** (47 mg, 0.141 mmol, 2.1 equiv.) was charged into a Schlenk flask under darkness. The complex was heated at 100 °C under vacuum for 1 h to remove any coordinating EtCN ligands, then allowed to cool to room temperature. $[Pd(P^\wedge C)(\mu_2\text{-}Cl)]_2$ palladacycle **17** (60 mg, 0.0674 mmol) was added under N_2, followed by THF (6 mL, dry, degassed). The colorless mixture was stirred for 30 min at room temperature, after which a direct NMR aliquot was taken for analysis. The mixture was allowed to settle, and the liquor isolated by canula filtration. The solvent was removed *in vacuo*, giving a dark red residue. This residue was analyzed by NMR (C_6D_6, dry, degassed), and ~10 mg was set up for recrystallization overnight (C_6D_6/pentane) under argon (slow vapor diffusion). A small quantity of orange-yellow crystals of **16a** suitable for single crystal XRD were obtained, and these were analyzed by FTIR and mass spectrometry.

The bridging Pd-Ar bond is weak and easily cleaved by even a mildly coordinating solvent. As such, in THF, there is exclusively the monomeric complex **16a-m**, likely coordinated with THF. As the coordinating solvent is removed, the more stable dimer **16a** is formed, and this is what crystallizes out. Analysis in dry Benzene-d_6 reveals that even in non-coordinating solvent, the monomer forms. Over longer time periods (ca. 2 days) degradation of the complex is observed, which has made larger scale synthesis and purification impossible. LIFDI mass spectrometry analysis shows only the monomer present, as the dimer **16a** likely cleaves under mass spectrometry conditions.

Mixture of monomer and dimer in Benzene-d_6; ^{31}P NMR (243 MHz, Benzene-d_6, 298 K) δ 53.17 (dimer), 26.09 (monomer); ^{19}F NMR (565 MHz, Benzene-d_6, 298 K) δ -95.92 (dimer), -113.77 (d, J = 35.5 Hz, monomer), -145.28 (dimer), -160.79 – -161.08 (m, dimer/monomer), -162.34 – -162.55 (m, dimer/monomer).

16a-m in THF: ^{31}P NMR (203 MHz, THF-unlocked, 298 K) δ 25.60 (tt, J = 12.2, 9.2 Hz); ^{19}F NMR (471 MHz, THF-unlocked, 298 K) δ -114.77 (dt, J = 34.2, 9.6 Hz), -163.80 (d, J = 21.5 Hz), -164.54.

HRMS (LIFDI) $(C_{27}H_{20}F_5PPd)^{+\bullet}$ *m/z* (calculated) 576.02521, (found) 576.02786, mass difference 4.59 ppm. (ATIR): $\tilde{\nu}$ (cm^{-1}) 3060 (C-H aromatic), 2925 (C-H aliphatic), 2857 (C-H aliphatic), 1624 (C=C aromatic), 1590 (C=C aromatic), 1580, 1492 (C-P), 1441 (s, C-F), 1320, 1282, 1263, 1246, 1203, 1132, 1048, 951, 803, 752, 713, 671, 577, 559, 521, 507, 460.

Lab book ref. DRH-03-76, DRH-03-79 (X-ray diffraction structure ijsf22048)

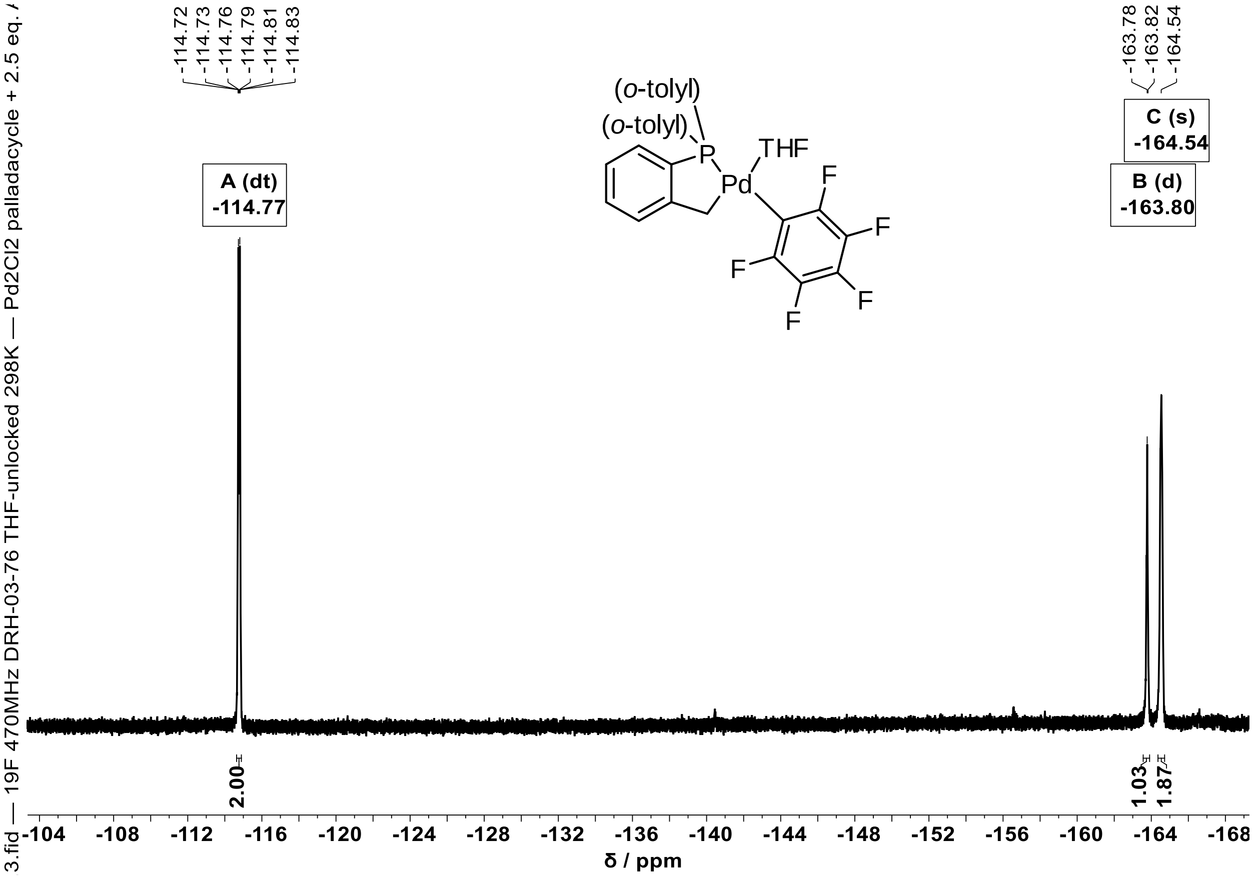

Figure 5: ^{19}F NMR (470 MHz, THF-unlocked, 128 sc, 298 K) of the [Pd(P^C)(Ar^F)] palladacyclic monomer (16a-m), with suspected THF coordination. Lab book ref. DRH-03-76

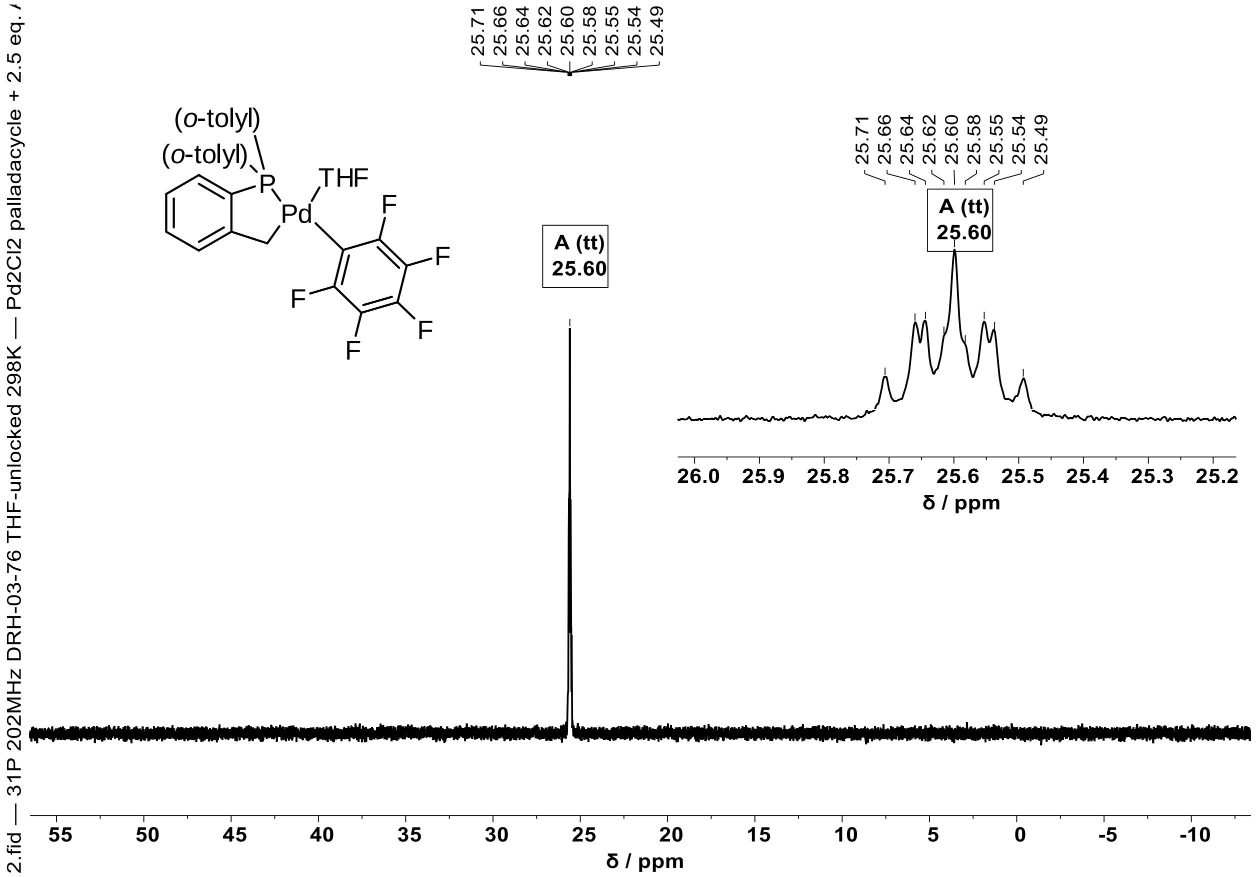

Figure 6: ^{31}P NMR (203 MHz, THF-unlocked, 128 sc, 298 K) of the [Pd(P^C)(Ar^F)] palladacyclic monomer with suspected THF coordination. Lab book ref. DRH-03-76

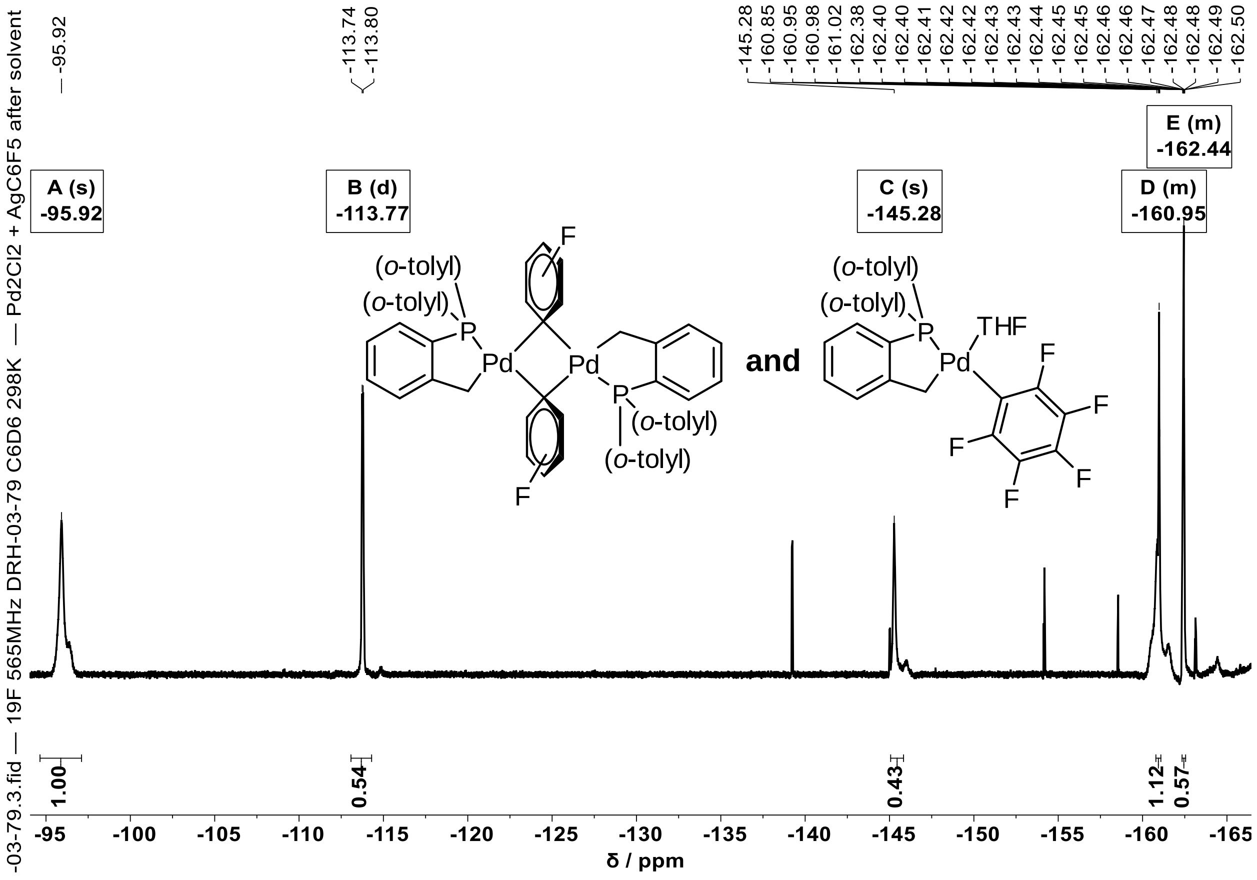

Figure 7: ^{19}F NMR (565 MHz, C_6D_6, 128 sc, 298 K) of the [Pd(P^C)(μ_2-Ar^F)]$_2$ palladacycle 16a and the [Pd(P^C)(Ar^F)] palladacyclic monomer 16a-m in solution. Lab book ref. DRH-03-79

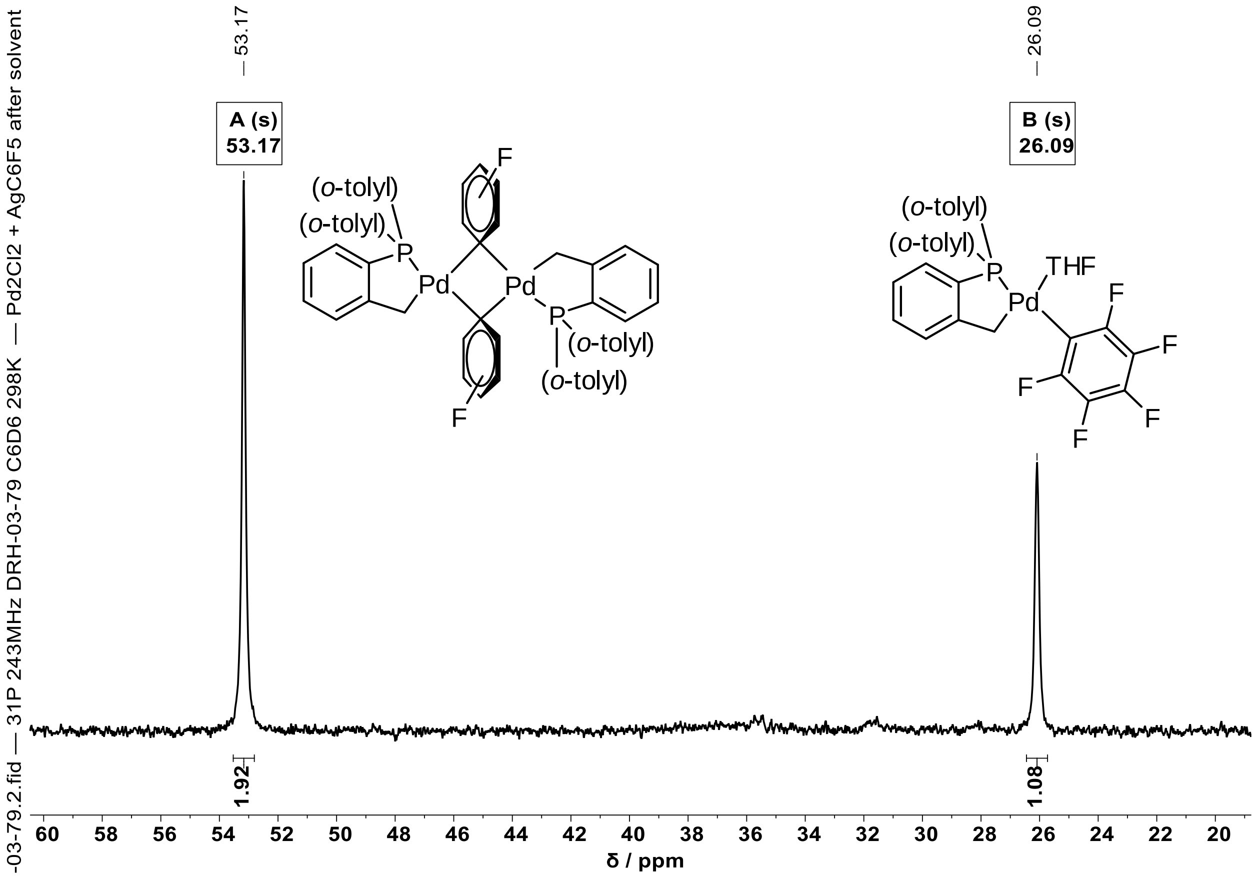

Figure 8: **^{31}P NMR (243 MHz, C_6D_6, 128 sc, 298 K) of the [Pd(P^C)(μ$_2$-Ar^F)]$_2$ palladacycle 16a and the [Pd(P^C)(Ar^F)] palladacyclic monomer 16a-m in solution. Lab book ref. DRH-03-79**

4. *In situ* IR Data

The experimental data from in situ IR monitoring (product formation) was fitted in OrginPro 2022b (64-bit) SR1 9.9.5.171 using the Hill equation with the Levenburg Marquardt iteration algorithm:

$$y = \frac{V_{max}x^n}{(k^n + x^n)}$$

Where V_{max} is the maximum concentration of product (mol dm^{-3}), k is the time at which 50% of V_{max} value is achieved (h), n is the Hill coefficient (unitless), y is the product concentration (mol dm^{-3}), and x is time (s). 95% confidence intervals were used to calculate errors in fitting.

The value of k can be used as a measure of reaction rate, with a small value of k indicating a rapid reaction. The value of n indicates the level of cooperativity in the reaction. For example, when n>1, there is positive cooperativity with respect to the reagent binding the catalyst. This means that the catalyst has more than one binding site, and the kinetic profile will appear sigmoidal with an induction period. When n=1, there is one binding site in the catalyst, and the Hill equation simplifies to the Michaelis-Menten equation. When n<1, there is negative cooperativity with respect to the reagent binding the catalyst. Practically for Pd chemistry, this is an indication of aggregation or off-cycle reactions, as the number of catalyst molecules required to do each reaction is increased. All fitting done on these reactions falls into the n<1 situation to varying degrees.

$$1)\ n = 1 \quad Pd + [S] \rightarrow P$$

$$2)\ n = 2 \quad Pd + 2[S] \rightarrow 2P$$

$$3)\ n = 0.5 \quad 2Pd + [S] \rightarrow P$$

For a high-level assessment of reaction rates, the Hill equation fitting gives a good comparison of the efficacy of catalysts. However, due to the inherent complexity of the system, the co-operativity value of n can be misleading and should not be used to infer catalyst behavior.

4.1 Heck Reaction Profiles

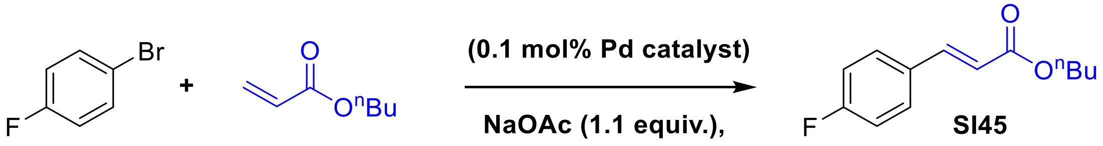

10 mmol [1.0 M] 3

(1.2 equiv.) SI44

SI45

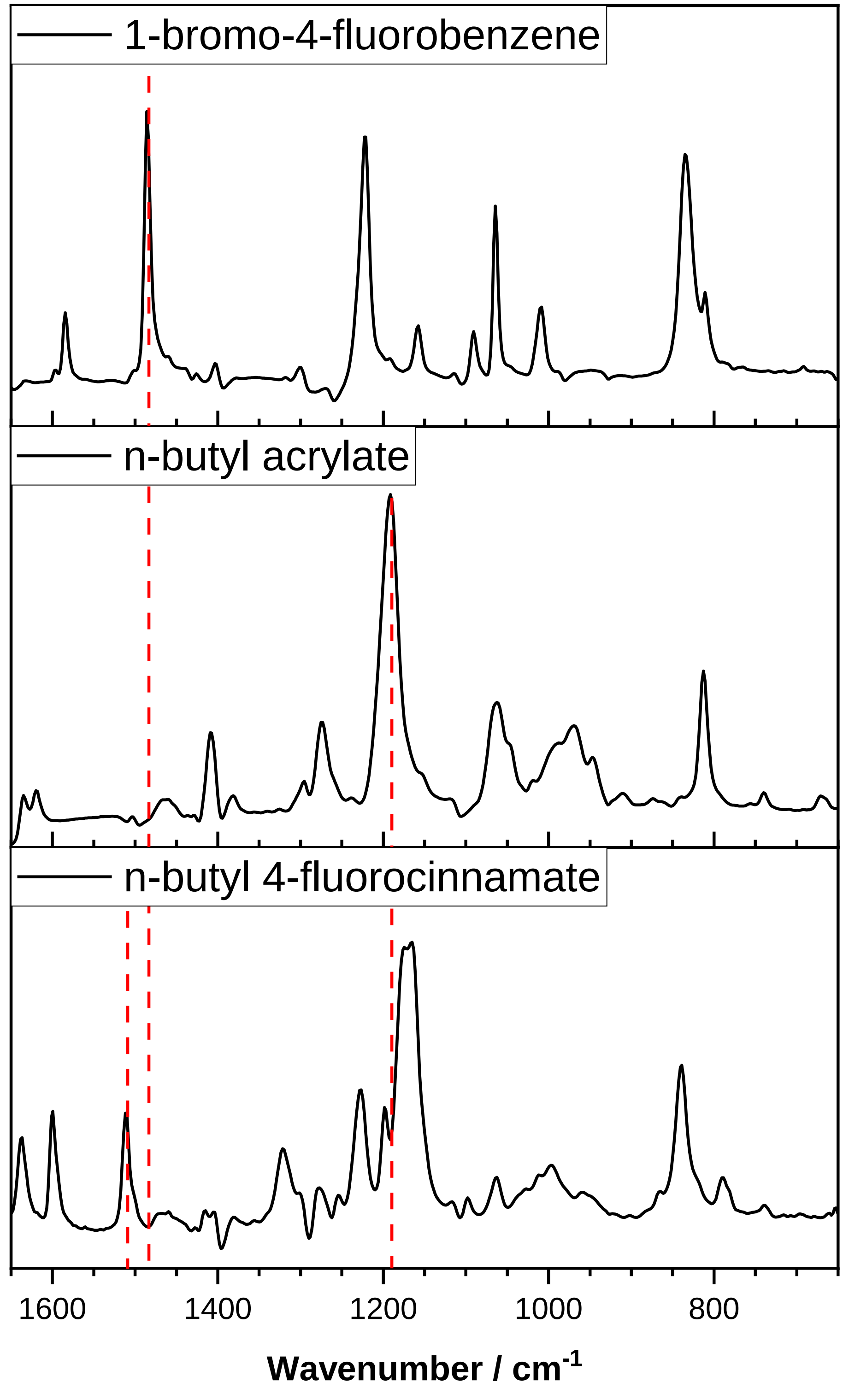

Figure 9: Reference spectra of (top) 1-bromo-4-fluorobenzene 3, (middle) n-butyl acrylate SI41, (bottom) n-butyl 4-fluorocinnamate product SI42 in NMP. Spectra taken using ReactIR 15 (Diamond probe) at 23 °C. The key peaks that

are followed by IR during the reaction are indicated by the dashed red lines (1484 cm^{-1} for 1-bromo-4-fluorobenzene 3, 1190 cm^{-1} for n-butyl acrylate SI41 and 1509 cm^{-1} for n-butyl 4-fluorocinnamate SI42.

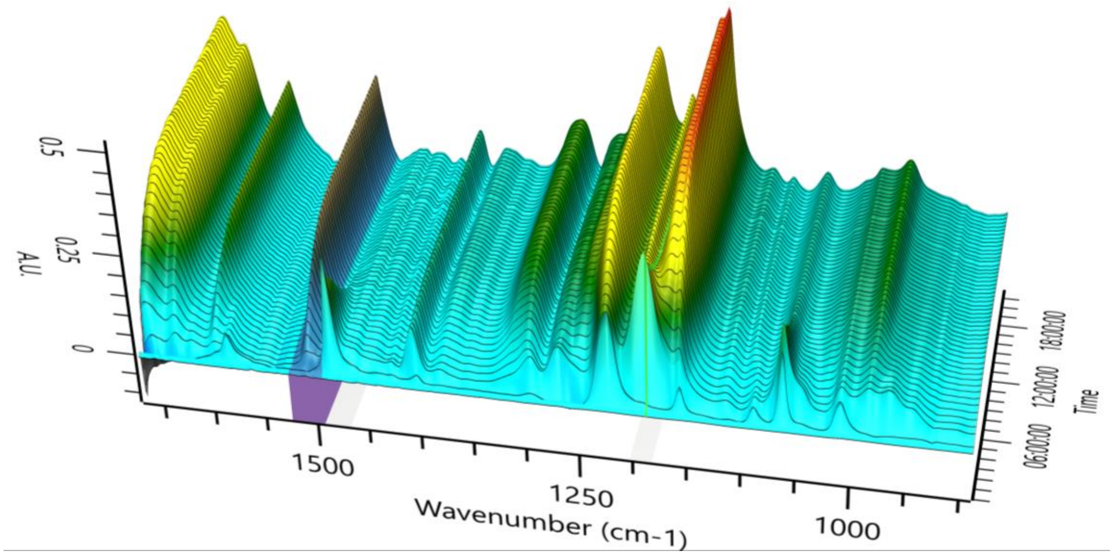

Figure 10: ***in situ*** **IR compilation surface generated by iC IR 7.1 software for the Heck reaction (lab book ref. DRH-01-144)**

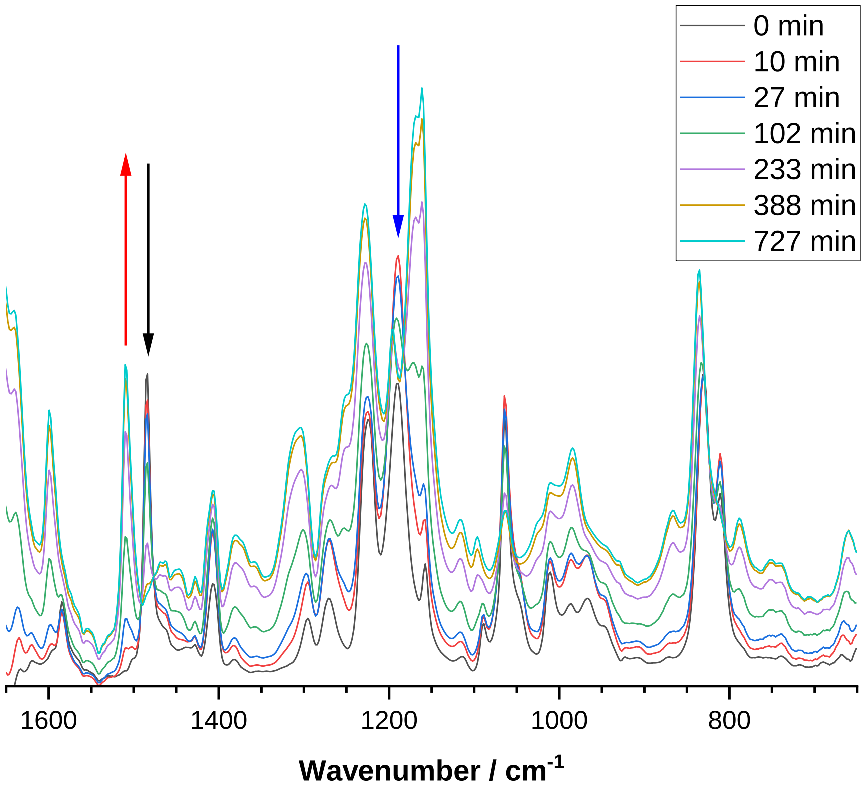

Figure 11: Selected spectra of the Heck reaction (lab book ref. DRH-01-144) over the course of the reaction. The reaction was sampled every 1 min. The direction of key peaks are indicated by arrows (blue for n-butyl acrylate SI41 (1190 cm^{-1}), black for 1-bromo-4-fluorobenzene 3 (1484 cm^{-1}) and red for n-butyl 4-fluorocinnamate SI42 (1509 cm^{-1})).

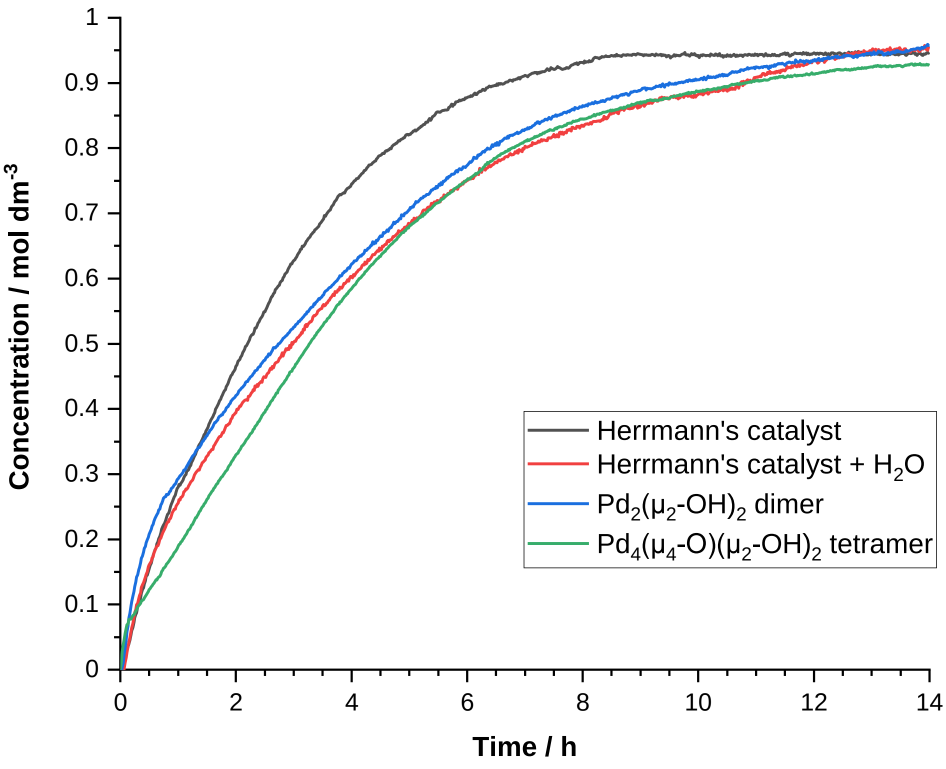

Figure 12: Comparison of Heck reactions (product SI42 formation) with different pre-catalysts (General Procedure 2.1)

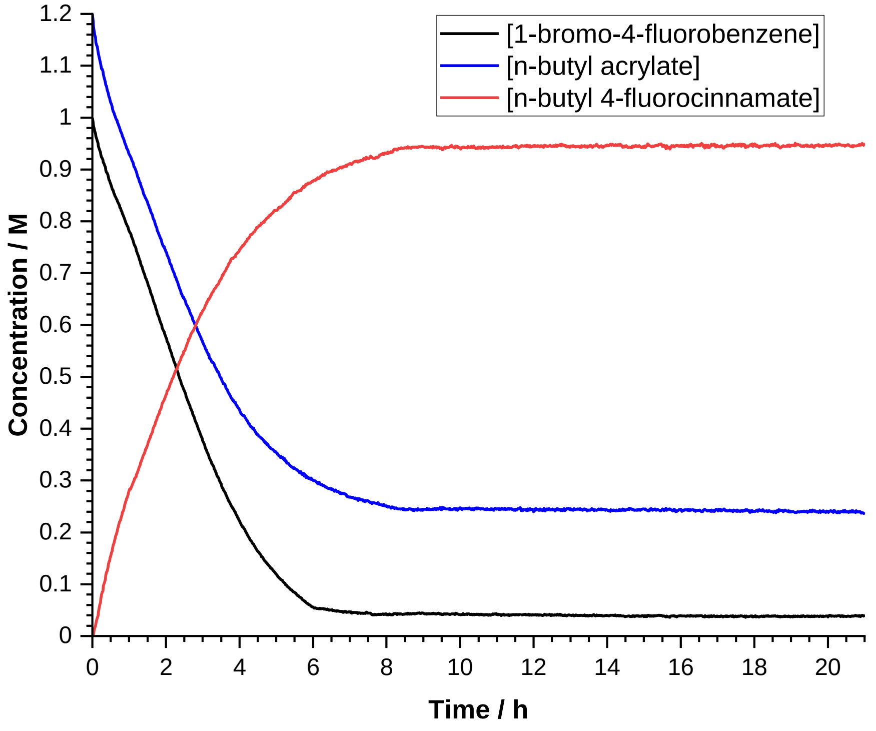

Figure 13: Heck reaction catalyzed with Herrmann's catalyst 1 (General Procedure 2.1). Lab book ref. DRH-01-144

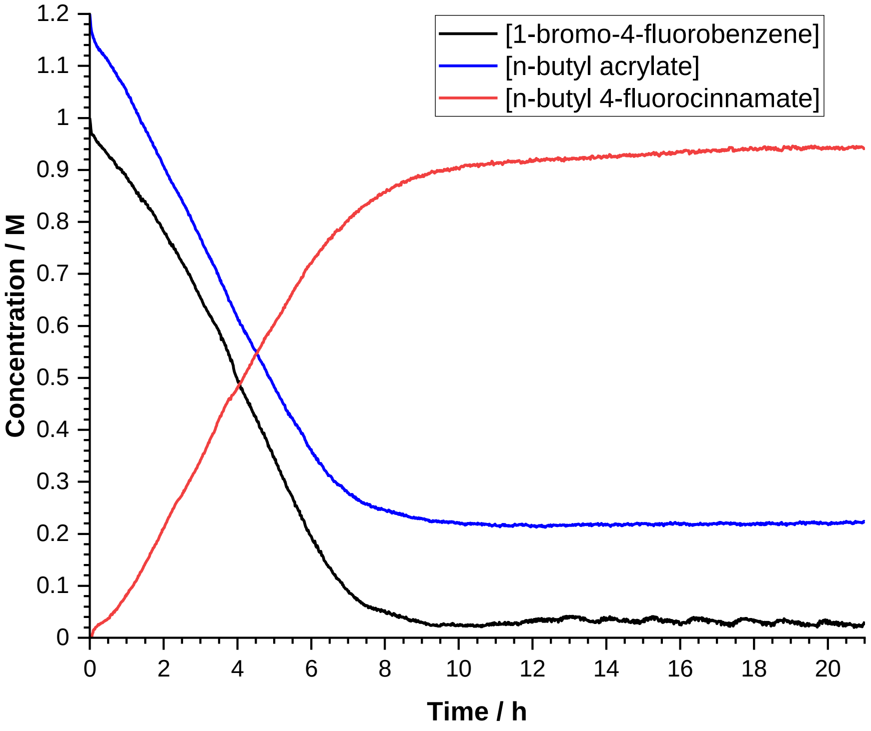

Figure 14: Heck reaction catalyzed by Herrmann's catalyst 1 with 50 μL water added (General Procedure 2.1). Lab book ref. DRH-01-145

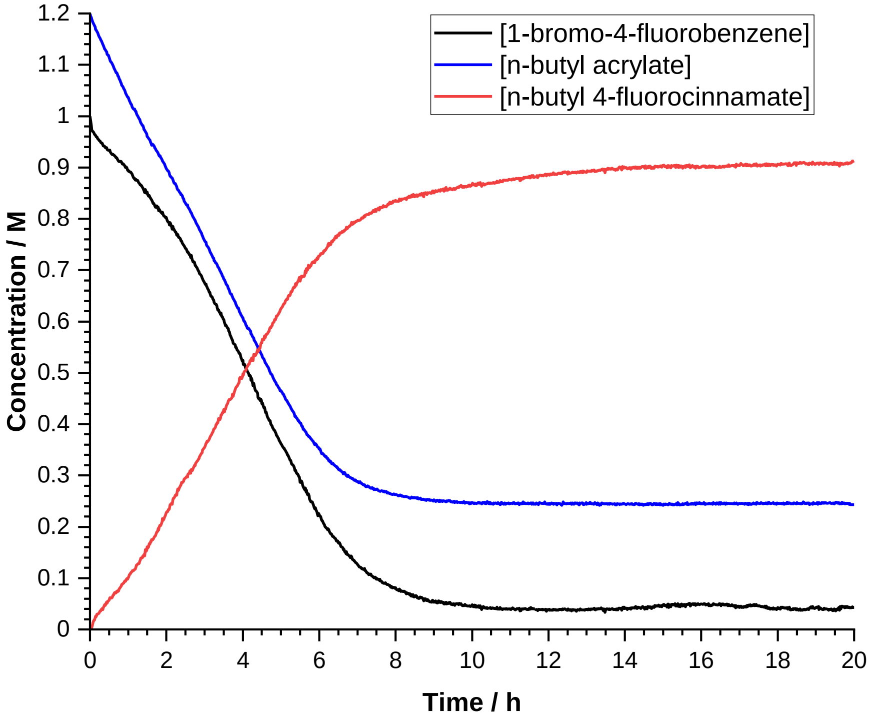

Figure 15: Heck reaction catalyzed by [Pd(P^C)(μ_2-OH)]$_2$ palladacycle 2 (General Procedure 2.1). Lab book ref. DRH-01-151

4.2 Effect of Catalyst Identity on SMCC

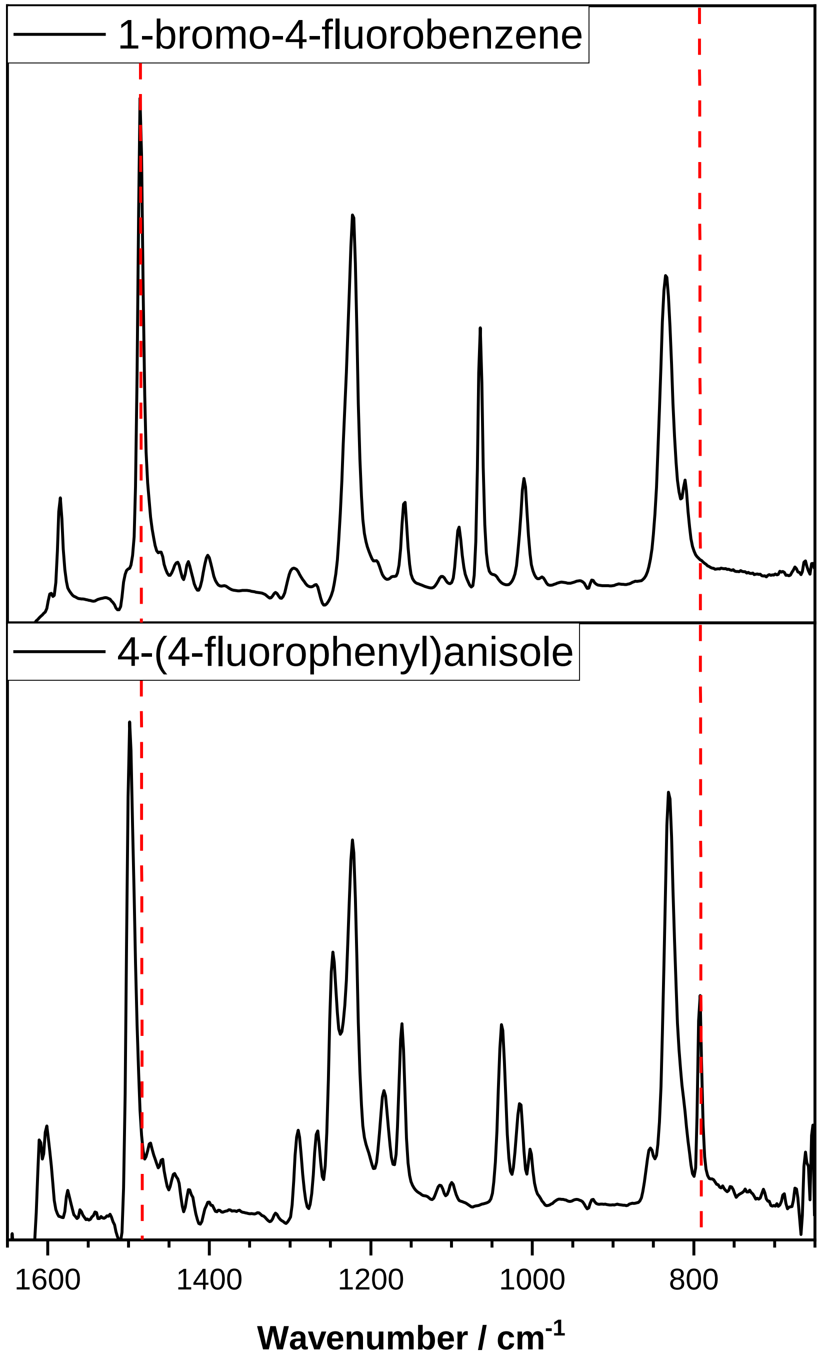

Figure 16: Reference spectra of (top) 1-bromo-4-fluorobenzene 3 and (bottom) 4-(fluorophenyl)anisole 5 product in NMP / H_2O (11:3). Spectra taken using ReactIR 15 (Diamond probe) at 23 °C. The key peaks that are followed by IR during the reaction are indicated by the dashed red lines (1486 cm^{-1} for 1-bromo-4-fluorobenzene 3 and 783 cm^{-1} for

4-(fluorophenyl)anisole 5. In a few cases, the 4-(fluorophenyl)anisole 5 peak at 1500 cm^{-1} was also used, but this tended to overlap with other peaks.

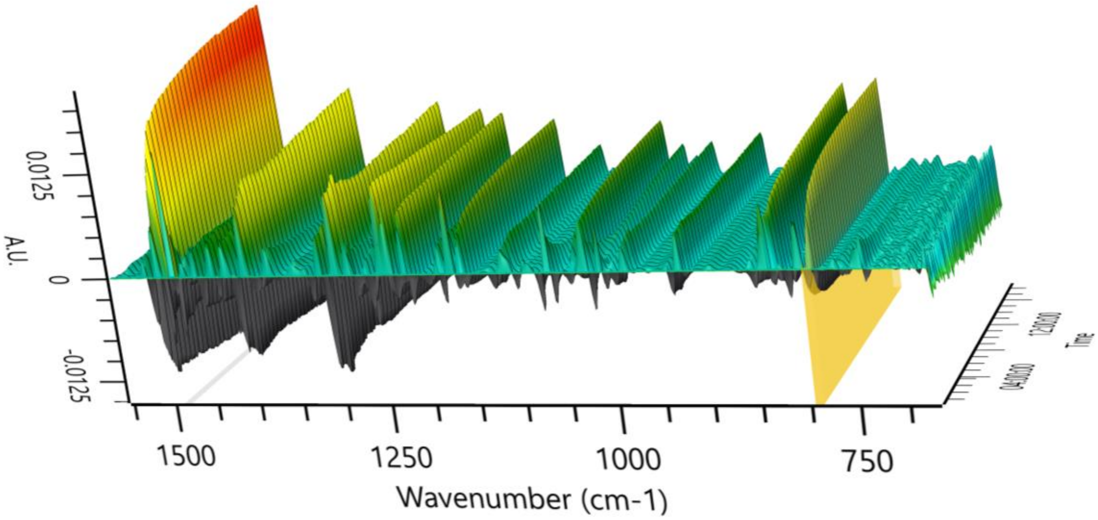

Figure 17: *in situ* IR compilation surface generated by iC IR 7.1 software for the SMCC reaction (lab book ref. DRH-03-111)

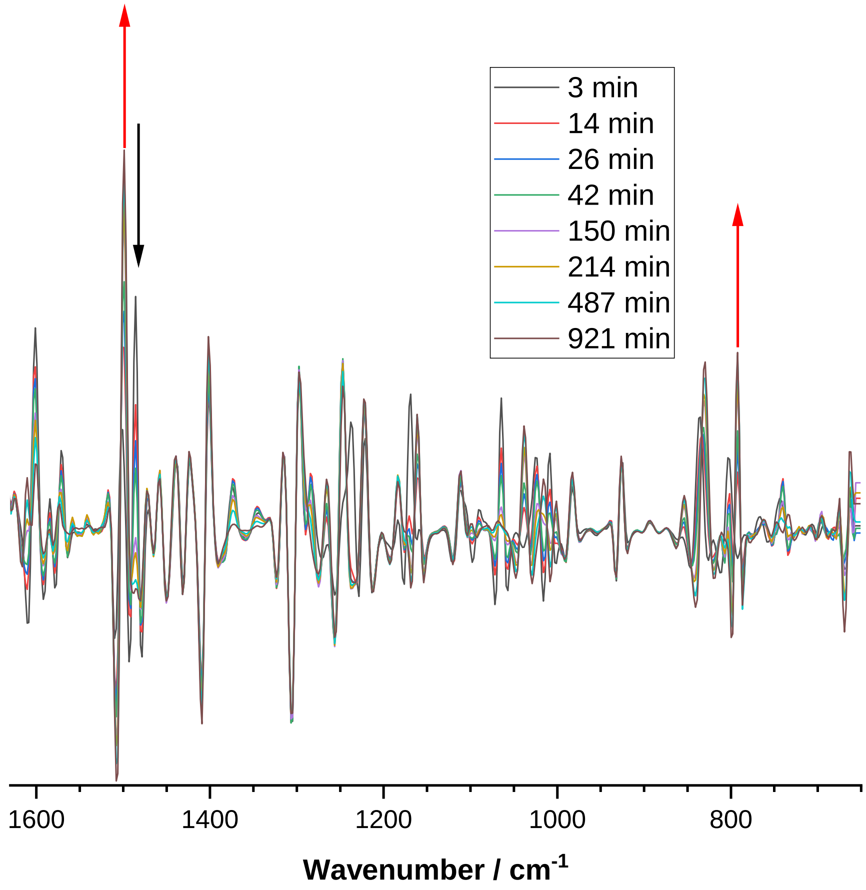

Figure 18: Selected spectra of the SMCC reaction (lab book ref. DRH-03-111) over the course of the reaction. The data has been processed using 2nd Derivative analysis to deconvolute peaks, with sampling every 1 min. The

direction of key peaks (see Figure 16) are indicated by arrows (black for 1-bromo-4-fluorobenzene 3, red for 4-(fluorophenyl)anisole 5).

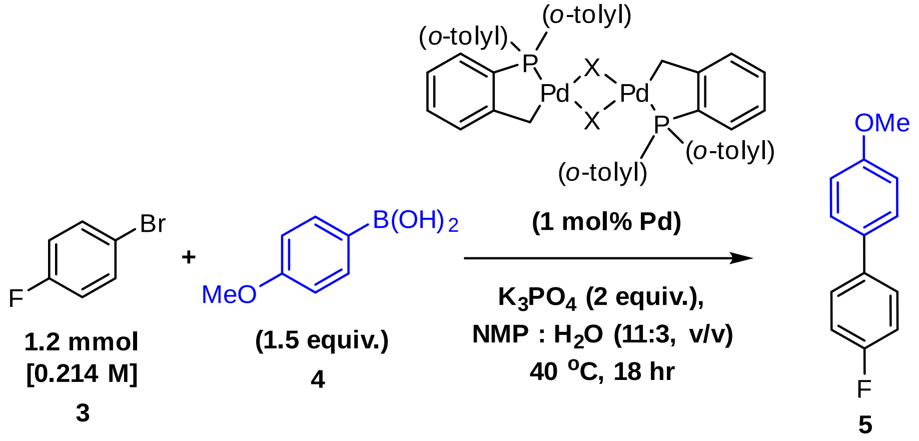

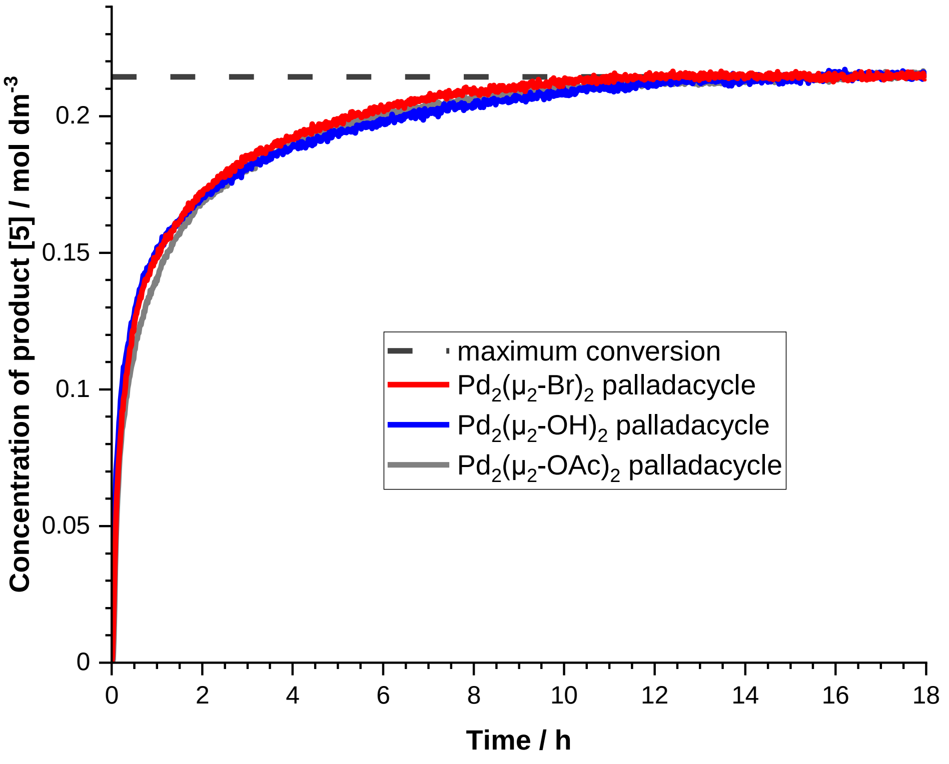

Figure 19: Compiled ReactIR data for the standard Suzuki reaction (product formation only) using $Pd_2(\mu_2\text{-}X)_2$ palladacycle pre-catalysts (X = Br (35), OH (2), OAc (1)).

Table 3: Fitted Hill equation parameters for kinetic data

Pd catalyst	V_{max} / mol dm^{-3}	k / h	n
$Pd_2(\mu_2\text{-}Br)_2$ palladacycle (**35**)	0.2311 ± 0.0003	0.455 ± 0.003	0.755 ± 0.005
$Pd_2(\mu_2\text{-}OH)_2$ palladacycle (**2**)	0.2385 ± 0.0004	0.423 ± 0.003	0.603 ± 0.004

$Pd_2(\mu_2\text{-}OAc)_2$ palladacycle (**1**)	0.2296 ± 0.0002	0.542 ± 0.002	0.790 ± 0.004
$Pd^{(0)}[P(o\text{-tolyl})_3]_2$	0.2214 ± 0.0005	0.762 ± 0.007	1.05 ± 0.01

Comparison of $[Pd(P\hat{}C)(\mu_2\text{-}OH)]_2$ palladacycle 2 with $Pd^{(0)}$ source

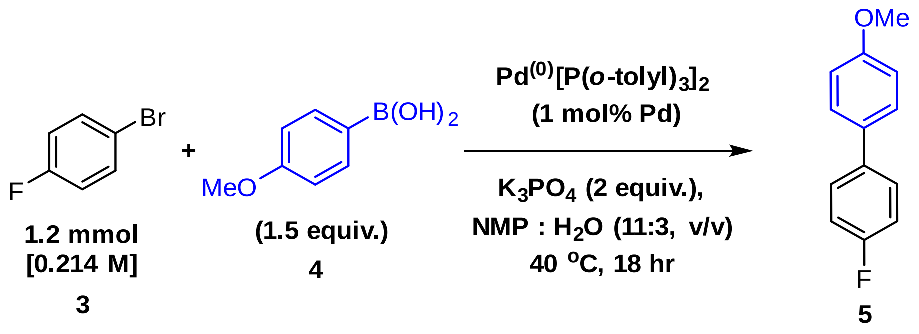

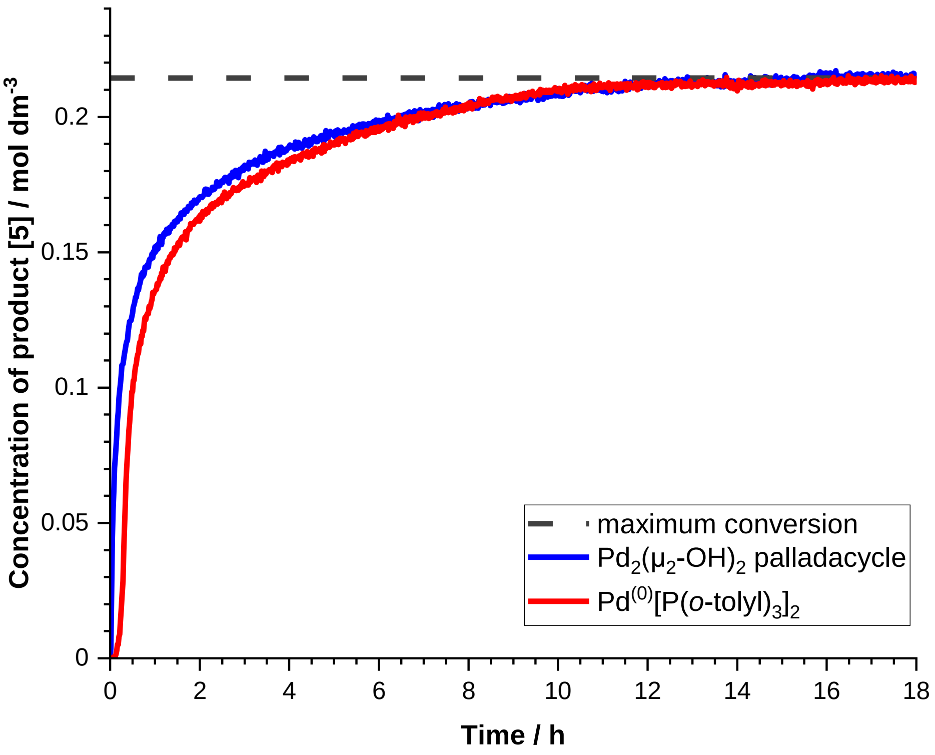

Figure 20: Compiled ReactIR data for the standard Suzuki reaction (General Procedure 2.2 product 5 formation only) using $[Pd(P\hat{}C)(\mu_2\text{-}OH)]_2$ palladacycle 2 and $Pd^{(0)}[P(o\text{-tolyl})_3]_2$ as pre-catalysts.

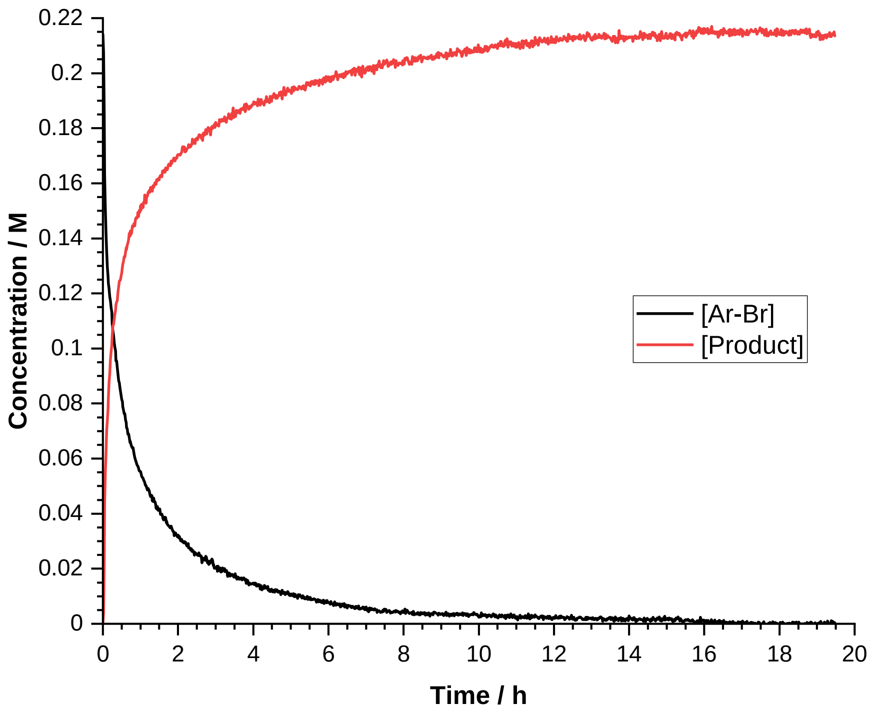

Figure 21: [Pd(P^C)(μ_2-OH)]$_2$ palladacycle 2 under standard Suzuki-Miyaura conditions (General Procedure 2.2). Lab book ref. DRH-02-91

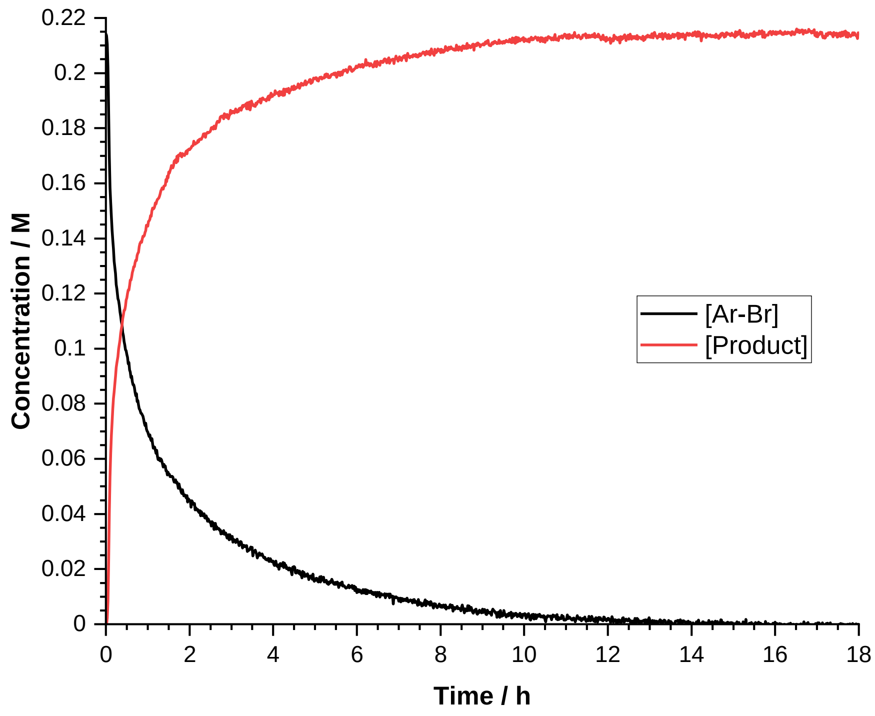

Figure 22: Herrmann's catalyst 1 under standard Suzuki-Miyaura conditions (General Procedure 2.2). Lab book ref. DRH-03-111

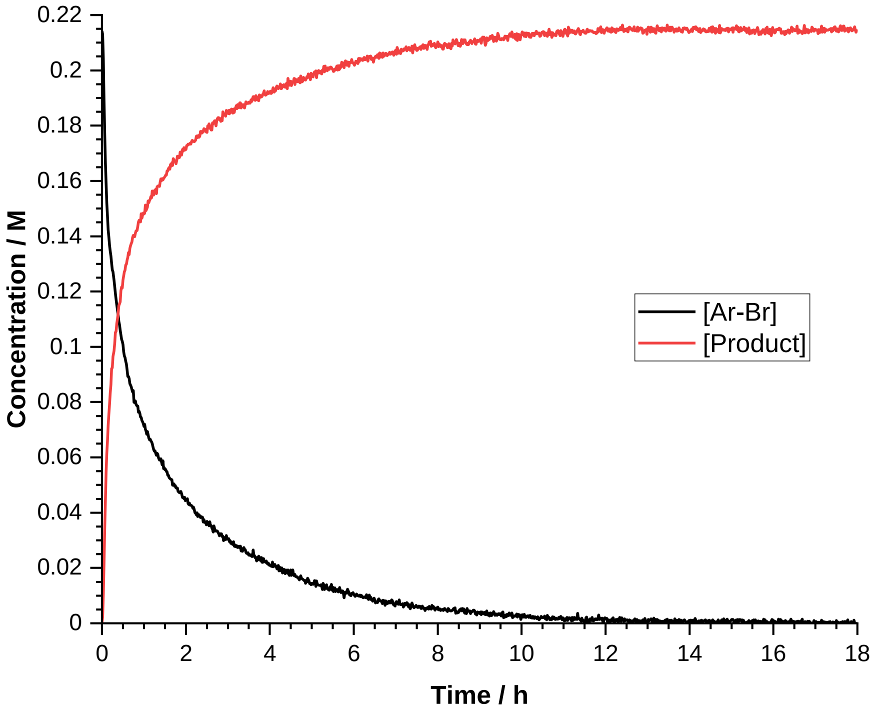

Figure 23: [Pd(P^C)(μ_2-Br)]$_2$ palladacycle 35 under standard Suzuki-Miyaura conditions (General Procedure 2.2). Lab book ref. DRH-02-115

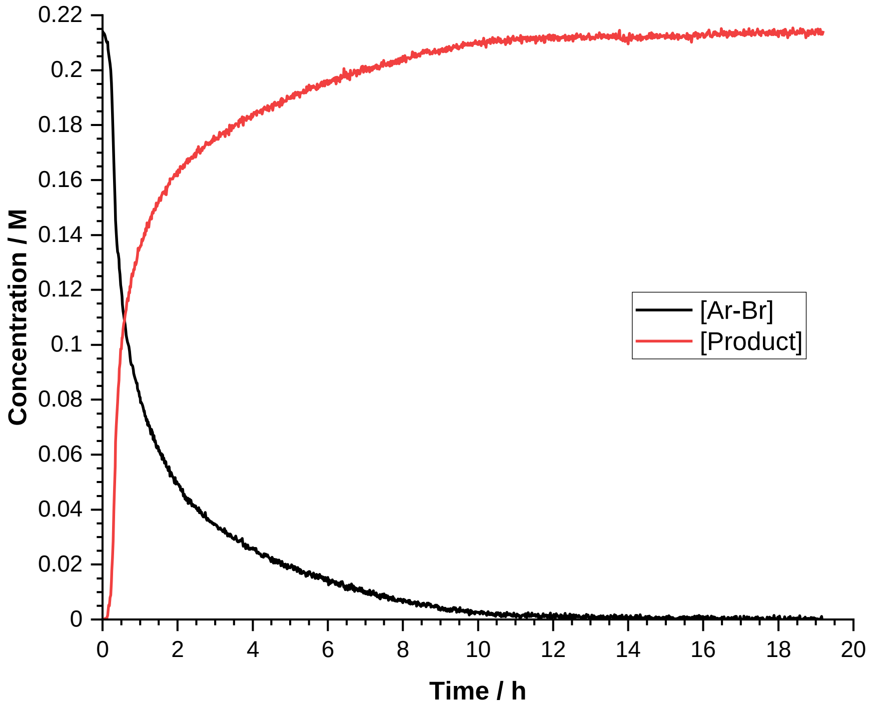

Figure 24: Pd$^{(0)}$[P(*o*-tolyl)$_3$]$_2$ catalyst under standard Suzuki-Miyaura conditions (General Procedure 2.2). Lab book ref. DRH-02-96

4.3 Recharge Experiment

To verify if the Pd species present at the end of a reaction are catalytically competent, a recharge experiment was undertaken. This involved the addition of an extra 50% of each reagent (4-fluorobromobenzene, 4-methoxyphenyl boronic acid, K_3PO_4) relative to the starting quantities for (General Procedure 2.2, $[Pd(P^\wedge C)(\mu_2\text{-}Br)]_2$ palladacycle **35**) to the reaction after 3 hours, with *in situ* IR monitoring. After a further 16 hours, a ^{19}F NMR sample was taken to calculate conversion (100%).

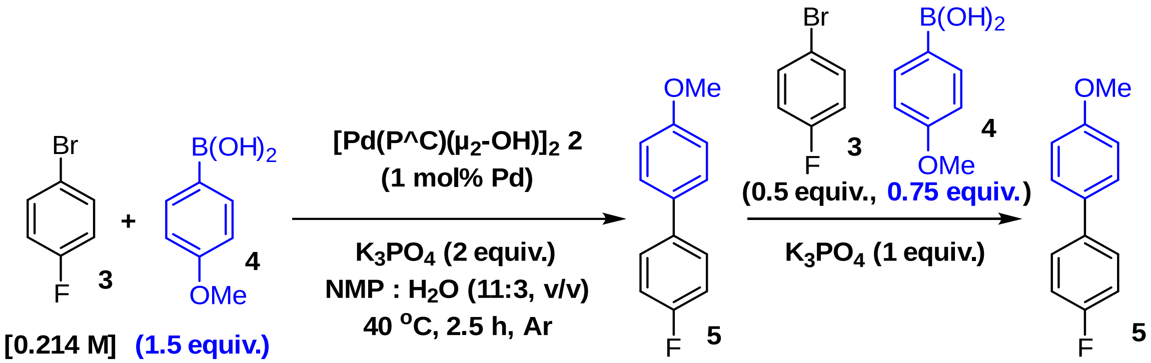

Scheme 2: Recharge experiment demonstrating the catalyst activity towards the end of a reaction by addition of more reagents.

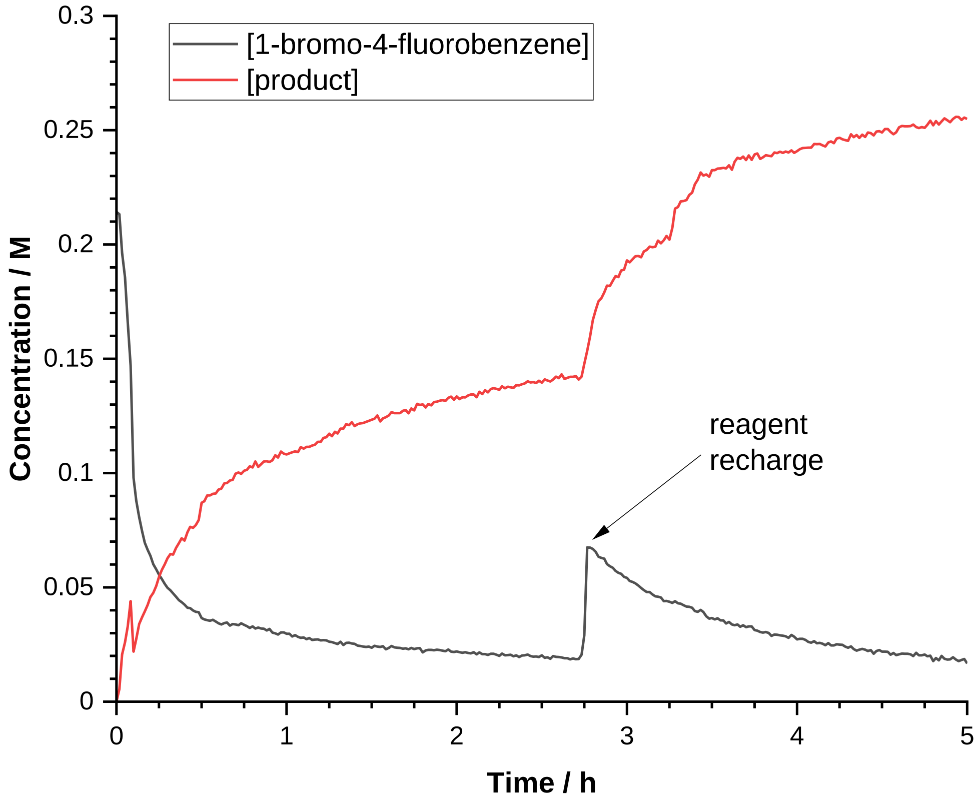

Figure 25: ReactIR trace for the recharge experiment (Scheme 2). At higher product concentrations, a small amount of probe fouling and peak drift occurred, so only the relevant recharge region is shown. Lab book ref. DRH-02-171

Although there is a small amount of probe fouling from residues in the concentrated reaction, it is clear that the reaction proceeds with a recharge (albeit at a slower rate), indicating that the Pd species present at the end of the reaction are still catalytically competent.

4.4 Effect of Pinacol Additive on Kinetics

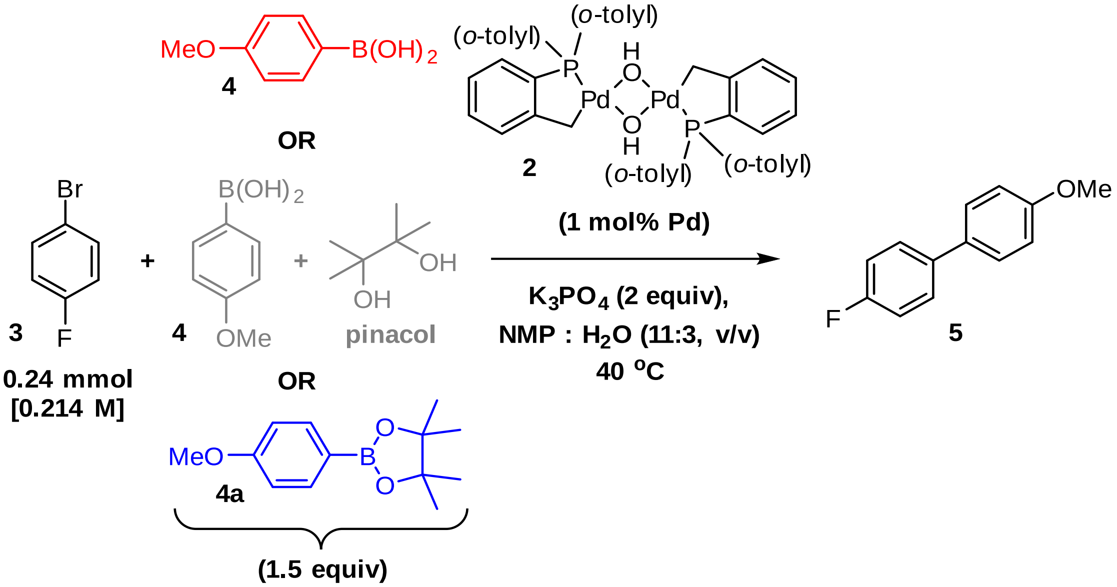

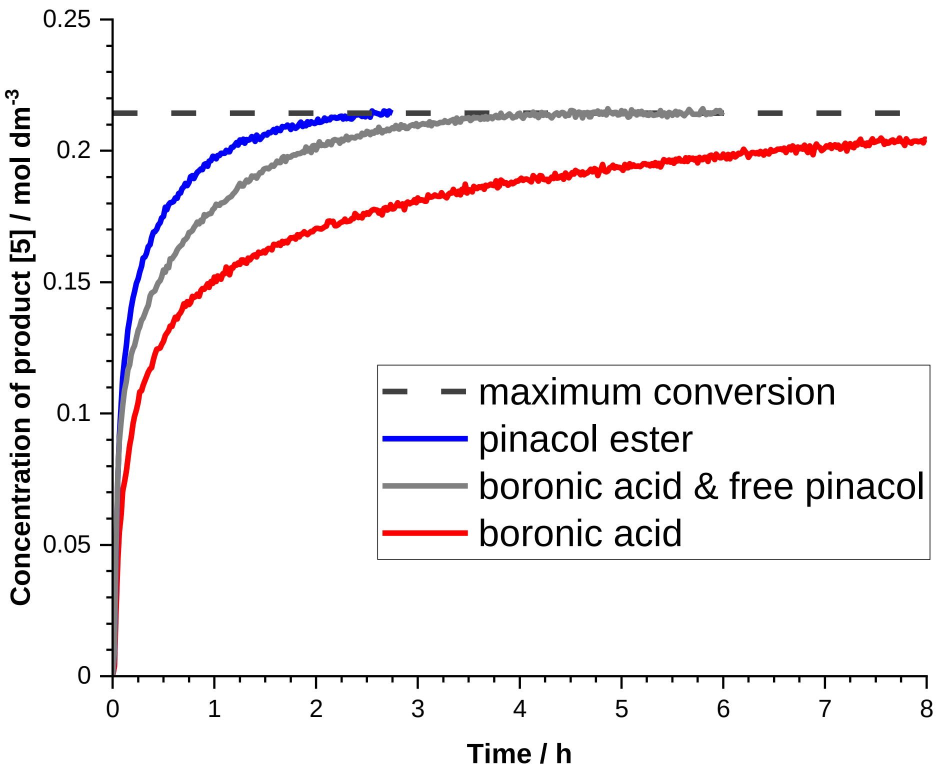

Figure 26: Compiled kinetic profiles of the standard SMCC (General Procedure 2.2) with changing the boron coupling partner or adding a pinacol additive

Table 4: Fitted Hill equation parameters for kinetic data when the boron coupling partner is changed

Boron coupling partner	V_{max} / mol dm^{-3}	k / h	n
No additive (arylboronic acid **4)**	0.2385 ± 0.0004	0.423 ± 0.003	0.603 ± 0.004
Pinacol additive (**4** + pinacol)	0.2186 ± 0.0003	0.160 ± 0.002	0.93 ± 0.01
Arylboronic pinacol ester **4a**	0.228 ± 0.001	0.118 ± 0.002	0.88 ± 0.02

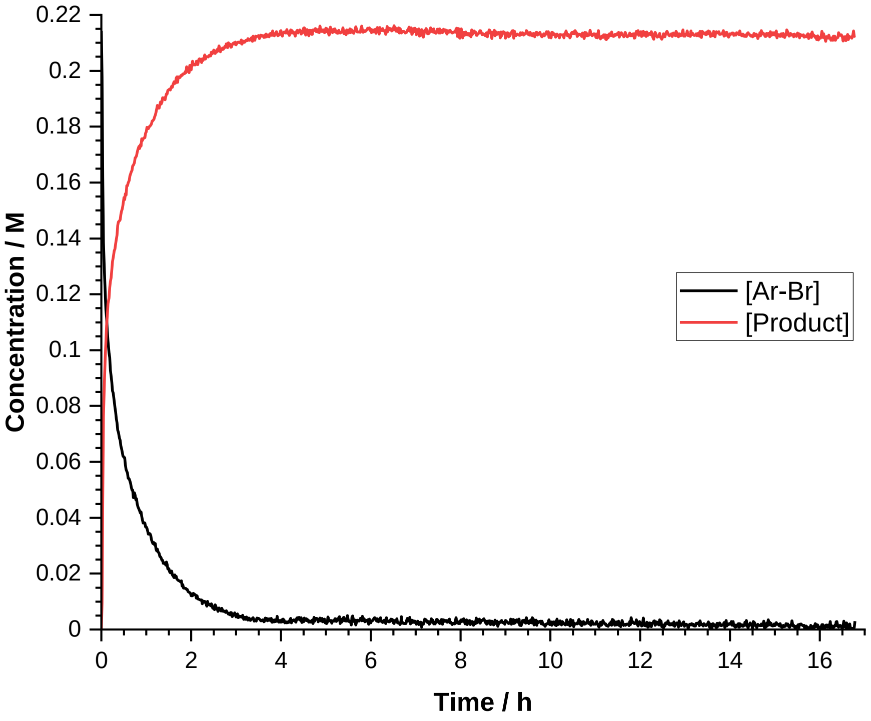

Figure 27: Suzuki-Miyaura cross-coupling reaction under standard conditions (General Procedure 2.2), with 1 equiv. (w.r.t. arylboronic acid) pinacol additive. Lab book ref. DRH-02-126

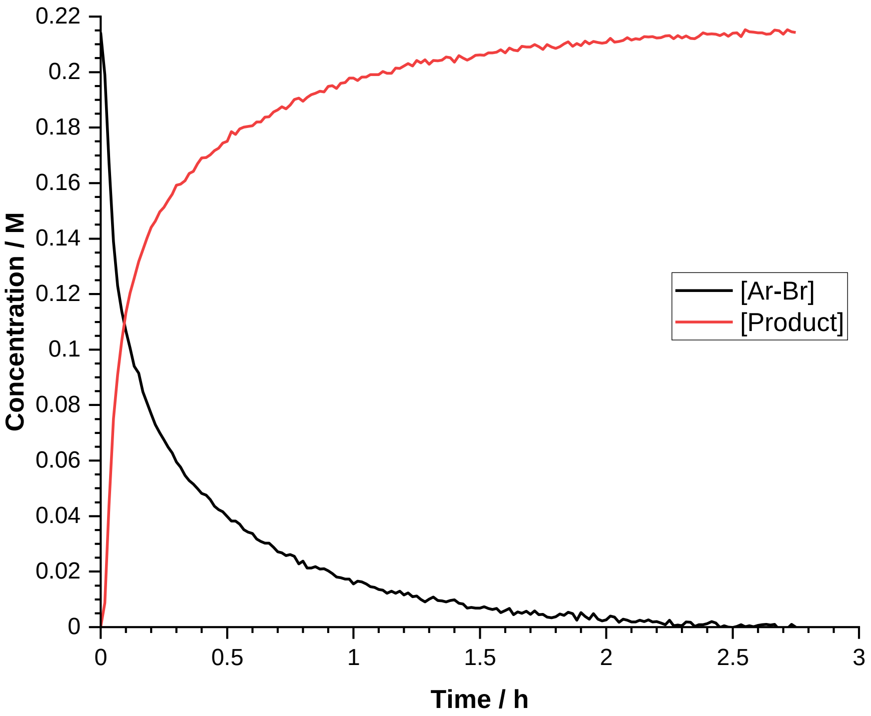

Figure 28: Suzuki-Miyaura cross-coupling reaction under standard conditions (General Procedure 2.2), with arylboronic pinacol ester 4a being used instead of arylboronic acid. Lab book ref. DRH-02-121-2

4.5 Use of PdNPs as Catalysts

DMF-stabilized palladium nanoparticles (DMF-PdNPs) were synthesized according to a literature proceedure.[10] An amount corresponding to 1 mol% Pd loading for a Suzuki reaction (General Procedure 2.2) of freshly made DMF-PdNPs was isolated by solvent removal (*in vacuo*, with heating), redissolved in NMP (1 mL, dry, degassed), and added directly into a Suzuki reaction mixture for monitoring by *in situ* IR. For reactions utilizing a phosphine ligand, 1 mol% of the corresponding ligand was added to the reaction prior to PdNP addition. The kinetic data are presented in Figure 7 within the manuscript.

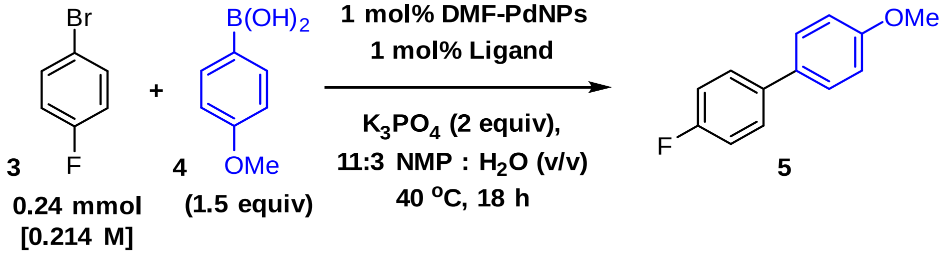

Table 5: Fitted linear equation (y = mx + c) parameters for kinetic data of SMCC catalyzed by PdNPs. Errors displayed are the Standard errors on the fitting.

Catalyst	Rate / M h^{-1}	Intercept / M	Adjusted R-squared
PdNPs + P(*o*-tolyl)$_3$	0.00238 ± 4×10^{-6}	0.00284 ± 0.00004	0.99701
PdNPs	0.00105 ± 6×10^{-6}	0.00117 ± 0.00006	0.96798
PdNPs + P(*p*-tolyl)$_3$	0.00087 ± 6×10^{-6}	0.00030 ± 0.00005	0.95923

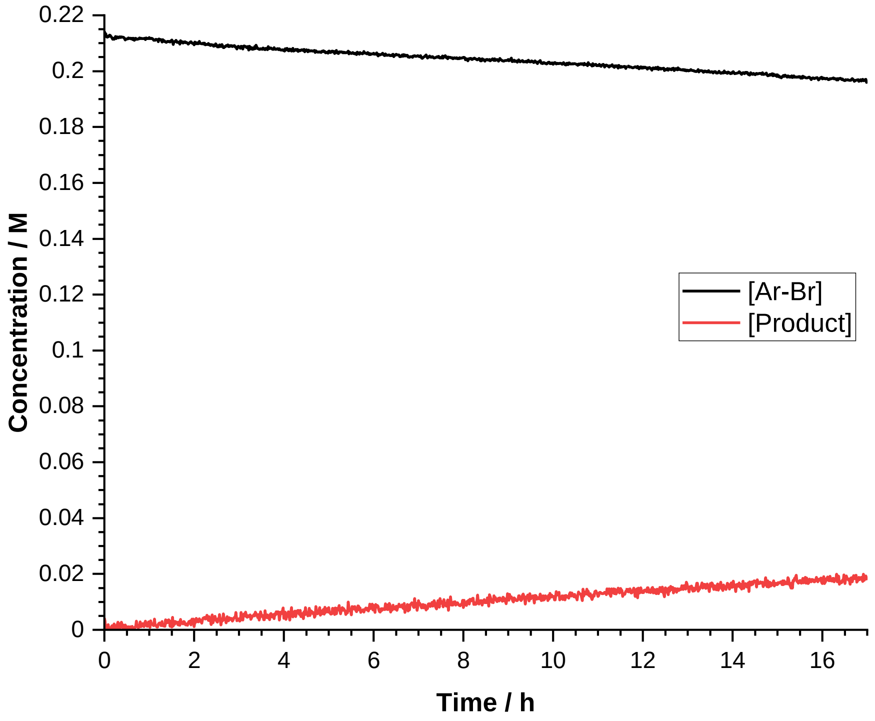

Figure 29: Suzuki-Miyaura cross-coupling reaction under standard conditions (General Procedure 2.2), using 1 mol% PdNPs. Lab book ref. DRH-03-80

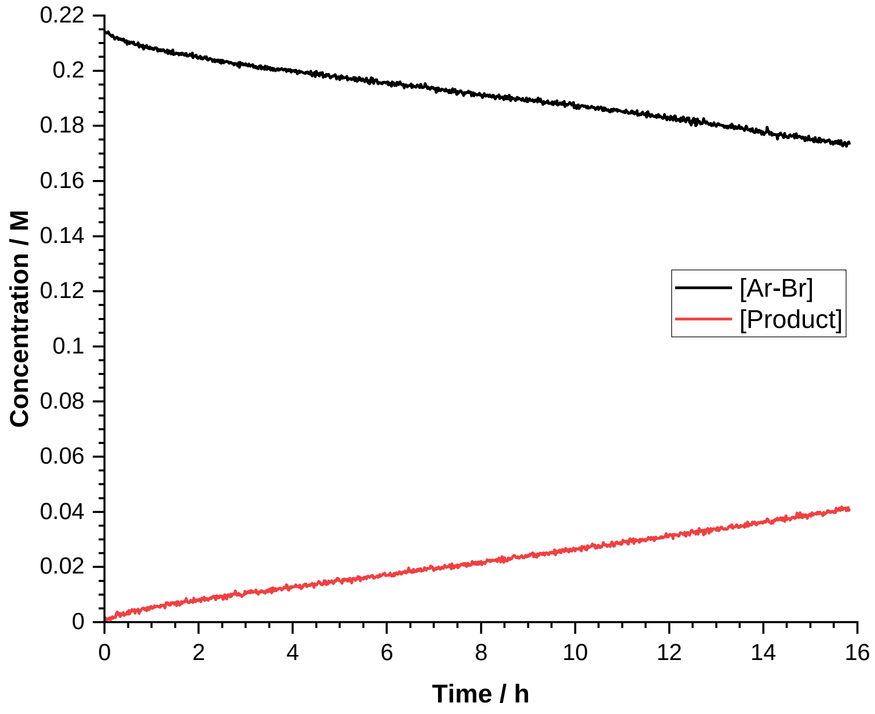

Figure 30: Suzuki-Miyaura cross-coupling reaction under standard conditions (General Procedure 2.2), using 1 mol% PdNPs with 1 mol% P(*o*-tolyl)$_3$. Lab book ref. DRH-03-81.

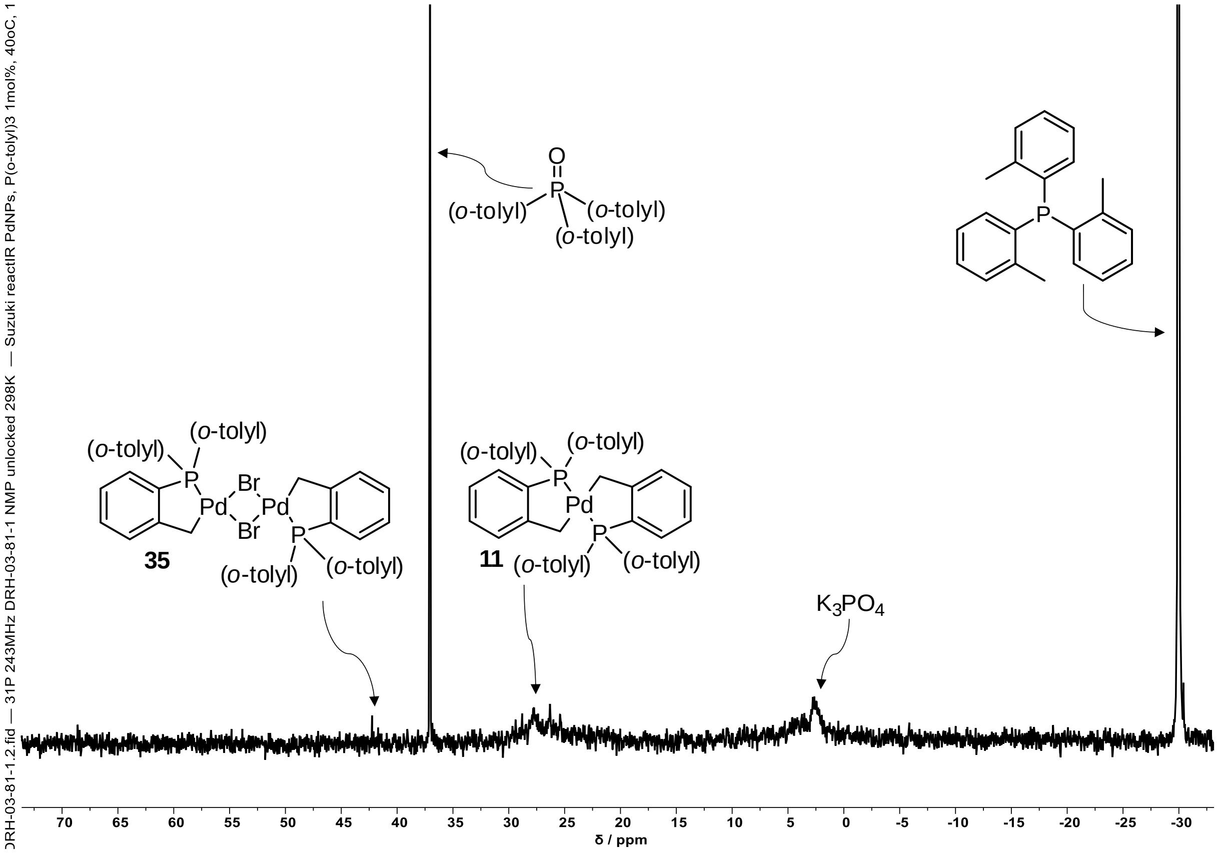

Figure 31: High scan ^{31}P NMR (243 MHz, NMP unlocked, 57344 scans, 298 K) of reaction mixture from Figure 30, P(*o*-tolyl)$_3$ ligand) confirming that homogeneous Pd complexes are present. Lab book ref. DRH-03-81-1.

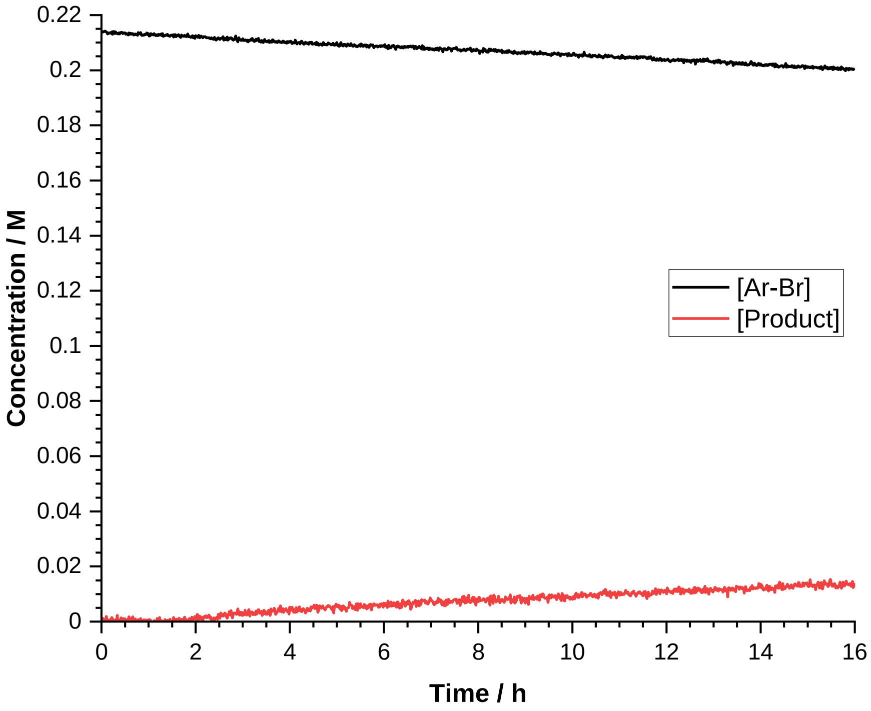

Figure 32: Suzuki-Miyaura cross-coupling reaction under standard conditions (General Procedure 2.2), using 1 mol% PdNPs with 1 mol% P(*p*-tolyl)$_3$. Lab book ref. DRH-03-85

4.6 Catalyst Concentration effect on kinetics

All reactions were carried out according to General Procedure 2.2, with varying concentrations of reagents. For varying the catalyst loading, General Procedure 2.2 at half concentration and 1.2 equiv. of boronic acid **4** was employed. [Ar-Br] refers to the concentration of aryl bromide **3** (4-fluorobromobenzene), [product] refers to the concentration of product **5** (4-(4-fluorophenyl)anisole), and [boronic acid] refers to the concentration of arylboronic acid **4** (4-methoxyphenylboronic acid).

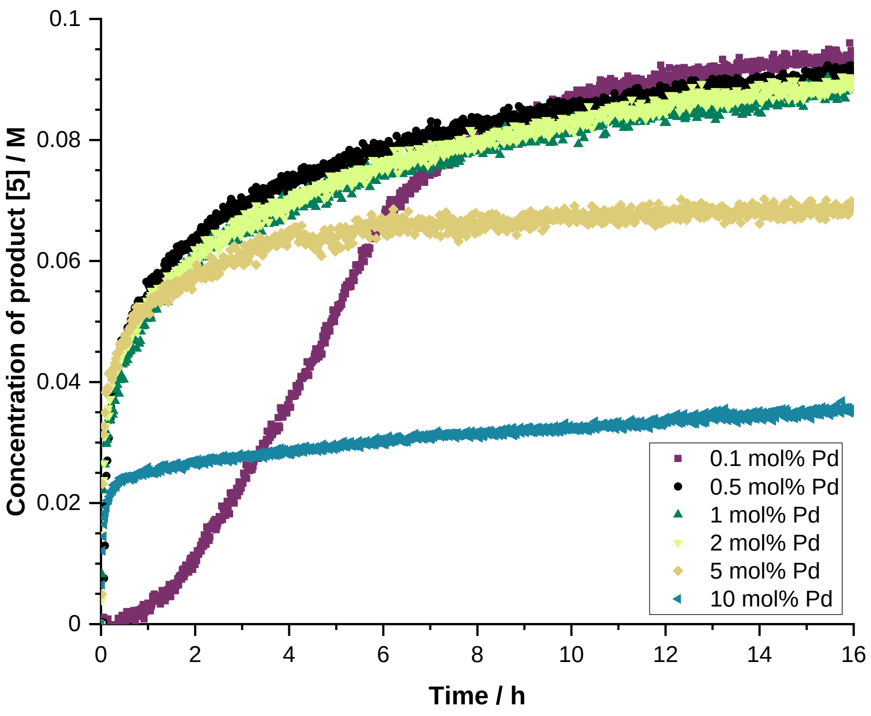

Figure 33: Compilation graph of product 5 formation with different catalyst loadings monitored by *in situ* IR. Lab book refs. DRH-02-80-2 (0.1 mol% Pd, 0.00011 M), DRH-02-64 (0.5 mol% Pd, 0.00054 M), DRH-02-61-3 (1 mol% Pd, 0.0011 M), DRH-02-63-2 (2 mol% Pd, 0.0021 M), DRH-02-65 (5 mol% Pd, 0.0054 M), DRH-02-67 (10 mol% Pd, 0.011 M)

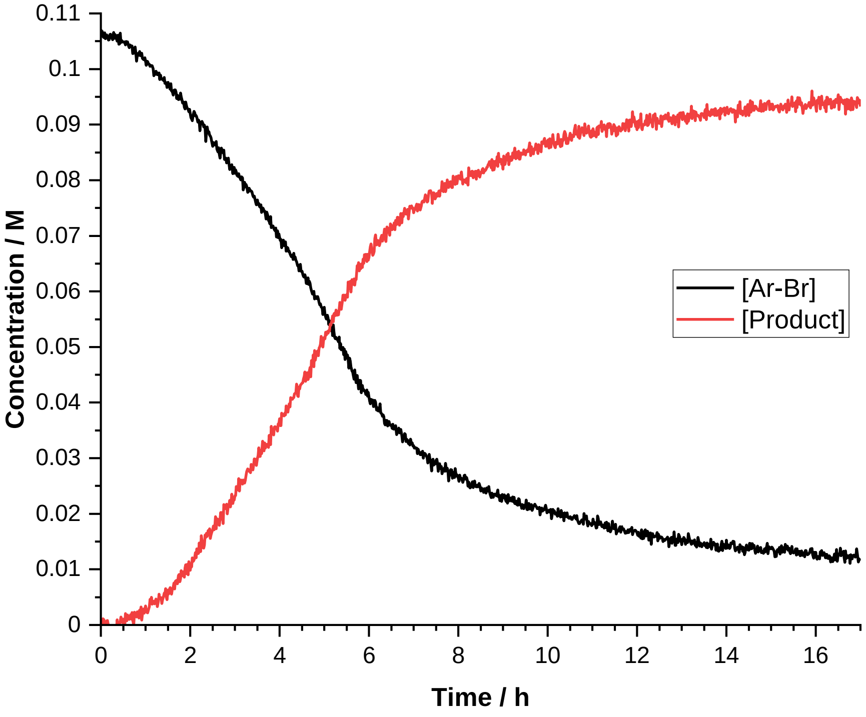

Figure 34: [Pd] = 0.1 mol% for Suzuki-Miyaura initial rates experiment. Lab book ref. DRH-02-80-2

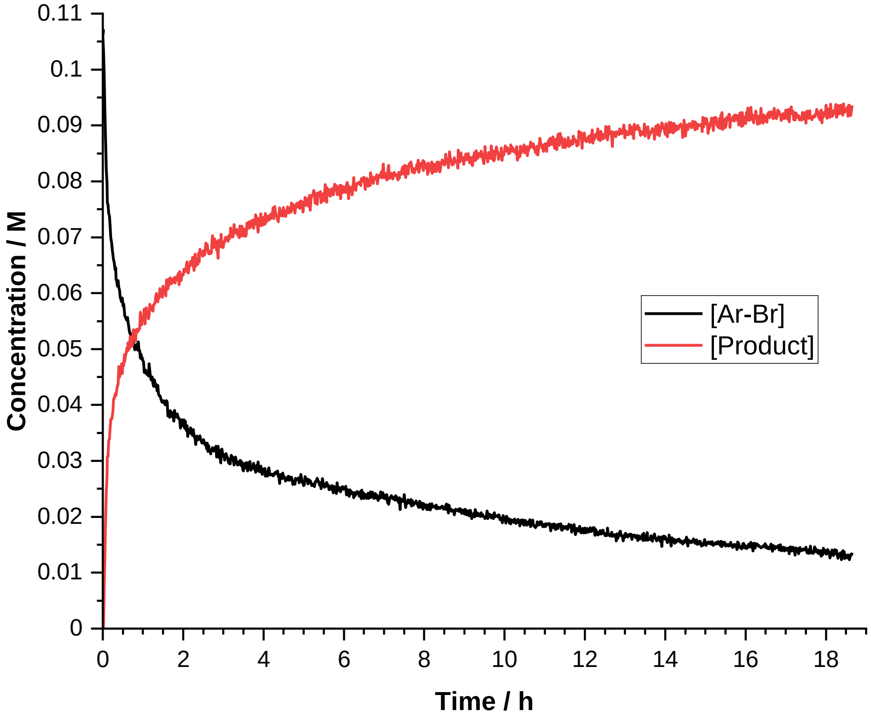

Figure 35: [Pd] = 0.5 mol% for Suzuki-Miyara initial rates experiment. Lab book ref. DRH-02-64

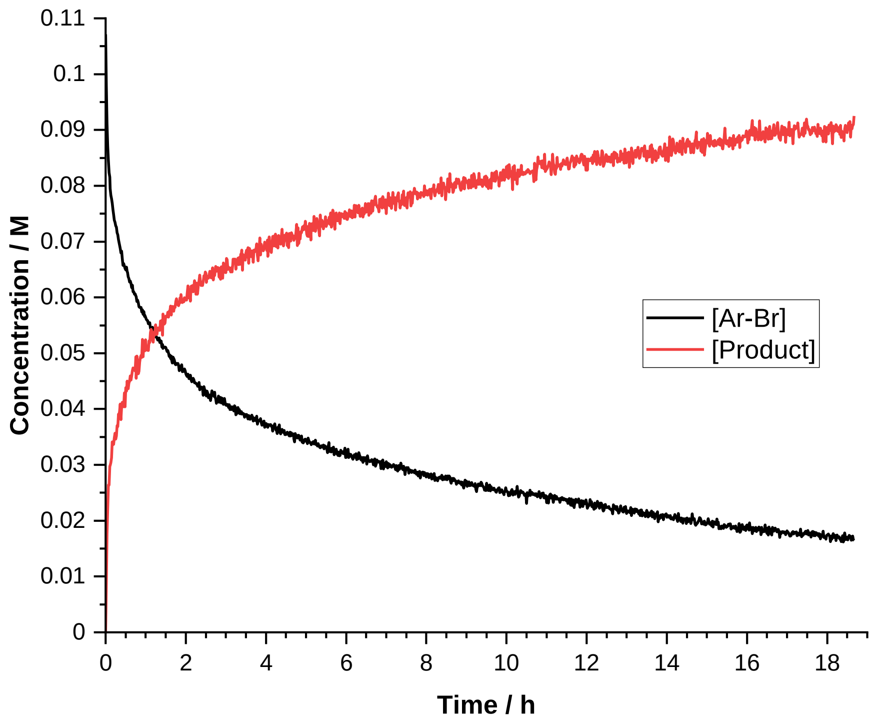

Figure 36: [Pd] = 1 mol% for Suzuki-Miyaura initial rates experiment. Lab book ref. DRH-02-61-3

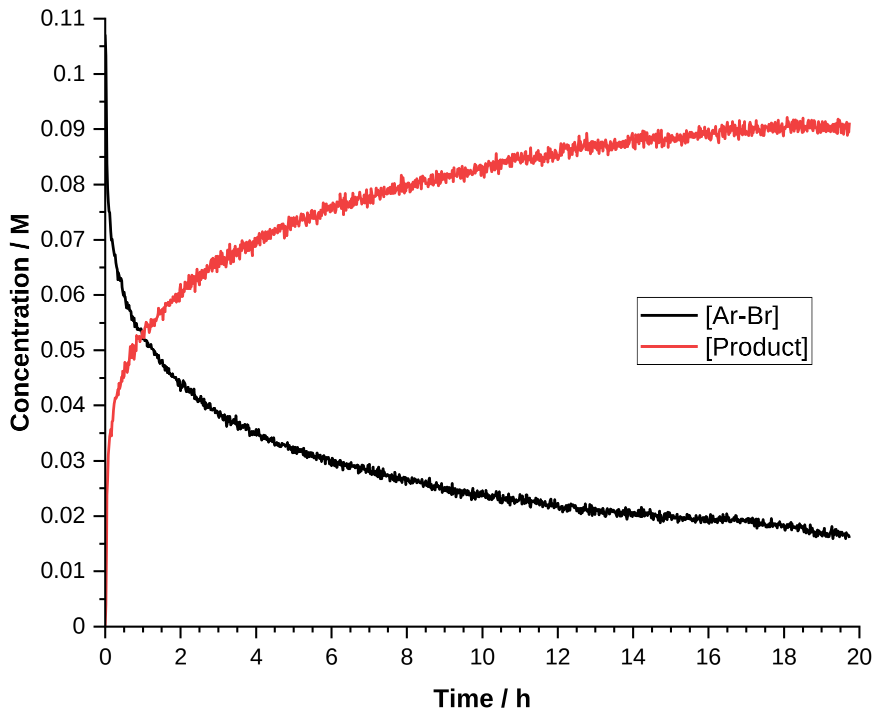

Figure 37: [Pd] = 2 mol% for Suzuki-Miyaura initial rates experiment. Lab book ref. DRH-02-63-2

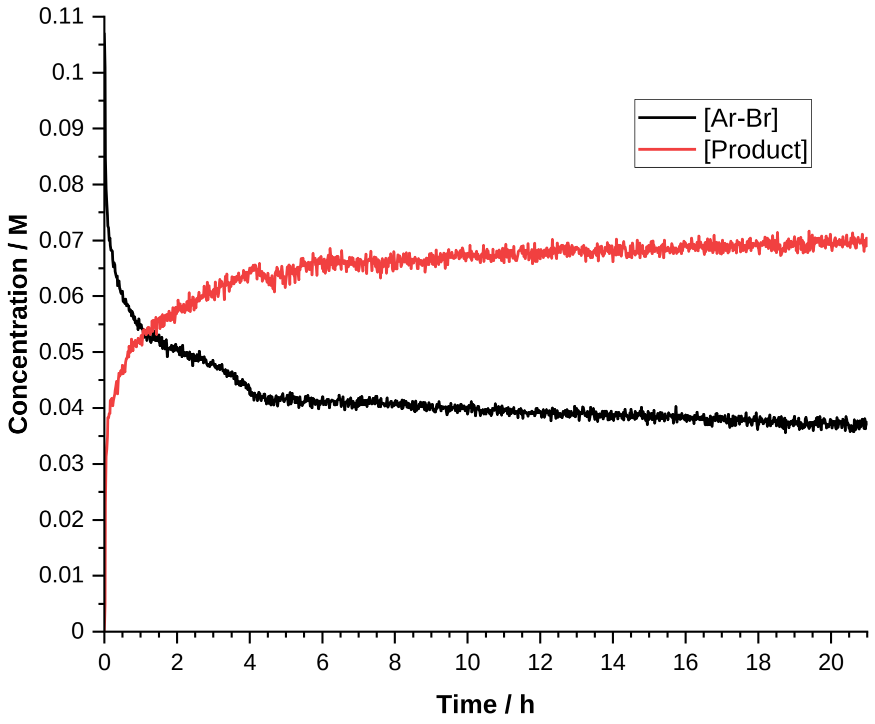

Figure 38: [Pd] = 5 mol% for Suzuki-Miyaura initial rates experiment. Lab book ref. DRH-02-65

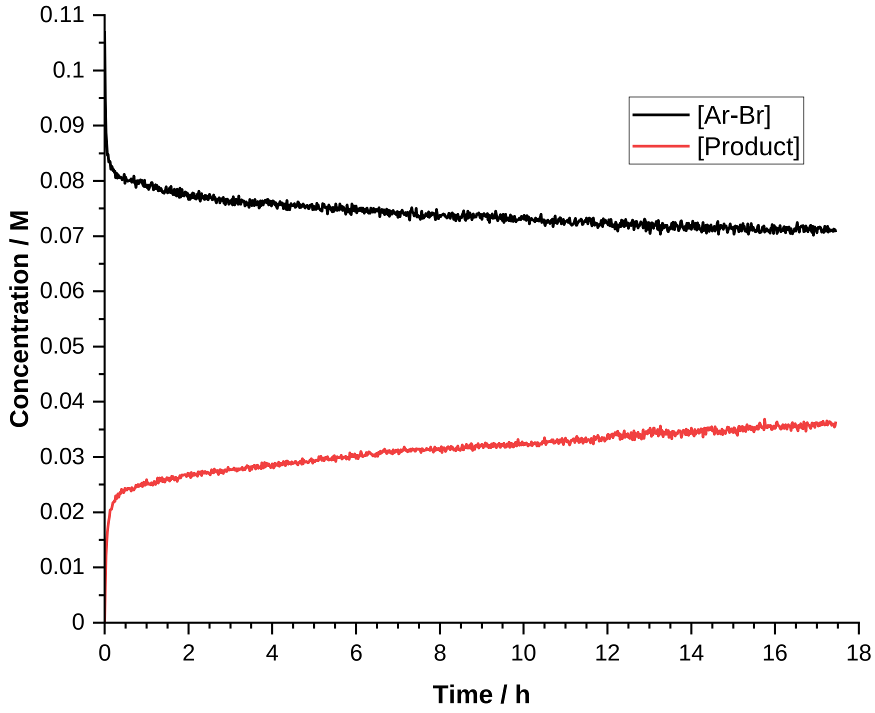

Figure 39: [Pd] = 10 mol% for Suzuki-Miyaura initial rates experiment. Lab book ref. DRH-02-67

4.7 Mercury Drop Test for PdNPs under SMCC Conditions

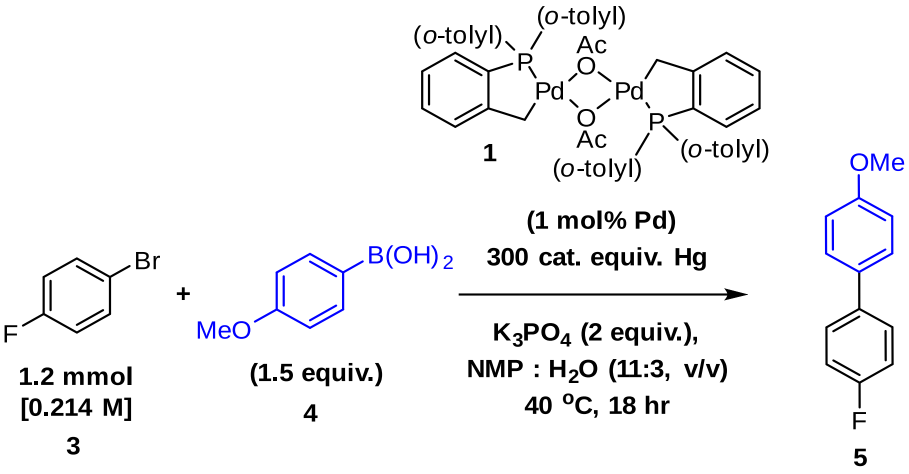

To investigate the suspected role of PdNPs in the standard Suzuki-Miyaura cross-coupling reaction, a mercury (Hg) drop test was performed. The presence of mercury should consume any PdNPs generated during a reaction and halt catalysis. If no PdNPs are present, the reaction should proceed as normal. As the Diamond probe ReactIR has a gold seal, it was impractical to do a Hg drop test using this probe for reaction monitoring, so an older Silicon probe was used instead. This has a significantly lower S/N, so the data is noisier than usual, and due to the shape, probe fouling by residues generated *in situ* was an issue. The same peaks were followed as for the standard reaction.

The first experiment used the General Procedure 2.2 conditions (using pre-catalyst **1**), with 300 catalyst equivalents of Hg added before the catalyst. Unfortunately, probe fouling occurred, and it was not possible to follow the reaction progress by IR. However, ^{19}F NMR showed that the reaction had reached 75% conversion after 18 h (Lab book ref. DRH-03-112-2). A follow up experiment involved running a Standard conditions reaction and introducing 300 catalyst equivalents of Hg after 19 min (approx. 50% conversion by IR of aryl halide consumption). This resulted in the reaction slowly stopping, reaching 75% conversion (^{19}F NMR) after 18 h. This is indicative of PdNPs being formed later in the reaction and being consumed by the Hg drop.

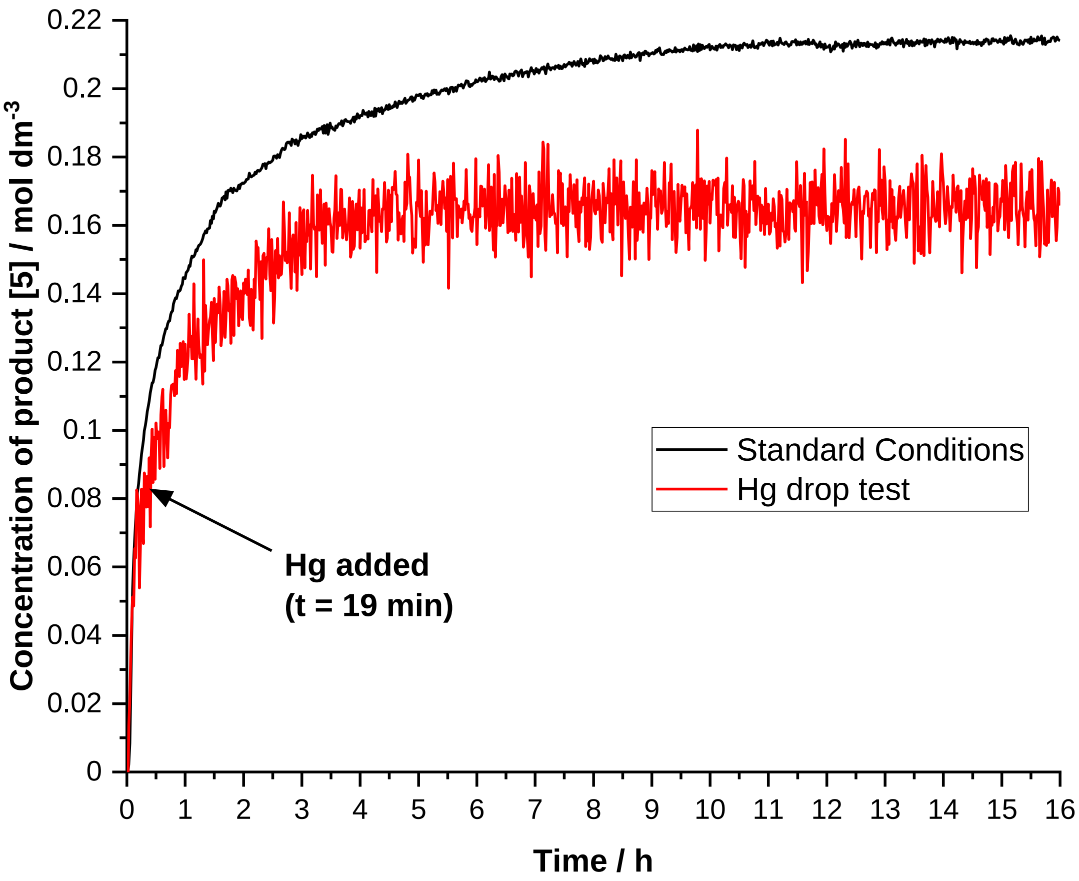

Figure 40: Mercury drop test under conditions from General Procedure 2.2 (Product 5 formation) Hg added at t = 19 min. Lab book ref. DRH-03-112, "Standard conditions" DRH-03-111

A third Hg drop test was performed using pinacol ester **4a** instead of boronic acid **4**. Standard conditions were used, again with the Silicon probe ReactIR. Hg was added at t = 9 min (approx. 50% conversion by IR of aryl halide consumption). As this reaction was expected to be faster than previous Hg drop tests, and as the S/N ratio for the product peak was particularly poor over these timescales, NMR aliquots were taken to track the conversion of **3** to product **5** using ^{19}F NMR. These datapoints are plotted on the graph below. NMR aliquots were taken by removing ~0.1 mL of reaction mixture using a N_2 purged needle and quenching the reaction with ~0.5 mL 1 M aqueous HCl solution. The mixture was then extracted using ~0.5 mL of C*D*Cl_3 for NMR analysis.

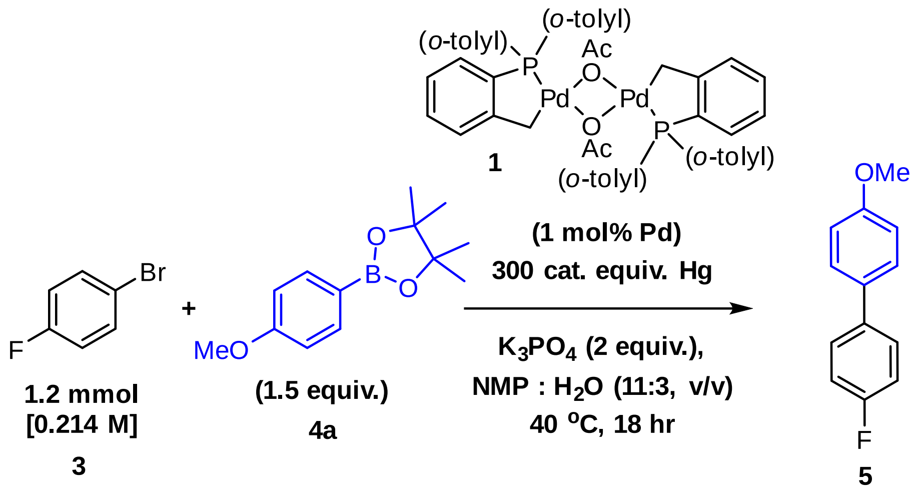

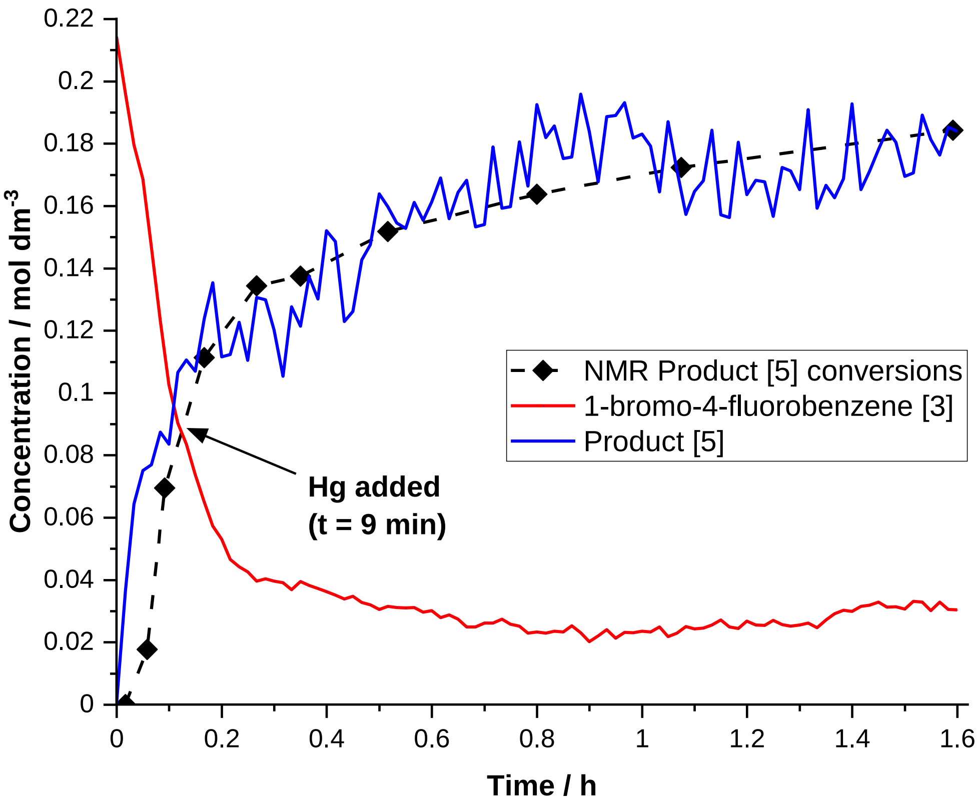

Figure 41: Mercury drop test using arylboronic pinacol ester 4a. Hg added at t = 9 min. Lab book ref. DRH-03-128

5. Transmission Electron Microscopy (TEM) for PdNP Observation

5.1 TEM from Suzuki Reaction Employing Arylboronic pinacol ester 4a

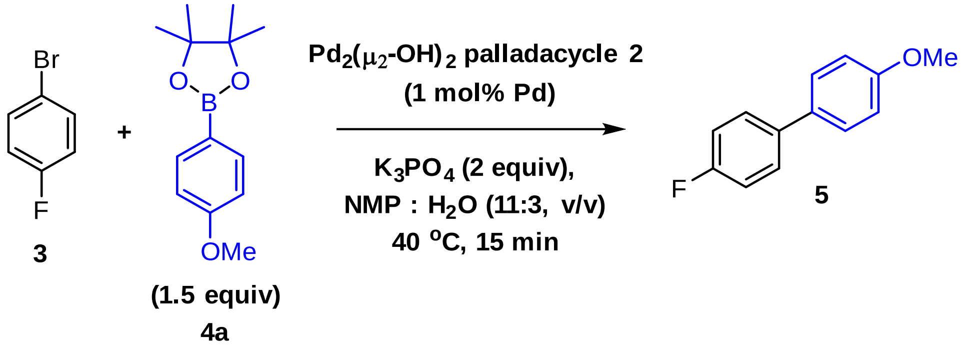

A Suzuki reaction following the General Procedure 2.2 at half scale (0.6 mmol aryl halide, 0.214 M effective concentration) was set up and sampled after 15 minutes (approx. 70% conversion). 1 mL of the reaction mixture was transferred to a vial, and the solvent removed by vigorous heating with a heat gun under vacuum to prepare the sample for TEM. Separately, 1 mL of reaction mixture was transferred to a vial containing 5.5 mg PVP polymer (27000), shaken and prepared for TEM in the same way.

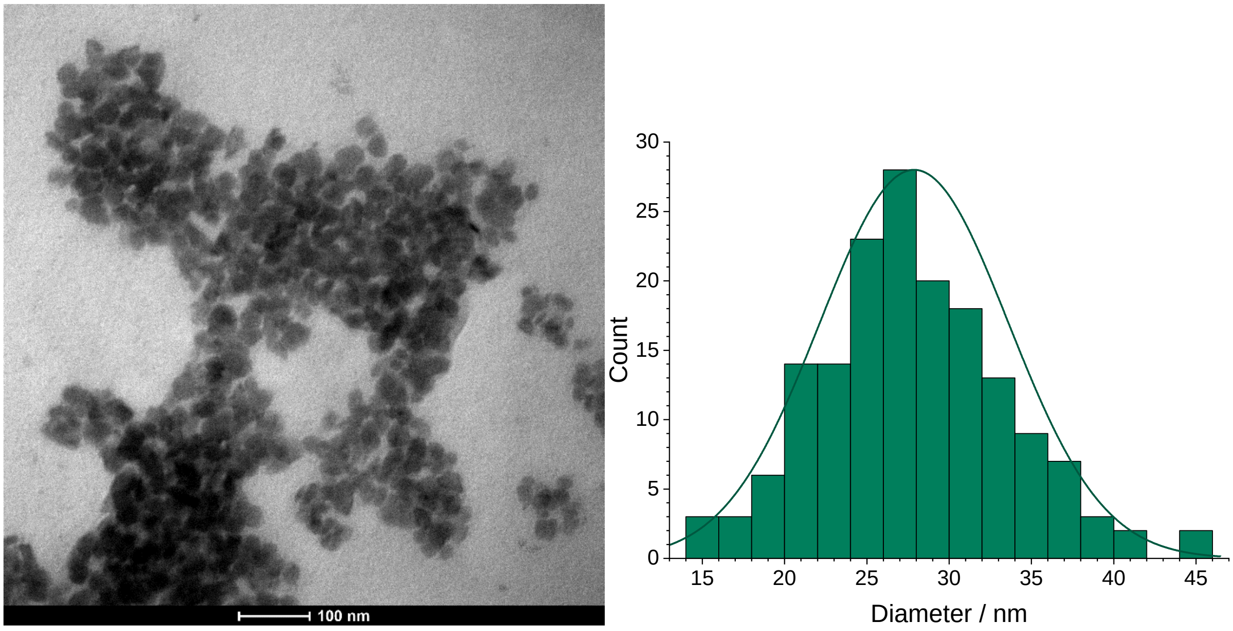

Figure 42: TEM image and distribution of measured nanoparticles from a Suzuki reaction employing arylboronic pinacol ester 4a, un-stabilized nanoparticles. Lab book ref. DRH-03-48-1

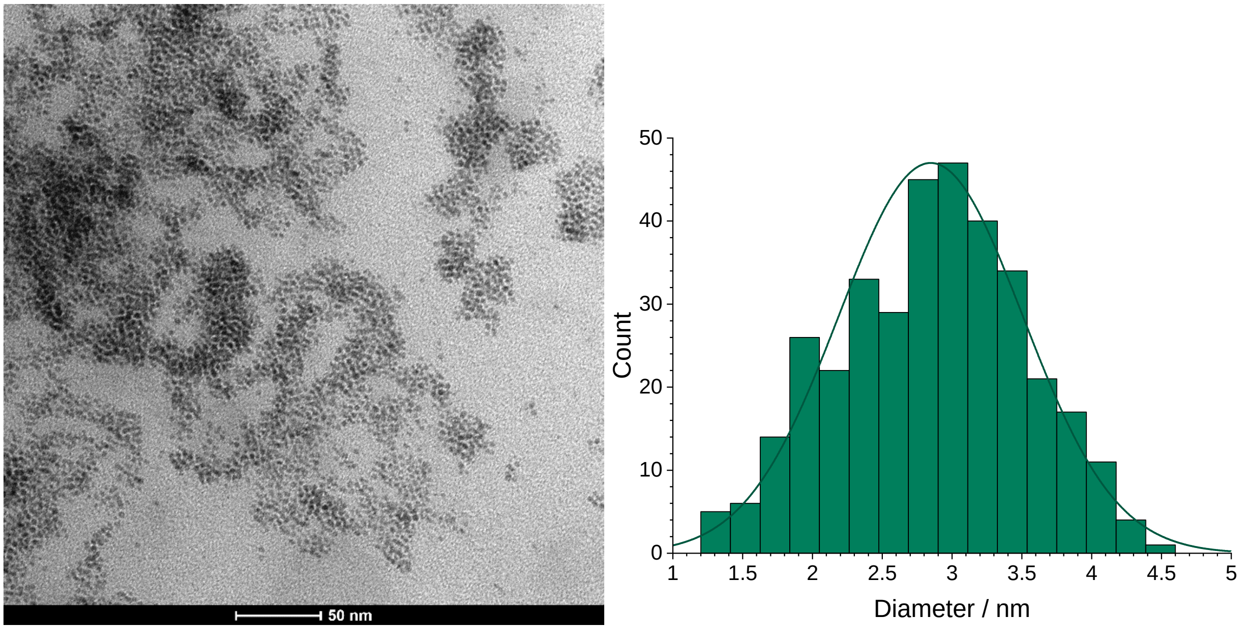

Figure 43: TEM image and distribution of measured nanoparticles from a Suzuki reaction employing arylboronic pinacol ester 4a, PVP-stabilized nanoparticles. Lab book ref. DRH-03-48-2

5.2 TEM from Suzuki Reaction Employing Arylboronic acid 4

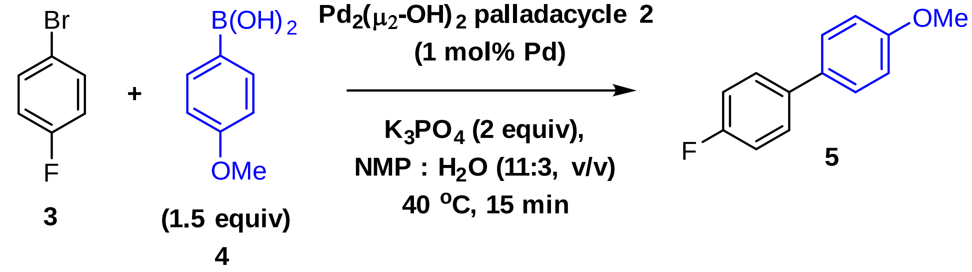

A Suzuki reaction following the General Procedure 2.2 at half scale (0.6 mmol aryl halide, 0.214 M effective concentration) was set up and sampled after 35 minutes (approx. 70% conversion). 1 mL of the reaction mixture was transferred to a vial, and the solvent removed by vigorous heating with a heat gun under vacuum to prepare the sample for TEM. Separately, 1 mL of reaction mixture was transferred to a vial containing 5.5 mg PVP polymer (27000), shaken and prepared for TEM in the same way.

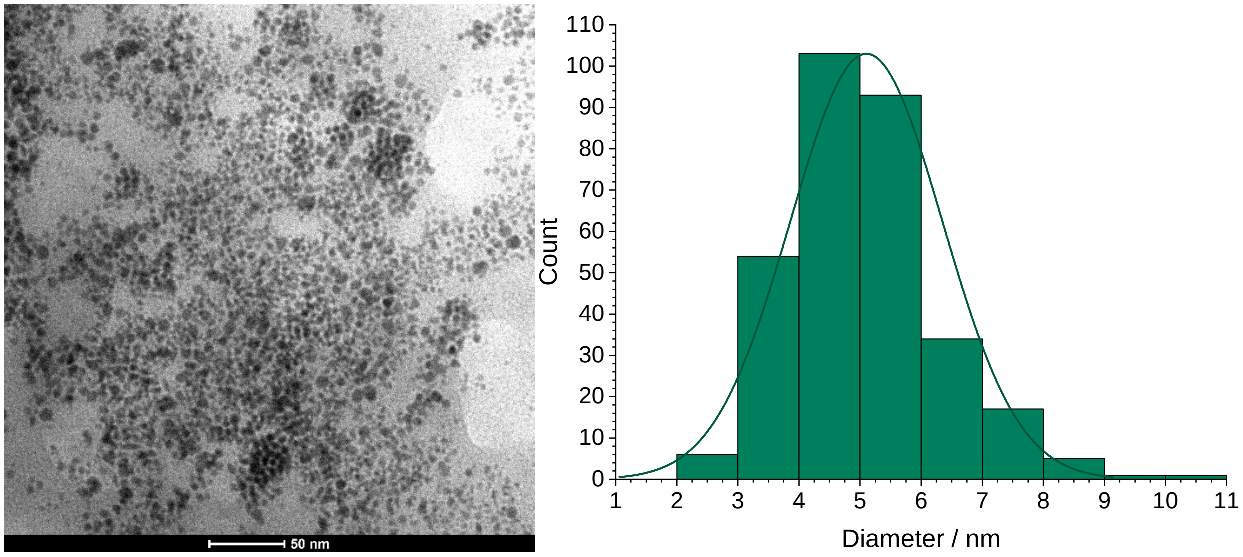

Figure 44: TEM image and distribution of measured nanoparticles from a Suzuki reaction employing arylboronic acid 4, un-stabilized nanoparticles. Lab book ref. DRH-03-61-1

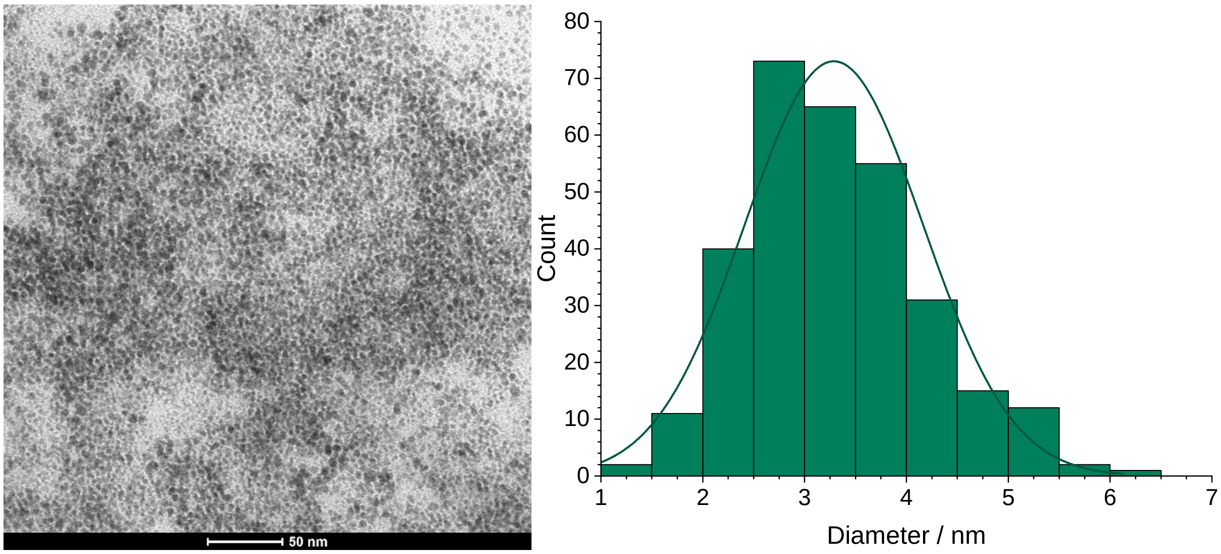

Figure 45: TEM image and distribution of measured nanoparticles from a Suzuki reaction employing arylboronic acid 4, PVP-stabilized nanoparticles. Lab book ref. DRH-03-61-2

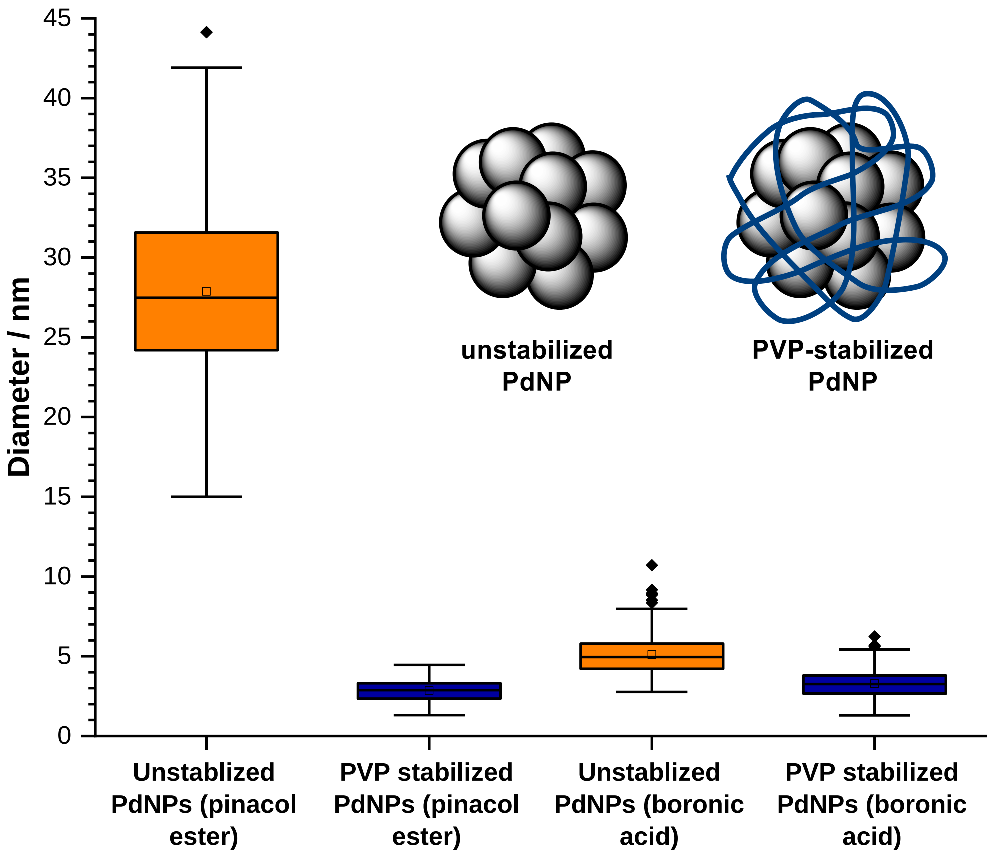

Figure 46: Statistical box and whisker plot of PdNP distribution (un-stabilized and PVP-stabilized) from TEM experiments with arylboronic pinacol ester 4a and arylboronic acid 4.

6. [Pd(C^P)(μ$_2$-OH)]$_2$ Palladacycle 2 Activation Data

6.1 Test for Arylation and Identification of Inert Pd(C^P)$_2$ Palladacycle 11, and Identification of Generated Species

[Pd(P^C)(μ$_2$-OH)]$_2$ palladacycle **2** (5 mg, 0.00586 mmol, 1 eq.) and pinacol (2.8 mg, 0.0235 mmol, 4 equiv.) were added to a J-Young tap NMR tube and evacuated and backfilled with N_2. THF (0.4 mL, dry, degassed) was added, followed by as a stock solution of 4-fluorophenylboronic acid **9** in THF (100 μL of [**9**] = 0.117 M, 0.0117 mmol, 2 equiv.). The reaction mixture was shaken to ensure mixing, and immediately analyzed by NMR spectroscopy.

After analysis, 1-bromo-4-fluorobenzene **3** (3 μL, 0.0235 mmol, 4 equiv.) was added to the reaction mixture under N_2, and the reaction mixture checked by NMR spectroscopy for oxidative addition.

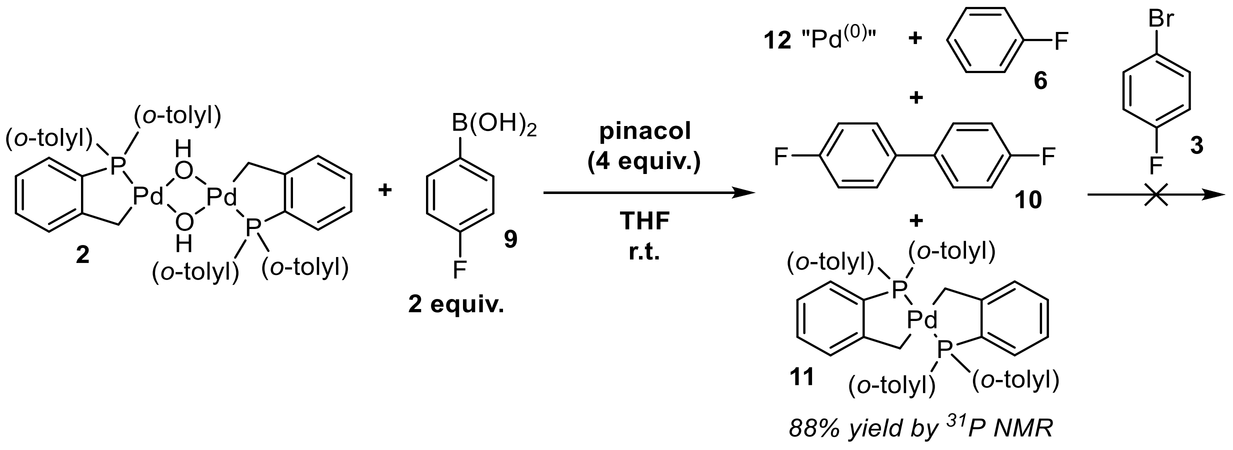

Lab book ref. DRH-03-30

There was no observed oxidative addition to form dimer **SI45**, with the species represented at ^{31}P δ 26.5 ppm being unreactive. This lack of reactivity confirmed that the proposed [Pd$^{(0)}$(P(*o*-tolyl)$_3$)] complex does not form, and the Pd$^{(II)}$ bipalladacycle **11** is the dominant observed species.

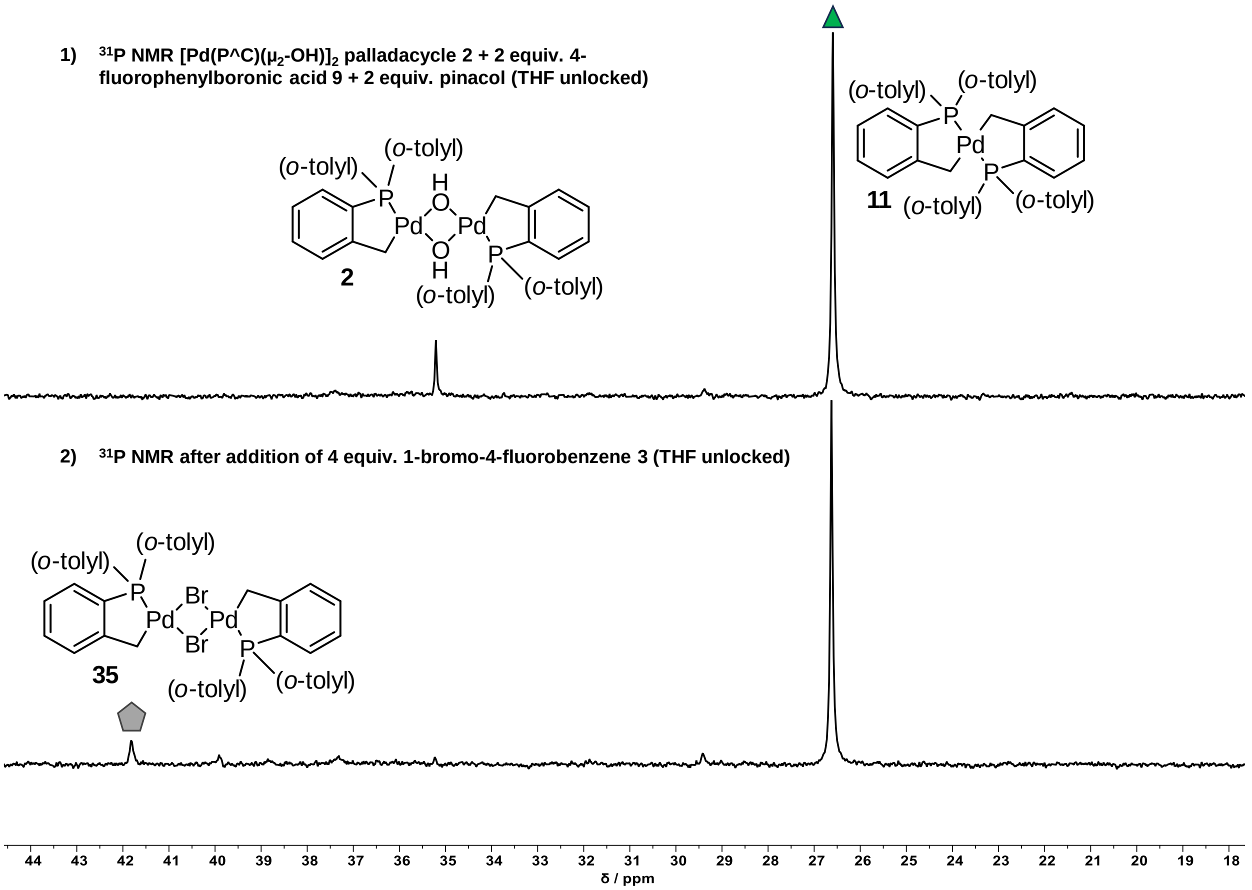

Figure 47: ^{31}P NMR (203 MHz, THF-unlocked, 298 K) showing the lack of oxidative addition by 1-bromo-4-fluorobenzene 3 in the presence of proposed $[Pd(P^{\wedge}C)]$ bipalladacycle 11 at 26.5 ppm (0.5 mL scale, concentration of $[Pd(P^{\wedge}C)(\mu_2\text{-}OH)]_2$ palladacycle 2 = [0.012 M]). Instead, $Pd_2(\mu_2\text{-}Br)_2$ palladacycle forms (grey triangle). Lab book ref. DRH-03-30. Integrals of 1): 2 = 0.11, 35 = 1.00, Integrals of 2): 35 = 0.09, 11 = 1.00

The species at ^{31}P δ 26.5 ppm was confirmed by LIFDI mass spectrometry of the reaction mixture to be $[Pd(P^{\wedge}C)]$ bipalladacycle **11** (HRMS (LIFDI) $(C_{42}H_{40}P_2Pd)^{+\bullet}$ *m/z* (calculated) 712.16346, (found) 712.16665, mass difference 4.48 ppm, lab book refs. DRH-02-41, DRH-02-176, DRH-03-54-2).

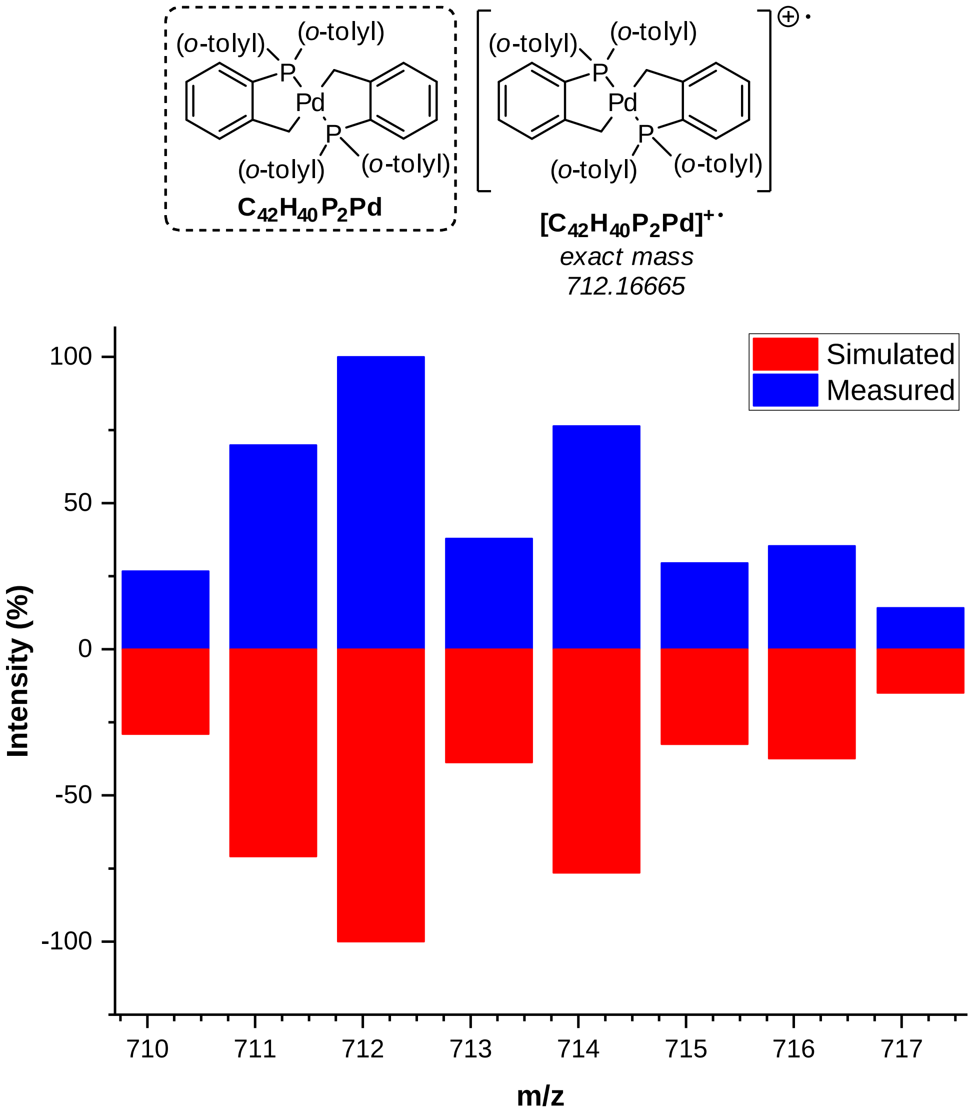

Figure 48: LIFDI mass spectrometry isotope pattern (measured *vs*. simulated) for the complex with m/z corresponding to $[C_{42}H_{40}P_2Pd]^{+\cdot}$ radical cation, suspected of being the [Pd(P^C)] bipalladacycle 11 (shown top left)

Further investigations revealed that fluorobenzene **6** (protodeboronated product) and 4,4'-difluorobiphenyl (homo-coupled product 10) were generated in this reaction. These species were confirmed by spiking known compounds into a reaction mixture after pre-catalyst activation (Figure 49).

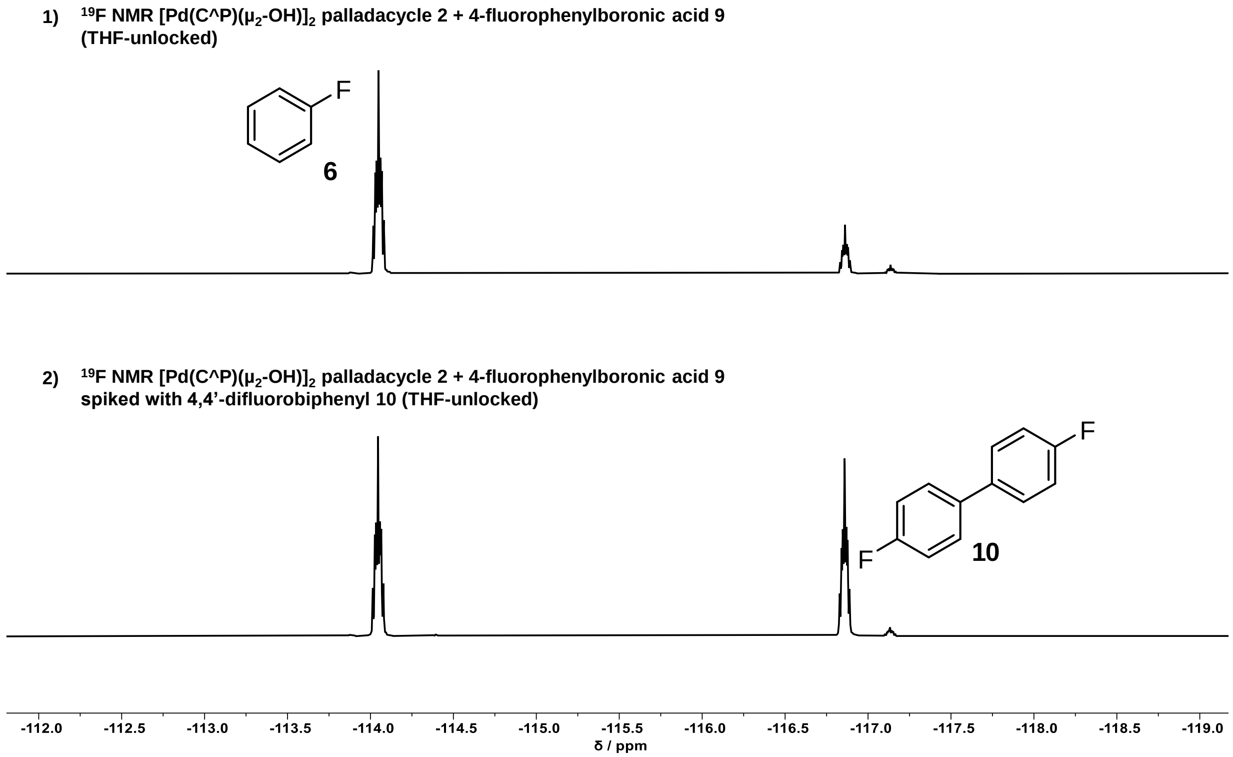

Figure 49: ^{19}F **NMR (470 MHz, THF-unlocked, 298 K) stacked spectra showing the identity of the products from activation of the $[Pd(P^{\wedge}C)(\mu_2\text{-}OH)]_2$ palladacycle 2 with arylboronic acid 9 (Scheme 4). These were determined by spiking the reaction mixture with authentic samples of fluorobenzene (not shown) and 4,4'difluorobiphenyl. Lab book ref. DRH-02-41-4**

6.2 General Procedure for Arylation

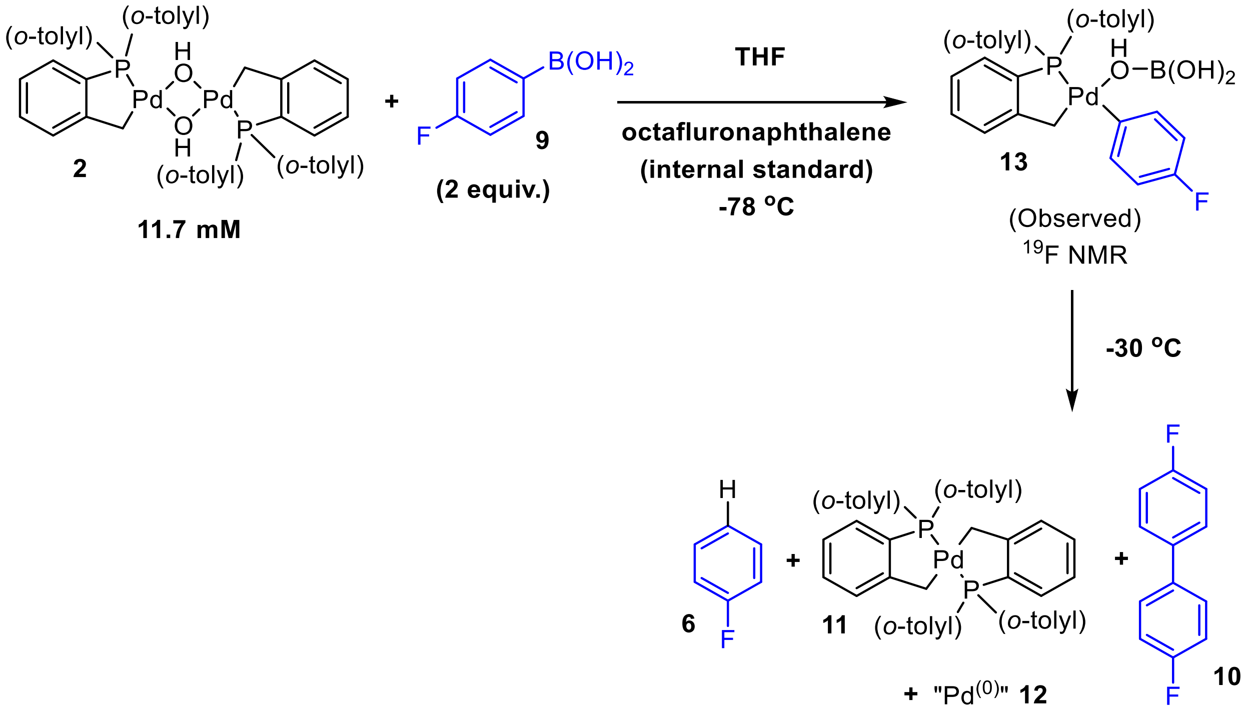

[Pd(P^C)(μ_2-OH)]$_2$ palladacycle **2** (5 mg, 0.00586 mmol, 1 eq.) and octafluoronapthalene (1.6 mg, 0.00586 mmol, 1 eq.) were dissolved in THF (0.40 mL, dry, degassed) in a J-Young tap NMR tube under a N_2 atmosphere. The tube was cooled to -78 °C, and 0.10 mL of a stock solution of 4-fluorophenylboronic acid **9** (and any other additive) in THF (16.4 mg in 1.0 mL dry, degassed THF, 2 eq. after addition) was added to the vessel. The tube was sealed under a N_2 atmosphere and vortexed at -78 °C, not shaken, to ensure mixing. The sample was loaded into a pre-cooled NMR spectrometer (500 MHz) at 243 K, and analyzed by ^{19}F NMR timecourse (8 scans, relaxation delay 5 s, acquisition time 1 s) every 3 minutes. The peaks followed were Pd transmetallation complex **13** at -123 .7 ppm, fluorobenzene **6** at -114.4 ppm, and 4,4'-difluorobiphenyl **10** at -117.2 ppm. Slight drift in peak positions was observed over different experiments due to small tuning changes in the spectrometer.

Using Mestrenova, absolute integrals of peaks were followed, converted to concentrations using the octafluoronapthalene peak at -147.9 ppm, and plotted vs time. Rate constants were determined by fitting experimental data in OrginPro 2022b (64-bit) SR1 9.9.5.171 using the equation y = A1*exp(x/t1). The rate constant was obtained from calculating k = 1/t1

Compounds were verified in reference to the literature[12] and via spiking with authentic samples. ^{31}P NMR was used to verify the identity of Pd$^{(0)}$ complex. A more detailed discussion around complex **13** (observation of formation and degradation by ^{31}P and ^{19}F NMR at different temperatures) can be found in Section 7.3.

6.3 Run 1 – Unchanged General Procedure 6.2

Lab book ref. DRH-02-110

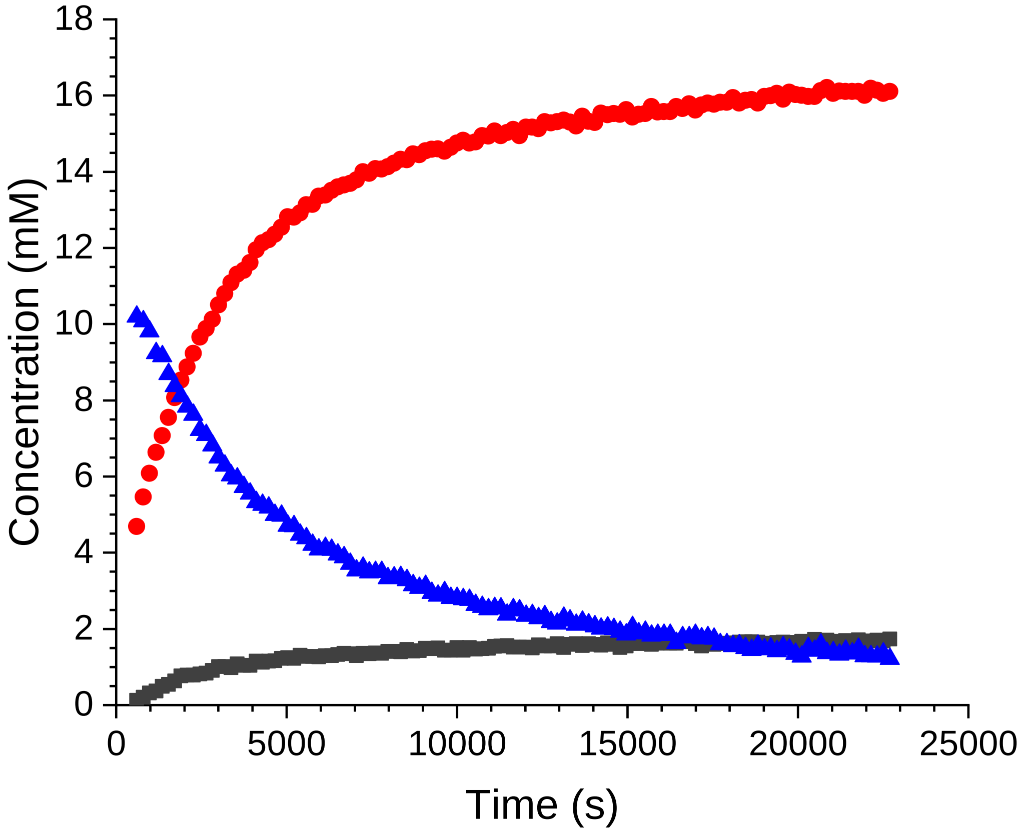

Figure 50: Concentration vs. time plot for the activation of [Pd(P^C)(μ_2-OH)]$_2$ palladacycle 2 using arylboronic acid 9 in THF. Concentrations calculated from an octafluoronapthelene internal standard. Key: ▲ Pd transmetallation complex 13; ● fluorobenzene 6; ■ 4,4'-difluorobiphenyl 13.

Table 6: Fitted rate constants for Run 1 from concentration vs. time plot

	**Peak at -117.2 (10)**	**Peak at – 114.4 (6)**	**Peak at -123.7 (13)**
**Rate constant / s^{-1}**	2.585 x10^{-4}	2.610 x10^{-4}	-2.074 x10^{-4}
**Error / s^{-1}**	0.076 x10^{-4}	0.043 x10^{-4}	0.032 x10^{-4}

Table 7: Data for Run 1 concentration vs. time plot

**Time / s**	**Peak at -117.2 (10) / mM**	**Peak at – 114.4 (6) / mM**	**Peak at -123.7 (13) / mM**
**600**	0.126025	4.683251	10.20176

**800**	0.199818	5.456533	10.07348
**983**	0.311924	6.083309	9.812615
**1168**	0.365849	6.634771	9.23486
**1352**	0.485627	7.06778	9.157795
**1535**	0.541455	7.545469	8.690666
**1719**	0.63021	8.07173	8.375611
**1903**	0.764275	8.530612	8.11158
**2087**	0.788289	8.874905	7.833826
**2271**	0.77952	9.232389	7.618227
**2455**	0.818096	9.653071	7.22236
**2639**	0.831578	9.878773	7.089741
**2823**	0.905457	10.13038	6.814673
**3007**	1.006347	10.49987	6.502547
**3191**	1.006341	10.79801	6.284354
**3375**	0.97647	11.08764	6.027509
**3559**	1.075603	11.31125	5.951049
**3743**	1.041697	11.41215	5.728289
**3927**	1.041261	11.6142	5.547265
**4111**	1.147699	11.94737	5.328503
**4295**	1.119542	12.1317	5.251574
**4479**	1.152197	12.20902	5.183099
**4663**	1.1582	12.35908	4.98963
**4847**	1.220719	12.53632	4.970153
**5031**	1.245713	12.81082	4.707271
**5215**	1.223092	12.80854	4.700295
**5399**	1.304405	12.91845	4.467481
**5583**	1.269305	13.1313	4.382411
**5767**	1.27626	13.13666	4.210782
**5951**	1.258685	13.35902	4.086662
**6135**	1.290554	13.38063	4.12809
**6319**	1.293639	13.50428	4.076009
**6503**	1.328489	13.59999	3.953895
**6687**	1.358346	13.65239	3.883139
**6871**	1.347182	13.69345	3.704549
**7055**	1.294702	13.78534	3.534705
**7239**	1.356323	13.99572	3.605629
**7423**	1.343011	13.95628	3.483553
**7607**	1.367328	14.0808	3.490777

**7791**	1.354627	14.073	3.492414
**7975**	1.406886	14.12437	3.332078
**8159**	1.407447	14.21974	3.353703
**8343**	1.390907	14.31961	3.357368
**8527**	1.456465	14.30844	3.279917
**8711**	1.41502	14.46287	3.149683
**8895**	1.421761	14.44589	3.072854
**9079**	1.486233	14.54461	3.131789
**9263**	1.475961	14.58705	2.939359
**9447**	1.498863	14.59992	2.876405
**9631**	1.439639	14.53948	2.967163
**9815**	1.439263	14.63928	2.811832
**9999**	1.511007	14.74571	2.807116
**10183**	1.435698	14.81903	2.776961
**10367**	1.511928	14.75159	2.757449
**10551**	1.466141	14.77821	2.630174
**10735**	1.479572	14.94392	2.573852
**10919**	1.491899	14.93053	2.519494
**11103**	1.539578	15.06152	2.550546
**11287**	1.545267	14.9397	2.53389
**11471**	1.556256	15.02629	2.376959
**11655**	1.521216	15.10471	2.513856
**11839**	1.516839	14.94368	2.48578
**12023**	1.514975	15.16474	2.337932
**12207**	1.498234	15.17038	2.361063
**12391**	1.579464	15.12823	2.286322
**12575**	1.547573	15.31162	2.32904
**12759**	1.550127	15.28293	2.175731
**12943**	1.607975	15.30744	2.137646
**13127**	1.511765	15.35216	2.296202
**13311**	1.584293	15.3047	2.21819
**13495**	1.613594	15.19657	2.109477
**13679**	1.558305	15.45723	2.187068
**13863**	1.609327	15.32132	2.122223
**14047**	1.590823	15.28937	2.070009
**14231**	1.56894	15.53154	2.006895
**14415**	1.627372	15.48954	2.032441
**14599**	1.585188	15.52877	2.000319
**14783**	1.503813	15.50908	1.923249
**14967**	1.548172	15.62168	1.856713
**15151**	1.622041	15.43302	2.048568
**15335**	1.612385	15.50413	1.88854
**15519**	1.648274	15.52336	1.922727

**15703**	1.585247	15.71288	1.825492
**15887**	1.618157	15.5677	1.848886
**16071**	1.643448	15.57393	1.842621
**16255**	1.618104	15.58044	1.844937
**16439**	1.622122	15.70314	1.631816
**16623**	1.685182	15.65972	1.781635
**16807**	1.660283	15.77633	1.784117
**16991**	1.610521	15.61668	1.84518
**17175**	1.547906	15.75374	1.753056
**17358**	1.605639	15.79578	1.78359
**17542**	1.588637	15.77262	1.738293
**17726**	1.615954	15.82328	1.600422
**17911**	1.613342	15.81884	1.601708
**18095**	1.631418	15.94286	1.548931
**18279**	1.647792	15.80093	1.573109
**18463**	1.664592	15.86865	1.494713
**18647**	1.657366	15.89567	1.449838
**18831**	1.652415	15.80107	1.556798
**19015**	1.611269	15.97163	1.471752
**19199**	1.617482	15.99819	1.484621
**19383**	1.64367	16.05175	1.419787
**19567**	1.646534	15.8978	1.518135
**19750**	1.642553	16.08358	1.452344
**19935**	1.635734	16.02216	1.345273
**20118**	1.669888	16.00129	1.269671
**20303**	1.657197	15.97492	1.502388
**20486**	1.719539	15.96811	1.424055
**20670**	1.695151	16.12093	1.588645
**20855**	1.699526	16.20402	1.362369
**21039**	1.624106	16.05066	1.386208
**21223**	1.673654	16.11923	1.327437
**21406**	1.69373	16.10104	1.422983
**21591**	1.597858	16.10377	1.358119
**21775**	1.71107	16.10155	1.474808
**21959**	1.637584	15.99887	1.278862
**22143**	1.676244	16.19108	1.27907
**22327**	1.699837	16.13878	1.26931
**22510**	1.678251	16.05683	1.351615
**22695**	1.730471	16.10205	1.211296

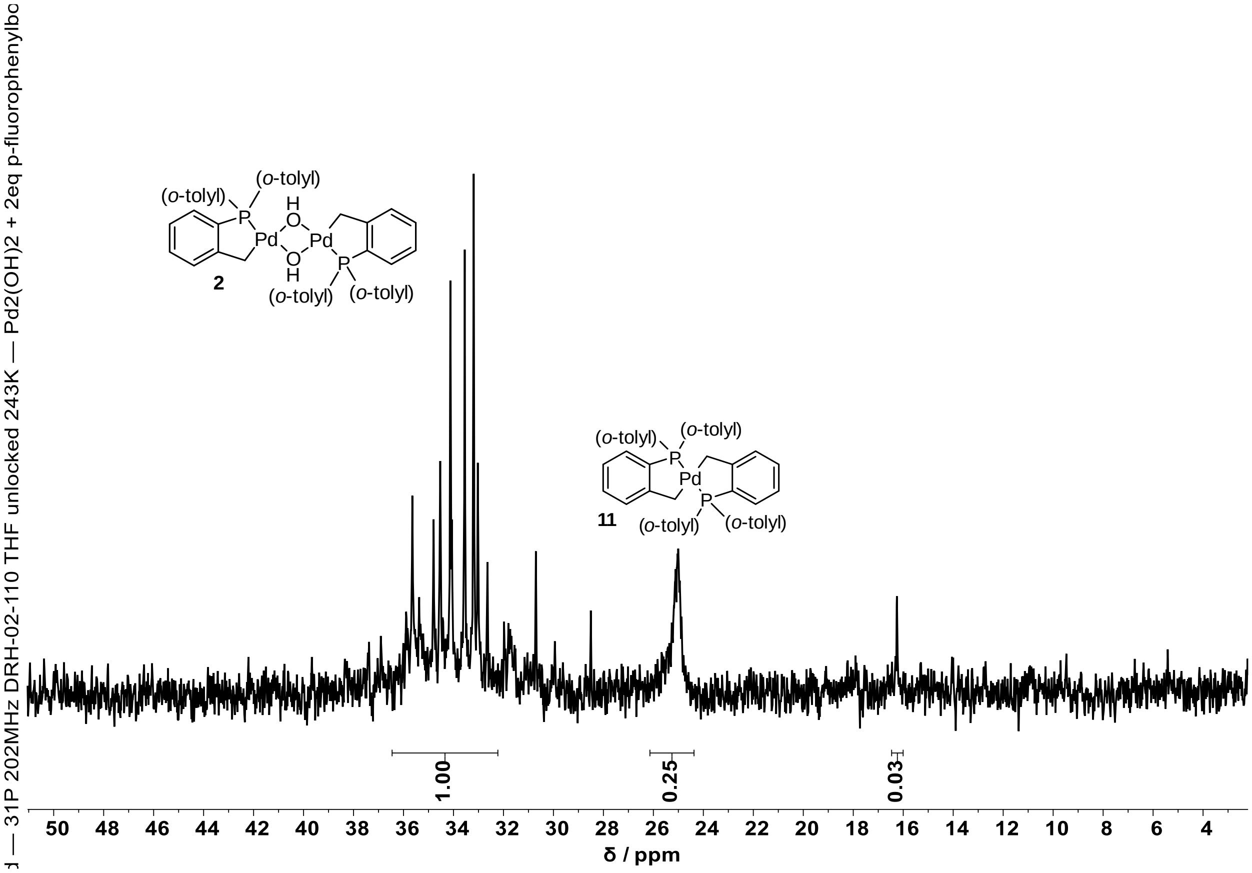

Figure 51: ^{31}P NMR (243 MHz, THF-unlocked, 243 K) spectrum after the kinetic run. Lab book ref. DRH-02-110.

6.4 Run 2 – General Procedure 6.2 with D_2O additive

Lab book ref. DRH-02-110-2

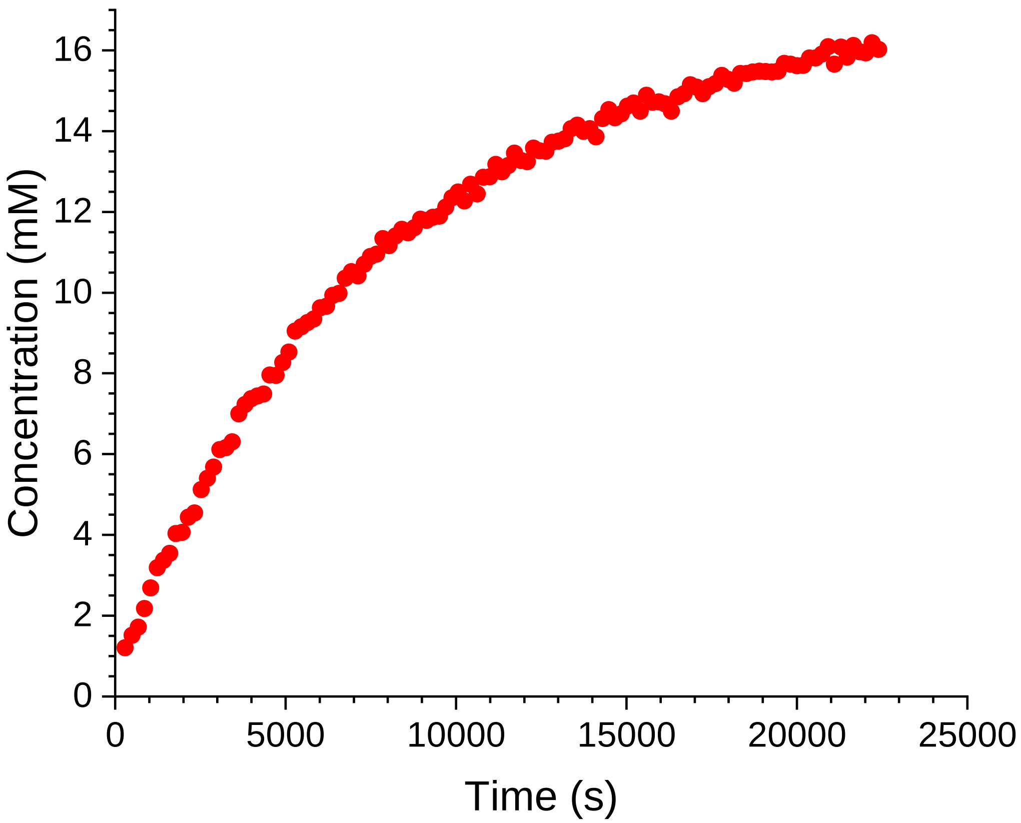

Figure 52: Concentration vs. time plot for the activation of [Pd(P^C)(μ_2-OH)]$_2$ palladacycle 2 using arylboronic acid 9 in THF with D_2O additive. Concentrations calculated from an octafluoronapthelene internal standard. Key: ● fluorobenzene 6. Only fluorobenzene was observed as product in this run, with no Pd complex observed. K = 1.313 x10^{-4} ± 0.014 s^{-1}

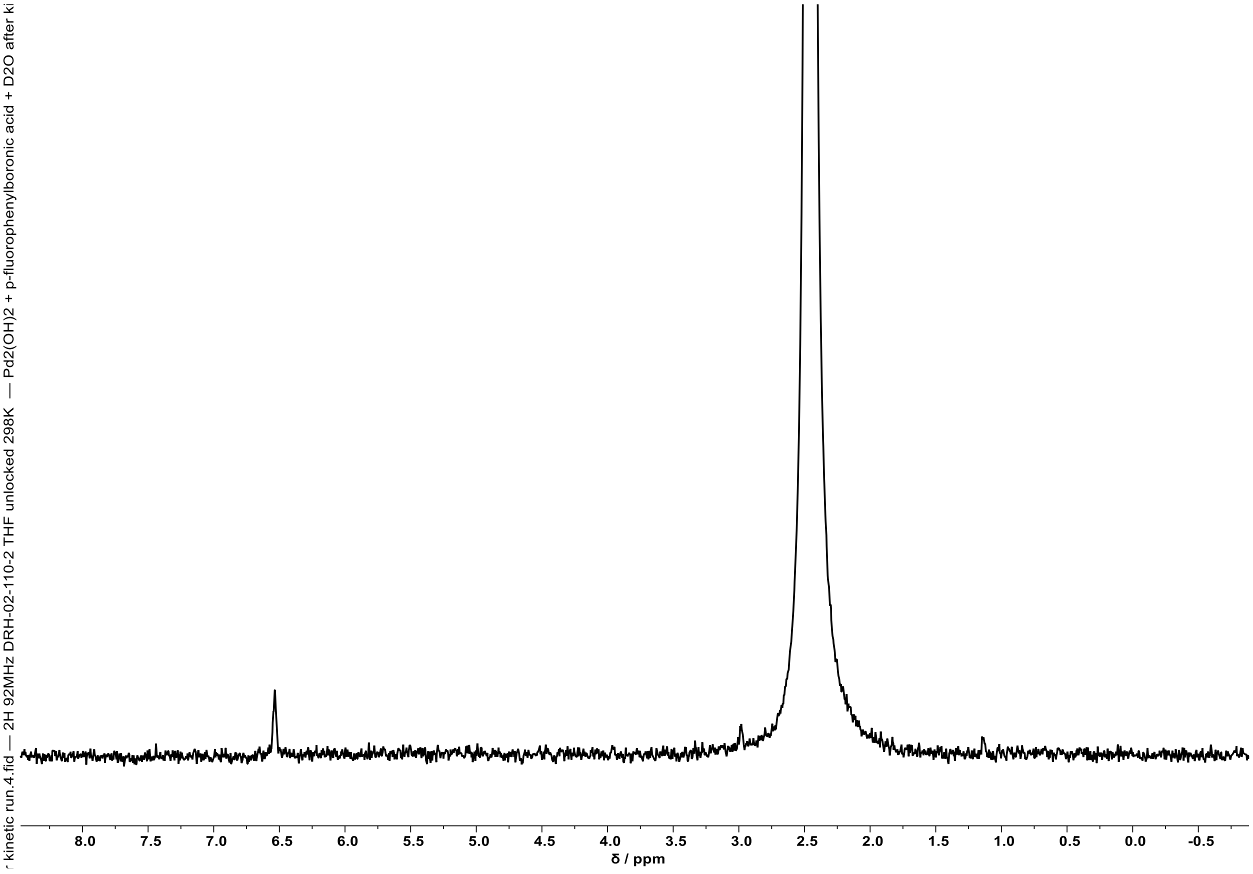

Figure 53: ^{2}H NMR (THF-unlocked, 92 MHz, 128 scans, 298K) spectrum of reaction end-point of Run 2. ^{2}H incorporation observed at 6.5 ppm – fluorobenzene 6, and 3.0 ppm (P(*o*-tolyl)$_3$ ligand CH_2D).

Table 8: Data for Run 2 concentration vs. time plot

Time / s	Peak at – 114.4 (6) / mM
**300**	1.198362
**499**	1.509397
**683**	1.716426
**868**	2.177033
**1052**	2.683964
**1236**	3.186711
**1420**	3.365764
**1604**	3.540333
**1788**	4.03233
**1972**	4.060909

**2156**	4.432616
**2340**	4.542155
**2524**	5.121424
**2708**	5.392793
**2892**	5.678314
**3076**	6.106329
**3260**	6.160574
**3444**	6.300195
**3628**	6.992408
**3812**	7.228375
**3996**	7.369048
**4180**	7.433048
**4364**	7.48198
**4548**	7.9518
**4732**	7.947205
**4916**	8.261528
**5101**	8.525715
**5285**	9.046755
**5469**	9.153252
**5652**	9.252395
**5837**	9.345175
**6021**	9.616105
**6205**	9.654707
**6389**	9.925967
**6573**	9.976517
**6757**	10.35199
**6941**	10.51279
**7125**	10.40824
**7309**	10.70154
**7493**	10.89155
**7677**	10.95197
**7861**	11.33081
**8045**	11.15994
**8229**	11.39626
**8413**	11.56561
**8597**	11.47368
**8781**	11.59911
**8965**	11.81249
**9149**	11.78504
**9333**	11.8627
**9517**	11.89585
**9701**	12.11128
**9885**	12.34626

**10069**	12.48584
**10253**	12.2692
**10437**	12.67728
**10621**	12.44233
**10805**	12.85155
**10989**	12.86239
**11173**	13.16774
**11357**	12.98878
**11541**	13.1383
**11725**	13.44904
**11909**	13.26829
**12093**	13.24255
**12277**	13.57643
**12461**	13.51017
**12645**	13.49928
**12829**	13.7198
**13013**	13.74811
**13197**	13.81005
**13381**	14.06064
**13565**	14.14481
**13749**	13.98731
**13933**	14.05157
**14117**	13.85051
**14302**	14.30873
**14486**	14.52362
**14670**	14.32427
**14854**	14.42105
**15037**	14.61242
**15221**	14.6918
**15406**	14.49253
**15590**	14.88242
**15774**	14.70696
**15958**	14.72354
**16142**	14.67483
**16326**	14.49312
**16510**	14.84021
**16694**	14.91885
**16878**	15.14719
**17062**	15.08204
**17246**	14.92255
**17430**	15.09039
**17614**	15.16891
**17798**	15.37649

**17982**	15.28219
**18166**	15.17774
**18350**	15.42399
**18534**	15.42307
**18718**	15.46063
**18902**	15.48324
**19086**	15.46907
**19270**	15.46
**19454**	15.47681
**19638**	15.66832
**19822**	15.6514
**20006**	15.61043
**20190**	15.62805
**20374**	15.81032
**20558**	15.80311
**20742**	15.90357
**20926**	16.08323
**21110**	15.65319
**21294**	16.08004
**21478**	15.82917
**21662**	16.11259
**21846**	15.96048
**22030**	15.93413
**22214**	16.17924
**22398**	16.0224

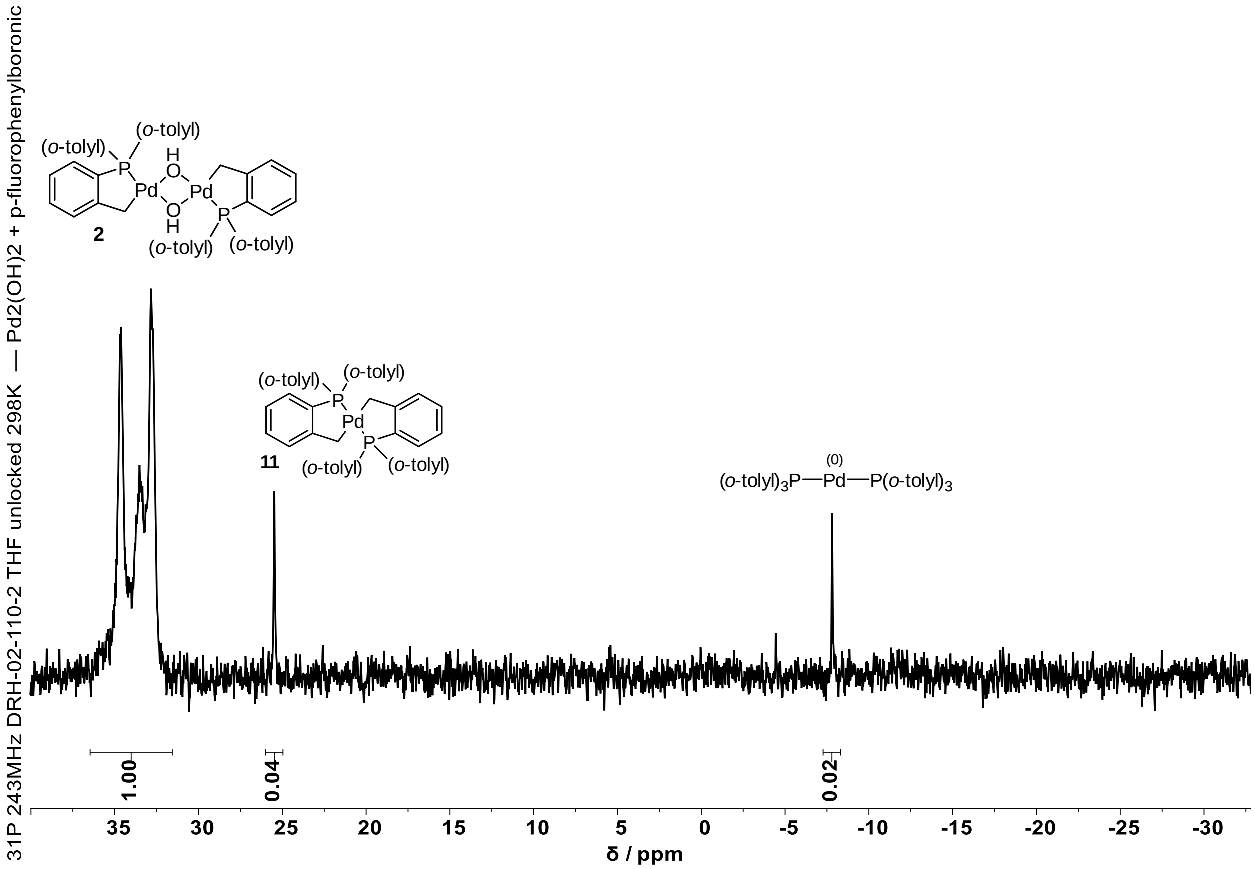

Figure 54: ^{31}P NMR (243 MHz, THF-unlocked, 298 K) spectrum after the kinetic run in the presence of D_2O. Lab book ref. DRH-02-110-2

6.5 Run 3 – General Procedure 6.2 with Boric Acid Additive (4 equiv.)

Lab book ref. DRH-03-32

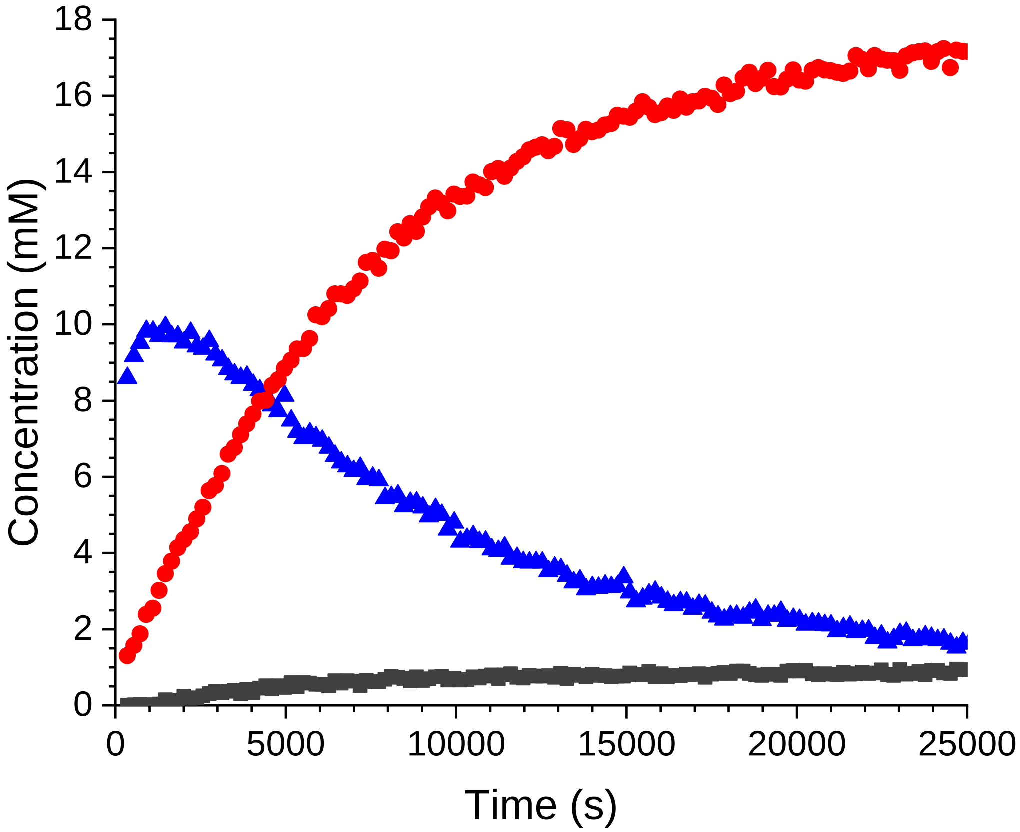

Figure 55: Concentration vs. time plot for the activation of [Pd(P^C)(μ$_2$-OH)]$_2$ palladacycle 2 using arylboronic acid 9 in THF with boric acid additive. Concentrations calculated from an octafluoronapthelene internal standard. Key: ▲ Pd transmetallation complex 13; ● fluorobenzene 6; ■ 4,4'-difluorobiphenyl 10.

Table 9: Fitted rate constants for Run 3 from concentration vs. time plot. The fitting for 13 was started at 4000 s to avoid the initial complex formation period.

	Peak at -117.2 (10)	Peak at – 114.4 (6)	Peak at -123.7 (13)
**Rate constant / s^{-1}**	1.877 x10^{-4}	1.363 x10^{-4}	-1.216 x10^{-4}
**Error / s^{-1}**	0.071 x10^{-4}	0.011 x10^{-4}	0.020 x10^{-4}

Table 10: Data for Run 3 concentration vs. time plot

Time / s	Peak at -117.2 (10) / mM	Peak at – 114.4 (6) / mM	Peak at -123.7 (13) / mM
**350**	0.00353	1.308823	8.601496
**547**	0.007003	1.570033	9.164561
**731**	0.022518	1.873291	9.511365
**916**	0.013087	2.38701	9.833307
**1100**	0.009931	2.546615	9.804768
**1284**	0.028782	3.016405	9.698428
**1468**	0.140904	3.450125	9.934258
**1653**	0.114767	3.780838	9.69212
**1837**	0.129868	4.130841	9.682798
**2021**	0.237194	4.350319	9.522418
**2206**	0.182454	4.549717	9.782468
**2390**	0.194099	4.893728	9.425826
**2574**	0.242229	5.197467	9.36669
**2758**	0.30903	5.638134	9.565911
**2943**	0.351729	5.761202	9.211469
**3127**	0.322354	6.0838	9.054302
**3311**	0.365123	6.589341	8.829479
**3496**	0.382613	6.768187	8.69215
**3680**	0.30507	7.102432	8.598268
**3864**	0.415182	7.390414	8.628111
**4048**	0.337571	7.639437	8.409194
**4233**	0.447074	7.980821	8.269991
**4417**	0.50905	8.005224	8.10425
**4601**	0.437535	8.396391	7.874226
**4786**	0.511317	8.550327	7.721724
**4970**	0.465762	8.839378	8.129329
**5154**	0.593258	9.054796	7.472905
**5338**	0.489876	9.350084	7.182944
**5523**	0.593577	9.35713	7.019708
**5707**	0.57768	9.623463	7.135829
**5891**	0.552261	10.24845	7.043377
**6076**	0.553803	10.19925	6.943875
**6260**	0.506388	10.41134	6.766442
**6444**	0.646001	10.79822	6.548835
**6629**	0.579893	10.80298	6.376663

**6813**	0.643218	10.75395	6.280014
**6997**	0.629711	10.93266	6.155307
**7182**	0.515748	11.13465	6.230538
**7366**	0.648464	11.62274	5.941539
**7550**	0.623812	11.67744	5.979171
**7734**	0.615321	11.47376	5.909839
**7919**	0.68322	11.96528	5.441203
**8103**	0.754907	11.93102	5.473838
**8287**	0.732077	12.42542	5.508206
**8471**	0.702543	12.26374	5.226277
**8656**	0.64681	12.6461	5.312872
**8840**	0.739306	12.43737	5.322824
**9024**	0.649302	12.81823	5.197756
**9208**	0.688432	13.08049	4.957979
**9393**	0.743403	13.31312	5.156338
**9577**	0.750274	13.18155	4.999244
**9761**	0.659912	12.97521	4.610861
**9946**	0.706051	13.41981	4.800639
**10130**	0.663	13.35848	4.296972
**10314**	0.674966	13.36122	4.37399
**10498**	0.738687	13.72817	4.44115
**10683**	0.711501	13.66319	4.288587
**10867**	0.761832	13.59019	4.300162
**11051**	0.791202	14.00537	4.09467
**11236**	0.70235	14.09239	4.054551
**11420**	0.793418	13.88587	4.141561
**11604**	0.827375	14.10231	3.847334
**11788**	0.734556	14.27192	3.873163
**11973**	0.710829	14.3922	3.75441
**12157**	0.788883	14.57944	3.75239
**12341**	0.759656	14.64928	3.76227
**12526**	0.762972	14.7126	3.750361
**12710**	0.774395	14.55353	3.520878
**12894**	0.735839	14.66625	3.618686
**13078**	0.837366	15.13989	3.57359
**13263**	0.704207	15.10891	3.405411
**13447**	0.813257	14.71604	3.227385
**13631**	0.788442	14.87605	3.275715
**13815**	0.754098	15.11408	3.049817
**13999**	0.814269	15.0533	3.110032
**14184**	0.782845	15.09724	3.087841
**14368**	0.769957	15.22813	3.150609
**14552**	0.744658	15.27221	3.107166

**14736**	0.768955	15.48324	3.125068
**14921**	0.761399	15.45961	3.366013
**15105**	0.84734	15.43706	2.96314
**15289**	0.803265	15.59476	2.733572
**15474**	0.797789	15.83759	2.802483
**15658**	0.888269	15.70096	2.916055
**15842**	0.755662	15.50804	2.987286
**16027**	0.827188	15.55058	2.829262
**16211**	0.743144	15.72646	2.716058
**16395**	0.788079	15.62023	2.628802
**16580**	0.775168	15.90918	2.709354
**16764**	0.817481	15.69624	2.70376
**16948**	0.809338	15.84537	2.533555
**17132**	0.821404	15.86028	2.639972
**17317**	0.733456	15.9839	2.618878
**17501**	0.817974	15.93696	2.437258
**17685**	0.837443	15.77245	2.336566
**17870**	0.856311	16.27894	2.25303
**18054**	0.838861	16.04925	2.343904
**18238**	0.900119	16.11118	2.351989
**18423**	0.899751	16.46673	2.304224
**18607**	0.83639	16.61233	2.437602
**18791**	0.795656	16.31755	2.512888
**18975**	0.785074	16.45103	2.245197
**19160**	0.811558	16.66643	2.353843
**19344**	0.818363	16.23472	2.349349
**19528**	0.789062	16.23106	2.450575
**19713**	0.897618	16.42796	2.219316
**19897**	0.911707	16.67479	2.272349
**20081**	0.901197	16.4152	2.236474
**20266**	0.914124	16.37923	2.115949
**20450**	0.827097	16.67027	2.159751
**20634**	0.790181	16.7344	2.14944
**20819**	0.824503	16.67921	2.10474
**21003**	0.819019	16.65968	2.096224
**21187**	0.807524	16.61719	1.946841
**21372**	0.862719	16.5807	2.027174
**21556**	0.810853	16.64506	2.066778
**21740**	0.822765	17.04754	1.925411
**21925**	0.864864	16.95309	1.955109
**22109**	0.838505	16.70931	1.961881
**22293**	0.841928	17.0501	1.777511
**22478**	0.931619	16.96258	1.835485

**22663**	0.807858	16.93367	1.652037
**22847**	0.785888	16.92323	1.740442
**23031**	0.935462	16.66638	1.879201
**23215**	0.809929	17.04617	1.903056
**23400**	0.838876	17.12707	1.715613
**23584**	0.884553	17.15881	1.73577
**23768**	0.802904	17.17953	1.816703
**23953**	0.907535	16.89851	1.772839
**24137**	0.91261	17.15579	1.714524
**24321**	0.845856	17.23625	1.732378
**24505**	0.835683	16.74148	1.622382
**24690**	0.94352	17.19673	1.516173
**24874**	0.93798	17.16703	1.635149
**25058**	0.927365	17.15833	1.616999
**25243**	0.826451	17.17506	1.513134
**25427**	0.851561	17.31808	1.710349
**25611**	0.937956	17.24116	1.595258
**25795**	0.908372	17.12941	1.606106
**25979**	0.871093	17.40011	1.696144
**26164**	0.816747	17.57738	1.465817
**26348**	0.899492	17.49823	1.49007
**26532**	0.842173	17.02273	1.646968
**26717**	0.818788	17.50367	1.556868
**26901**	0.752031	17.41077	1.490283
**27085**	0.916265	17.07332	1.446375
**27270**	0.809475	17.4251	1.48798
**27454**	0.898948	17.50435	1.528379
**27638**	0.9057	17.42184	1.504891
**27823**	0.905413	17.6714	1.248439
**28007**	0.879687	17.35234	1.406296
**28191**	0.945286	17.37485	1.496882
**28375**	0.807073	17.39903	1.377773
**28559**	0.960722	17.50387	1.497276
**28744**	0.863847	17.66136	1.367419
**28928**	0.856213	17.85759	1.435333
**29112**	0.9464	17.83662	1.136142
**29297**	0.950341	17.59943	1.218212
**29481**	0.911987	17.6107	1.353779
**29665**	0.851577	17.59305	1.270987
**29849**	0.88112	17.60375	1.336312

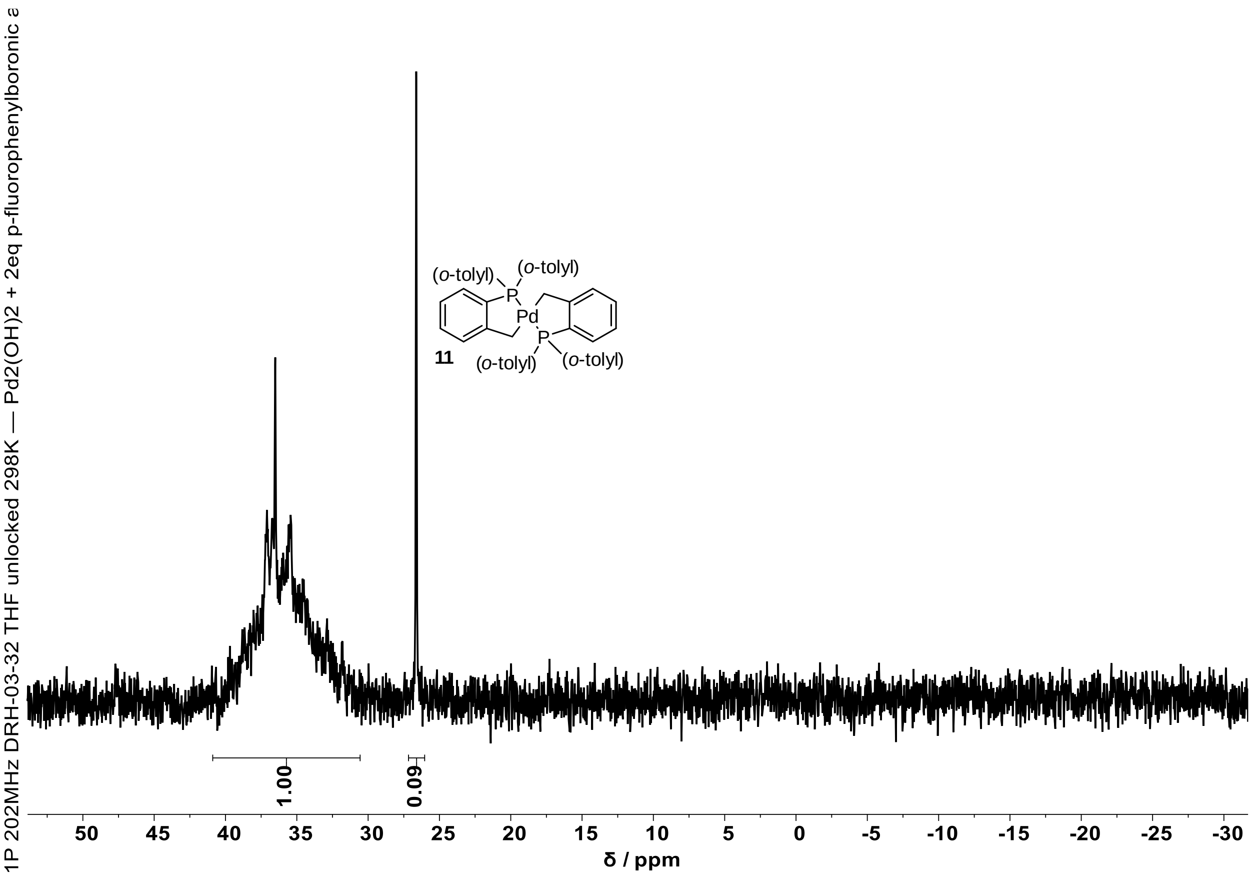

Figure 56: ^{31}P NMR (202 MHz, THF-unlocked, 298 K) spectrum after the kinetic run in the presence of excess boric acid. Lab book ref. DRH-03-32.

6.6 Run 4 – General Procedure 6.2 with Excess Arylboronic Acid (10 equiv.)

Lab book ref. DRH-02-111

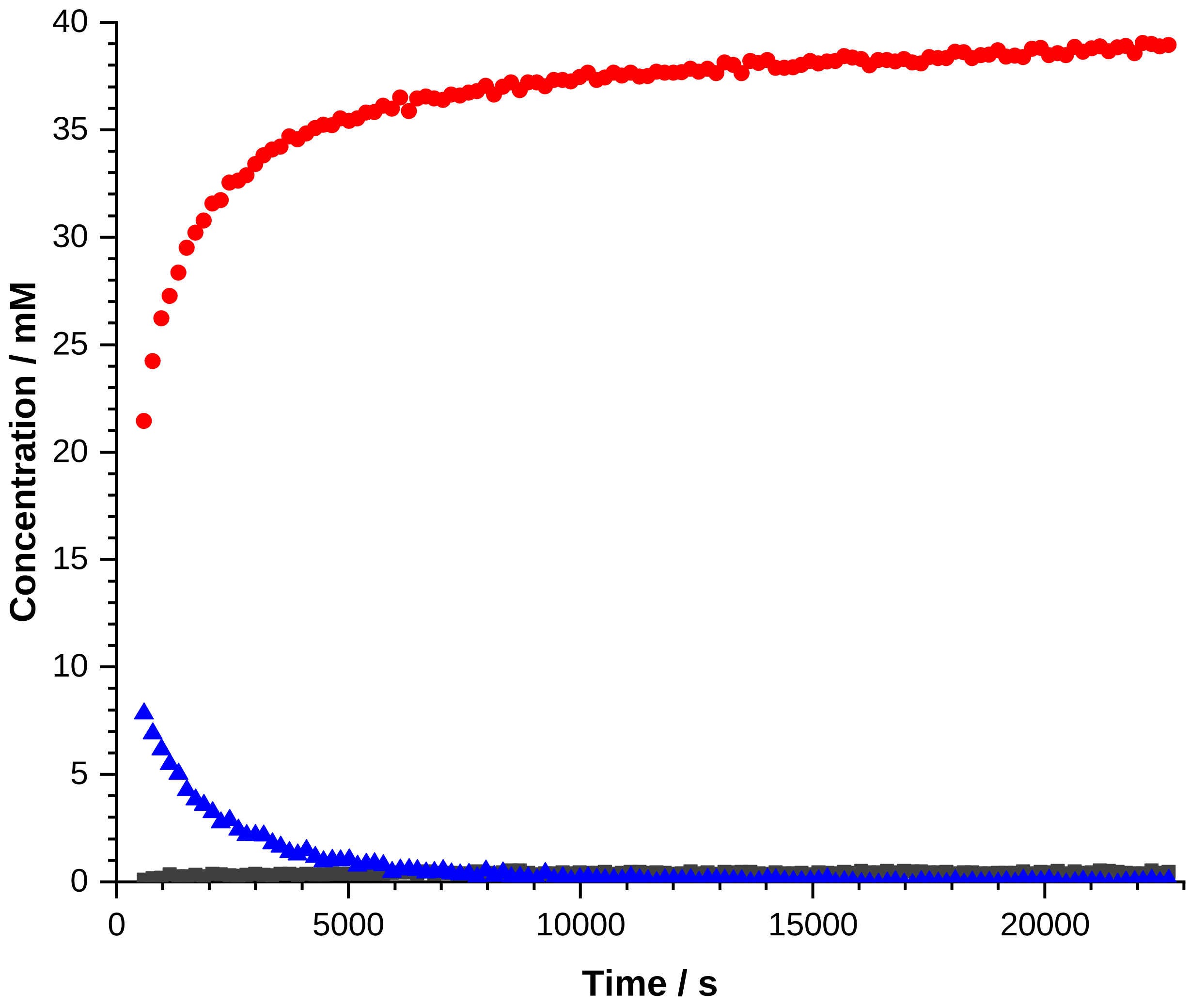

Figure 57: Concentration vs. time plot for the activation of [Pd(P^C)(μ_2-OH)]$_2$ palladacycle 2 using excess arylboronic acid 9 (10 equiv.) in THF. Concentrations calculated from an octafluoronapthelene internal standard. Key: ▲ Pd transmetallation complex 13; ● fluorobenzene 6; ■ 4,4'-difluorobiphenyl 10.

Table 11: Fitted rate constants for Run 4 from concentration vs. time plot.

	Peak at -117.2 (10)	Peak at – 114.4 (6)	Peak at -123.7 (13)
**Rate constant / s^{-1}**	4.061 $\times 10^{-4}$	4.079 $\times 10^{-4}$	-5.277 $\times 10^{-4}$
**Error / s^{-1}**	0.571 $\times 10^{-4}$	0.142 $\times 10^{-4}$	0.087 $\times 10^{-4}$

Table 12: Data for Run 4 concentration vs. time plot

Time / s	Peak at -117.2 (10) / mM	Peak at – 114.4 (6) / mM	Peak at -123.7 (13) / mM
**600**	0.083491	21.42943	7.832831
**787**	0.151805	24.22993	6.902381
**971**	0.181406	26.21133	6.1592
**1155**	0.328426	27.2537	5.486894
**1338**	0.256552	28.34313	5.030622
**1522**	0.245929	29.49332	4.261248
**1706**	0.320306	30.2087	3.823109
**1890**	0.25392	30.77351	3.568044
**2074**	0.35328	31.55548	3.235294
**2258**	0.3314	31.71324	2.765801
**2442**	0.293324	32.53733	2.87612
**2626**	0.277386	32.61071	2.426502
**2810**	0.312686	32.87607	2.183618
**2994**	0.358139	33.39697	2.178782
**3178**	0.310712	33.78939	2.14055
**3362**	0.293253	34.06582	1.793538
**3546**	0.371249	34.20746	1.61998
**3729**	0.362951	34.67006	1.374773
**3913**	0.319687	34.54822	1.268744
**4097**	0.360484	34.82454	1.481136
**4281**	0.346825	35.07225	1.147597
**4465**	0.343019	35.21645	0.947414
**4649**	0.393203	35.20063	1.02723
**4833**	0.37141	35.52638	0.988435
**5017**	0.354507	35.40226	1.042051
**5201**	0.362979	35.52675	0.750501
**5385**	0.35637	35.79552	0.847245
**5569**	0.366454	35.80089	0.857587
**5753**	0.394281	36.104	0.762036
**5936**	0.411608	35.97092	0.461678
**6120**	0.438828	36.48396	0.564115
**6304**	0.426958	35.85428	0.583196
**6488**	0.387052	36.43562	0.554277
**6672**	0.471937	36.52669	0.437461
**6856**	0.353681	36.43906	0.46273

**7040**	0.412222	36.36677	0.533658
**7224**	0.397613	36.6313	0.385465
**7408**	0.367421	36.57864	0.332952
**7592**	0.383325	36.72146	0.355417
**7776**	0.467846	36.78291	0.207349
**7960**	0.39841	37.03096	0.521072
**8143**	0.385225	36.62238	0.261682
**8327**	0.431756	36.99204	0.422471
**8511**	0.531138	37.19852	0.20039
**8695**	0.515834	36.83691	0.229723
**8879**	0.398713	37.18377	0.206707
**9063**	0.335443	37.19678	0.110185
**9247**	0.384738	37.01151	0.412597
**9431**	0.375935	37.29562	0.120526
**9615**	0.436283	37.29562	0.205979
**9799**	0.394498	37.24717	0.074108
**9983**	0.42618	37.44807	0.149709
**10166**	0.415839	37.63674	0.195844
**10350**	0.396747	37.29966	0.138214
**10534**	0.460166	37.40977	0.120227
**10718**	0.371474	37.65127	0.123543
**10902**	0.410694	37.511	0.100059
**11086**	0.460487	37.6496	0.219627
**11270**	0.454696	37.47457	0.109934
**11454**	0.406305	37.48659	0.059121
**11638**	0.426403	37.69201	-0.01348
**11822**	0.415416	37.63445	0.102503
**12006**	0.368158	37.63789	0.11867
**12190**	0.394551	37.66056	0.081594
**12374**	0.465052	37.81877	0.111101
**12558**	0.39349	37.69989	-0.00825
**12742**	0.426443	37.82075	0.133682
**12926**	0.358038	37.63009	0.103885
**13110**	0.457204	38.1148	0.095201
**13293**	0.42222	37.99943	0.059909
**13477**	0.452201	37.6269	0.06411
**13661**	0.446268	38.17954	-0.02155
**13845**	0.374902	38.08717	0.014457
**14029**	0.368195	38.22556	0.137935
**14213**	0.437376	37.87461	0.122914
**14397**	0.395325	37.87593	0.039415
**14581**	0.378233	37.88731	0.013578
**14765**	0.398197	38.00989	0.015729

**14949**	0.345094	38.18299	0.04661
**15133**	0.4259	38.07732	0.067146
**15317**	0.39725	38.16221	0.165609
**15500**	0.39103	38.19802	-0.04614
**15684**	0.444982	38.41625	0.012407
**15868**	0.404312	38.34556	-0.00935
**16052**	0.508648	38.27661	-0.06838
**16236**	0.429642	37.99261	-0.04559
**16420**	0.428444	38.22547	-0.09334
**16604**	0.493083	38.23191	-0.03736
**16788**	0.425622	38.16431	0.016982
**16972**	0.495783	38.27444	-0.08906
**17156**	0.472179	38.11195	-0.12142
**17340**	0.471199	38.07101	0.012481
**17524**	0.427423	38.37685	0.033894
**17707**	0.422084	38.33241	-0.05089
**17891**	0.445	38.32958	-0.07263
**18075**	0.384254	38.62171	0.063353
**18259**	0.431309	38.59538	-0.08343
**18443**	0.421005	38.32781	-0.0049
**18627**	0.386075	38.44962	-0.01728
**18811**	0.3771	38.47314	0.002745
**18995**	0.396373	38.68106	-0.04881
**19179**	0.400617	38.3866	0.014139
**19363**	0.410506	38.44066	-0.05474
**19547**	0.482789	38.3602	0.085693
**19731**	0.387377	38.7619	0.049497
**19915**	0.450927	38.80196	0.031834
**20098**	0.421211	38.45897	0.089829
**20282**	0.498287	38.5484	-0.02196
**20466**	0.377195	38.4608	-0.10052
**20650**	0.468697	38.83849	-0.03041
**20834**	0.413681	38.60785	0.043307
**21018**	0.434792	38.77623	0.004578
**21202**	0.51321	38.86995	0.00585
**21386**	0.486766	38.64869	-0.05938
**21570**	0.44457	38.8188	-0.08183
**21754**	0.417908	38.89141	-0.00408
**21938**	0.385952	38.54955	0.031806
**22122**	0.383136	39.0153	0.017843
**22306**	0.511689	38.97404	0.08519
**22489**	0.414328	38.8634	-0.02675
**22673**	0.447455	38.92793	0.088586

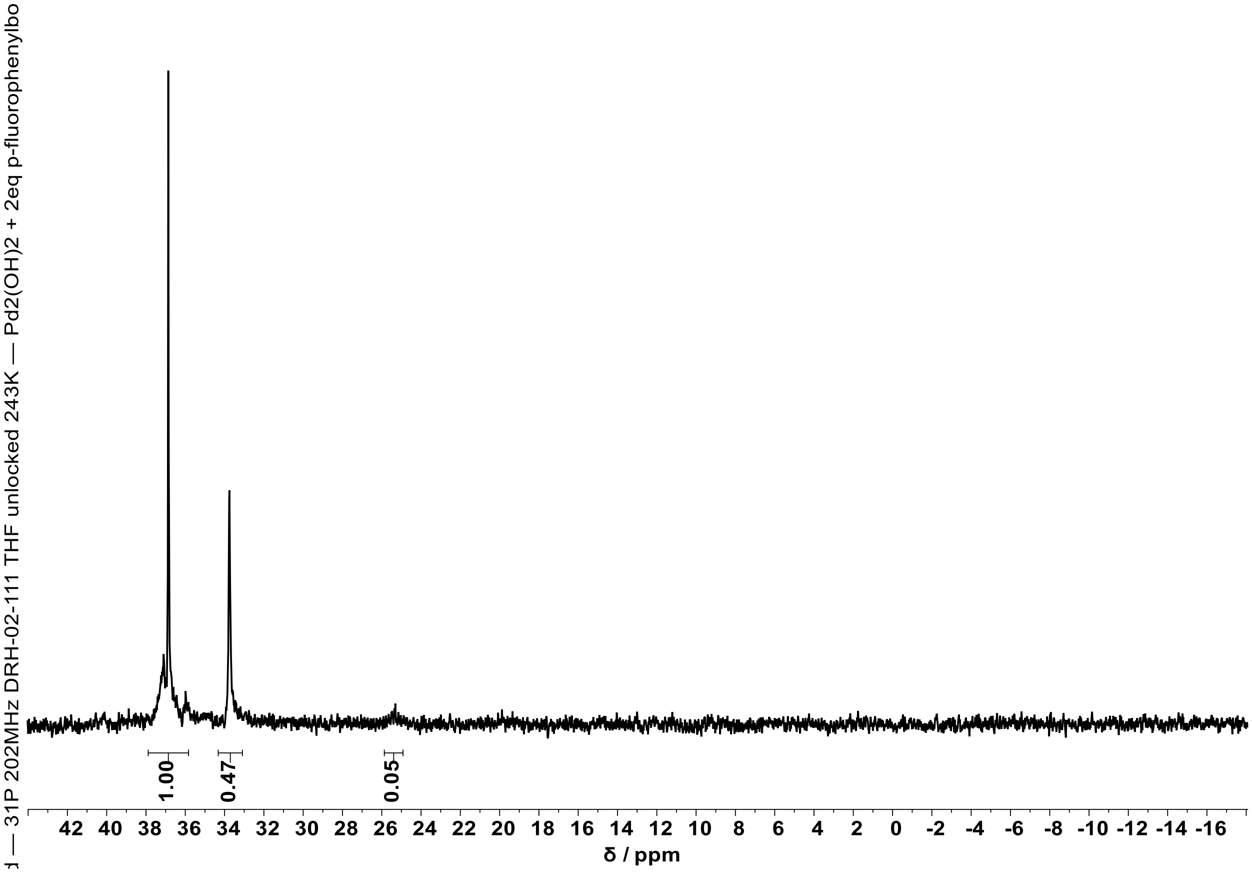

Figure 58: ^{31}P NMR (202 MHz, THF-unlocked, 243 K) spectrum after the kinetic run in the presence of excess arylboronic acid 9. Lab book ref. DRH-02-111.

7. Stoichiometric [Pd(P^C)(μ_2-OH)]$_2$ Palladacycle 2 Activation Experiments

7.1 Activation of [Pd(P^C)(μ_2-OH)]$_2$ Palladacycle 2 by Polar Solvents

[Pd(P^C)(μ_2-OH)]$_2$ palladacycle **2** (5 mg, 0.0059 mmol) was placed into a J-Young tap NMR tube and the vessel was evacuated/backfilled 3 times with N_2. Anhydrous degassed solvent (0.5 mL) was added, and the vessel was sealed under N_2. The solution was sonicated for 2 min to aid dissolution before analysis by NMR spectroscopy.

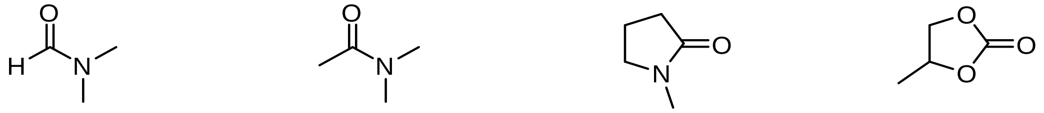

N,N-dimethylformamide *(DMF)* **12.7**

N,N-dimethylacetamide *(DMA)* **12.4**

N-methyl-2-pyrrolidone *(NMP)* **13.6**

propylene carbonate *(PC)* **16.5**

Scheme 3: Structures of solvents commonly used in cross-coupling reactions, here used for pre-catalyst activation, with their associated dipole moment (in Coulombmeter, Cm). Values from Sneddon *et al*.[13]

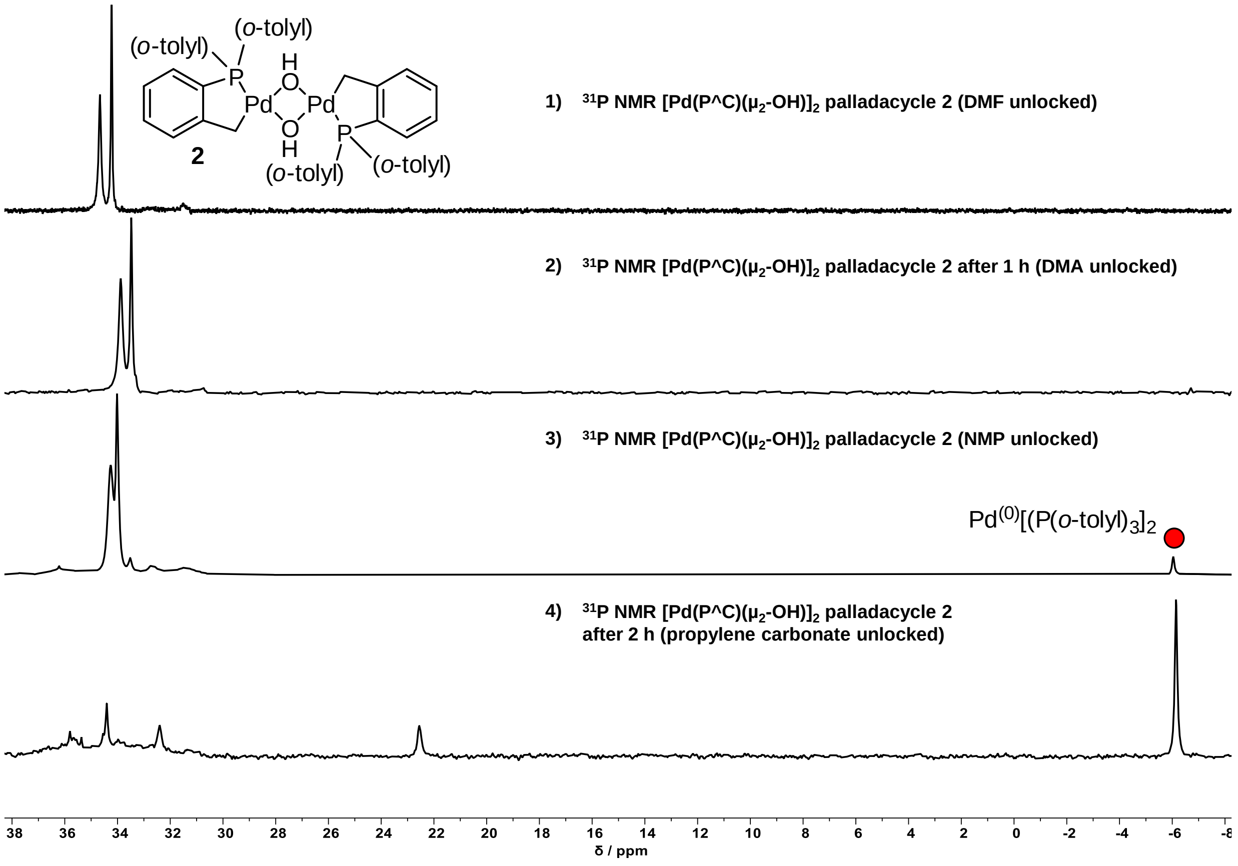

Figure 59: ^{31}P NMR spectra showing the activation of [Pd(P^C)(μ_2-OH)]$_2$ palladacycle 2 in different solvents; 1) DMF (lab book ref. DRH-02-28), 2) DMA (lab book ref. DRH-02-38-2), 3) NMP (lab book ref. DRH-01-128-5), 4) propylene carbonate (lab book ref. DRH-02-38)

It was determined that $[Pd(P^\wedge C)(\mu_2\text{-}OH)]_2$ palladacycle **2** activates to $Pd^{(0)}[P(o\text{-tolyl})_3]_2$ in certain polar solvents. This effect was solvent dependent, with **2** stable in DMF for several hours. DMF is known to decompose and form trace $HNMe_2$ and CO over time (especially at elevated temperatures),[14] but the effects of these impurities are not apparent here. Changing to DMA gave rise to a very small peak at ^{31}P δ -6.5 ppm, which is diagnostic of $Pd^{(0)}[P(o\text{-tolyl})_3]_2$. This peak became more pronounced in NMP and was concurrent with a color change of the sample from pale yellow to brown. This indicates that aggregation to PdNPs and larger aggregates occurs in NMP, in line with polar solvents stabilizing PdNPs.[15] In propylene carbonate, this activation is almost immediate and total, with little **2** remaining after 2 h. It should be noted that these processes in neat solvent are relatively slow compared to the speed of SMCCs, so the mode of activation under reaction conditions probably does not involve this pathway.

7.2 Reaction Endpoint

A ^{31}P NMR of a reaction endpoint was taken for a cross-coupling employing $[Pd(P^\wedge C)(\mu_2\text{-}OH)]_2$ palladacycle **2** (General Procedure 2.2, lab book ref. DRH-02-1-1). The peak at ^{31}P δ 26.5 ppm was determined to be $Pd^{(II)}$ bipalladacyclic species **11**. Tri(*o*-tolyl)phosphine oxide was proposed to be the peak at ^{31}P δ 42 ppm, but an authentic sample in the reaction solvent suggested otherwise. There was a positive match for $[Pd(P^\wedge C)(\mu_2\text{-}Br)]_2$ palladacycle **35**, the identity of which was confirmed by comparing the NMR of an authentic sample.

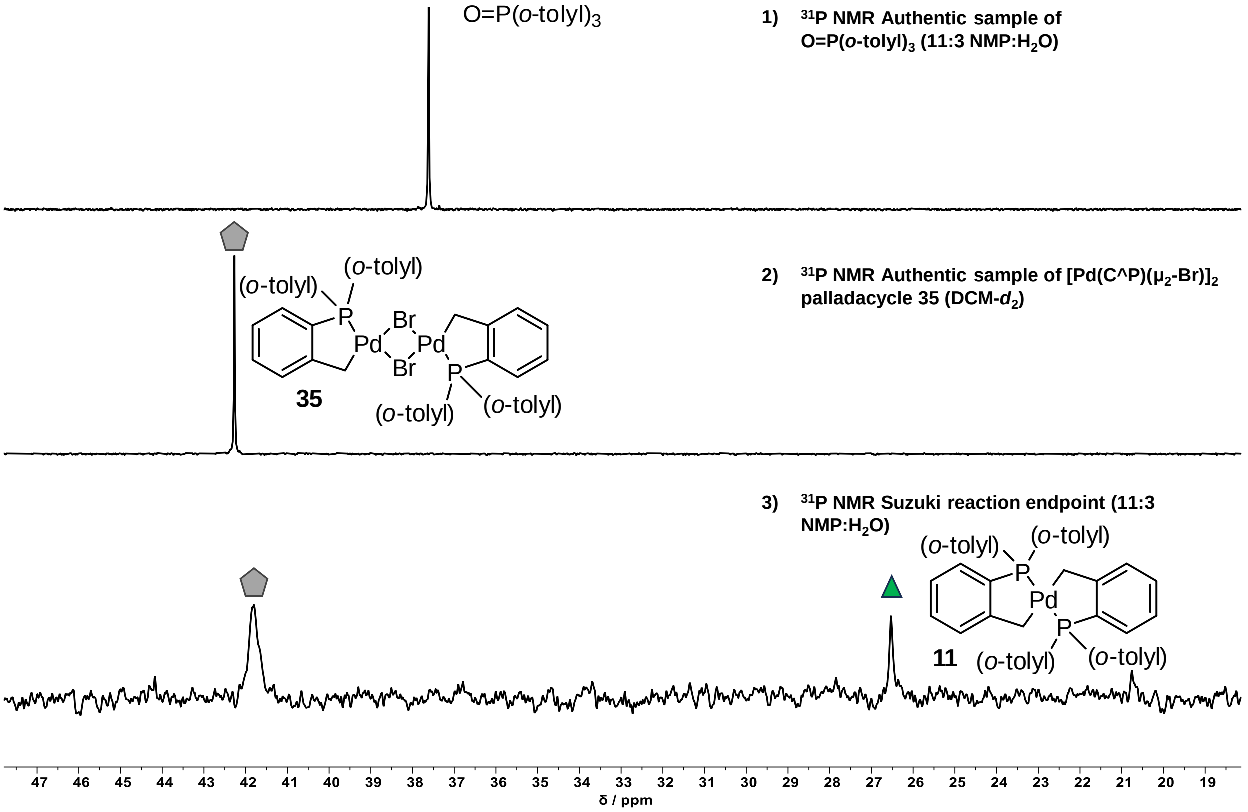

Figure 60: ^{31}P NMR (243 MHz, 298K) stacked spectra showing the reaction endpoint of a standard Suzuki reaction (Lab book ref. DRH-02-1-1) and potential identities of peaks with authentic samples. O=P(o-tolyl)$_3$ lab book ref. DRH-02-8-3, $Pd_2(\mu_2\text{-}Br)_2$ palladacycle 35 lab book ref. DRH-02-8. Integrals of 3): 35 = 2.39, 11 = 1.00.

7.3 Trapping Pd$^{(0)}$ with Excess Phosphine

[Pd(P^C)(μ_2-OH)]$_2$ palladacycle **2** (5 mg, 0.00586 mmol, 1 eq.) and 4-fluorophenylboronic acid **9** (1.6 mg, 0.0117 mmol, 2 eq.) were added to a J-Young tap NMR tube and evacuated and backfilled with N_2. The tube was cooled to -78 °C, and THF (0.5 mL, dry, degassed) was added, and the reaction mixture was shaken vigorously to aid dissolution. The sample was placed into a pre-cooled NMR spectrometer (203 K) and analyzed by NMR spectroscopy at different temperatures.

For Figure 64 **2)**, P(*o*-tolyl)$_3$ (3.6 mg, 0.0117 mmol, 2 eq.) was added prior to addition of THF, and the reaction was done at room temperature.

Lab book ref. DRH-02-98, DRH-02-189

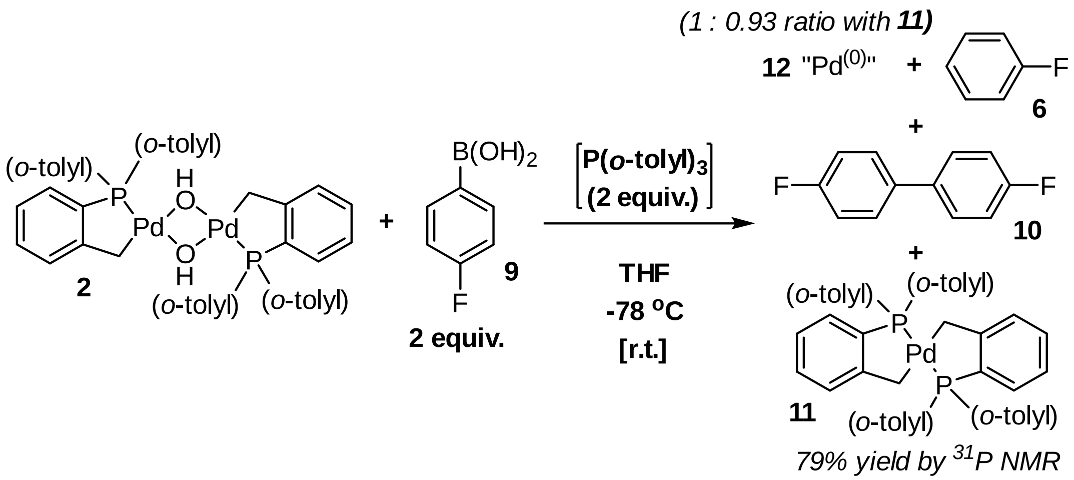

Scheme 4: Activation of the $Pd_2(\mu_2\text{-}OH)_2$ palladacycle by boronic acid in the presence and absence of P(*o*-tolyl)$_3$ for $Pd^{(0)}$ species trapping

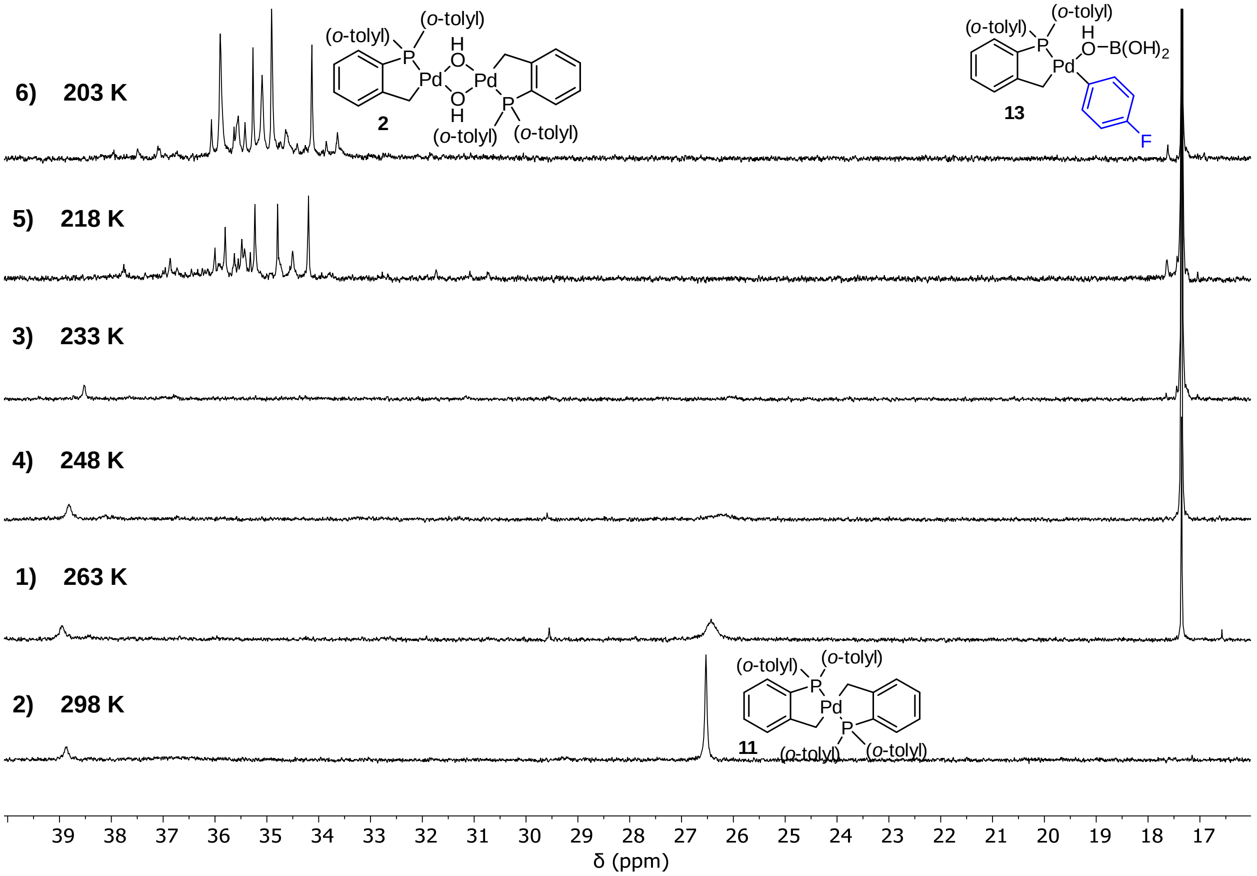

Figure 61: ^{31}P NMR (202 MHz, THF-unlocked) of the reaction of 2 with 2 equiv. of 4-fluorophenylboronic acid 9 at different temperatures. The peak at δ 17.4 is proposed to be transmetallation complex 13, and the peaks in the

region δ 34 – 36 ppm belong to 2 (dimer and encounter complex with boronic acid). Lab book ref. DRH-02-98 (2, 8, 12, 18, 22, 26). The peak at δ 39 ppm is proposed to be O=P(*o*-tolyl)₃.

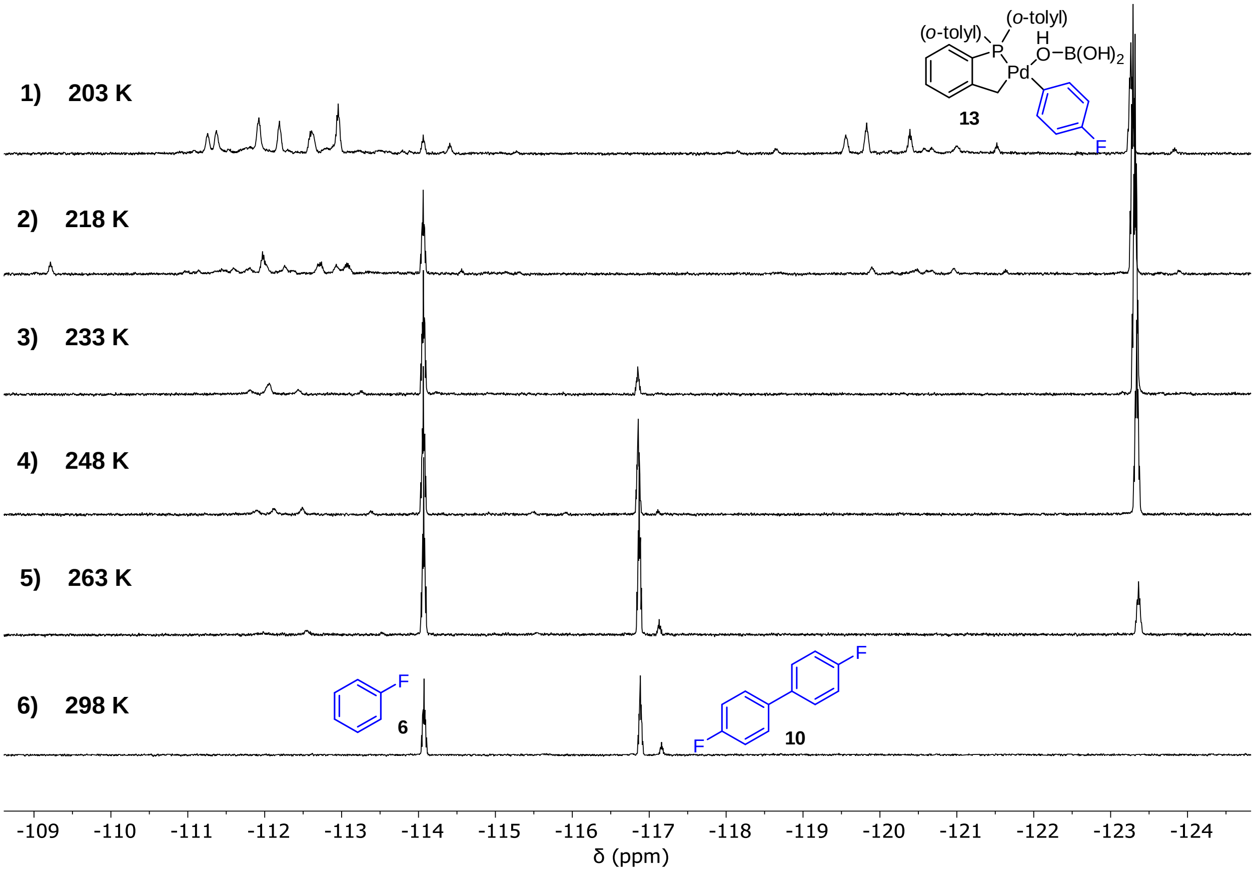

Figure 62: ^{19}F NMR (470 MHz, THF-unlocked) of the reaction of 2 with 2 equiv. of 4-fluorophenylboronic acid 9 at different temperatures. The peak at δ -123.3 is proposed to be transmetallation complex 13. Lab book ref. DRH-02-98 (4, 10, 16, 20, 24, 28). Integrals in 6): 6 = 1.00, 10 = 1.04.

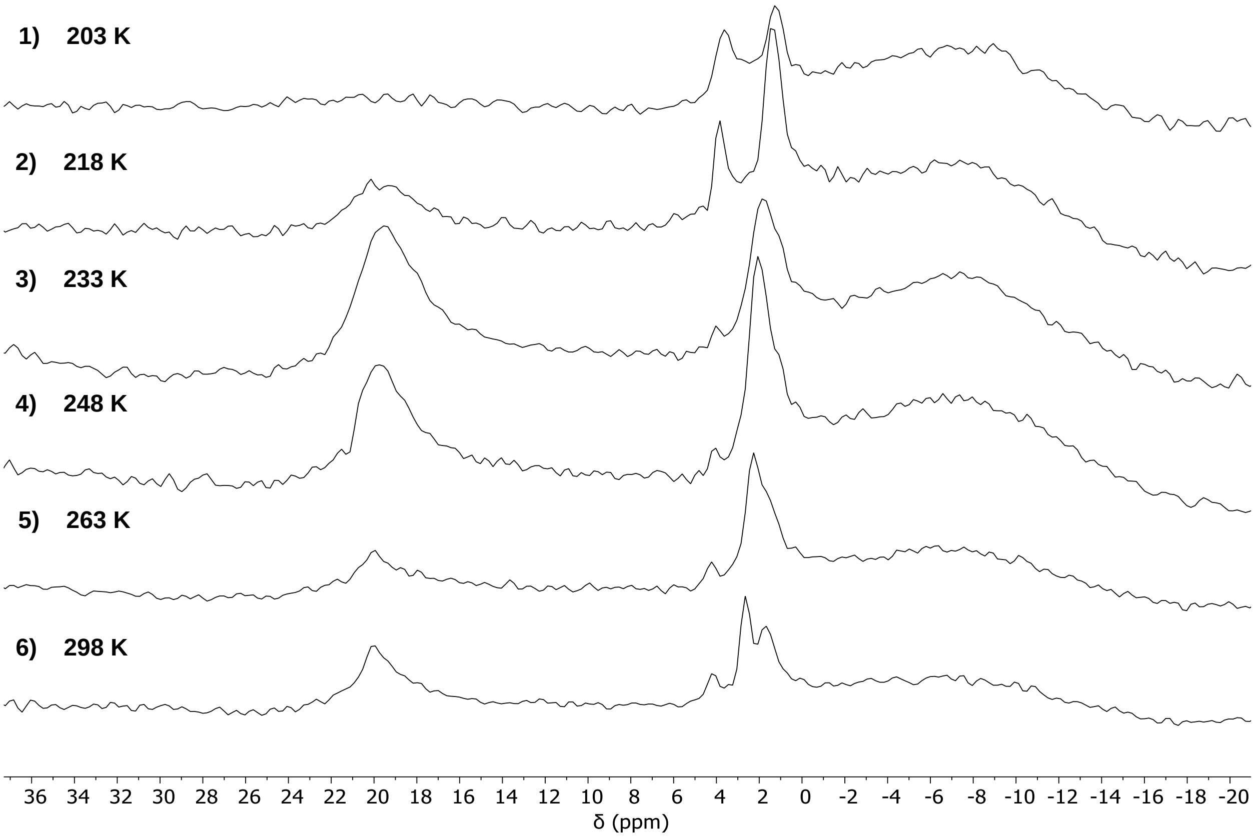

Figure 63: ^{11}B NMR (160 MHz, THF-unlocked) of the reaction of 2 with 2 equiv. of 4-fluorophenylboronic acid 9 at different temperatures. Lab book ref. DRH-02-98 (3, 9, 15, 19, 23, 27).

The proposed transmetallation complex **13** was detected by ^{19}F NMR (δ -123.3 ppm) and ^{31}P NMR (δ 17.4 ppm). These peaks grow in as the temperature increases from 203 K to 233 K, before decaying at the same rate at higher temperatures to form **6** and **10** (by ^{19}F NMR, δ -114.0 and -116.9 ppm) and **11** (by ^{31}P NMR, δ 26.5 ppm). At 203 K, there are many peaks in the ^{31}P (δ 34 – 38 ppm) and ^{19}F (δ -119 – -122 ppm) spectra that are likely from pre-transmetallation intermediates (boronic acid coordinating to the μ_2-OH group of **2**, in both monomer and dimer form) as well as residual peaks for **2**. These peaks disappear at 233 K, all having formed transmetallation complex **13**.

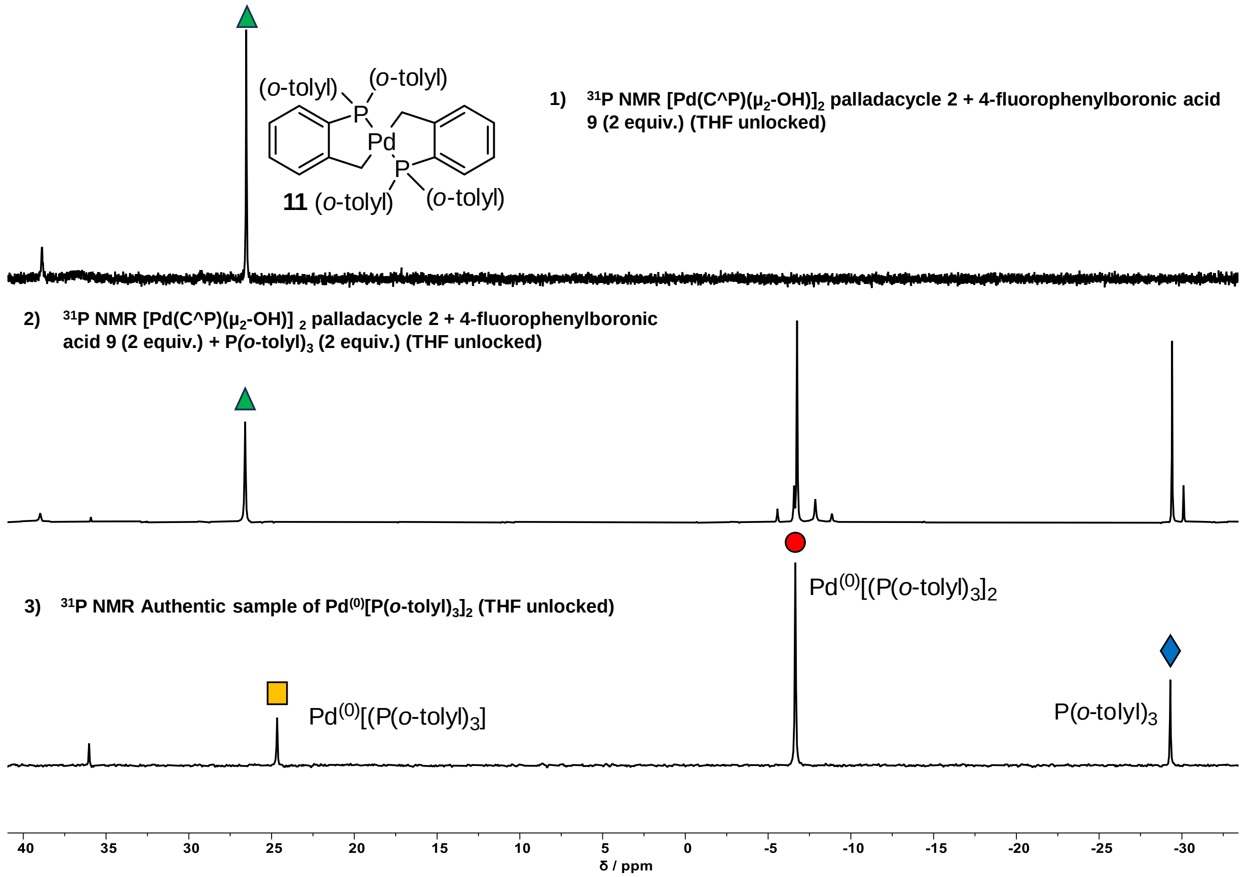

Figure 64: ^{31}P NMR (243 MHz, 298K) stacked spectra showing 1) the identity of the observed [Pd(P^C)] bipalladacycle 11 complex by ^{31}P NMR (Scheme 4, Lab book ref. DRH-02-98), 2) the same reaction in the presence of P(*o*-tolyl)$_3$ to give the Pd$^{(0)}$[P(*o*-tolyl)$_3$]$_2$ complex in solution (Lab book ref. DRH-02-189), 3) an authentic sample of Pd$^{(0)}$[P(o-tolyl)$_3$]$_2$ (Lab book ref. DRH-02-49-1), which dissociates to the monoligated Pd$^{(0)}$[P(*o*-tolyl)$_3$] (14) complex in solution. Integrals of 2): 11 = 1.00, Pd$^{(0)}$[P(*o*-tolyl)$_3$]$_2$ = 1.72, P(*o*-tolyl)$_3$ = 0.77.

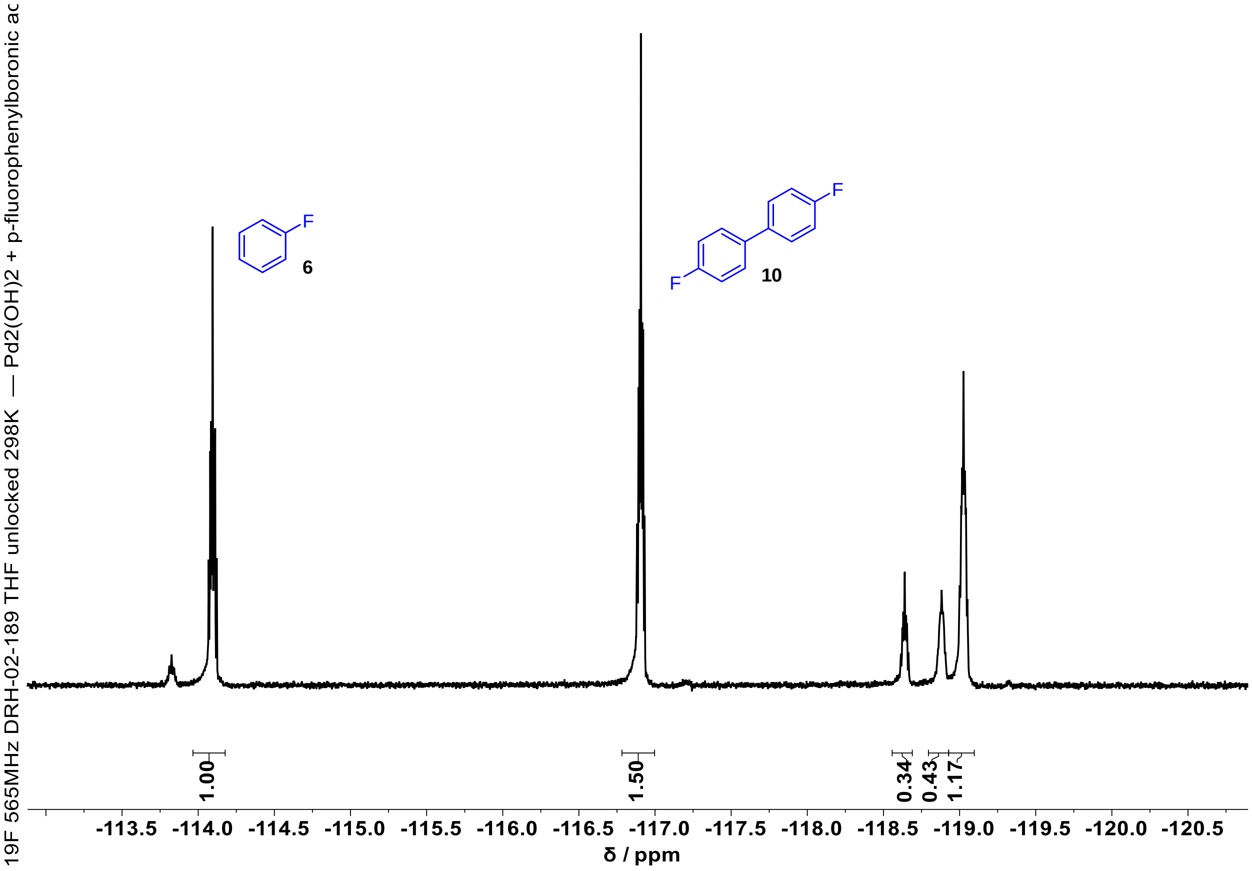

Figure 65: ^{19}F NMR (565 MHz, THF-unlocked, 298K) spectra of the reaction between palladacycle 2 and 4-fluorophenylboronic acid 9 in the presence of excess P(*o*-tolyl)$_3$. The peaks at δ -118.5 – -119.5 are likely from a side reaction where boronic acid aryl groups that have eliminated onto the phosphine ligand during activation. Lab book ref. DRH-02-189.

7.4 Stoichiometric Cross-Coupling Reaction using [Pd(P^C)(μ_2-OH)]$_2$ Palladacycle 2 with Arylboronic Acid 9

[Pd(P^C)(μ_2-OH)]$_2$ palladacycle **2** (5 mg, 0.0059 mmol, 1 eq.), 4-fluorophenylboronic acid **9** (3 mg, 0.0214 mmol, 4 equiv.) and 4-fluorobromobenzene **3** (13 µL, 0.118 mmol, 20 equiv.) were added to a J-Young tap NMR tube under N_2. DCM-*d*$_2$ (0.5 mL, dry, degassed) was added and the vessel was sealed under N_2 and shaken to ensure dissolution (resulting in a rapid color change from colorless to dark red to yellow in ~ 2 s). The sample was immediately analyzed by NMR spectroscopy. Lab book ref. DRH-02-55.

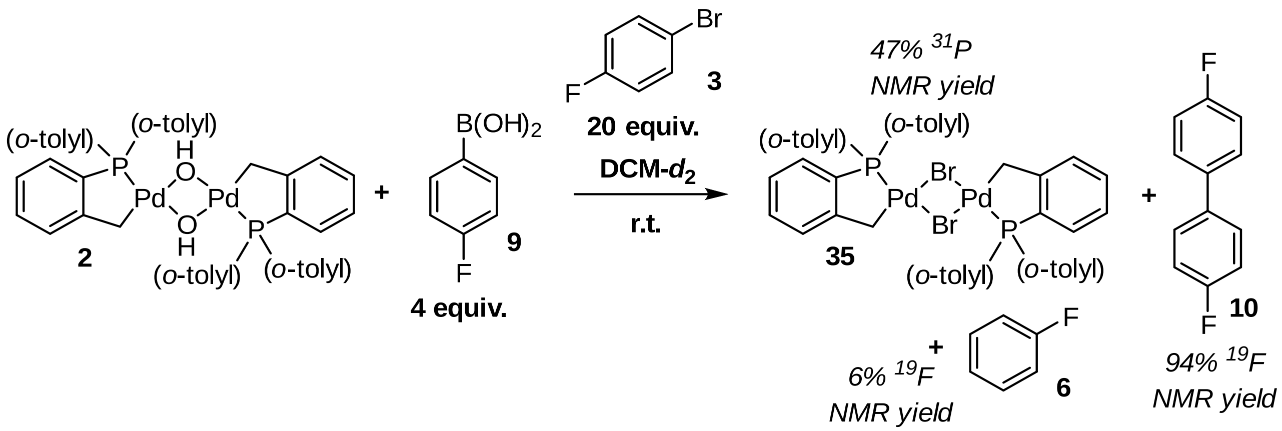

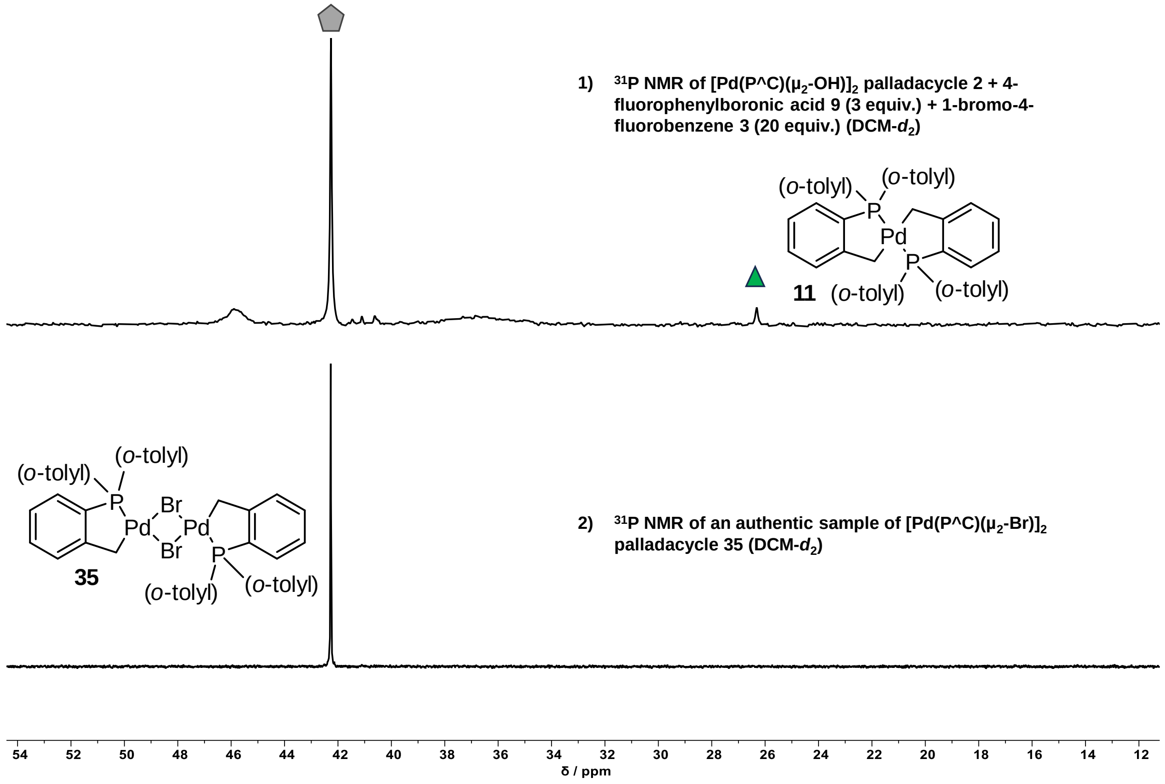

Figure 66: ^{31}**P NMR (243 MHz, 298K) stacked spectra showing the conversion of [Pd(P^C)(μ$_2$-OH)]$_2$ palladacycle 2 to [Pd(P^C)(μ$_2$-Br)]$_2$ palladacycle 35 in the presence of 9 and 3. (lab book ref. DRH-02-55). Integrals of 1): δ 46 ppm = 1.00, 35 = 2.21, 38-35 ppm = 1.35, 11 = 0.17.**

7.5 Stoichiometric Cross-Coupling Reaction using [Pd(P^C)(μ_2-OH)]$_2$ Palladacycle 2 using Arylboronic Acid 4

[Pd(P^C)(μ_2-OH)]$_2$ palladacycle **2** (5 mg, 0.0059 mmol, 1 eq.), deuterated 4-methoxyphenylboronic acid **4** (3 mg, 0.019 mmol, 3.3 equiv., generated from **4** recrystallized from refluxing D_2O) and 4-fluorobromobenzene **3** (6.5 µL, 0.059 mmol, 10 equiv.) were added to a J-Young tap NMR tube under N_2. DCM-d_2 (0.5 mL, dry, degassed) was added and the vessel was sealed under N_2 and shaken to ensure dissolution (resulting in a rapid color change from colorless to dark red to yellow in ~ 2 s). The sample was immediately analyzed by NMR spectroscopy.

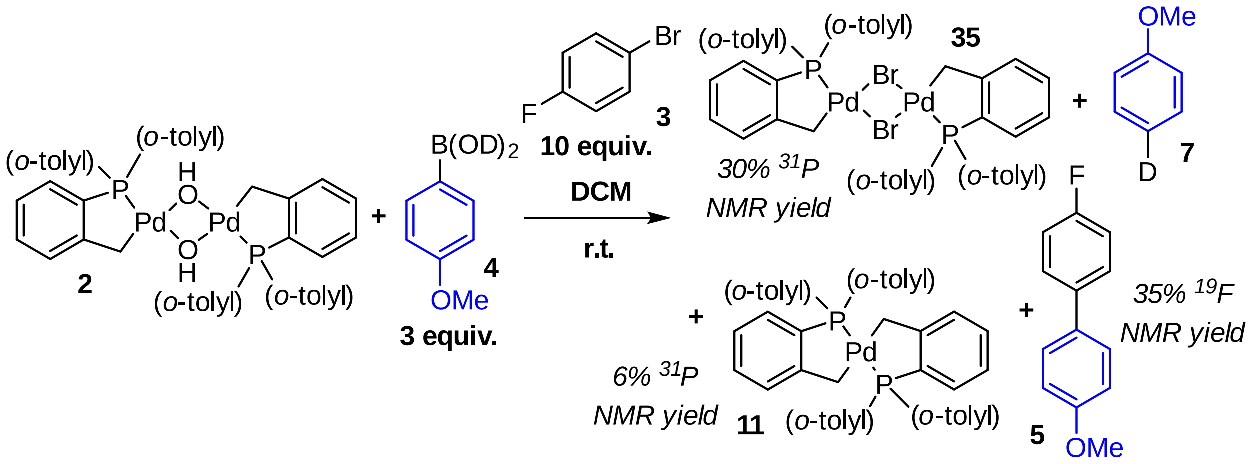

Lab book ref. DRH-03-156

^{31}P NMR spectroscopic analysis revealed that the stable [Pd(P^C)(μ_2-Br)]$_2$ palladacycle **35** was formed rapidly in appreciable quantities. Interestingly, ^{19}F NMR revealed a significant quantity of cross-coupled product **5**, and only trace amounts of fluorobenzene (likely from breaking the palladacycle bond and reformation by reductive elimination.

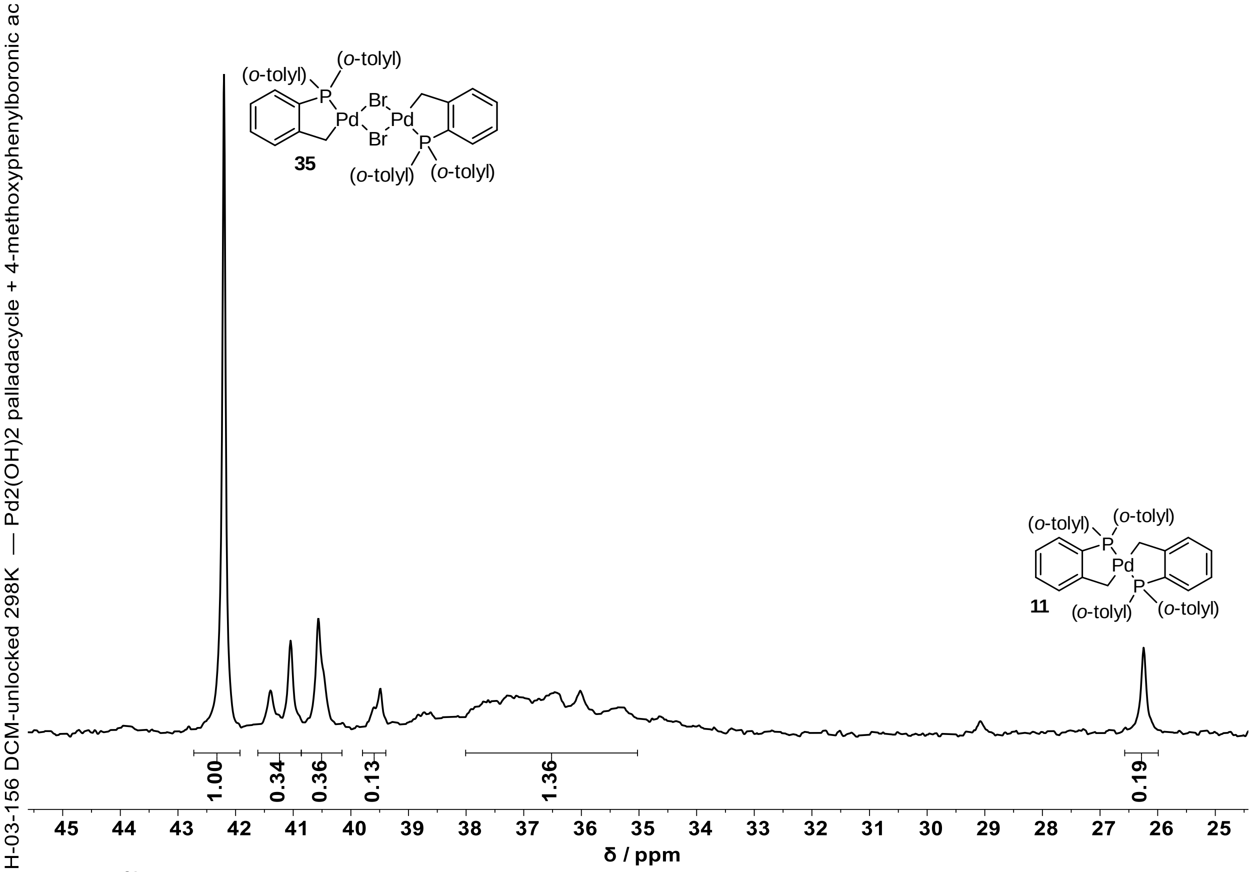

Figure 67: ^{31}**P NMR analysis (243 MHz, DCM-unlocked, 1024 sc, 298 K) following the activation of [Pd(P^C)(μ_2-OH)]$_2$ palladacycle 2 with arylboronic acid 4 in the presence of aryl halide 3 after 24 h (lab book ref. DRH-03-156 (9)).**

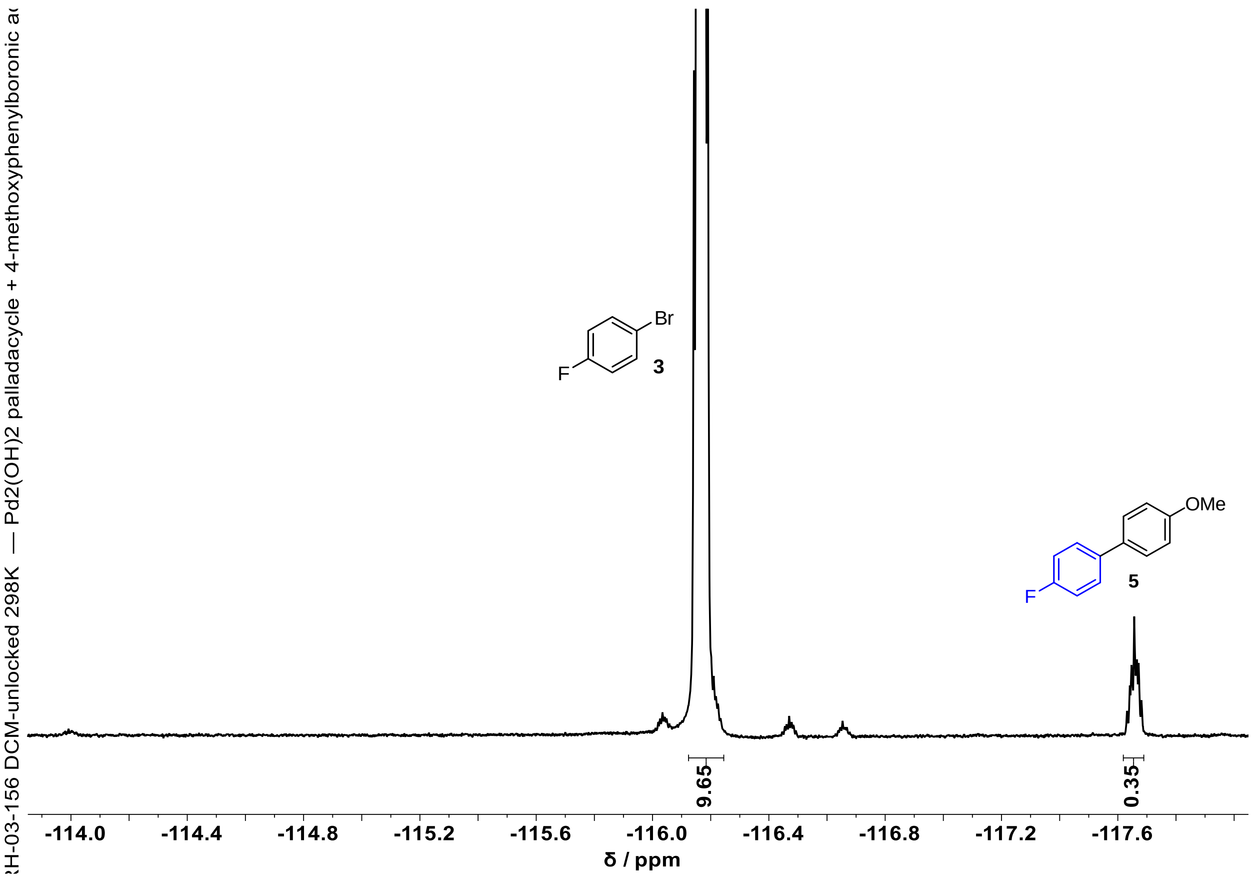

Figure 68: ^{19}F NMR analysis (565 MHz, DCM-unlocked, 256 sc, 298 K) following the activation of [Pd(P^C)(μ_2-OH)]$_2$ palladacycle 2 with arylboronic acid 4 in the presence of aryl halide 3 after 24 h (lab book ref. DRH-03-156 (10)).

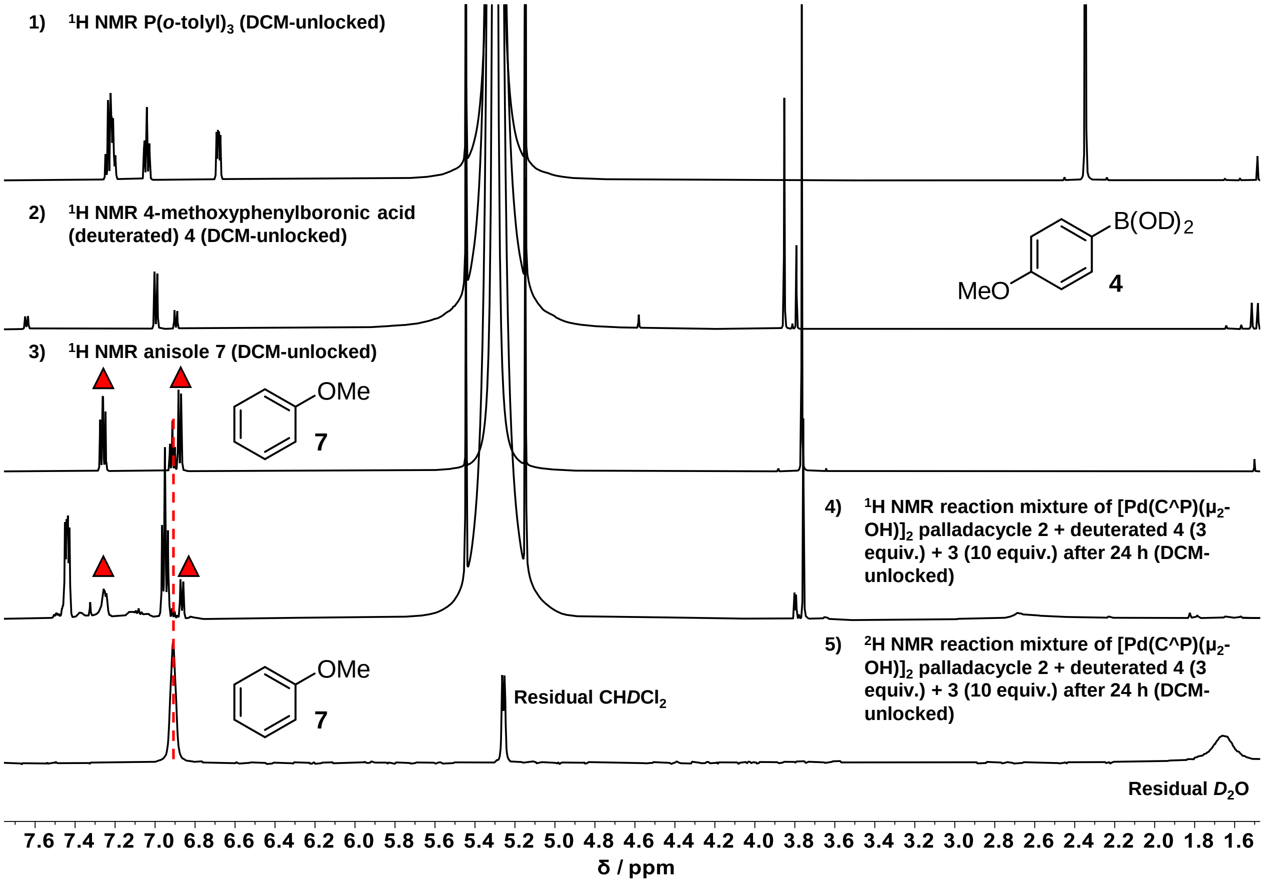

Figure 69: (5) ^{2}H NMR (92 MHz, DCM-unlocked, 38912 sc, 298 K) of the activation of [Pd(P^C)(μ_2-OH)]$_2$ palladacycle 2 with deuterated boronic acid 4 in the presence of aryl halide 3 after 24 h (lab book ref. DRH-03-156). The other spectra are reference standards of potential targets for deuterium incorporation (^{1}H NMR, 600 MHz, DCM-unlocked, 298 K), with (4) being the reaction mixture. The deuterium spectrum is referenced to the same spectrometer frequency as (4) to enable comparison.

7.6 Oxidative Addition to Authentic Pd$^{(0)}$ Complex to form [Pd(μ_2-Br)(C_6H_4F)[P(*o*-tolyl)$_3$]]$_2$ Oxidative Addition Dimer SI45

Pd$^{(0)}$[P(o-tolyl)$_3$]$_2$ (5.0 mg, 0.007 mmol, 1 eq.) was dissolved in THF (0.5 mL, dry, degassed) in a J-Young tap NMR tube under Ar. 1-bromo-4-fluorobenzene **3** (13 µL, 0.12 mmol, 17 eq.) was added under nitrogen, the reaction was sealed and allowed to stand at r.t. for 18 h before NMR analysis.

Lab book ref. DRH-02-49

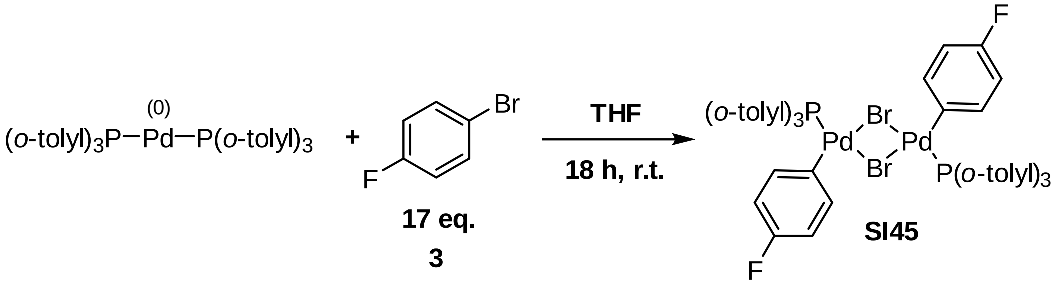

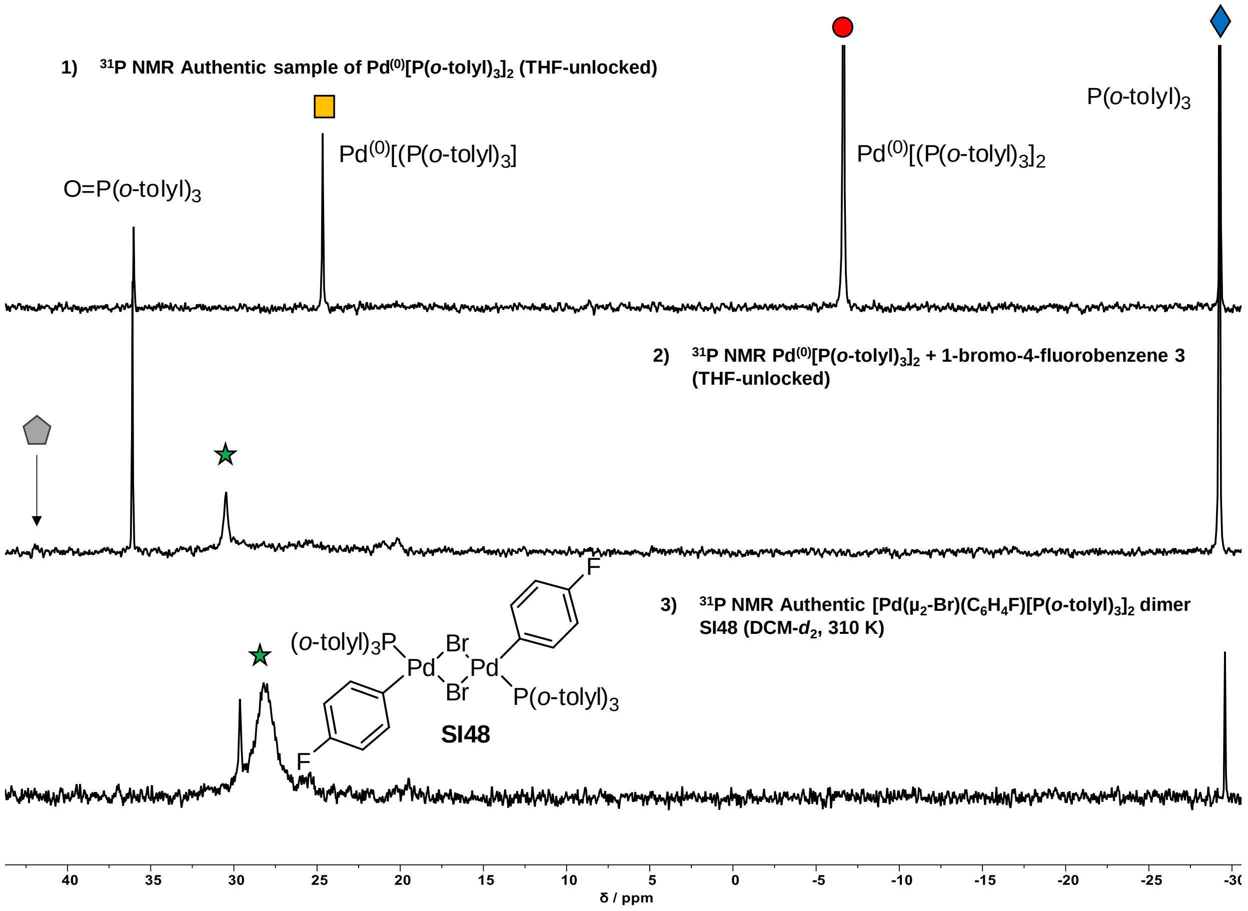

Figure 70: ^{31}**P NMR (243 MHz, 298K) stacked spectra showing the reaction of 1) Pd$^{(0)}$[P(o-tolyl)$_3$]$_2$ (lab book ref. DRH-02-49-1) with 2) an excess of 1-bromo-4-fluorobenzene 3 (lab book ref. DRH-02-49-2) generating the 3) $Pd_2(Br)_2(C_6H_4F)_2$[P(o-tolyl)$_3$]$_2$ oxidative addition dimer SI45, with comparison to an authentic sample (lab book ref. DRH-02-50-1). SI45 exists in solution as cis/trans dimers in equilibrium, so higher temperatures were used to coalesce the peaks for characterization. Key: Blue diamond – P(o-tolyl)$_3$, Red circle – Pd$^{(0)}$[P(o-tolyl)$_3$]$_2$, Orange square – Pd$^{(0)}$[P(o-tolyl)$_3$], Green star – $Pd_2(Br)_2(C_6H_4F)_2$[P(o-tolyl)$_3$]$_2$ dimer SI45, Grey pentagon – [Pd(P^C)(μ_2-Br)]$_2$ palladacycle 35.**

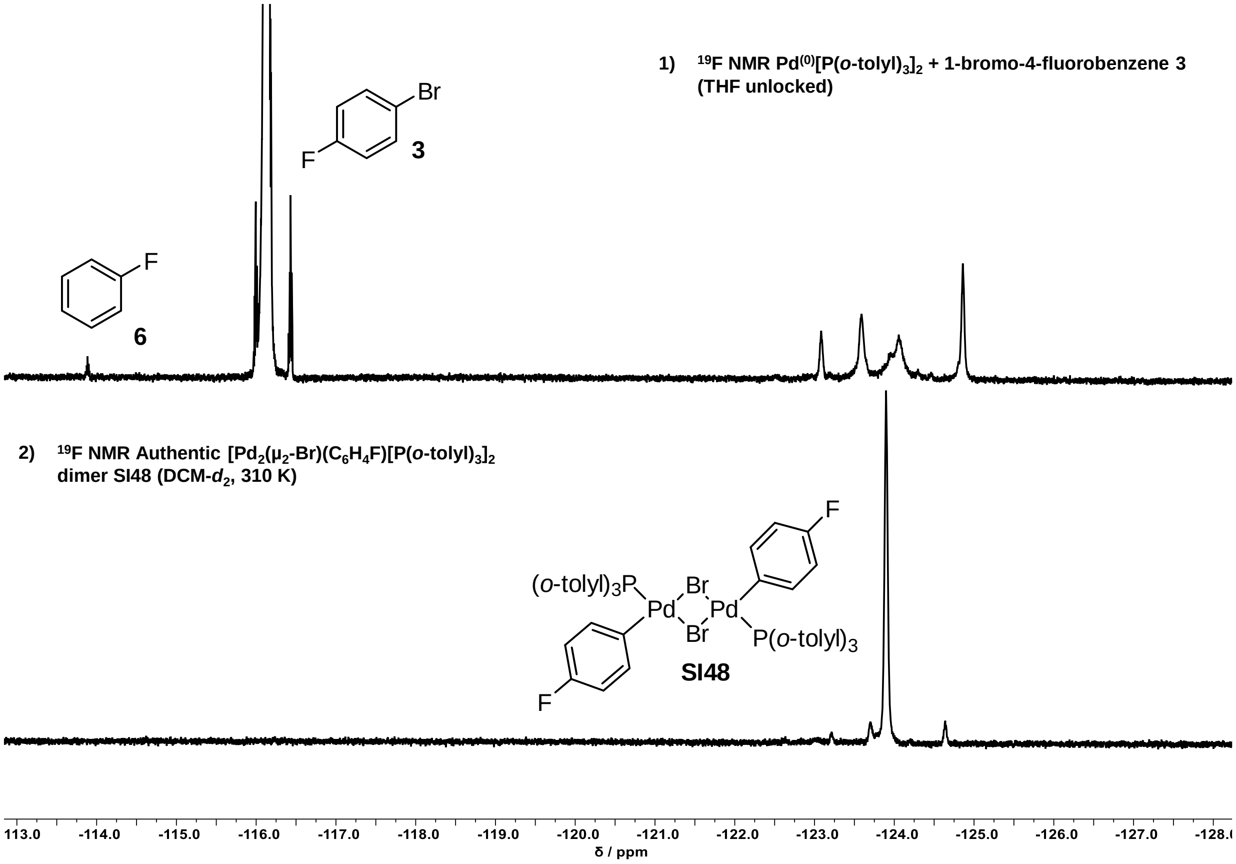

Figure 71: ^{19}F NMR (565 MHz, 298K) stacked spectra showing the reaction of 1) $Pd^{(0)}[P(o\text{-tolyl})_3]_2$ with an excess of 1-bromo-4-fluorobenzene 3 (Lab book ref. DRH-02-49-2), generating 2) the $Pd_2(Br)_2(C_6H_4F)[P(o\text{-tolyl})_3]_2$ oxidative addition dimer SI45, with comparison to an authentic sample. The several peaks in the -123 to -125 ppm region correspond to Pd-Ar-F complexes and are likely from dynamic processes involving the dimer. Fluorobenzene 6 was observed in the reaction mixture.

The ^{19}F NMR spectrum of the same process showed that a small quantity of fluorobenzene was generated in this reaction (Figure 71), which matches with the observation of $[Pd(P^{\wedge}C)(\mu_2\text{-}Br)]_2$ palladacycle **35** from the ^{31}P NMR. Taken with the previous experimental results, this indicates that the oxidative addition $Pd_2(\mu_2\text{-}Br)_2(P(o\text{-tolyl})_3)_2(C_6H_4F)_2$ dimer **SI45** can eliminate fluorobenzene under pseudo-reaction conditions, allowing the reformation of a stable palladacycle. This process has not been observed before in previous studies performed on $Pd_2(\mu_2\text{-}Br)_2(P(o\text{-tolyl})_3)_2(Ar)_2$ complexes,[5] and has significant implications for the overall stability of this class of Pd pre-catalysts. However, it should be emphasized that this is a trace reaction at relatively high concentrations, so the viability of this process under catalytic reaction conditions is questionable. The several peaks in the ^{19}F δ -123 to -125 ppm region correspond to Pd-Ar-F complexes and are likely from dynamic processes involving the dimer.

7.7 Exchange of [Pd(P^C)(μ_2-Br)]$_2$ Bridging Ligand with Base in Solution

To a J-Young tap NMR tube, [Pd(P^C)(μ_2-Br)]$_2$ palladacycle **35** (5 mg, 0.0051 mmol, 1 equiv.) and NMP (0.5 mL, dry, degassed) were added under N_2. The mixture was sonicated for 10 minutes to aid dissolution, but a significant amount of yellow solid remained. 100 µL of (n-Bu)$_4$NOH (1 M in H_2O, degassed, 0.10 mmol, 20 equiv.) was added under N_2, and the resulting mixture was vigorously shaken (5 mins) and sonicated (1 min) to ensure dissolution. The pale yellow/brown solution was then analyzed by NMR spectroscopy.

Lab book ref. DRH-02-37

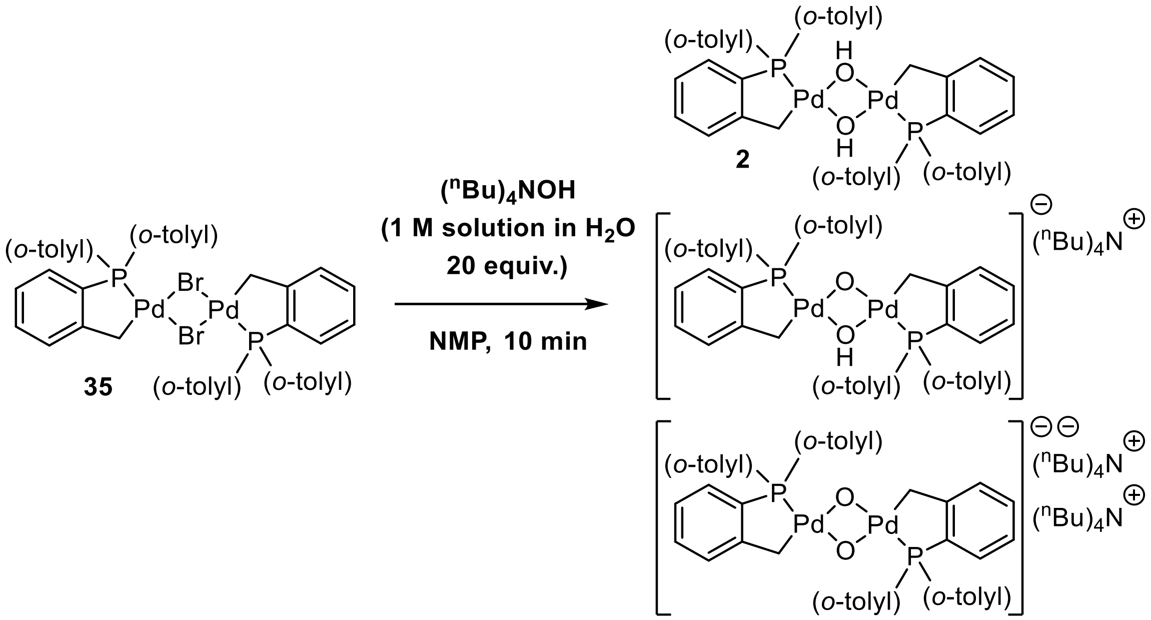

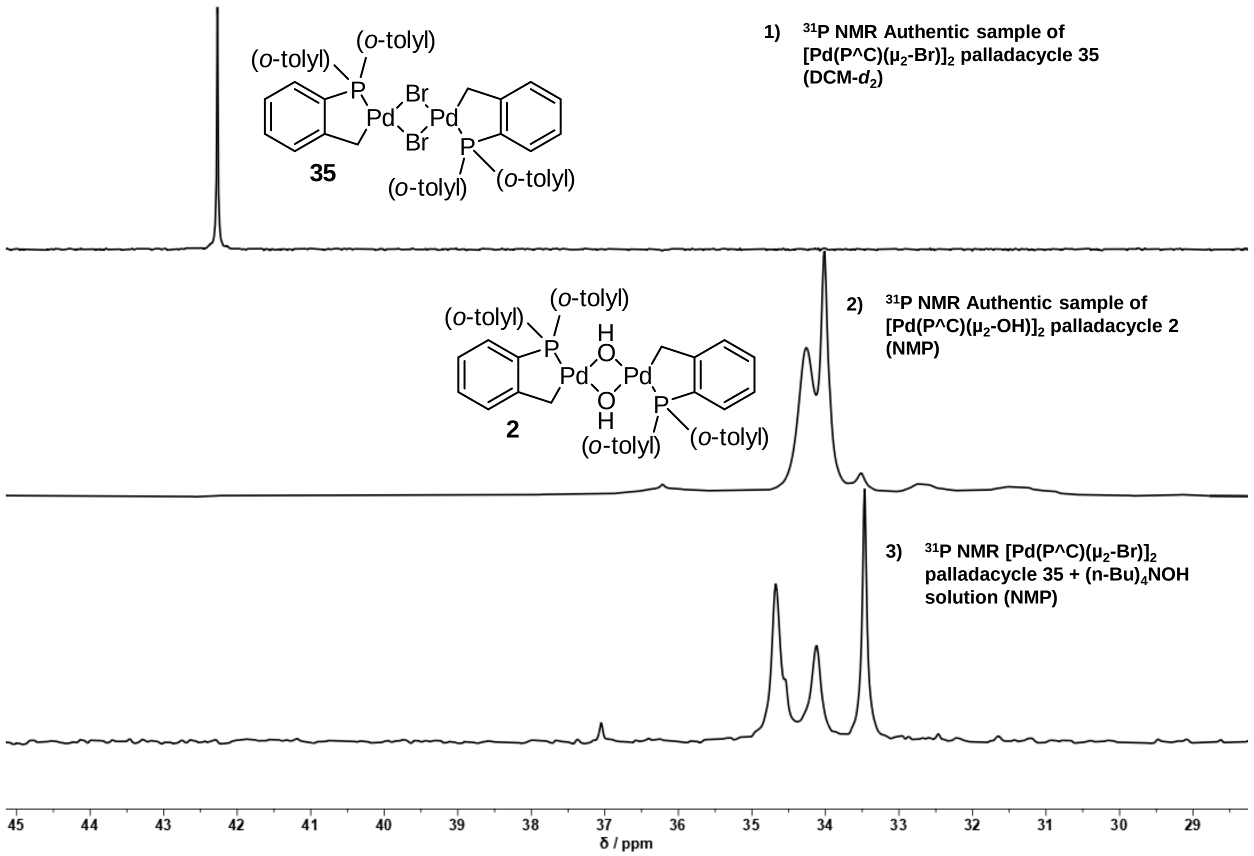

Figure 72: ^{31}P NMR (243 MHz, 298K) stacked spectra showing the reaction of [Pd(P^C)(μ_2-Br)]$_2$ palladacycle 35 with 3) excess n-BuNOH to generate the [Pd(P^C)(μ_2-OH)]$_2$ palladacycle 2 *in situ* (Lab book ref. DRH-02-37). The small change in peak position and identity is likely due to deprotonation of the bridging OH ligand to form ionic complexes. Authentic starting and product complexes are also shown. [Pd(P^C)(μ_2-OH)]$_2$ palladacycle 2 in NMP Lab book ref. DRH-01-128-5.

7.8 Activation of $[Pd(P^\wedge C)(\mu_2\text{-}Ar^F)]_2$ Palladacycle 16a with Excess Water

To a J-Young tap NMR tube in an argon glove box, $[Pd(P^\wedge C)(\mu_2\text{-} Ar^F)]_2$ palladacycle **16a** (5 mg, 0.0043 mmol, 1 equiv.) and benzene-d_6 (0.5 mL, dry, degassed) were added. The solution was sealed, removed from the glovebox and put under a N_2 atmosphere, where water (degassed, 10 µL, 0.56 mmol, 130 equiv.) was added. The vessel was sealed under N_2, shaken to ensure mixing immediately analyzed by NMR spectroscopy.

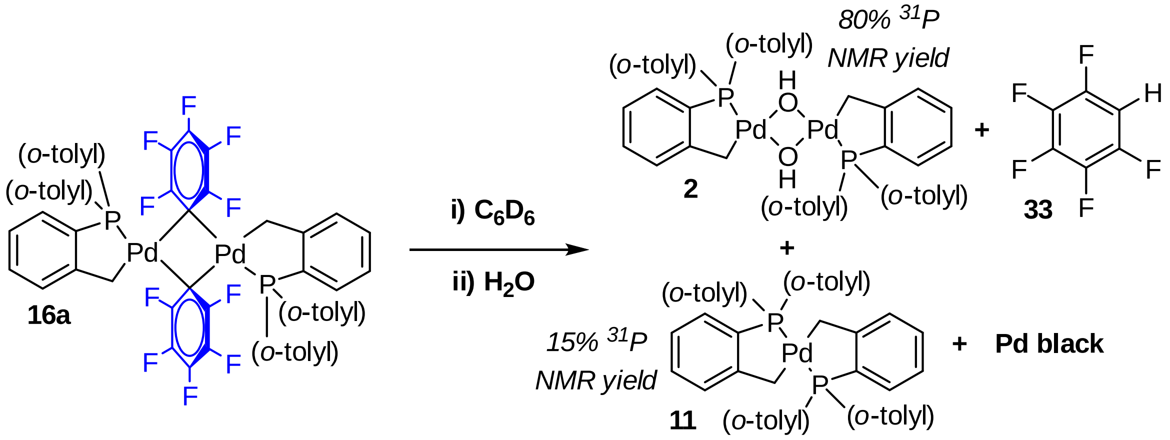

Lab book ref. DRH-03-83-1

$[Pd(P^\wedge C)(\mu_2\text{-} Ar^F)]_2$ palladacycle **16a** was shown to be unstable with respect to water, as the addition of excess water to a sample in C_6D_6 caused rapid degradation and generation of pentafluorobenzene **33**. The ^{31}P NMR showed a mixture of $[Pd(P^\wedge C)(\mu_2\text{-}OH)]_2$ palladacycle **2** and what was likely $Pd^{(II)}$ bipalladacycle **11**, showing that water can either displace the bridging Ar^F ligand entirely or cause activation to $Pd^{(0)}$ *via* palladacycle cleavage.

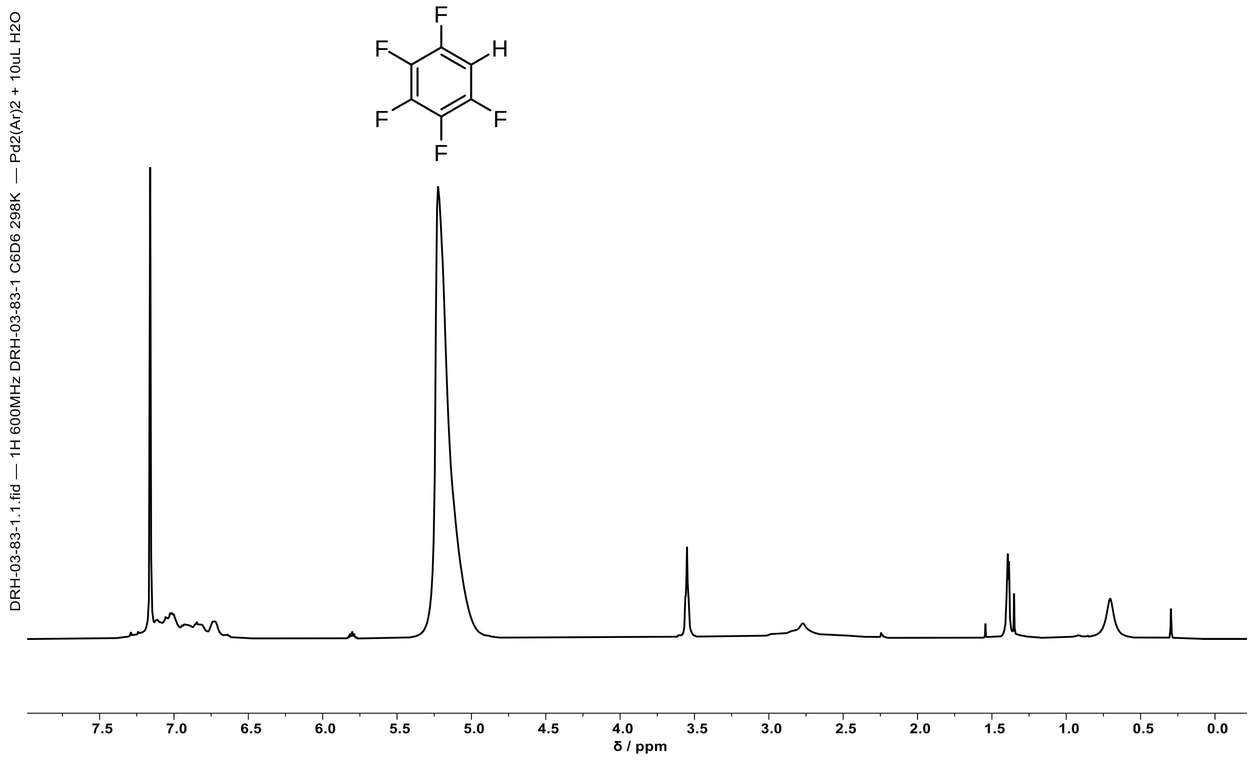

Figure 73: ^{1}H NMR analysis (600 MHz, C_6D_6, 32 sc, 298 K) of the reaction mixture between the [Pd(P^C)(μ_2-Ar^F)]$_2$ palladacycle 16a and an excess of water. Lab book ref. DRH-03-83-1

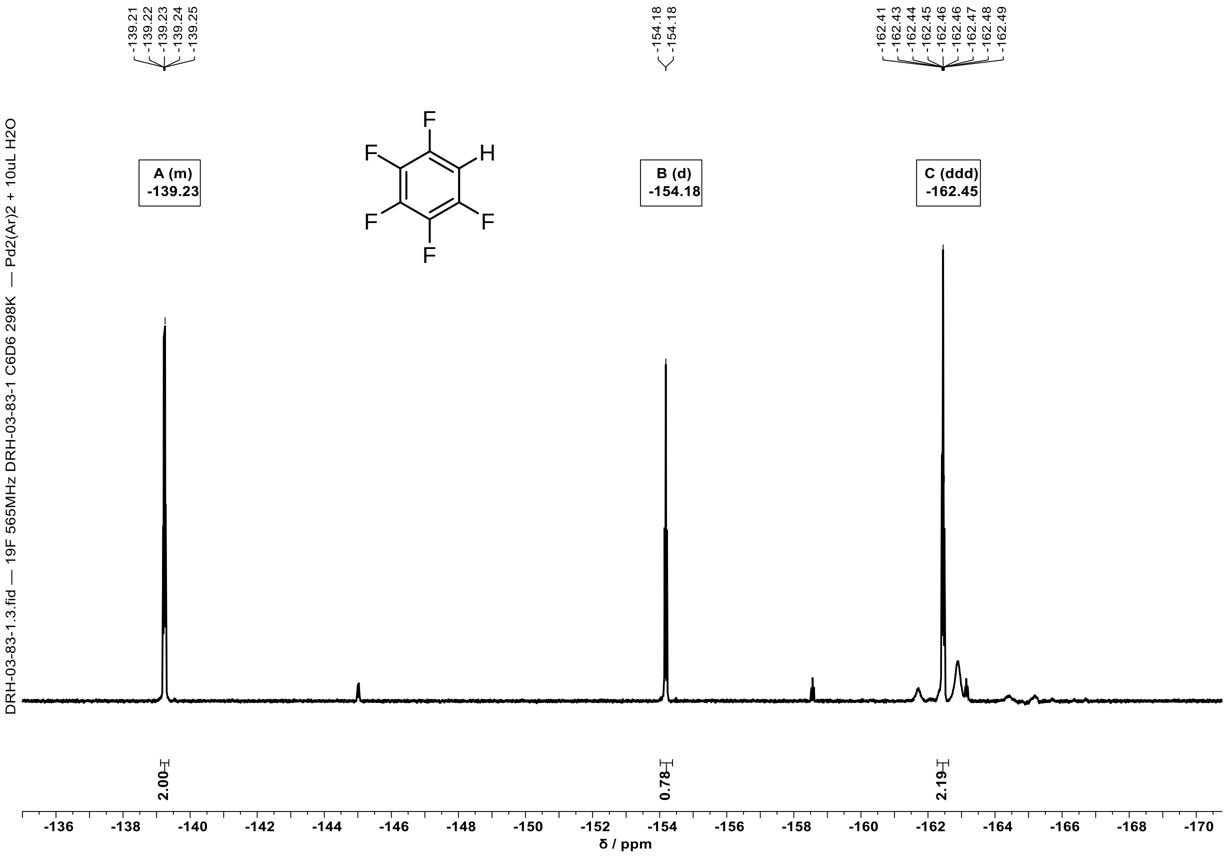

Figure 74: ^{19}F NMR (565 MHz, C_6D_6, 128 sc, 298 K) of the reaction mixture between [Pd(P^C)(μ$_2$-Ar^F)]$_2$ palladacycle 16a and an excess of water. Lab book ref. DRH-03-83-1

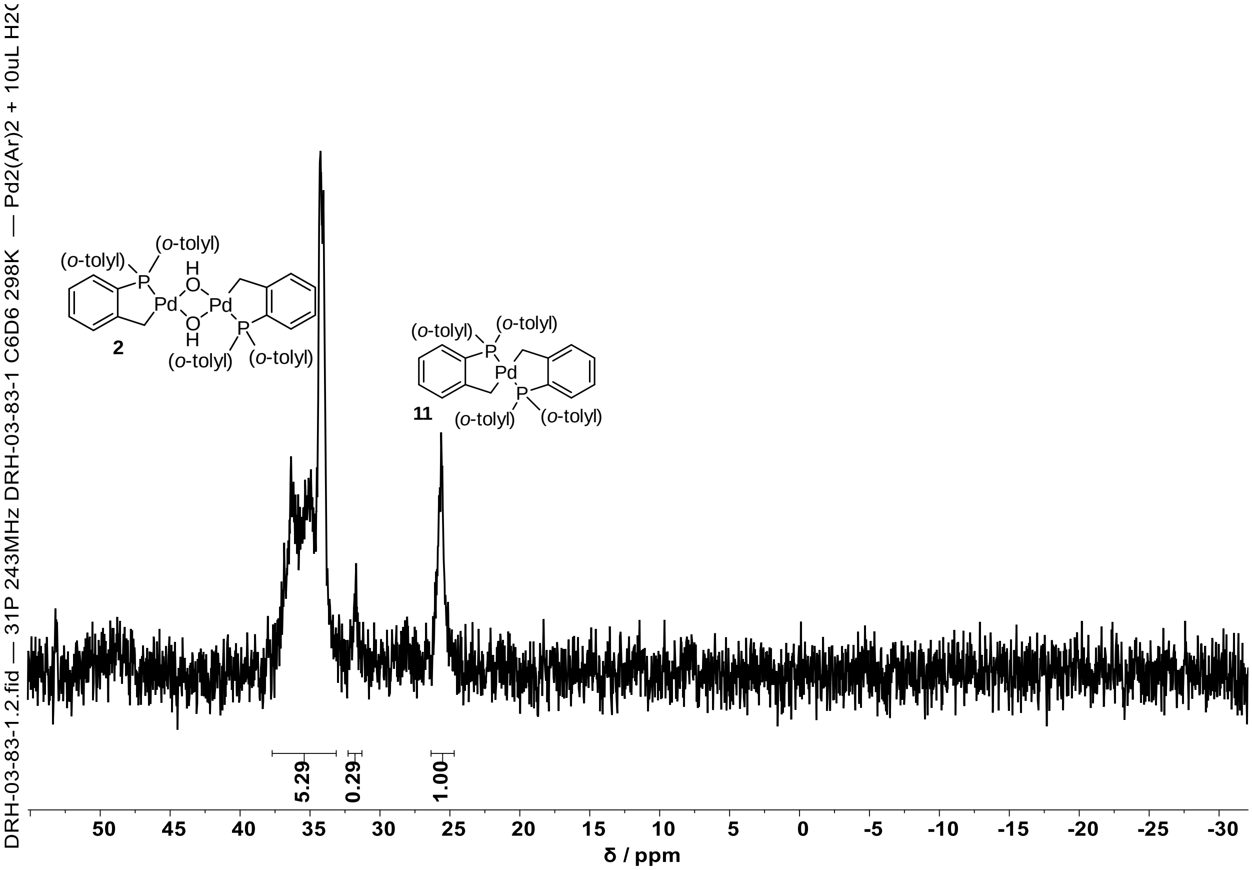

Figure 75: ^{31}P NMR (243 MHz, C_6D_6, 128 sc, 298 K) of the reaction mixture between [Pd(P^C)(μ_2-Ar^F)]$_2$ palladacycle 16a and an excess of water. Lab book ref. DRH-03-83-1

7.9 Cross-Coupling using [Pd(P^C)(μ_2- Ar^F)]$_2$ Palladacycle 16a

To a J-Young tap NMR tube in an argon glove box, [Pd(P^C)(μ_2- Ar^F)]$_2$ palladacycle **16a** (5 mg, 0.0043 mmol, 1 equiv.) and benzene-d_6 (0.5 mL, dry, degassed) were added. After shaking to ensure dissolution, 1-iodo-4-fluorobenzene **31** was added (10 µL, 0.0867 mmol, 20 equiv.), and the vessel was sealed. The sample was immediately analyzed by NMR spectroscopy, then left to stand at room temperature for 60 h before subsequent analysis.

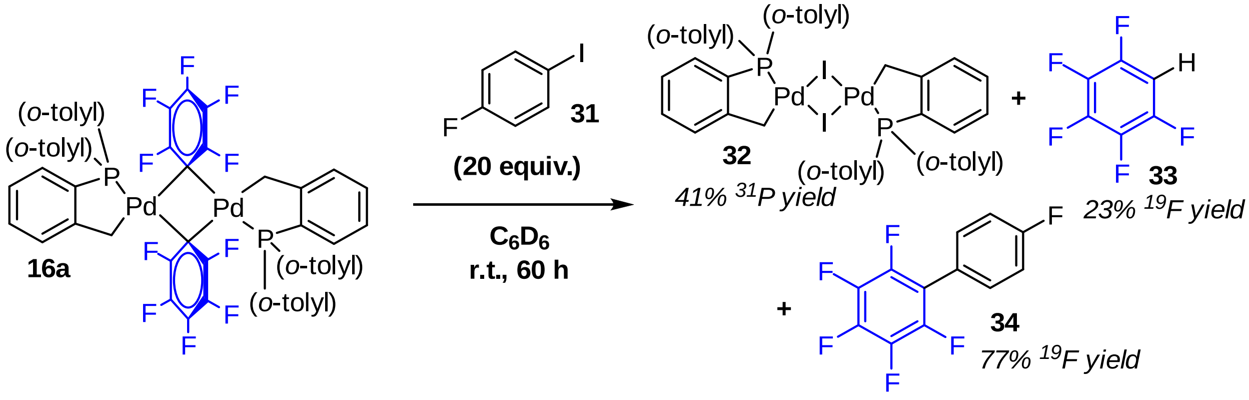

Lab book ref. DRH-03-83-2

In the presence of an aryl halide, the [Pd(P^C)(μ_2-Ar^F)]$_2$ palladacycle **16a** slowly cross-couples, forming (among other things) the [Pd(P^C)(μ_2-I)]$_2$ palladacycle **32** (confirmed by LIFDI mass spectrometry; ($C_{42}H_{40}P_2Pd_2I_2$)$^{+\bullet}$ *m/z* (calculated) 1071.87588, (found) 1071.87974, with a mass difference of 3.60 ppm.. A small amount of pentafluorobenzene **33** suggests that trace water was present, facilitating the activation to Pd$^{(0)}$. An unknown complex at ^{31}P δ 29 ppm was observed initially (Figure 77), which could be a Pd$^{(IV)}$ complex (stabilized by the C_6F_5 group) formed by the oxidative addition of aryl iodide to the Pd$^{(II)}$ monomer **16a-m**. Over time this complex decays and forms the cross-coupled product 34 and [Pd(P^C)(μ_2-I)]$_2$ palladacycle **32**.

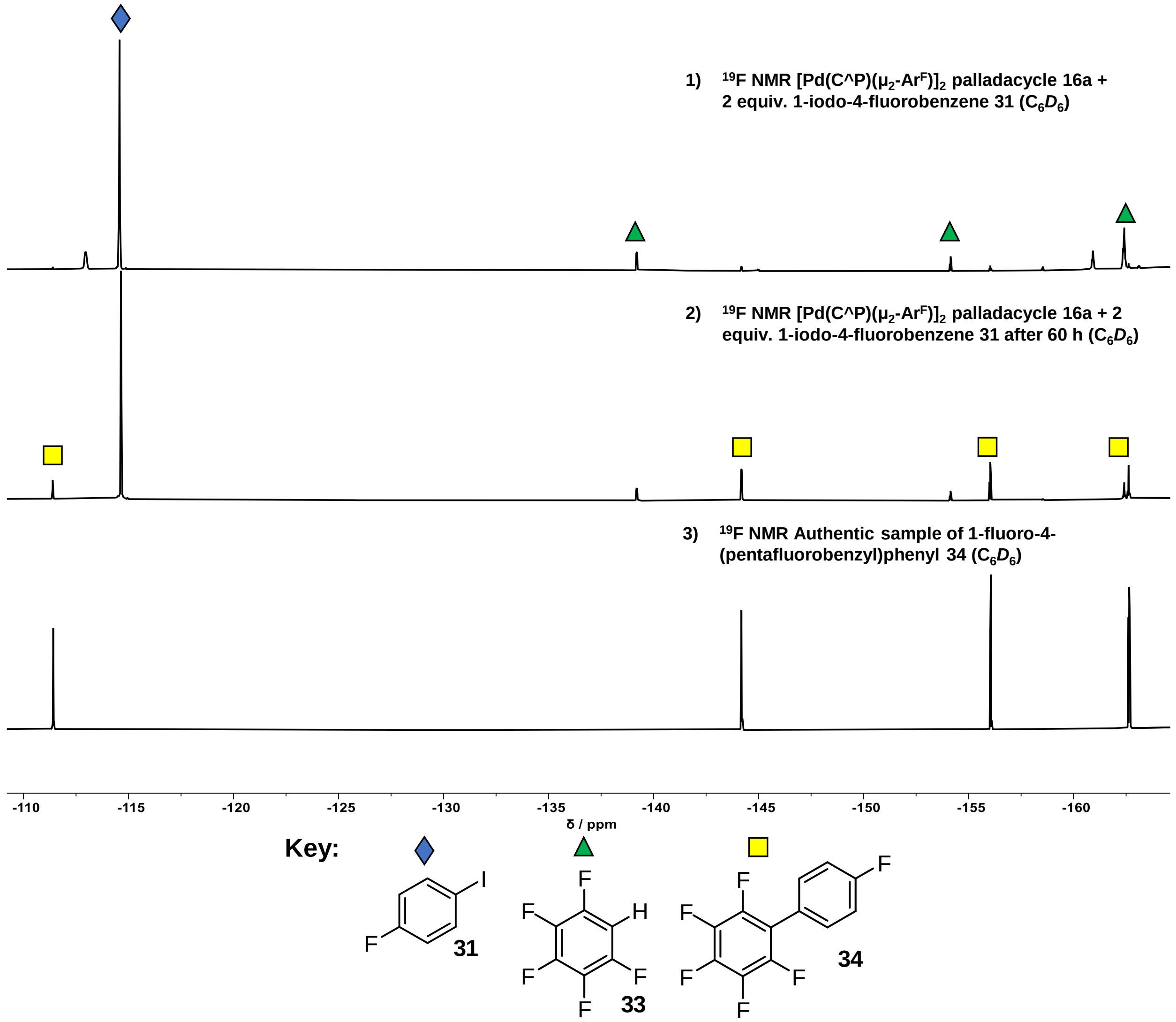

Figure 76: ^{19}F NMR (565 MHz, C_6D_6, 298 K) showing the activation of [Pd(P^C)(μ_2-Ar^F)]$_2$ palladacycle 16a with 1-iodo-4-fluorobenzene 31 (lab book ref. DRH-03-83-2), along with an authentic sample of 1-fluoro-4-(pentafluorobenzyl)phenyl 34 (provided by Dr. Gayathri Athavan, lab book ref. DRH-03-83-3). Integrals of 2): 33 (δ -139.2 ppm) = 1.00, 34 (δ -144.2 ppm) = 3.32.

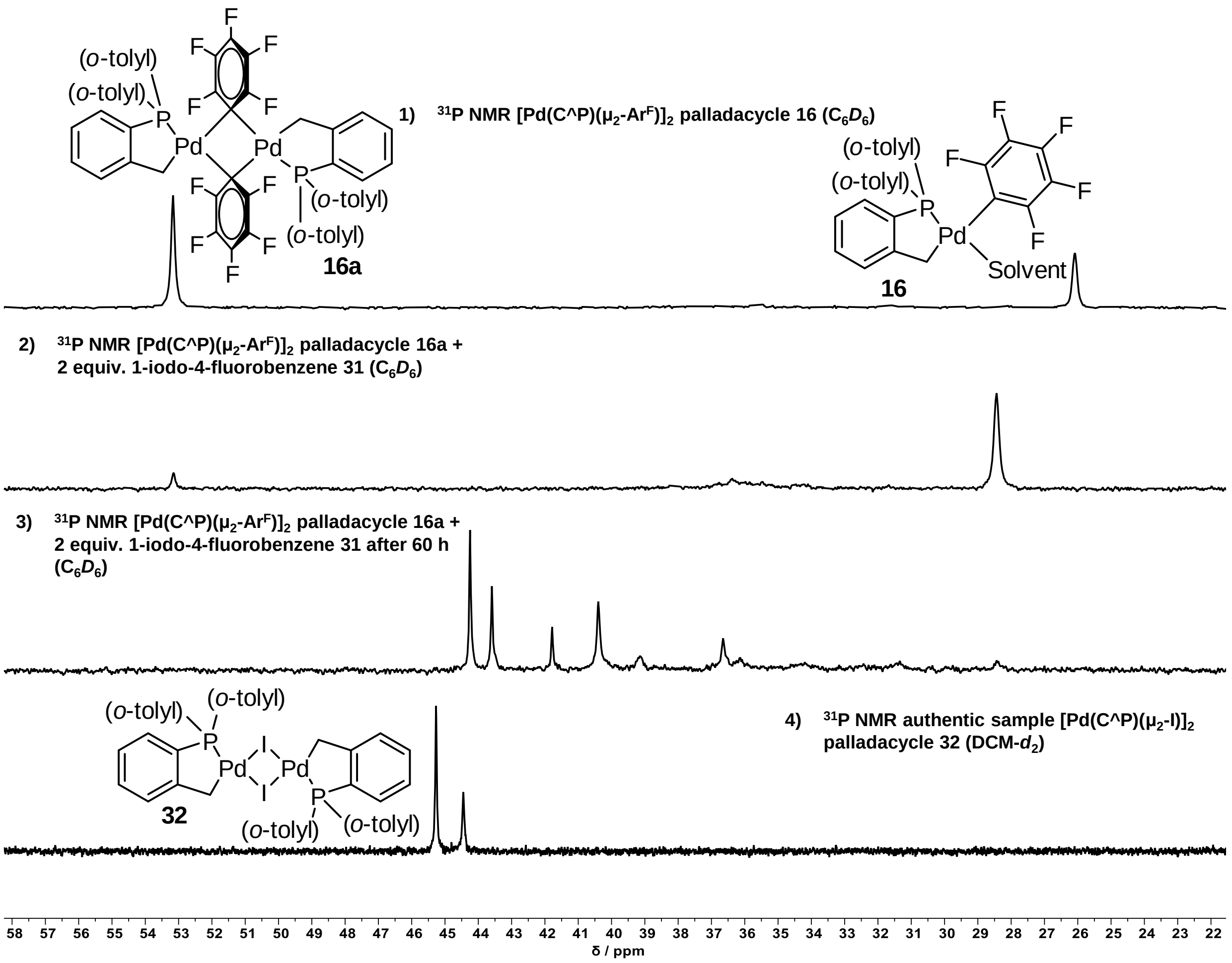

Figure 77: ^{31}P NMR (243 MHz, C_6D_6, 298 K) showing the activation of the [Pd(P^C)(μ$_2$-Ar^F)]$_2$ palladacycle 16a (lab book ref. DRH-03-79) with 1-iodo-4-fluorobenzene 31 (lab book ref. DRH-03-83-2), along with an authentic sample of [Pd(P^C)(μ$_2$-I)]$_2$ palladacycle 32 (202 MHz, DCM-d_2, 298K, lab book ref. DRH-02-3-2). Due to the low solubility of the [Pd(P^C)(μ$_2$-I)]$_2$ palladacycle 32 in most solvents, it was not possible to obtain a spectrum in C_6D_6. Integrals of 3): 32 (δ 44 ppm) = 1.00, (δ 42 ppm) = 0.19, (δ 40 ppm) = 0.52, (δ 39 ppm) = 0.17, (δ 36 ppm) = 0.47, (δ 28 ppm) = 0.10.

7.10 Incorporation of Deuterium under SMCC Reaction Conditions

To confirm that water is essential in activation of the [Pd(P^C)(μ$_2$-OH)]$_2$ palladacycle **2** complex *via* arylboronic acid, two standard Suzuki reactions were run, one as per General Procedure 2.2, and one with under the same conditions but with NMP / D_2O as the solvent. After 18 h, the reactions were worked up as per the general procedure, and the crude analyzed by ^{1}H and ^{2}H NMR. A clear ^{2}H peak at δ 7.03 ppm shows ^{2}H incorporation into anisole, the proposed activation product from the boronic acid.

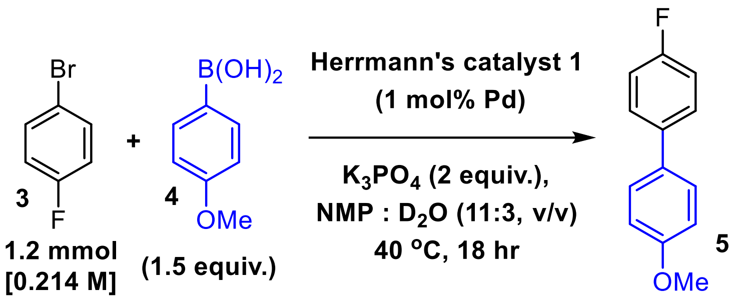

Lab book ref. DRH-03-42

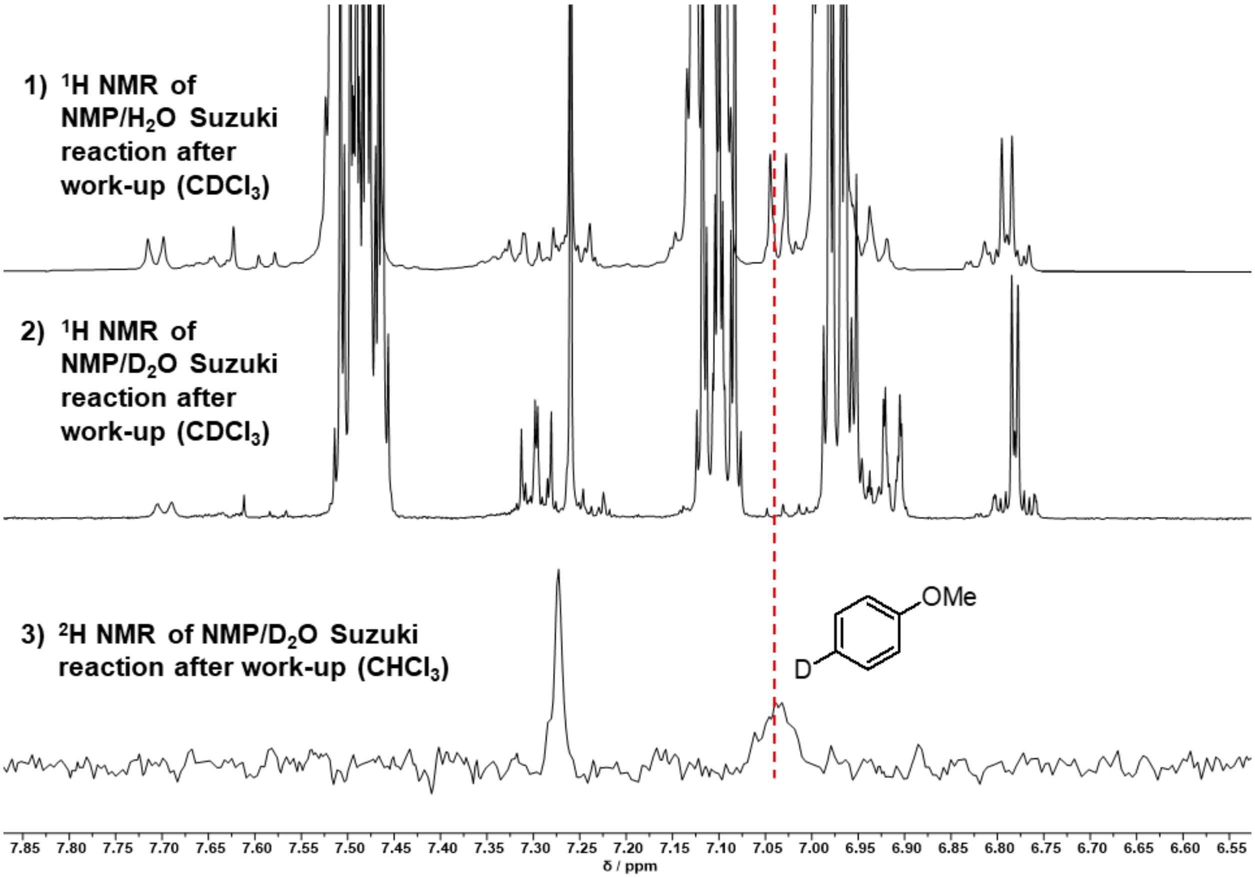

Figure 78: ^{1}H NMR (500 MHz, 298 K) and ^{2}H NMR (77 MHz, 298 K) stacked spectra showing the incorporation of ^{2}H into anisole during a SMCC reaction. Lab book ref. DRH-03-42

8. X-Ray Crystallography

Diffraction data were collected at 110 K on an Oxford Diffraction SuperNova diffractometer with Cu-K_α radiation (λ = 1.54184 Å) or Mo-K_α radiation (λ = 0.71073), using an EOS CCD camera. The crystal was cooled with an Oxford Instruments Cryojet. Diffractometer control, data collection, initial unit cell determination, frame integration and unit-cell refinement was carried out with “Crysalis”.[16] Face-indexed absorption corrections were applied using spherical harmonics, implemented in SCALE3 ABSPACK scaling algorithm.[17] OLEX2[18] was used for overall structure solution and refinement . Within OLEX2, the algorithm used for structure solution was “ShelXT dual-space”.[19] Refinement was carried out by full-matrix least-squares used the SHELXL-97[19] algorithm within OLEX2. All non-hydrogen atoms were refined anisotropically. Crystalmaker® software was used to visualize the structures as well as generating the figures presented herein.

ijsf2007: The Herrmann-Beller Palladacycle 1 CCDC 2288575 (lab book ref. DRH-01-47)

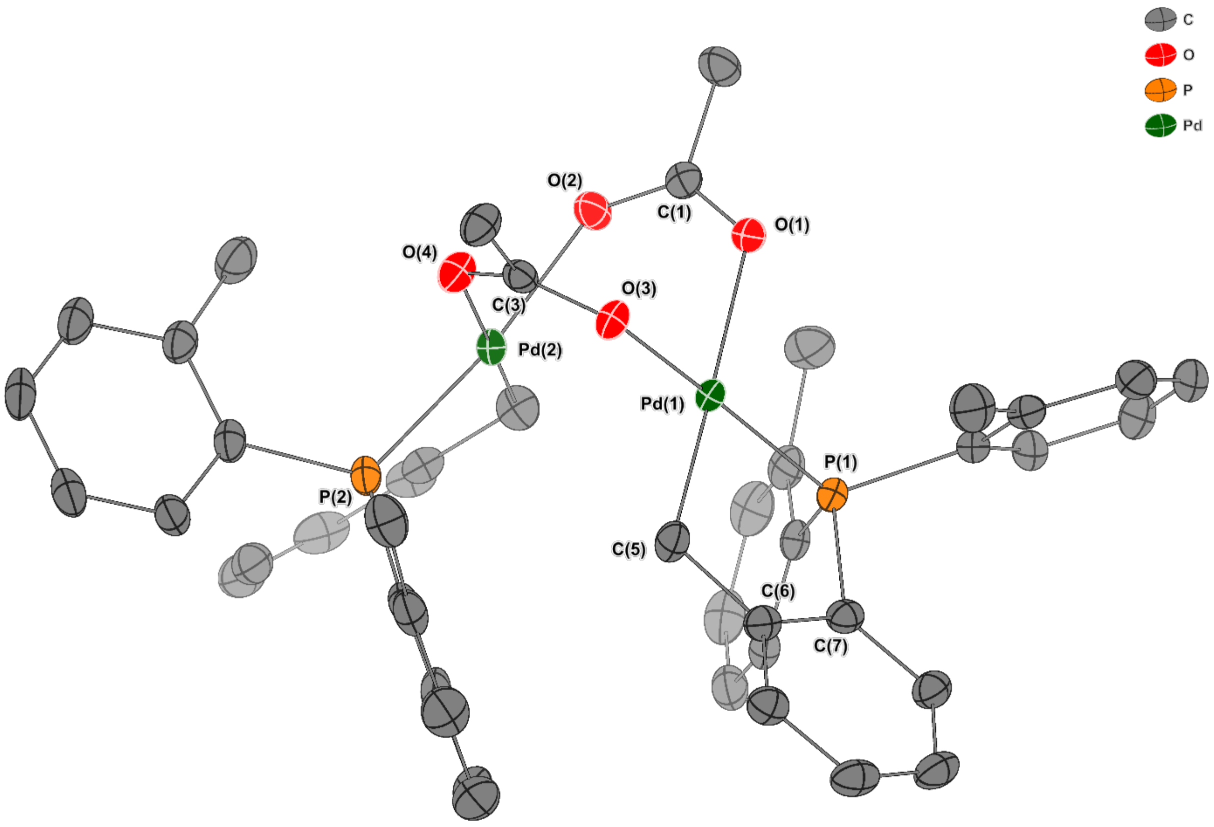

Figure 79: Structure, obtained by X-ray diffraction, of a single crystal of *trans*-bis(acetato)bis[*o*-(di-*o*-tolylphosphino)benzyl]dipalladium(II) 1 (thermal ellipsoids are set at 50% probability, H-atoms are omitted for clarity). Selected interatomic lengths /Å: Pd1-C5 = 2.030(3); Pd1-P1 = 2.2187(8); Pd1-O1 = 2.143(2); Pd1-O3 = 2.113(2); C1-O1 = 1.258(4); Pd1-Pd1 = 3.0979(3). Selected interatomic angles /°: C5-Pd1-P1 = 82.41(9); C5-Pd1-O3 = 88.53(11); P1-Pd1-O1 = 101.12(6); O1-Pd1-O3 = 87.42(8).

Refinement Special Details

The asymmetric unit contained a partially occupied hexane of crystallization. This was modelled in two positions with refined occupancies of 0.090(6) and 0.084(6). The hexanes were modelled isotropically with the ADP of the carbons in each hexane constrained to be the same. C-C bond lengths were restrained to be 1.52 angstroms and 1-3 C-C-C distances restrained to be 2.48 angstroms.

Table 13: X-Ray Diffraction Data for the Herrmann-Beller Palladacycle 1

Identification code	ijsf2007
**Empirical formula**	$C_{46.52}H_{47.22}O_4P_2Pd_2$
**Formula weight**	945.07
**Temperature/K**	110.00(10)
**Crystal system**	monoclinic
**Space group**	$P2_1/c$
**a/Å**	18.6081(4)

**b/Å**	24.0179(3)
**c/Å**	20.6550(3)
**α/°**	90
**β/°**	115.708(2)
**γ/°**	90
**Volume/Å^3**	8317.6(3)
**Z**	8
**ρ_{calc}g/cm^3**	1.509
**μ/mm^{-1}**	8.040
**F(000)**	3843.0
**Crystal size/mm^3**	0.189 × 0.066 × 0.035
**Radiation**	Cu-K_α (λ = 1.54184)
**2Θ range for data collection/°**	7.362 to 134.156
**Index ranges**	-21 ≤ h ≤ 22, -28 ≤ k ≤ 24, -22 ≤ l ≤ 24
**Reflections collected**	32181
**Independent reflections**	14840 [R_{int} = 0.0271, R_{sigma} = 0.0350]
**Data/restraints/parameters**	14840/18/1030
**Goodness-of-fit on F^2**	1.052
**Final R indexes [I>=2σ (I)]**	R_1 = 0.0292, wR_2 = 0.0645
**Final R indexes [all data]**	R_1 = 0.0399, wR_2 = 0.0703
**Largest diff. peak/hole / e Å^{-3}**	0.69/-0.80

ijsf2016: [Pd(P^C)(μ_2-OH)]$_2$ palladacycle 2 (DCM) CCDC 2288581 (lab book ref. DRH-01-108)

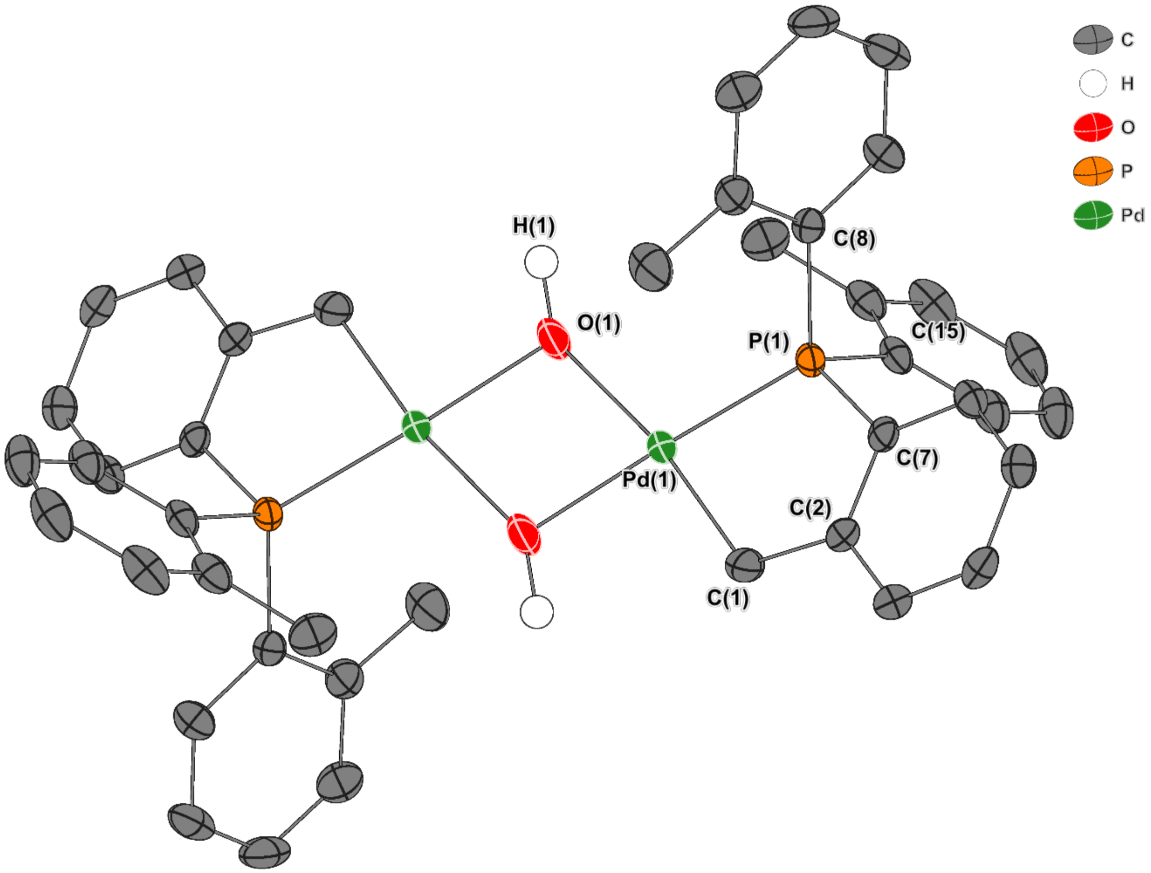

Figure 80: Structure, obtained by X-ray diffraction, of a single crystal of *trans*-di(μ-hydroxido)di[*o*-(di-*o*-tolylphosphino)benzyl] dipalladium(II) 2 (thermal ellipsoids are set at 50% probability, selected H-atoms are omitted for clarity). The asymmetric unit contained half of the complex plus 0.5 dichloromethane of crystallization. Selected interatomic lengths /Å: Pd1-C1 = 2.022(3); Pd1-P1 = 2.1967(7); Pd1-O1 = 2.116(2); Pd1-Pd1A = 3.2269(4). Selected interatomic angles /°: C1-Pd1-P1 = 83.62(9); P1-Pd1-O1 = 105.25(6); C1-Pd1-O1A = 91.81(11); O1-Pd1-O1A = 79.25(10).

Refinement Special Details

The dichloromethane was disordered and modeled over four sites which included a symmetry rotation about the b-axis. Hence, the asymmetric unit contained half a dichloromethane modeled in two positions with refined occupancies of 0.371:0.129(3). All the C-Cl bond lengths were restrained to be equal. The ADP of pairs of atoms were constrained to be equal due to their proximity: Cl2A & C22B, Cl1B & C22A. The OH hydrogen was located by difference map and the location fixed.

Table 14: X-Ray Diffraction Data for the [Pd(P^C)(μ_2-OH)]$_2$ palladacycle 2

**Identification code**	**ijsf2016**
**Empirical formula**	$C_{43}H_{44}Cl_2O_2P_2Pd_2$
**Formula weight**	938.42

**Temperature/K**	110.00(14)
**Crystal system**	monoclinic
**Space group**	C2/c
**a/Å**	15.3360(4)
**b/Å**	14.0754(3)
**c/Å**	18.0616(5)
**α/°**	90
**β/°**	97.241(3)
**γ/°**	90
**Volume/Å^3**	3867.68(17)
**Z**	4
**ρ_{calc}g/cm^3**	1.612
**μ/mm^{-1}**	1.188
**F(000)**	1896.0
**Crystal size/mm^3**	0.182 × 0.085 × 0.022
**Radiation**	Mo-K_α (λ = 0.71073)
**2Θ range for data collection/°**	6.576 to 60.16
**Index ranges**	-19 ≤ h ≤ 21, -19 ≤ k ≤ 17, -25 ≤ l ≤ 23
**Reflections collected**	11959
**Independent reflections**	5686 [R_{int} = 0.0339, R_{sigma} = 0.0542]
**Data/restraints/parameters**	5686/6/263
**Goodness-of-fit on F^2**	1.032
**Final R indexes [I>=2σ (I)]**	R_1 = 0.0391, wR_2 = 0.0765
**Final R indexes [all data]**	R_1 = 0.0606, wR_2 = 0.0850
**Largest diff. peak/hole / e Å^{-3}**	0.62/-0.72

ijsf21008a: [Pd(P^C)(μ_2-OH)]$_2$ palladacycle 2 (benzene) CCDC 2288586 (lab book ref. DRH-01-123)

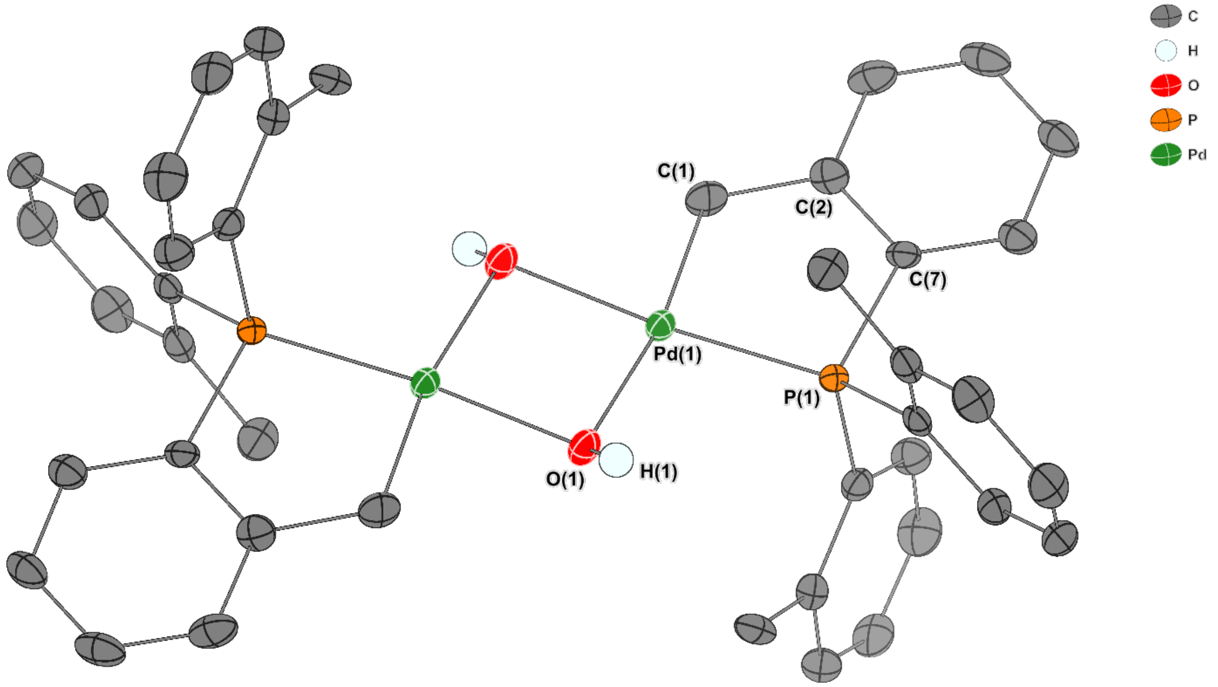

Figure 81: Structure, obtained by X-ray diffraction, of a single crystal of *trans*-di(μ-hydroxido)di[*o*-(di-*o*-tolylphosphino)benzyl] dipalladium(II) 2 (thermal ellipsoids are set at 50% probability, selected H-atoms are omitted for clarity). The unit cell contained one benzene of crystallization. Selected bond lengths (Å) and angles (°); Pd1-C1 = 2.036(3); Pd1-P1 = 2.1998(7); Pd1-O1 = 2.0768(19); Pd1-Pd1A = 3.2459(4); C1-Pd1-P1 = 83.67(9); P1-Pd1-O1 = 106.10(6); C1-Pd1-O1A = 91.57(11); O1-Pd1-O1A = 78.72(8).

Table 15: X-Ray Diffraction Data for the [Pd(P^C)(μ_2-OH)]$_2$ palladacycle 2

**Identification code**	**ijsf21008a**
**Empirical formula**	$C_{54}H_{54}O_2P_2Pd_2$
**Formula weight**	1009.71
**Temperature/K**	110.00(10)
**Crystal system**	triclinic
**Space group**	P-1
**a/Å**	9.8527(3)
**b/Å**	10.5488(3)
**c/Å**	11.3380(3)
**α/°**	81.736(2)
**β/°**	84.328(2)
**γ/°**	77.514(2)
**Volume/Å^3**	1135.78(6)
**Z**	1
**ρ_{calc}g/cm^3**	1.476

**μ/mm^{-1}**	7.366
**F(000)**	516.0
**Crystal size/mm^3**	0.075 × 0.052 × 0.044
**Radiation**	Cu Kα (λ = 1.54184)
**2Θ range for data collection/°**	7.9 to 134.09
**Index ranges**	-11 ≤ h ≤ 11, -12 ≤ k ≤ 11, -13 ≤ l ≤ 13
**Reflections collected**	12068
**Independent reflections**	4046 [R_{int} = 0.0348, R_{sigma} = 0.0379]
**Data/restraints/parameters**	4046/0/274
**Goodness-of-fit on F^2**	1.043
**Final R indexes [I>=2σ (I)]**	R_1 = 0.0290, wR_2 = 0.0721
**Final R indexes [all data]**	R_1 = 0.0328, wR_2 = 0.0746
**Largest diff. peak/hole / e Å^{-3}**	1.93/-0.61

ijsf21005: [Pd(P^C)(μ_2-OH)]$_2$ palladacycle 2 (THF) CCDC 2288585 (lab book ref. DRH-01-123)

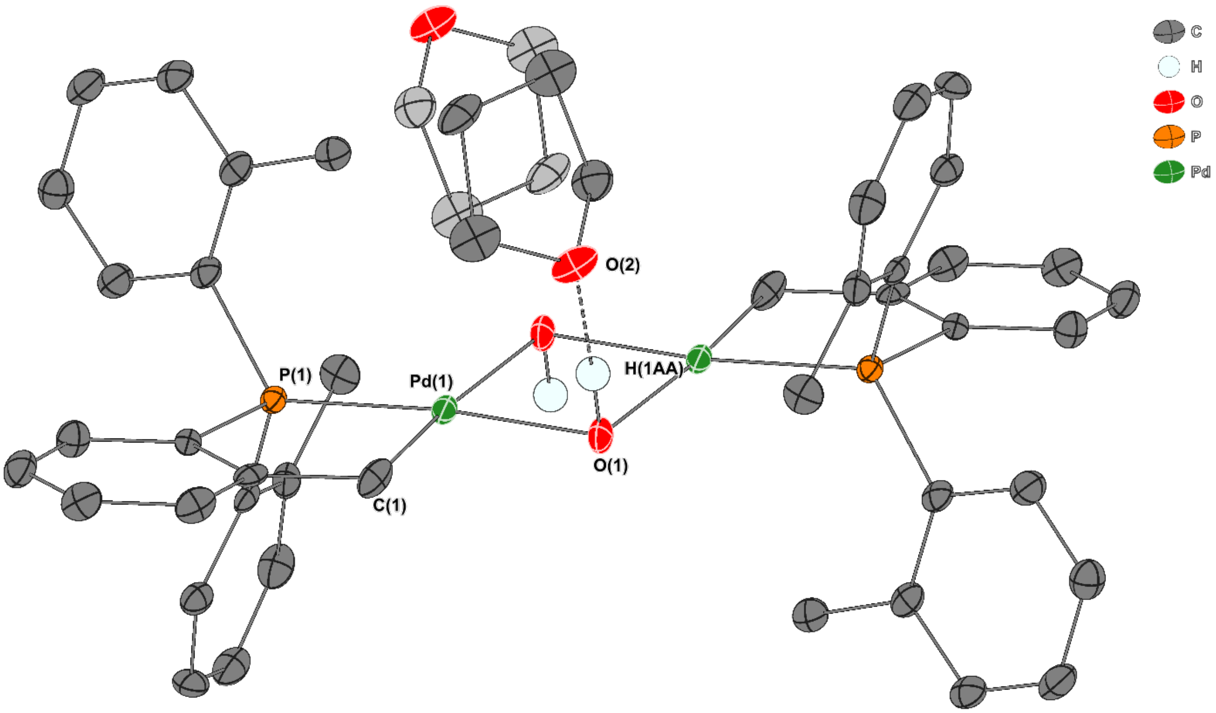

Figure 82: Structure, obtained by X-ray diffraction, of a single crystal of *trans*-di(μ-hydroxido)di[*o*-(di-*o*-tolylphosphino)benzyl] dipalladium(II) 2 (thermal ellipsoids are set at 50% probability, selected H-atoms are omitted for clarity). Selected bond lengths (Å) and angles (°); Pd1-C1 = 2.017(3); Pd1-P1 = 2.2095(8); Pd1-O1 = 2.095(2); Pd1-Pd1A = 3.2248(5); O1-O2 = 3.133(5); C1-Pd1-P1 = 84.29(9); P1-Pd1-O1A = 105.51(6); C1-Pd1-O1 = 89.86(11); O1-Pd1-O1A = 80.51(9).

Refinement Special Details

The unit cell contained one tetrahydrofuran of crystallization which was disordered about the inversion center (i.e. 50% occupancy in the asymmetric unit). Solution in P1 revealed that this disorder was random. This meant that, on average within the complex, only one of the hydroxyl protons was hydrogen-bonded to the tetrahydrofuran. The difference map obtained after other atoms were located and refined revealed two possible locations for the hydroxyl hydrogen and so the hydrogen was placed at both sites, each at 50% occupancy and then fixed to ride on the hydroxyl oxygen.

Table 16: X-Ray Diffraction Data for the [Pd(P^C)(μ_2-OH)]$_2$ palladacycle 2

Identification code	ijsf21005
Empirical formula	$C_{46}H_{50}O_3P_2Pd_2$
Formula weight	925.60
Temperature/K	110.00(10)
Crystal system	triclinic
Space group	P-1
a/Å	9.0785(4)
b/Å	9.3107(6)
c/Å	13.3265(5)
α/°	97.321(4)
β/°	94.084(4)
γ/°	118.918(6)
Volume/Å^3	966.44(10)
Z	1
ρ_{calc}g/cm^3	1.590
μ/mm^{-1}	8.613
F(000)	472.0
Crystal size/mm^3	0.16 × 0.12 × 0.02
Radiation	Cu Kα (λ = 1.54184)
2Θ range for data collection/°	11.048 to 134.158
Index ranges	$-8 \leq h \leq 10$, $-11 \leq k \leq 10$, $-15 \leq l \leq 15$
Reflections collected	6074
Independent reflections	3434 [R_{int} = 0.0329, R_{sigma} = 0.0490]
Data/restraints/parameters	3434/20/258
Goodness-of-fit on F^2	1.067
Final R indexes [I>=2σ (I)]	R_1 = 0.0293, wR_2 = 0.0676
Final R indexes [all data]	R_1 = 0.0351, wR_2 = 0.0709
Largest diff. peak/hole / e Å^{-3}	0.52/-0.76

ijsf21046a: Pd-OH boroxine complex 21 CCDC 2288574 (lab book ref. DRH-02-44)

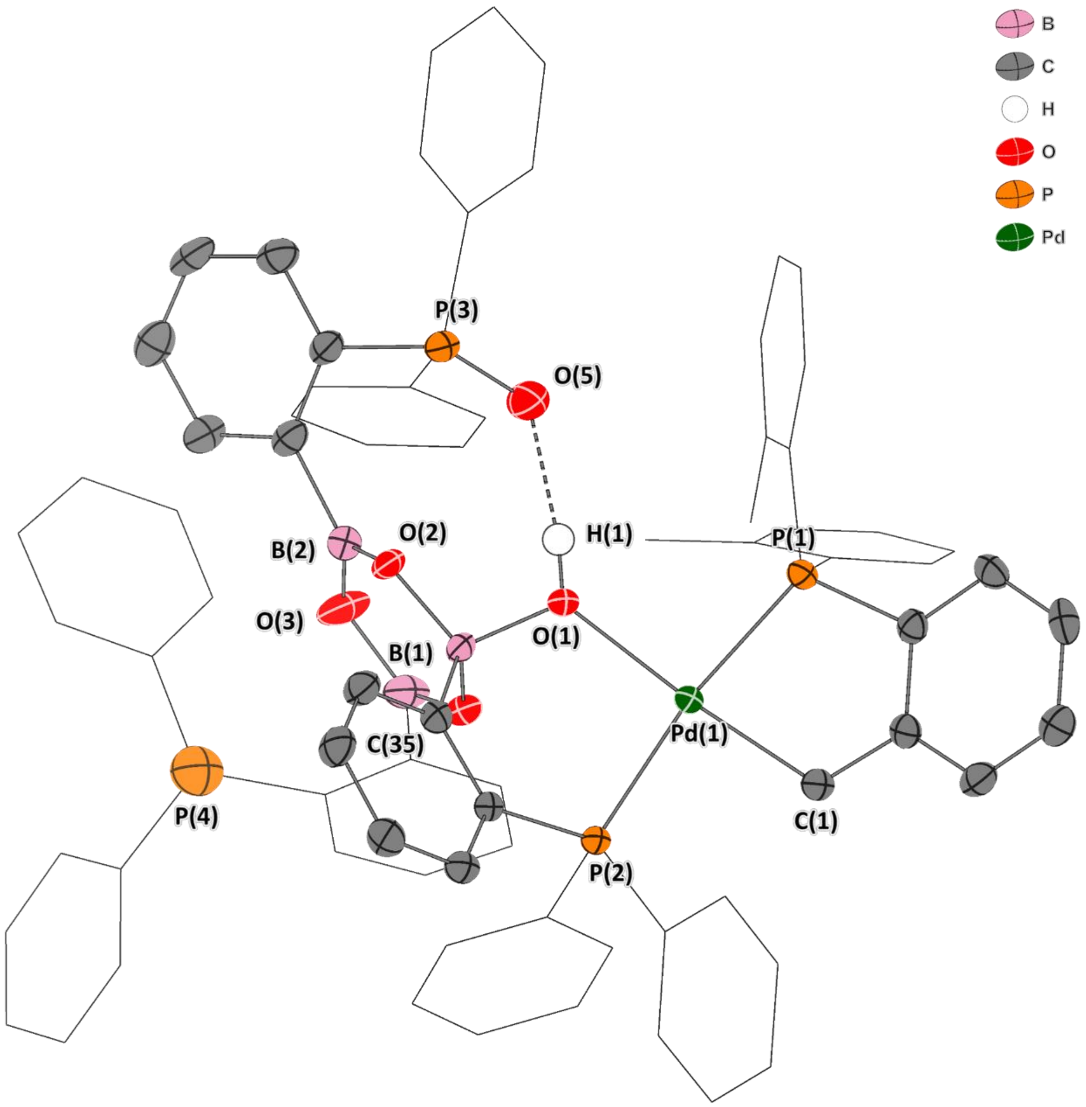

Figure 83: Structure, obtained by X-ray diffraction, of a single crystal of the Pd-OH boroxine complex 21 (thermal ellipsoids are set at 50% probability, selected H-atoms are omitted for clarity, for structural clarity most carbon atoms are displayed as a wireframe structure). Selected interatomic lengths /Å: Pd1-P1 = 2.3048(7); Pd1-P2 = 2.3270(8); Pd1-C1 = 2.060(3); Pd1-O1 = 2.131(2); O5-H1 = 1.969(2); O1-B1 = 1.488(4); O2-B1 = 1.475(4); Selected interatomic angles /°: C1-Pd1-P1 = 79.98(9); P1-Pd1-O1 = 96.22(6); C1-Pd1-P2 = 91.70(9); B1-O2-B2 = 122.0(3).

Table 17: X-Ray Diffraction Data for the Pd-OH boroxine complex 21

**Identification code**	**ijsf21046a**
**Empirical formula**	$C_{75}H_{63}B_3O_5P_4Pd$
**Formula weight**	1306.96
**Temperature/K**	110.00(14)
**Crystal system**	monoclinic
**Space group**	$P2_1/c$

**a/Å**	15.00078(17)
**b/Å**	13.93471(14)
**c/Å**	30.4831(3)
**α/°**	90
**β/°**	90.0585(10)
**γ/°**	90
**Volume/Å^3**	6371.92(11)
**Z**	4
**ρ_{calc}g/cm^3**	1.362
**μ/mm^{-1}**	3.713
**F(000)**	2696.0
**Crystal size/mm^3**	0.116 × 0.08 × 0.054
**Radiation**	Cu Kα (λ = 1.54184)
**2Θ range for data collection/°**	6.974 to 134.156
**Index ranges**	-17 ≤ h ≤ 17, -16 ≤ k ≤ 16, -24 ≤ l ≤ 36
**Reflections collected**	24381
**Independent reflections**	10504 [R_{int} = 0.0228, R_{sigma} = 0.0282]
**Data/restraints/parameters**	10504/0/797
**Goodness-of-fit on F^2**	1.033
**Final R indexes [I>=2σ (I)]**	R_1 = 0.0391, wR_2 = 0.1045
**Final R indexes [all data]**	R_1 = 0.0450, wR_2 = 0.1087
**Largest diff. peak/hole / e Å^{-3}**	0.89/-1.30

ijsf21049: Pd-OH pinacol ester complex 24 CCDC 2288576 (lab book ref. DRH-02-59)

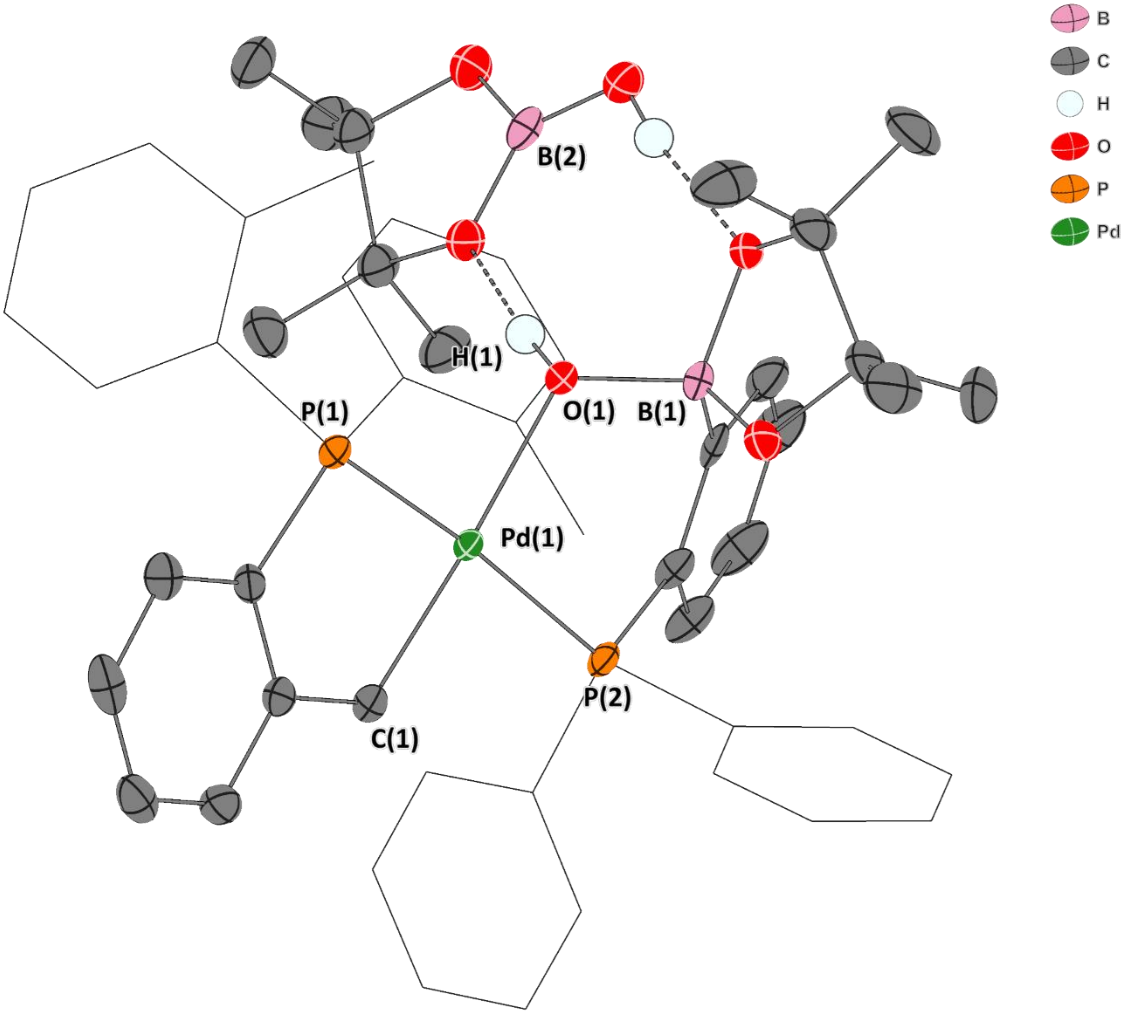

Figure 84: Structure, obtained by X-ray diffraction, of a single crystal of the Pd-OH pinacol ester complex 24 (thermal ellipsoids are set at 50% probability, selected H-atoms are omitted for clarity). Selected interatomic lengths /Å: Pd1-P1 = 2.3002(6); Pd1-P2 = 2.3104(6); Pd1-C1 = 2.045(2); Pd1-O1 = 2.1164(16); O5-H1 = 1.854(10); O1-B1 = 1.509(3); Selected interatomic angles /°: C1-Pd1-P1 = 84.65(7); P1-Pd1-O1 = 94.93(5); C1-Pd1-P2 = 89.79(7); P2-Pd1-O1 = 90.93(5), O1-H1-O5 = 174.6(17).

Table 18: X-Ray Diffraction Data for the Pd-OH pinacol ester complex 24

**Identification code**	**ijsf21049**
**Empirical formula**	$C_{51}H_{60}B_2O_6P_2Pd$
**Formula weight**	958.95
**Temperature/K**	110.00(10)
**Crystal system**	monoclinic
**Space group**	$P2_1/c$
**a/Å**	14.8238(3)
**b/Å**	14.0863(3)
**c/Å**	23.6678(5)

**α/°**	90
**β/°**	95.389(2)
**γ/°**	90
**Volume/Å^3**	4920.29(18)
**Z**	4
**ρ_{calc}g/cm^3**	1.295
**μ/mm^{-1}**	4.023
**F(000)**	2000.0
**Crystal size/mm^3**	0.242 × 0.185 × 0.16
**Radiation**	Cu Kα (λ = 1.54184)
**2Θ range for data collection/°**	7.312 to 142.45
**Index ranges**	-17 ≤ h ≤ 17, -17 ≤ k ≤ 9, -24 ≤ l ≤ 29
**Reflections collected**	18429
**Independent reflections**	9291 [R_{int} = 0.0373, R_{sigma} = 0.0516]
**Data/restraints/parameters**	9291/3/573
**Goodness-of-fit on F^2**	1.036
**Final R indexes [I>=2σ (I)]**	R_1 = 0.0334, wR_2 = 0.0765
**Final R indexes [all data]**	R_1 = 0.0408, wR_2 = 0.0811
**Largest diff. peak/hole / e Å^{-3}**	0.45/-0.86

ijsf21051: Pd bipalladacycle 25 CCDC 2288579 (lab book ref. DRH-02-66)

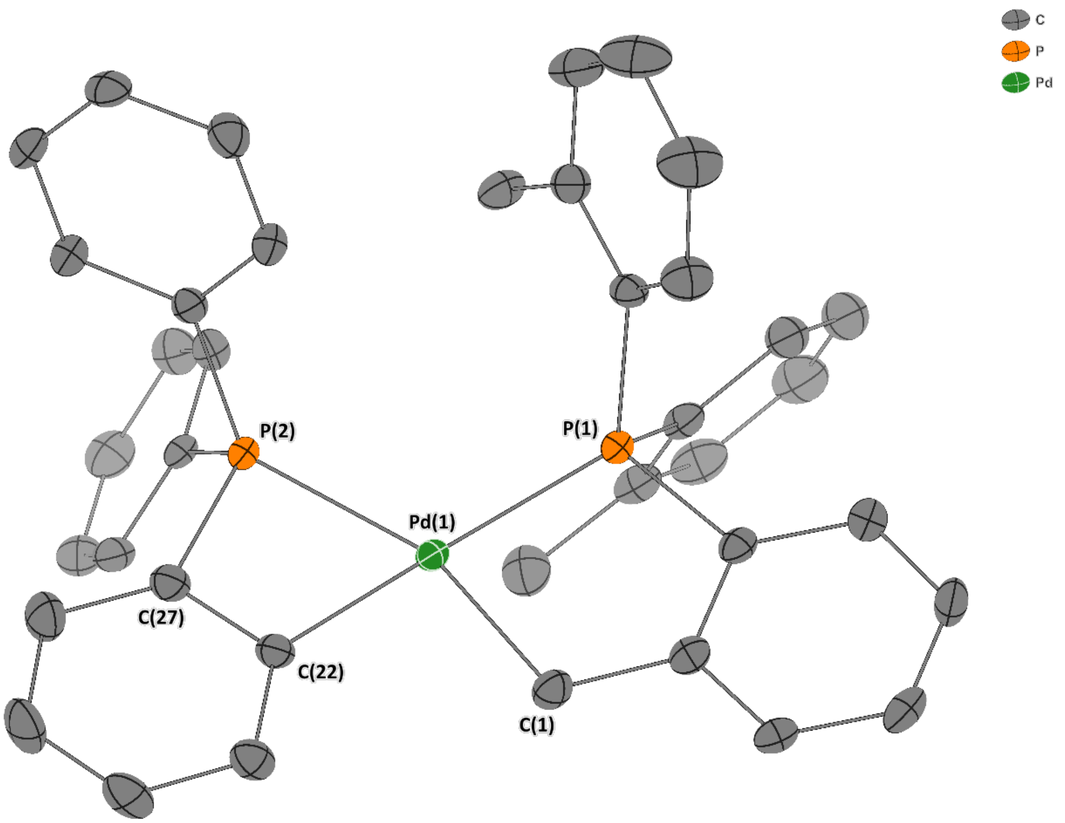

Figure 85: Structure, obtained by X-ray diffraction, of a single crystal of the Pd bipalladacycle complex 25 (thermal ellipsoids are set at 50% probability, H-atoms are omitted for clarity). Selected interatomic lengths /Å: Pd1-P1 = 2.3156(7); Pd1-P2 = 2.3580(7); Pd1-C1 = 2.064(3); Pd1-C22 = 2.054(3); P2-C27 = 1.808(3); C22-C27 = 1.395(4); Selected interatomic angles /°: P1-Pd1-P2 = 115.10(2); P1-Pd1-C1 = 84.72(8); C1-Pd1-C22 = 91.59(11); P2-Pd1-C22 = 68.70(8); P2-C27-C22 = 101.97(19); Pd1-P2-C27 = 82.94(9).

Table 19: X-Ray Diffraction Data for the Pd bipalladacycle complex 25

Identification code	ijsf21051
Empirical formula	$C_{42}H_{37}P_2Pd$
Formula weight	710.05
Temperature/K	110.00(10)
Crystal system	triclinic
Space group	P-1
a/Å	10.9865(4)
b/Å	12.3847(4)
c/Å	13.2985(5)
α/°	76.348(3)
β/°	74.727(3)
γ/°	77.403(3)
Volume/Å^3	1672.60(11)

**Z**	2
**ρ_{calc}g/cm^3**	1.410
**μ/mm^{-1}**	5.591
**F(000)**	730.0
**Crystal size/mm^3**	0.17 × 0.14 × 0.1
**Radiation**	CuKα (λ = 1.54184)
**2Θ range for data collection/°**	7.018 to 141.878
**Index ranges**	-13 ≤ h ≤ 10, -15 ≤ k ≤ 14, -16 ≤ l ≤ 13
**Reflections collected**	11737
**Independent reflections**	6319 [R_{int} = 0.0284, R_{sigma} = 0.0410]
**Data/restraints/parameters**	6319/3/461
**Goodness-of-fit on F^2**	1.020
**Final R indexes [I>=2σ (I)]**	R_1 = 0.0297, wR_2 = 0.0665
**Final R indexes [all data]**	R_1 = 0.0358, wR_2 = 0.0698
**Largest diff. peak/hole / e Å^{-3}**	0.72/-0.73

ijsf21052: Pd-maleic acid complex 27 CCDC 2288583 (lab book ref. DRH-02-79)

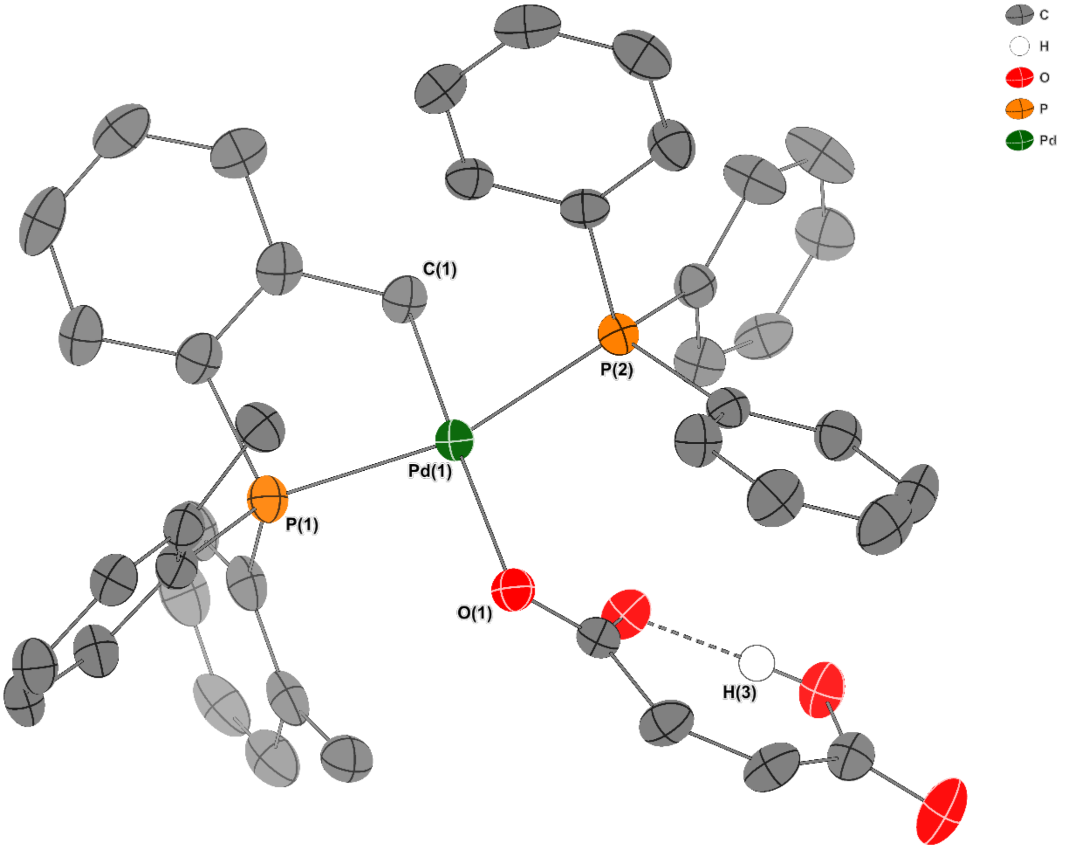

Figure 86: Structure, obtained by X-ray diffraction, of a single crystal of the Pd–maliec acid complex 27 (thermal ellipsoids are set at 50% probability, selected H-atoms are omitted for clarity). Selected interatomic lengths /Å: Pd1-P1 = 2.3200(10); Pd1-P2 = 2.3023(9); Pd1-C1 = 2.062(4); Pd1-O1 = 2.164(2); Selected interatomic angles /°: P1-Pd1-O1 = 100.06(7); P1-Pd1-C1 = 81.01(11); C1-Pd1-P2 = 86.65(11); P2-Pd1-O1 = 92.64(7).

Table 20: X-Ray Diffraction Data for the Pd-maleic acid complex 27

Identification code	ijsf21052
**Empirical formula**	$C_{43}H_{38}O_4P_2Pd$
**Formula weight**	787.07
**Temperature/K**	109.95(10)
**Crystal system**	orthorhombic
**Space group**	Pbca
**a/Å**	16.3173(3)
**b/Å**	19.2058(4)
**c/Å**	23.9483(6)
**α/°**	90
**β/°**	90
**γ/°**	90
**Volume/Å^3**	7505.1(3)

**Z**	8
**ρ_{calc}g/cm^3**	1.393
**μ/mm^{-1}**	5.124
**F(000)**	3232.0
**Crystal size/mm^3**	0.206 × 0.151 × 0.055
**Radiation**	Cu Kα (λ = 1.54184)
**2Θ range for data collection/°**	7.382 to 143.978
**Index ranges**	-18 ≤ h ≤ 19, -23 ≤ k ≤ 20, -28 ≤ l ≤ 27
**Reflections collected**	47012
**Independent reflections**	7209 [R_{int} = 0.1189, R_{sigma} = 0.0577]
**Data/restraints/parameters**	7209/0/457
**Goodness-of-fit on F^2**	1.075
**Final R indexes [I>=2σ (I)]**	R_1 = 0.0585, wR_2 = 0.1223
**Final R indexes [all data]**	R_1 = 0.0773, wR_2 = 0.1361
**Largest diff. peak/hole / e Å^{-3}**	1.08/-1.03

ijsf2008: [Pd(C^P)(OAc)(PPh_3)] palladacyclic monomer SI43 CCDC 2288577 (lab book ref. DRH-01-57)

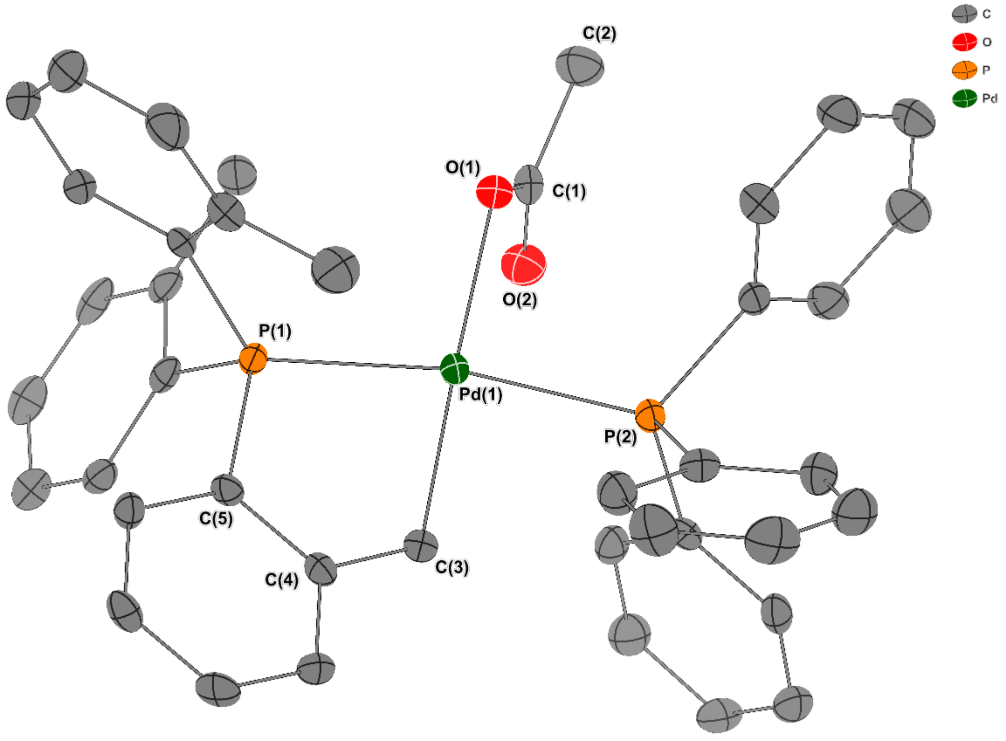

Figure 87: Structure, obtained by X-ray diffraction, of a single crystal of [*o*-(di-*o*-tolylphosphino)benzyl]acetatotriphenylphosphino palladium(II) SI43 (thermal ellipsoids are set at 50% probability, H-atoms are omitted for clarity). Selected bond lengths (Å) and angles (°); Pd1-C3 = 2.066(3); Pd1-P1 = 2.3120(8); Pd1-P2 = 2.2896(8); Pd1-O1 = 2.122(2); O1-C1 = 1.295(4); C3-Pd1-P1 = 81.27(9); C3-Pd1-P2 = 87.11(9); P1-Pd1-O1 = 100.96(6); P2-Pd1-O1 = 91.36(6).

Table 21: X-Ray Diffraction Data for [Pd(C^P)(OAc)(PPh_3)] palladacyclic monomer SI43

Identification code	ijsf2008
Empirical formula	$C_{41}H_{38}O_2P_2Pd$
Formula weight	731.05
Temperature/K	110.00(10)
Crystal system	triclinic
Space group	P-1
a/Å	10.6494(3)
b/Å	17.7946(5)
c/Å	19.4697(5)
α/°	104.753(2)
β/°	96.588(2)
γ/°	101.261(2)
Volume/Å^3	3446.19(18)
Z	4
ρ_{calc}g/cm^3	1.409
μ/mm^{-1}	5.488
F(000)	1504.0
Crystal size/mm^3	0.3 × 0.222 × 0.062
Radiation	Cu Kα (λ = 1.54184)
2Θ range for data collection/°	8.062 to 134.15
Index ranges	-12 ≤ h ≤ 12, -21 ≤ k ≤ 15, -22 ≤ l ≤ 23
Reflections collected	24253
Independent reflections	12307 [R_{int} = 0.0286, R_{sigma} = 0.0389]
Data/restraints/parameters	12307/0/836
Goodness-of-fit on F^2	1.052
Final R indexes [I>=2σ (I)]	R_1 = 0.0353, wR_2 = 0.0881
Final R indexes [all data]	R_1 = 0.0406, wR_2 = 0.0918
Largest diff. peak/hole / e Å^{-3}	1.13/-1.38

ijsf2005: [Pd(C^P)(Cl)(PPh$_3$)] palladacyclic monomer SI44 CCDC 2288578 (lab book ref. DRH-01-33)

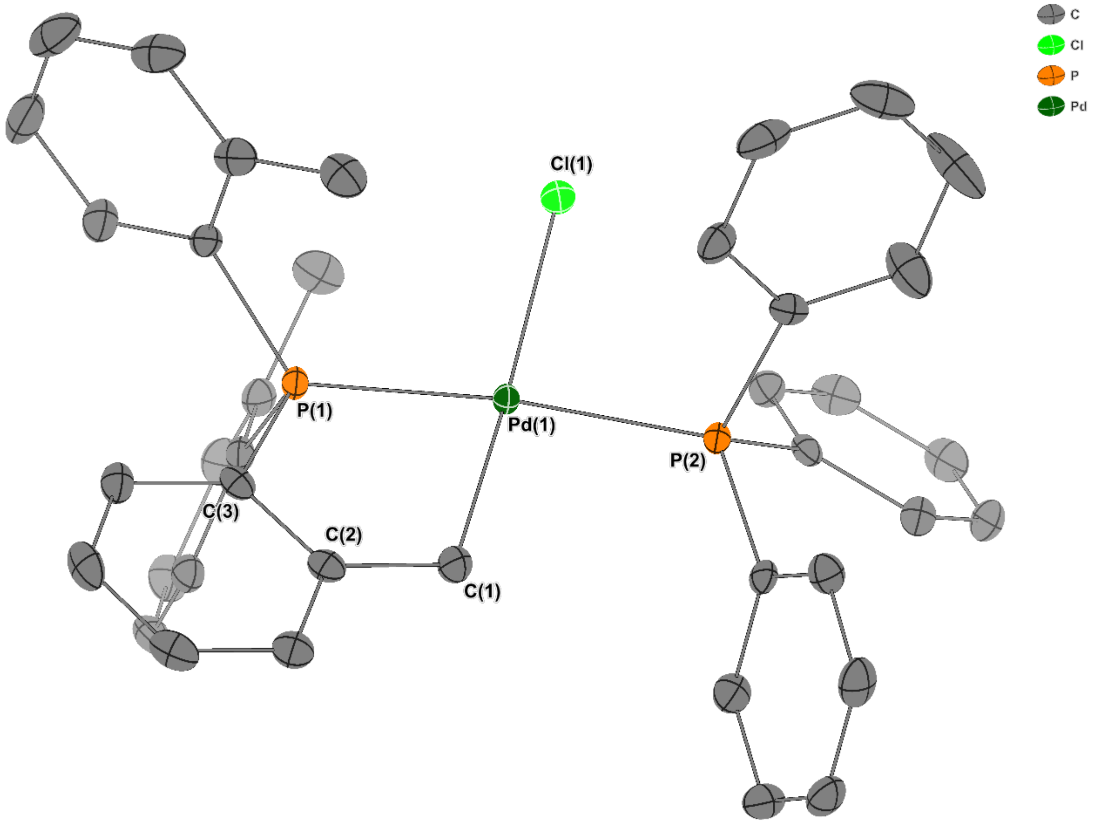

Figure 88: Structure, obtained by X-ray diffraction, of a single crystal of [*o*-(di-*o*-tolylphosphino)benzyl]chlorotriphenylphosphino palladium(II) SI44 (thermal ellipsoids are set at 50% probability, H-atoms are omitted for clarity). Selected bond lengths (Å) and angles (°); Pd1-C1 = 2.066(2); Pd1-P1 = 2.3010(6); Pd1-P2 = 2.3342(6); Pd1-Cl1 = 2.4038(6); C1-Pd1-P1 = 81.23(7); C1-Pd1-P2 = 93.07(7); P1-Pd1-Cl1 = 97.02(2); P2-Pd1-Cl1 = 88.860(19).

Table 22: X-Ray Diffraction Data for [Pd(C^P)(Cl)(PPh$_3$)] palladacyclic monomer SI44

Identification code	ijsf2005
Empirical formula	$C_{39}H_{35}P_2ClPd$
Formula weight	707.46
Temperature/K	110.00(10)
Crystal system	monoclinic
Space group	$P2_1/c$
a/Å	12.1582(3)
b/Å	15.4824(4)
c/Å	18.0829(4)
α/°	90
β/°	106.744(3)

**γ/°**	90
**Volume/Å^3**	3259.58(14)
**Z**	4
**ρ_{calc}g/cm^3**	1.442
**μ/mm^{-1}**	6.471
**F(000)**	1448.0
**Crystal size/mm^3**	0.154 × 0.124 × 0.027
**Radiation**	CuKα (λ = 1.54184)
**2Θ range for data collection/°**	7.594 to 134.16
**Index ranges**	-13 ≤ h ≤ 14, -18 ≤ k ≤ 18, -21 ≤ l ≤ 14
**Reflections collected**	12092
**Independent reflections**	5830 [R_{int} = 0.0239, R_{sigma} = 0.0325]
**Data/restraints/parameters**	5830/0/390
**Goodness-of-fit on F^2**	1.035
**Final R indexes [I>=2σ (I)]**	R_1 = 0.0252, wR_2 = 0.0579
**Final R indexes [all data]**	R_1 = 0.0298, wR_2 = 0.0599
**Largest diff. peak/hole / e Å^{-3}**	0.65/-0.49

ijsf21047: $Pd_2(Br)_2(C_6H_4F)_2[P(\textit{o}\text{-tolyl})_3]_2$ oxidative addition dimer SI45 CCDC 2288582 (lab book ref. DRH-02-50)

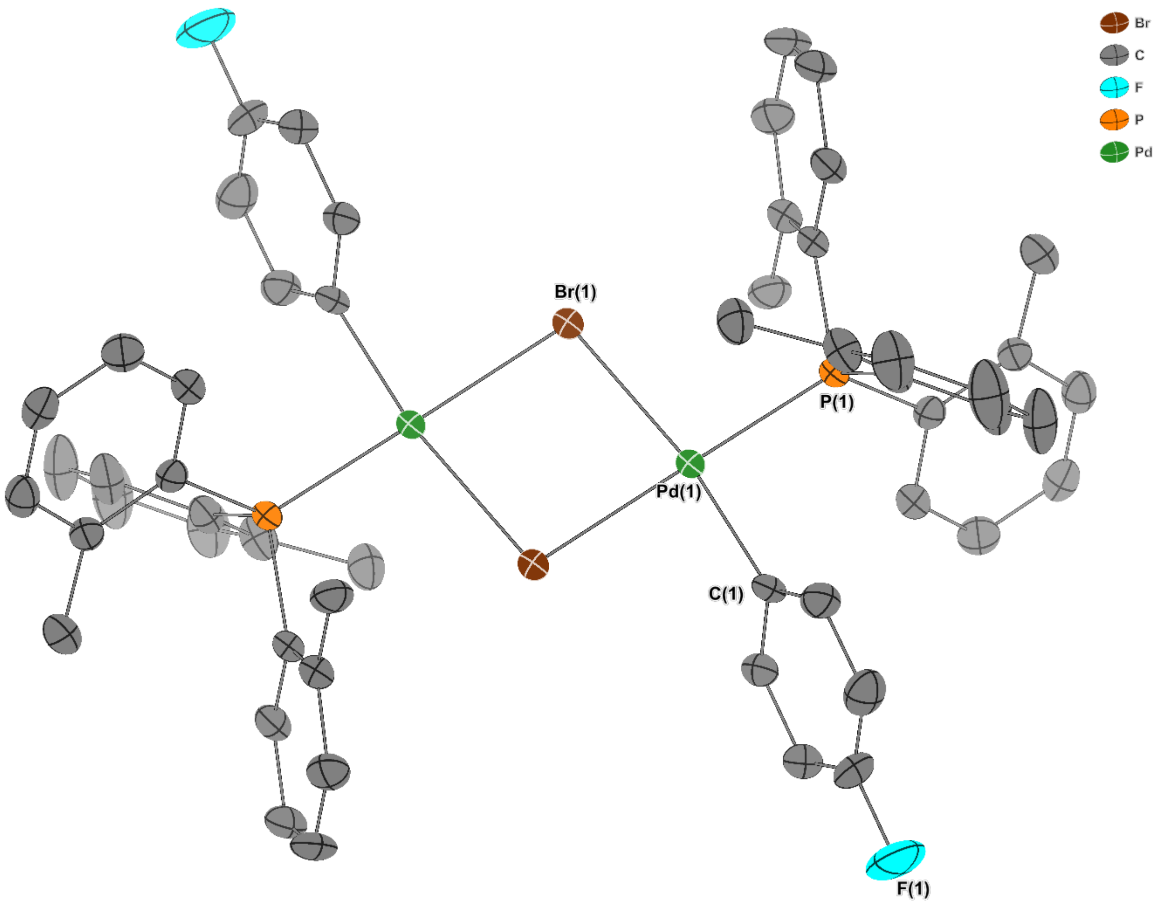

Figure 89: Structure, obtained by X-ray diffraction, of a single crystal of the $Pd_2(Br)_2(C_6H_4F)_2[P(\textit{o}\text{-tolyl})_3]_2$ oxidative addition dimer SI45 (thermal ellipsoids are set at 50% probability, H-atoms are omitted for clarity). Selected interatomic lengths /Å: Pd1-P1 = 2.2809(8); Pd1-Br1 = 2.5782(4); Pd1-C1 = 2.036(3). Selected interatomic angles /°: C1-Pd1-P1 = 90.59(10); P1-Pd1-Br1 = 98.00(2); C1-Pd1-Br1A = 87.71(9); Br1-Pd1-Br1A = 83.801(14).

Refinement Special Details

The crystal contained disordered solvent (one void per unit cell) for which a discrete atom model could not be obtained. This solvent was modelled using a solvent mask which determined a volume of 165.5 cubic angstroms containing an estimated 43 electrons. This would be consistent with the presence of either a pentane, a dichloromethane or a mixture of the two.

Table 23: X-Ray Diffraction Data for the $Pd_2(Br)_2(C_6H_4F)_2[P(\textit{o}\text{-tolyl})_3]_2$ oxidative addition dimer SI45

**Identification code**	**ijsf21047**
**Empirical formula**	$C_{54}H_{50}Br_2F_2P_2Pd_2$
**Formula weight**	1171.50
**Temperature/K**	110.00(10)
**Crystal system**	triclinic

**Space group**	P-1
**a/Å**	10.3478(5)
**b/Å**	10.9173(6)
**c/Å**	11.2204(4)
**α/°**	84.256(4)
**β/°**	88.306(3)
**γ/°**	84.946(4)
**Volume/Å^3**	1256.02(10)
**Z**	1
**ρ_{calc}g/cm^3**	1.549
**μ/mm^{-1}**	8.570
**F(000)**	584.0
**Crystal size/mm^3**	0.099 × 0.042 × 0.016
**Radiation**	Cu Kα (λ = 1.54184)
**2Θ range for data collection/°**	7.922 to 134.16
**Index ranges**	-12 ≤ h ≤ 12, -13 ≤ k ≤ 13, -13 ≤ l ≤ 12
**Reflections collected**	8328
**Independent reflections**	4472 [R_{int} = 0.0260, R_{sigma} = 0.0383]
**Data/restraints/parameters**	4472/0/283
**Goodness-of-fit on F^2**	1.024
**Final R indexes [I>=2σ (I)]**	R_1 = 0.0289, wR_2 = 0.0673
**Final R indexes [all data]**	R_1 = 0.0379, wR_2 = 0.0713
**Largest diff. peak/hole / e Å^{-3}**	1.05/-0.41

ijsf21041: $[Pd(C^\wedge P)(\mu_2\text{-}Br)]_2$ palladacycle 35 CCDC 2288571 (lab book ref. DRH-02-39)

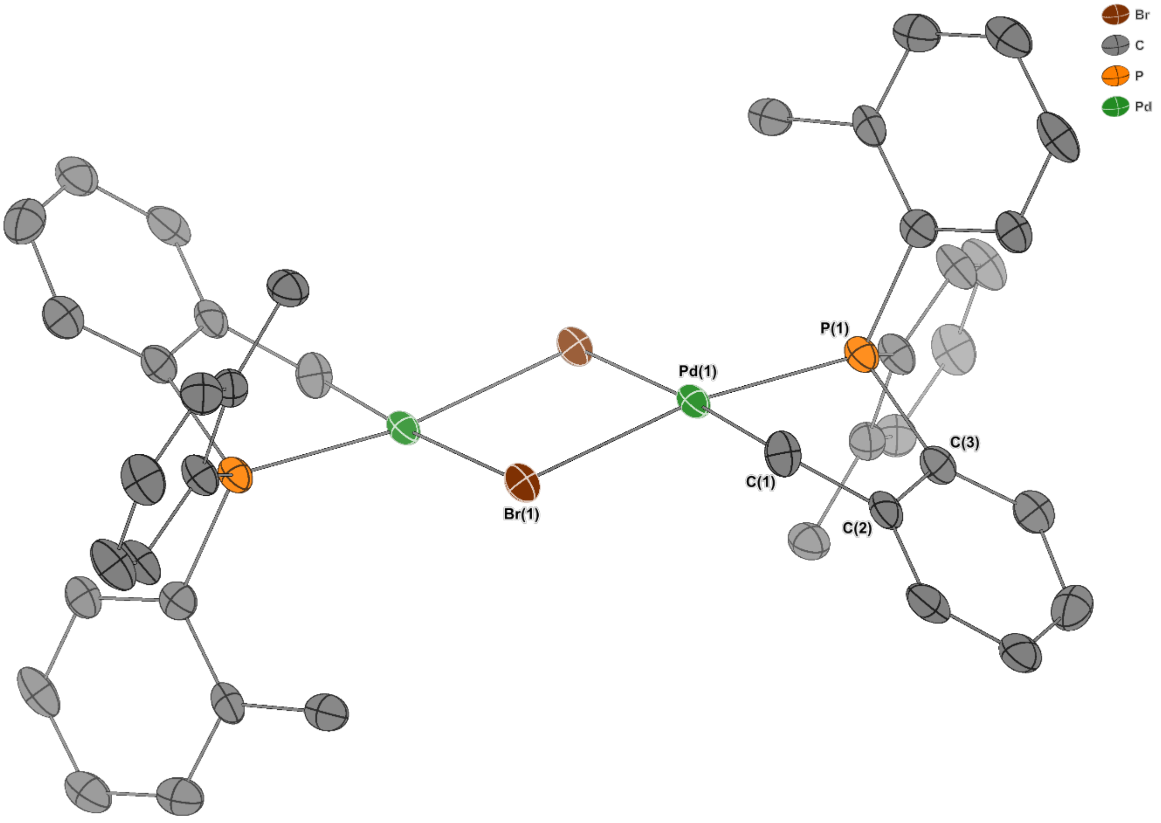

Figure 90: Structure, obtained by X-ray diffraction, of a single crystal of the $[Pd(C^\wedge P)(\mu_2\text{-}Br)]_2$ palladacycle 35 (thermal ellipsoids are set at 50% probability, selected H-atoms are omitted for clarity). Selected interatomic lengths /Å: Pd1-P1 = 2.2480(13); Pd1-Br1 = 2.5227(6); Pd1-C1 = 2.056(5); P1-C3 = 1.811(5). Selected interatomic angles /°: C1-Pd1-P1 = 80.16(15); P1-Pd1-Br1A = 102.42(4); C1-Pd1-Br1 = 91.07(15); Br1-Pd1-Br1A = 86.11(2).

Table 24: X-Ray Diffraction Data for the $[Pd(C^\wedge P)(\mu_2\text{-}Br)]_2$ palladacycle 35

Identification code	ijsf21041
Empirical formula	$C_{42}H_{40}Br_2P_2Pd_2$
Formula weight	979.30
Temperature/K	110.00(10)
Crystal system	monoclinic
Space group	$P2_1/n$
a/Å	9.3533(4)
b/Å	10.4619(4)
c/Å	19.0660(9)
α/°	90
β/°	99.200(4)
γ/°	90
Volume/Å^3	1841.67(14)
Z	2

**ρ_{calc}g/cm^3**	1.766
**μ/mm^{-1}**	11.464
**F(000)**	968.0
**Crystal size/mm^3**	0.076 × 0.045 × 0.032
**Radiation**	Cu Kα (λ = 1.54184)
**2Θ range for data collection/°**	9.398 to 141.864
**Index ranges**	-11 ≤ h ≤ 11, -12 ≤ k ≤ 6, -23 ≤ l ≤ 22
**Reflections collected**	6554
**Independent reflections**	3480 [R_{int} = 0.0358, R_{sigma} = 0.0474]
**Data/restraints/parameters**	3480/0/219
**Goodness-of-fit on F^2**	1.073
**Final R indexes [I>=2σ (I)]**	R_1 = 0.0457, wR_2 = 0.1169
**Final R indexes [all data]**	R_1 = 0.0524, wR_2 = 0.1229
**Largest diff. peak/hole / e Å^{-3}**	2.14/-1.16

ijsf22048: [Pd(C^P)(μ_2-Ar^F)]$_2$ palladacycle 16a CCDC 2288573 (lab book ref. DRH-03-79)

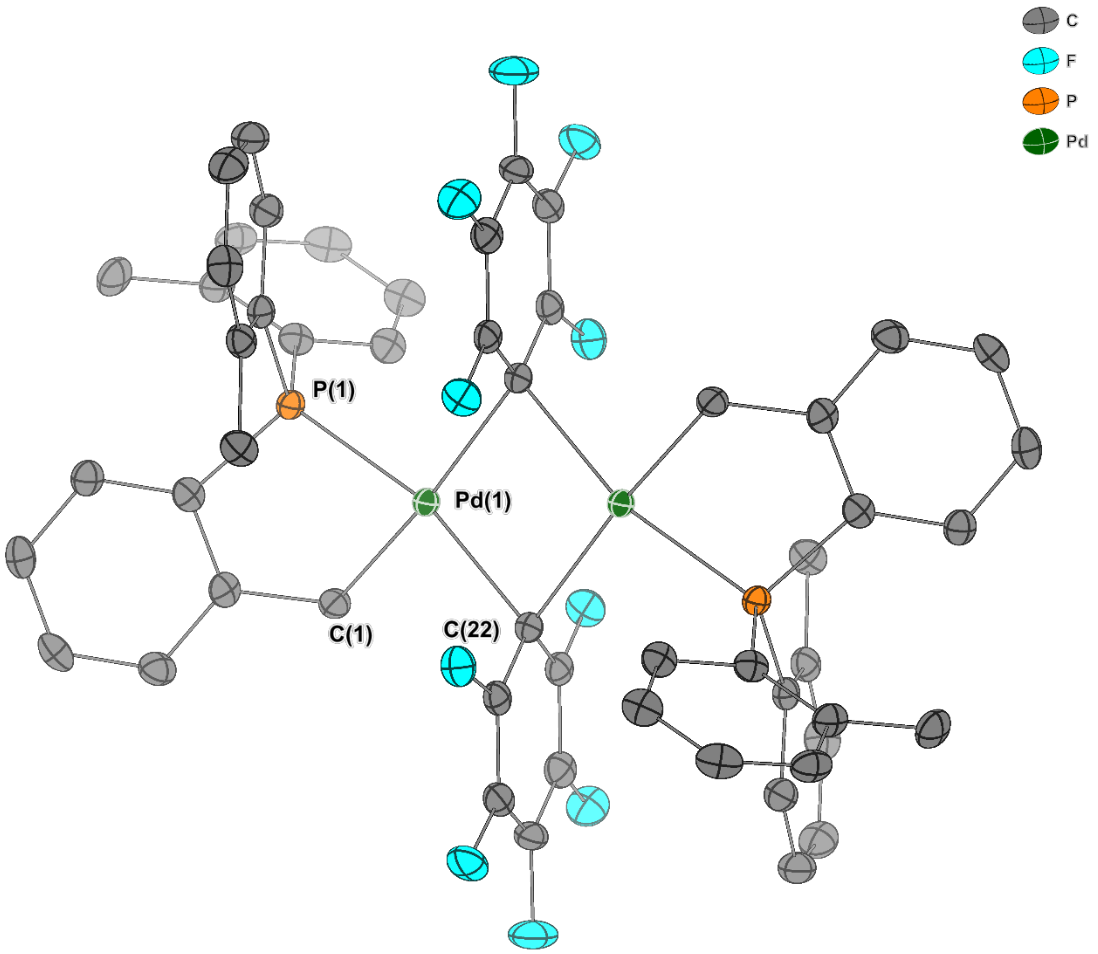

Figure 91: Structure, obtained by X-ray diffraction, of a single crystal of [Pd(C^P)(μ_2-Ar^F)]$_2$ palladacycle 16a (thermal ellipsoids are set at 50% probability, H-atoms are omitted for clarity). Selected interatomic lengths /Å: Pd1-Pd1^1 = 2.8391(3), Pd1-C22 = 2.180(2), Pd1-P1 = 2.2681(5), Pd1-C1 = 2.081(2), Pd1-C22^1 = 2.268(2). Selected interatomic angles /°: C22^1-Pd1-P1 = 94.78(5), P1-Pd1-C1 = 79.84(6), C1-Pd1-C22 = 84.84(8), C22-Pd1-C22^1 = 100.69(7).

Table 25: X-Ray Diffraction Data for [Pd(C^P)(μ_2-Ar^F)]$_2$ palladacycle 16a

Identification code	ijsf22048
Empirical formula	$C_{54}H_{40}F_{10}P_2Pd_2$
Formula weight	1153.60
Temperature/K	110.00(10)
Crystal system	monoclinic
Space group	$P2_1/n$
a/Å	12.62840(10)
b/Å	14.50970(10)
c/Å	12.69150(10)
α/°	90
β/°	108.3000(10)

**γ/°**	90
**Volume/Å^3**	2207.91(3)
**Z**	2
**ρ_{calc}g/cm^3**	1.735
**μ/mm^{-1}**	7.973
**F(000)**	1152.0
**Crystal size/mm^3**	0.19 × 0.12 × 0.08
**Radiation**	Cu Kα (λ = 1.54184)
**2Θ range for data collection/°**	8.618 to 153.94
**Index ranges**	-15 ≤ h ≤ 15, -6 ≤ k ≤ 18, -15 ≤ l ≤ 16
**Reflections collected**	13847
**Independent reflections**	4487 [R_{int} = 0.0324, R_{sigma} = 0.0325]
**Data/restraints/parameters**	4487/0/309
**Goodness-of-fit on F^2**	1.044
**Final R indexes [I>=2σ (I)]**	R_1 = 0.0254, wR_2 = 0.0658
**Final R indexes [all data]**	R_1 = 0.0267, wR_2 = 0.0668
**Largest diff. peak/hole / e Å^{-3}**	0.81/-0.68

ijsf23080: [Pd(P^C)(μ_2-I)]$_2$ palladacycle 32 CCDC 2313317 (lab book ref. DRH-03-83-2)

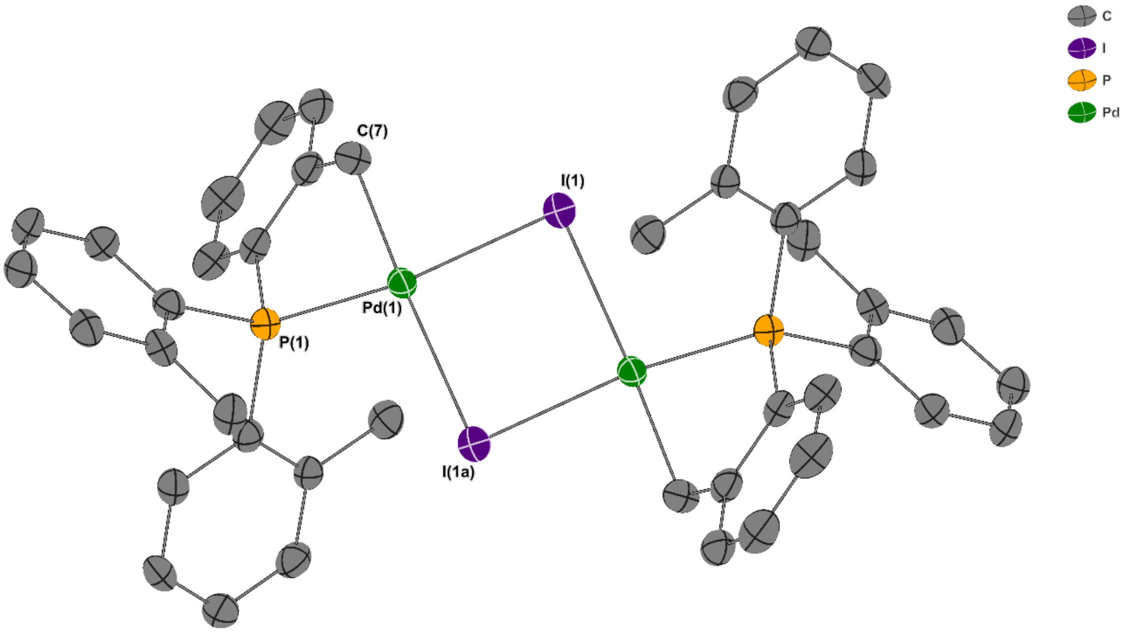

Figure 92: Structure, obtained by X-ray diffraction, of a single crystal of [Pd(P^C)(μ_2-I)]$_2$ palladacycle 32 (thermal ellipsoids are set at 50% probability, H-atoms are omitted for clarity). Selected interatomic lengths /Å: Pd1-I1 = 2.6430(5); Pd1-I1a = 2.7022(5); Pd1-C7 = 2.085(5); Pd1-P1 = 2.2575(13); Selected interatomic angles /°: I1a-Pd1-P1 = 101.69(3); C7-Pd1-P1 = 79.76(16); C7-Pd1-I1 = 90.77(15); I1-Pd1-I1a = 87.431(14).

This crystal structure was isolated from the reaction of **16a** with aryl iodide **31**. It confirms the presence of [Pd(P^C)(μ_2-I)]$_2$ palladacycle as the endpoint for this reaction, corroborating the ^{31}P NMR data and LIFDI mass spec data (see section 7.9).

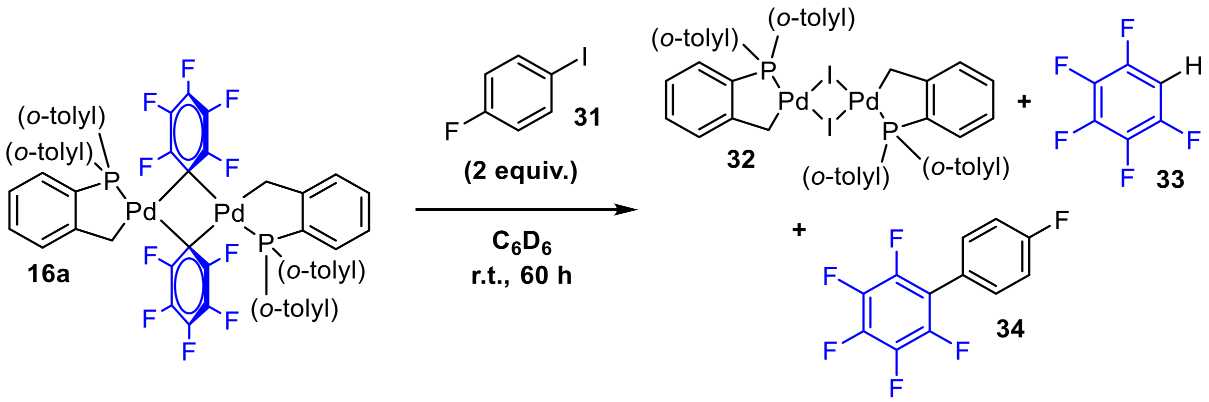

Table 26: X-Ray data for [Pd(P^C)(μ_2-I)]$_2$ palladacycle 32

Identification code	ijsf23080
Empirical formula	$C_{42}H_{40}P_2Pd_2I_2$
Formula weight	1073.28

**Temperature/K**	109.95(10)
**Crystal system**	monoclinic
**Space group**	$P2_1/n$
**a/Å**	9.39283(9)
**b/Å**	10.67677(13)
**c/Å**	19.0250(2)
**α/°**	90
**β/°**	99.4160(10)
**γ/°**	90
**Volume/Å^3**	1882.21(4)
**Z**	2
**ρ_{calc}g/cm^3**	1.894
**μ/mm^{-1}**	21.609
**F(000)**	1040.0
**Crystal size/mm^3**	0.085 × 0.063 × 0.045
**Radiation**	Cu Kα (λ = 1.54184)
**2Θ range for data collection/°**	9.424 to 136.502
**Index ranges**	-11 ≤ h ≤ 11, -11 ≤ k ≤ 12, -19 ≤ l ≤ 22
**Reflections collected**	17522
**Independent reflections**	3432 [R_{int} = 0.0361, R_{sigma} = 0.0268]
**Data/restraints/parameters**	3432/0/219
**Goodness-of-fit on F^2**	1.053
**Final R indexes [I>=2σ (I)]**	R_1 = 0.0322, wR_2 = 0.0931
**Final R indexes [all data]**	R_1 = 0.0357, wR_2 = 0.0954
**Largest diff. peak/hole / e Å^{-3}**	1.36/-1.21

9. NMR Spectral Data for Organic and Inorganic Compounds

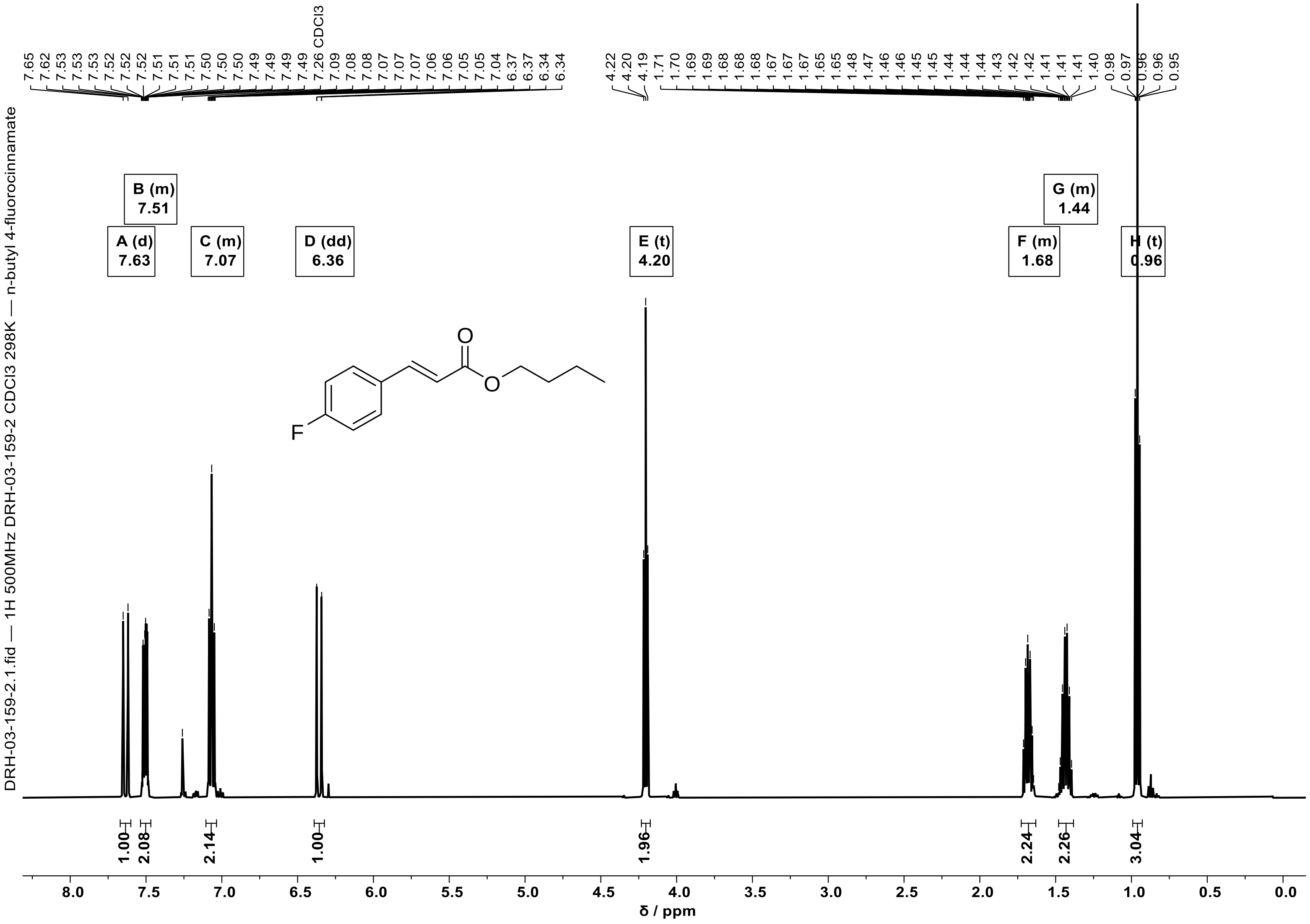

Figure 93: ^{1}H NMR (500 MHz, C*D*Cl$_3$, 32 scans, 298 K) spectrum of n-butyl 4-fluorocinnamate SI42. Lab book ref. DRH-03-159

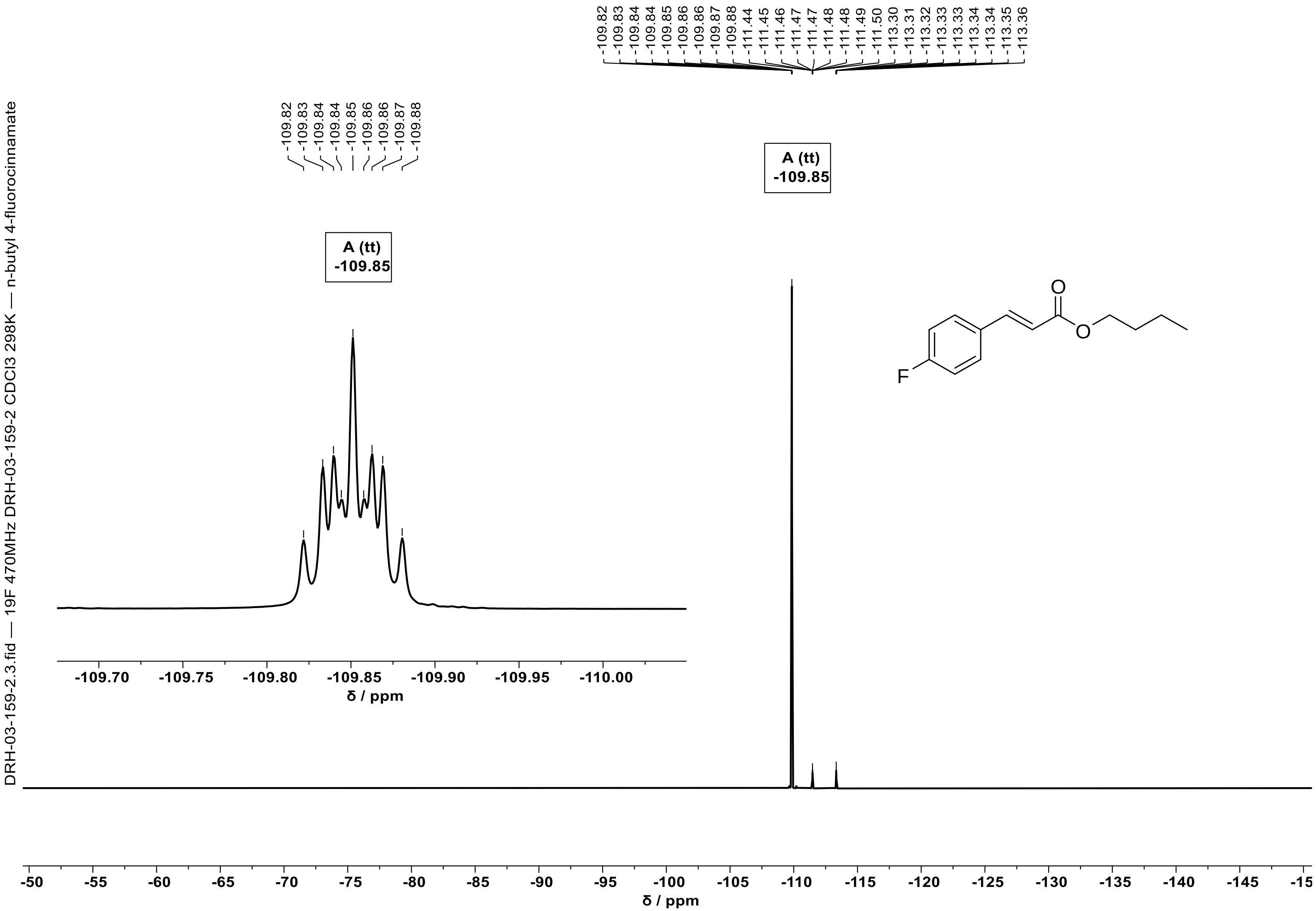

Figure 94: ^{19}**F NMR (470 MHz, C*D*Cl$_3$, 64 scans, 298 K) spectrum of n-butyl 4-fluorocinnamate SI42. Lab book ref. DRH-03-159**

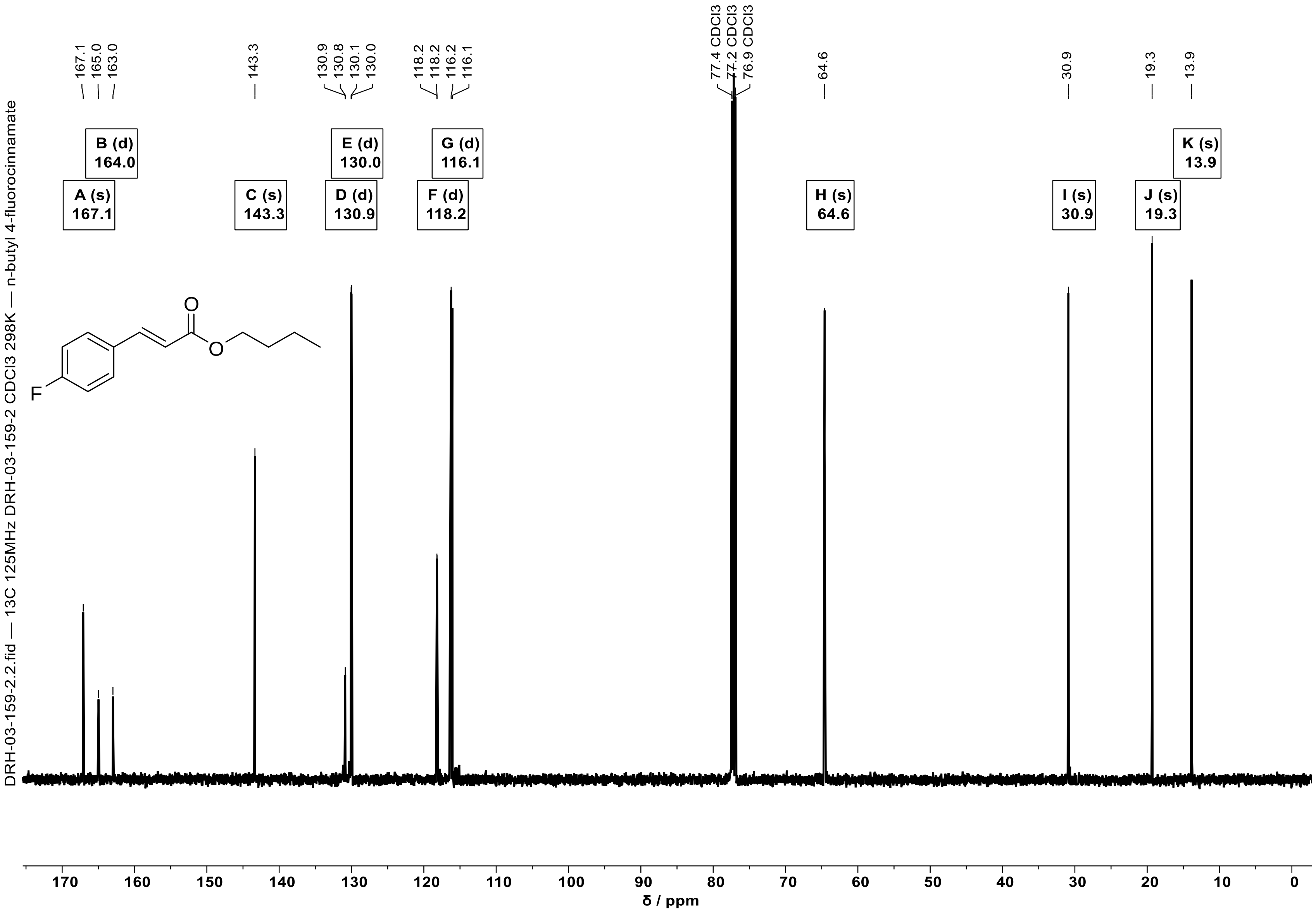

Figure 95: ^{13}C NMR (125 MHz, C*D*Cl$_3$, 128 scans, 298 K) spectrum of n-butyl 4-fluorocinnamate SI42. Lab book ref. DRH-03-159

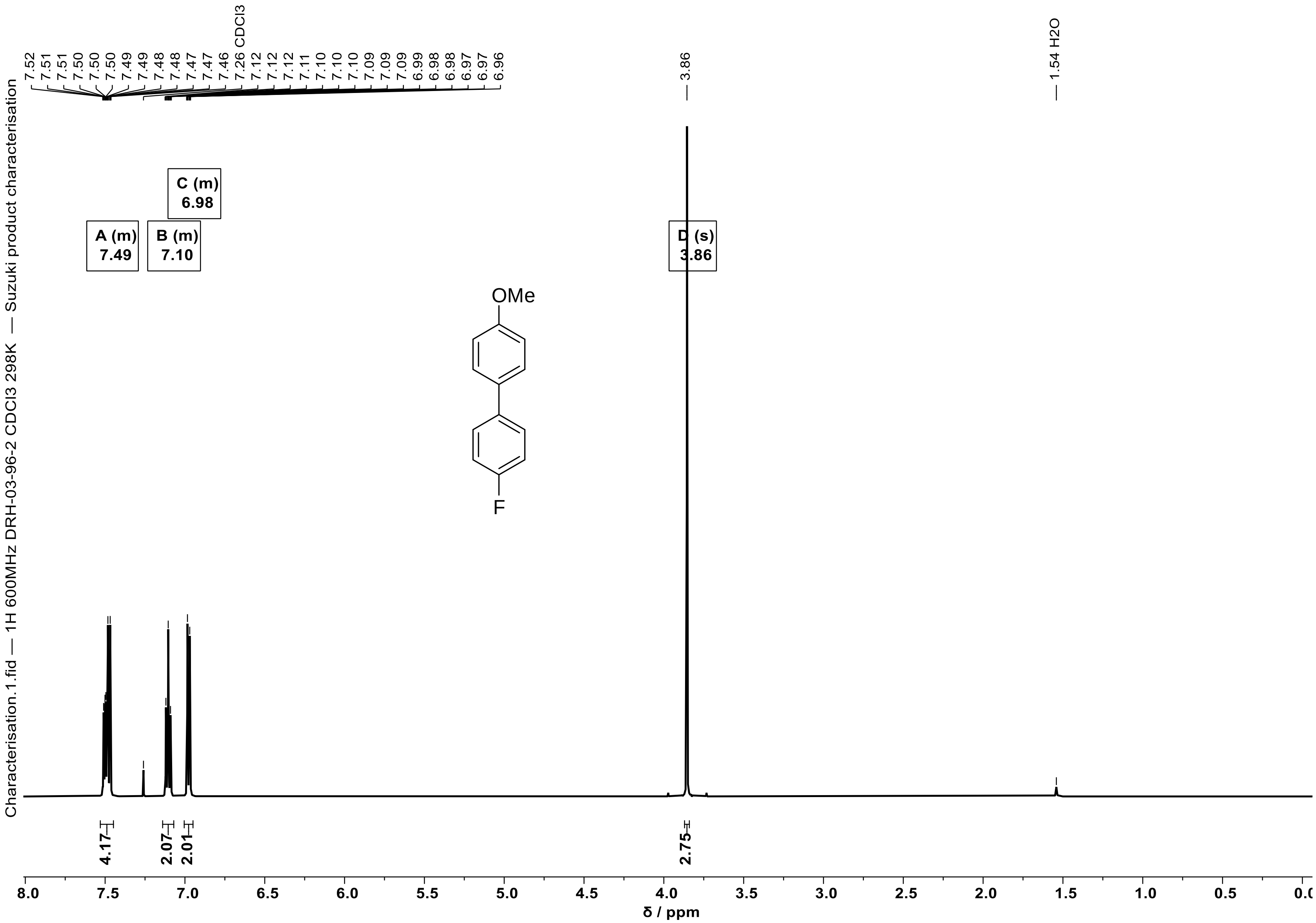

Figure 96: ^{1}H NMR (600 MHz, C*D*Cl$_3$, 16 scans, 10s relaxation delay 298 K) spectrum of 4-(4-fluorophenyl)anisole 5. Lab book ref. DRH-03-96-3

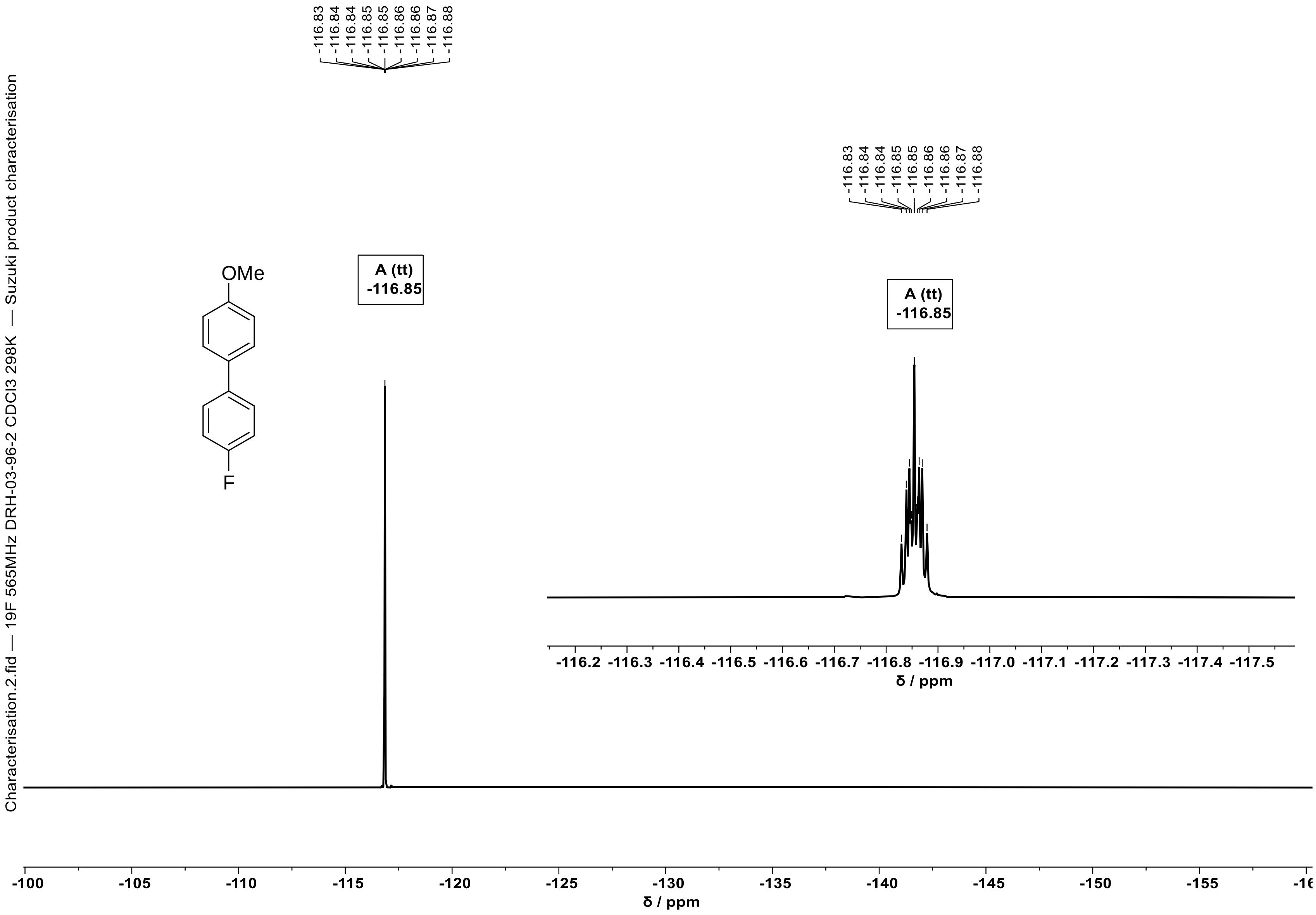

Figure 97: ^{19}F NMR (565 MHz, C*D*Cl$_3$, 64 scans, 298 K) spectrum of 4-(4-fluorophenyl)anisole 5. Lab book ref. DRH-03-96-3

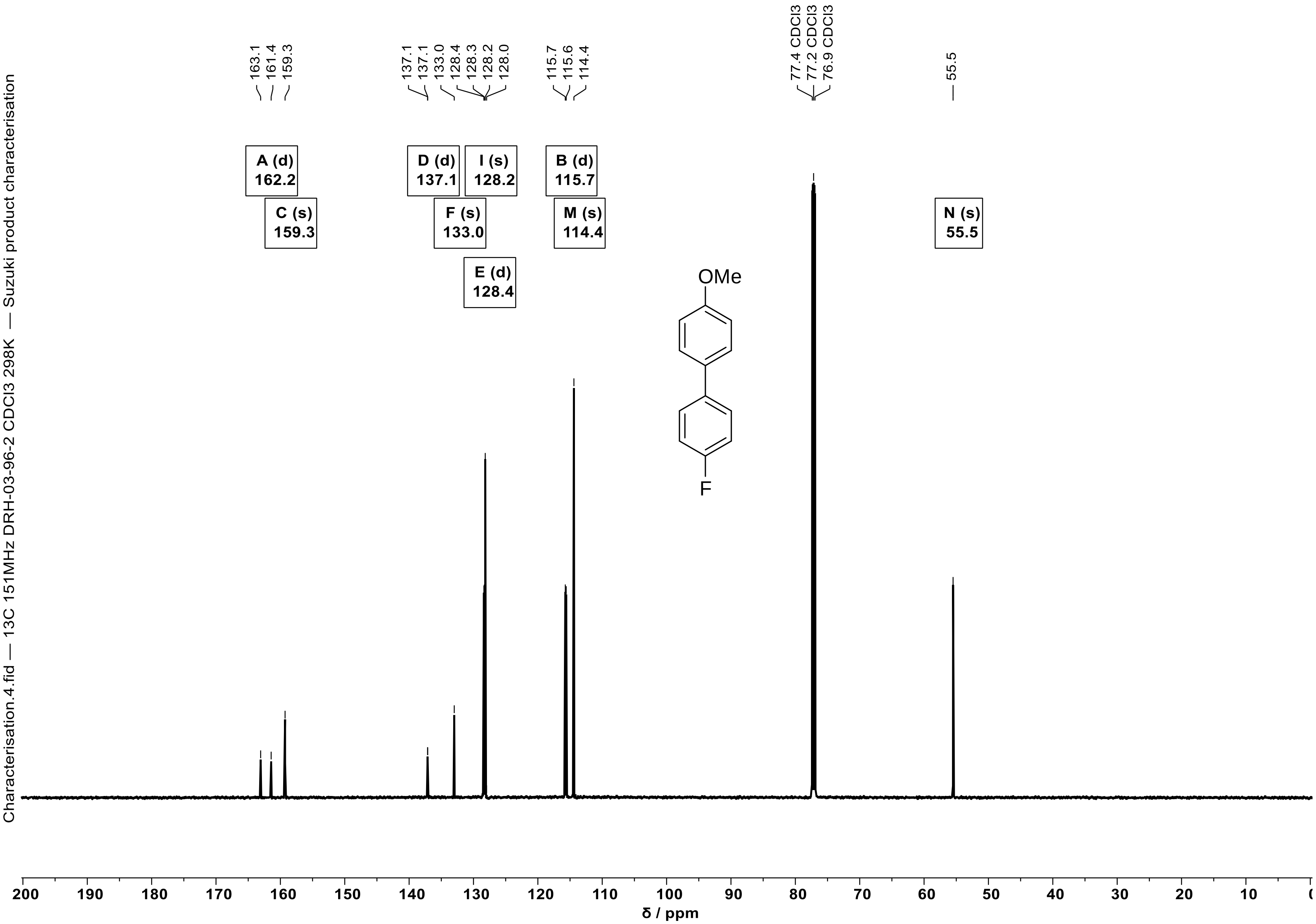

Figure 98: ^{13}C NMR (151 MHz, C*D*Cl$_3$, 1024 scans, 298 K) spectrum of 4-(4-fluorophenyl)anisole 5. Lab book ref. DRH-03-96-3

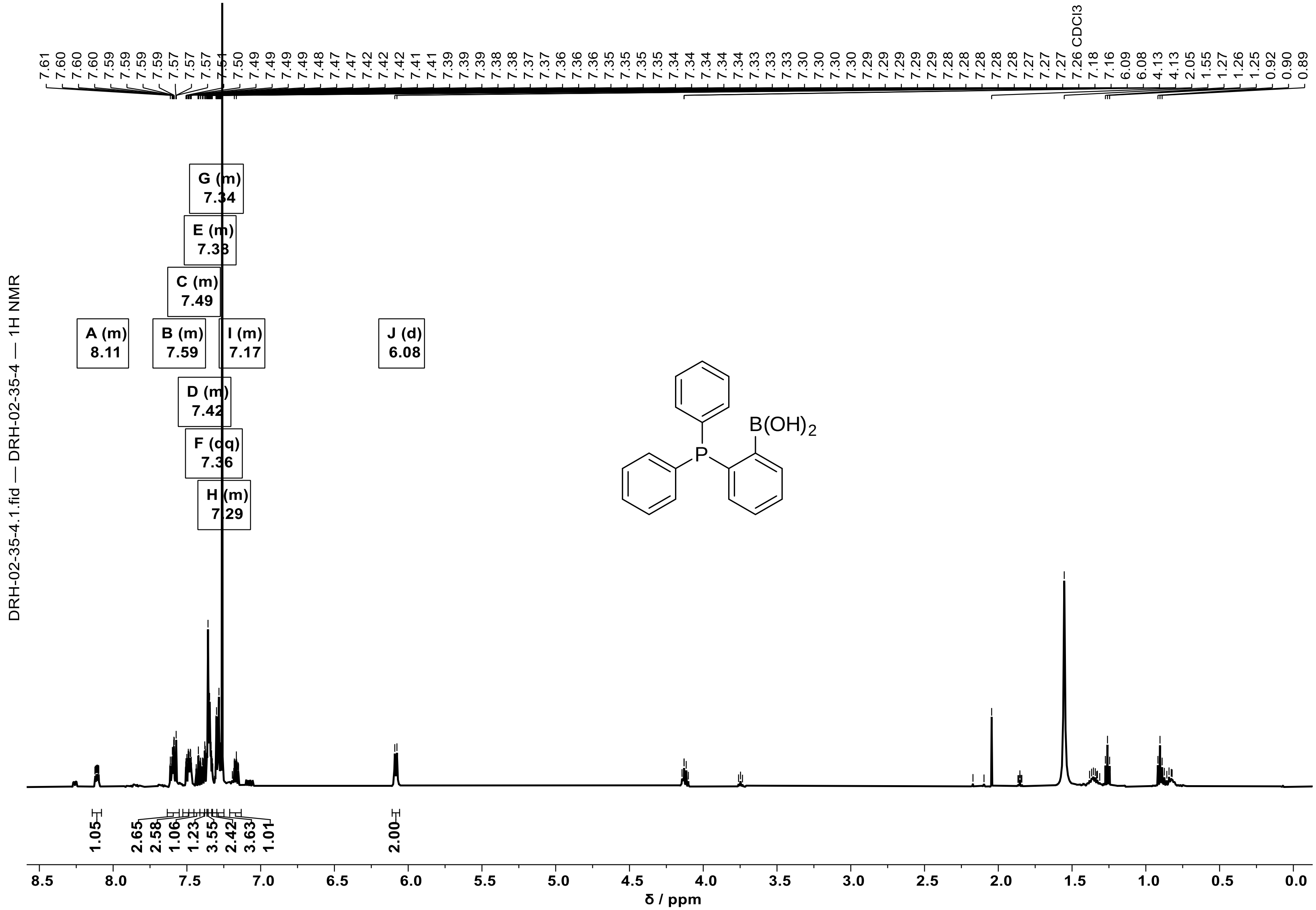

Figure 99: ^{1}H NMR (500 MHz, C*D*Cl$_3$, 64 scans, 298 K) spectrum of PPh$_2$(2-boronic acid phenyl) 19. Peaks below 4.5 ppm are residual solvents (likely water, ethyl acetate, fructose), so have not been integrated. Lab book ref. DRH-02-35-4

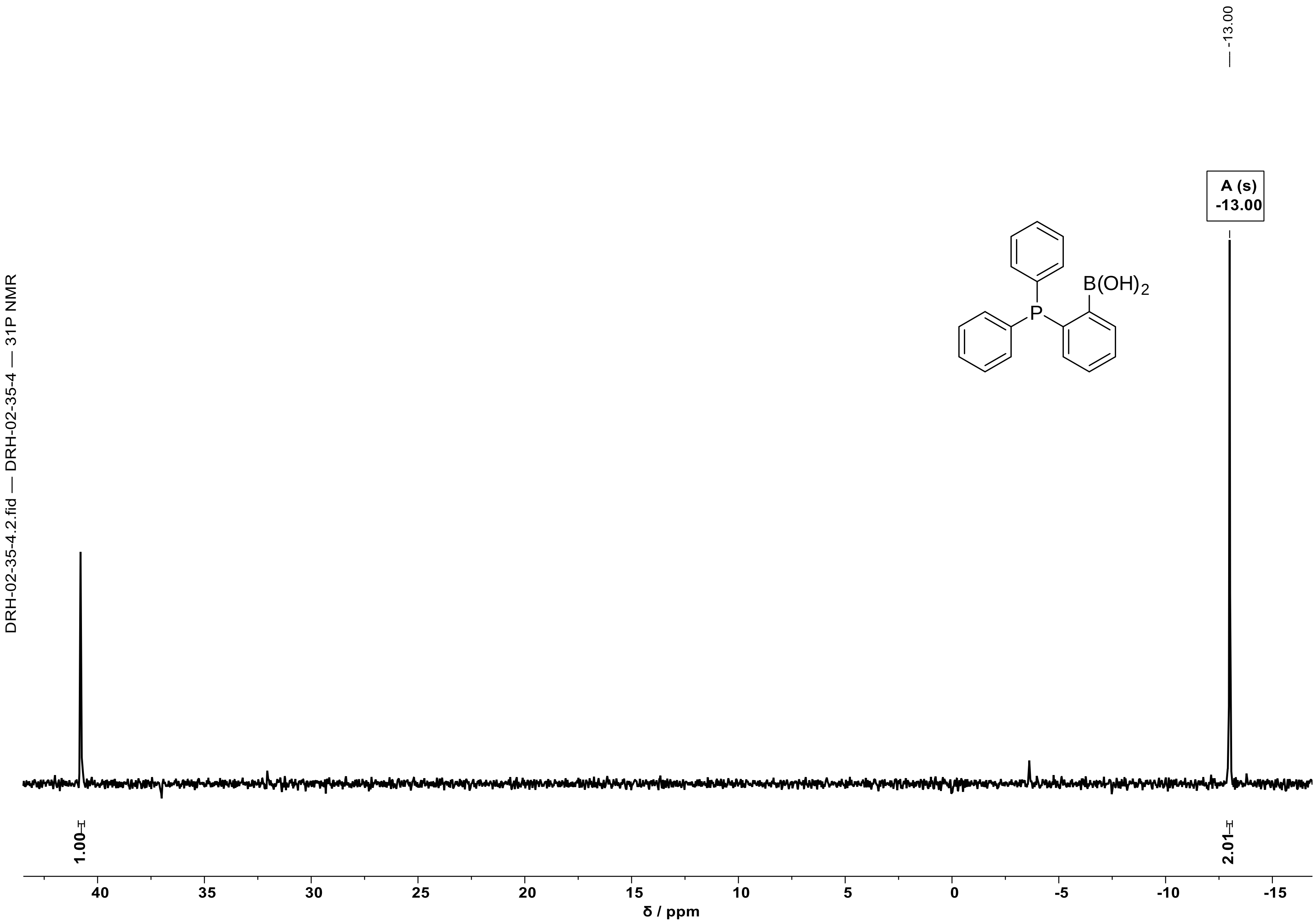

Figure 100: ^{31}P NMR (203 MHz, C*D*Cl$_3$, 64 scans, 298 K) spectrum of PPh$_2$(2-boronic acid phenyl) 19. Lab book ref. DRH-02-35-4

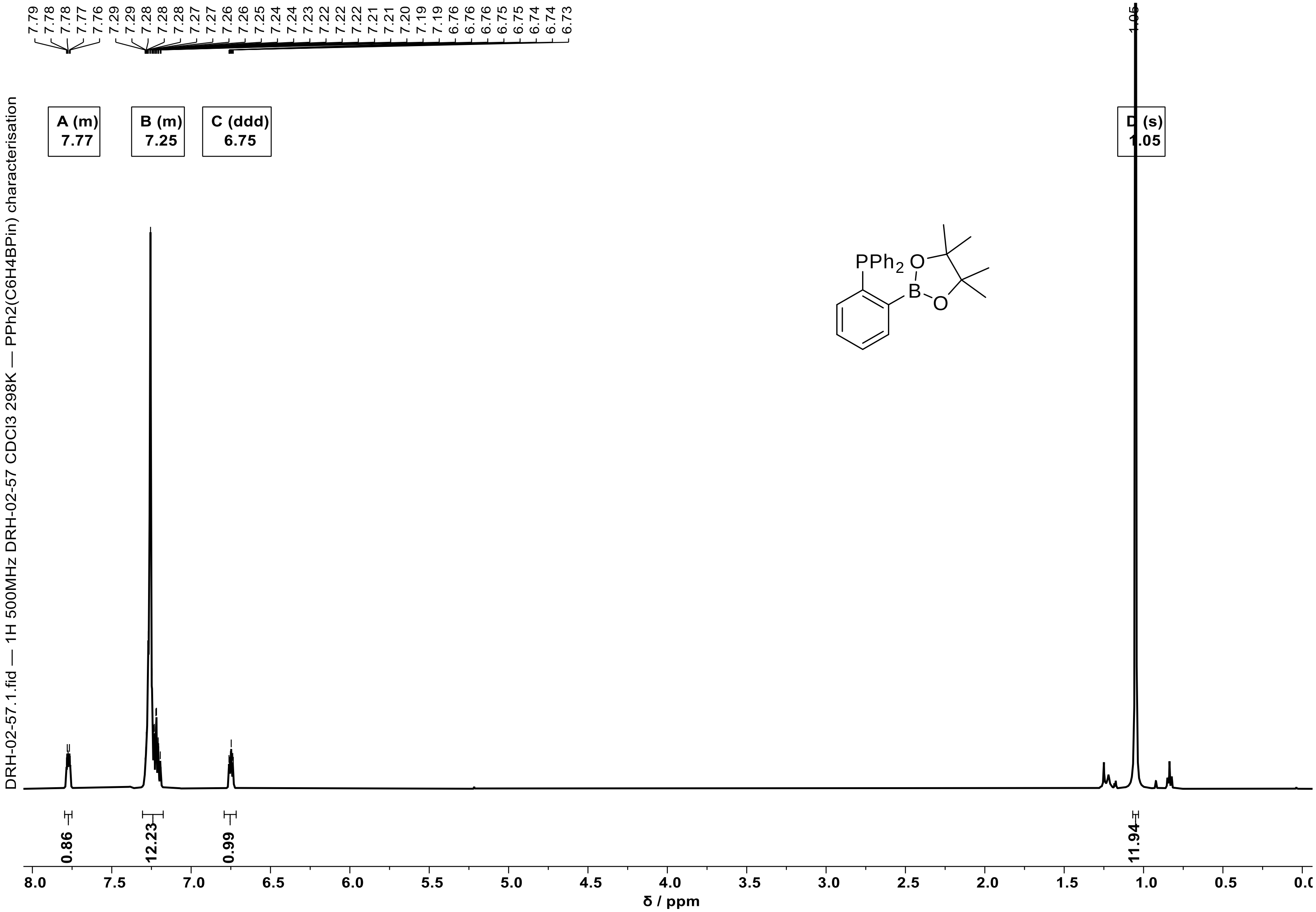

Figure 101: ^{1}H NMR (500 MHz, C*D*Cl_3, 32 scans, 298 K) spectrum of PPh_2(2-pinacol ester phenyl) 22. Lab book ref. DRH-02-57

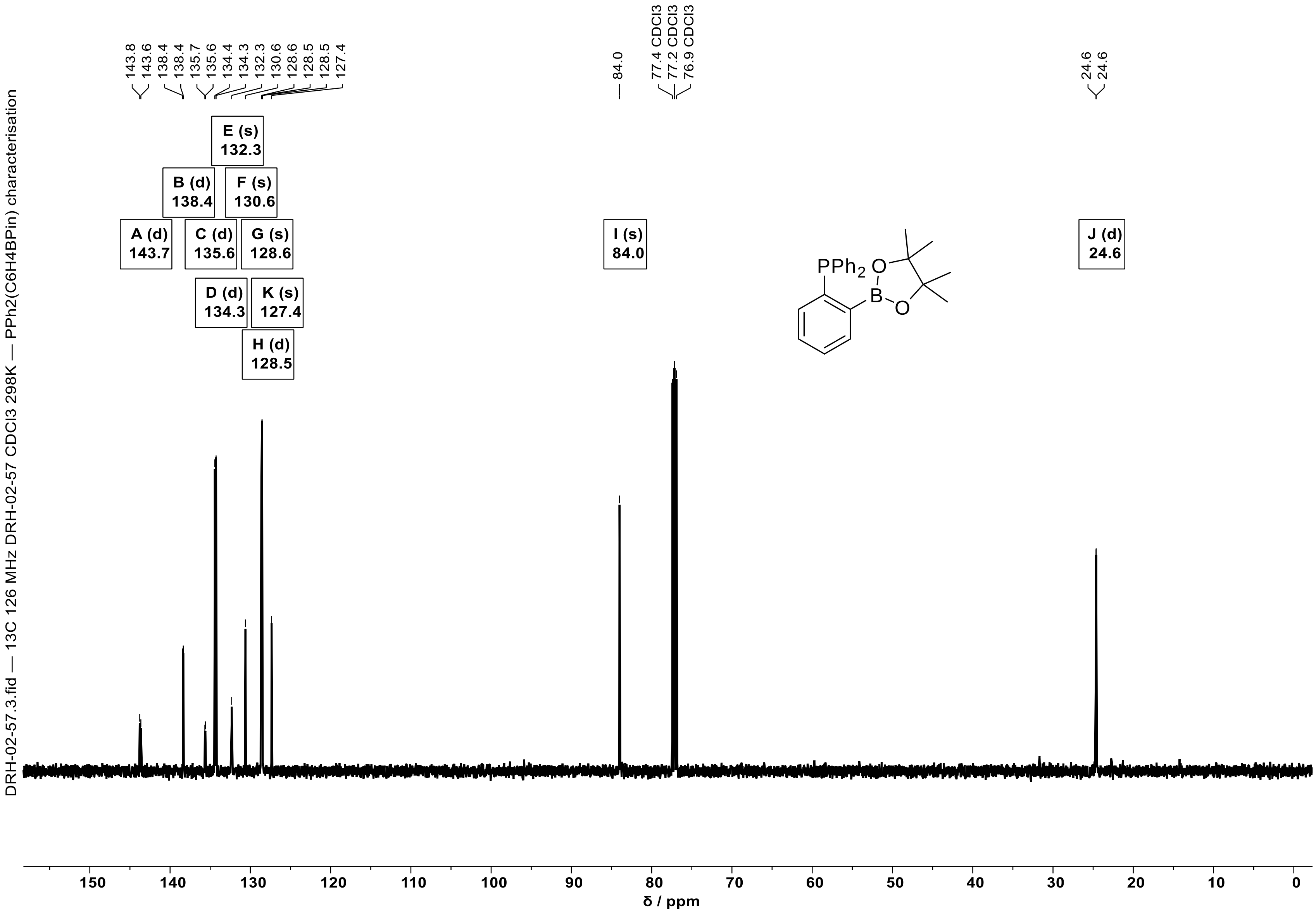

Figure 102: ^{13}**C NMR (126 MHz, C*D*Cl$_3$, 128 scans, 298 K) spectrum of PPh$_2$(2-pinacol ester phenyl) 22. Lab book ref. DRH-02-57**

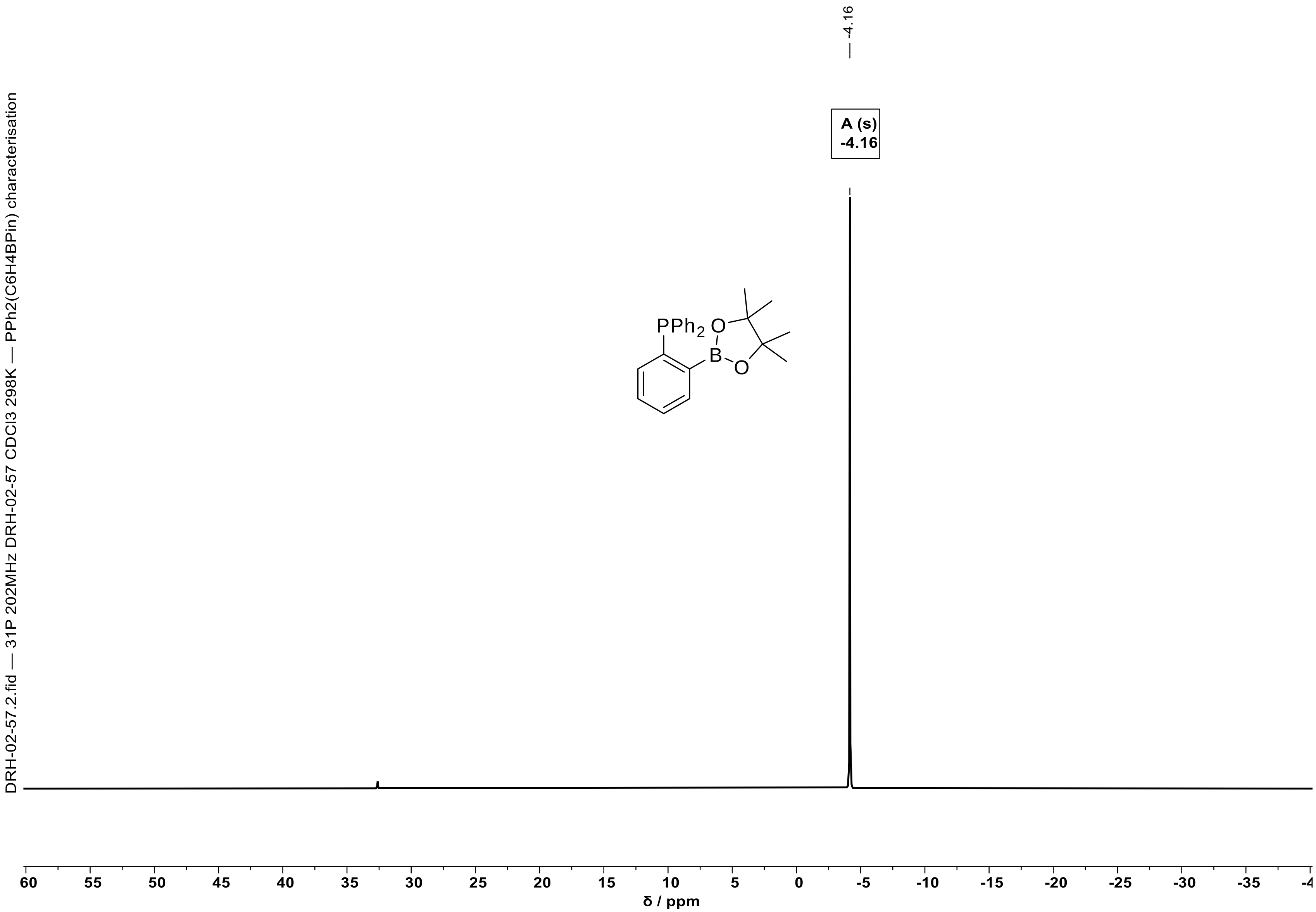

Figure 103: ^{31}**P NMR (203 MHz, C*D*Cl$_3$, 64 scans, 298 K) spectrum of PPh$_2$(2-pinacol ester phenyl) 22. Lab book ref. DRH-02-57**

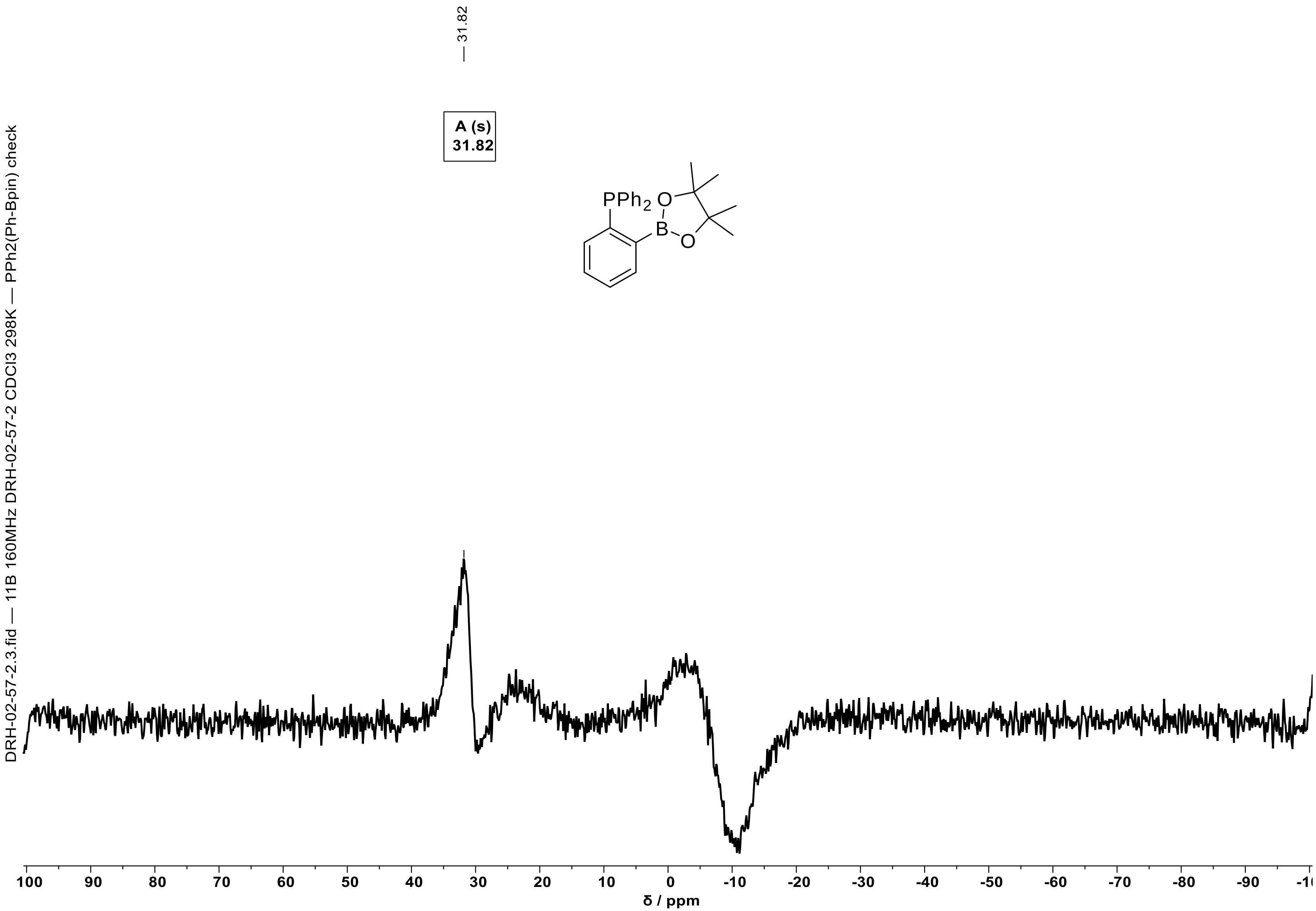

Figure 104: ^{11}B NMR (160 MHz, C*D*Cl$_3$, 256 scans, 298 K) spectrum of PPh$_2$(2-pinacol ester phenyl) 22. Lab book ref. DRH-02-57

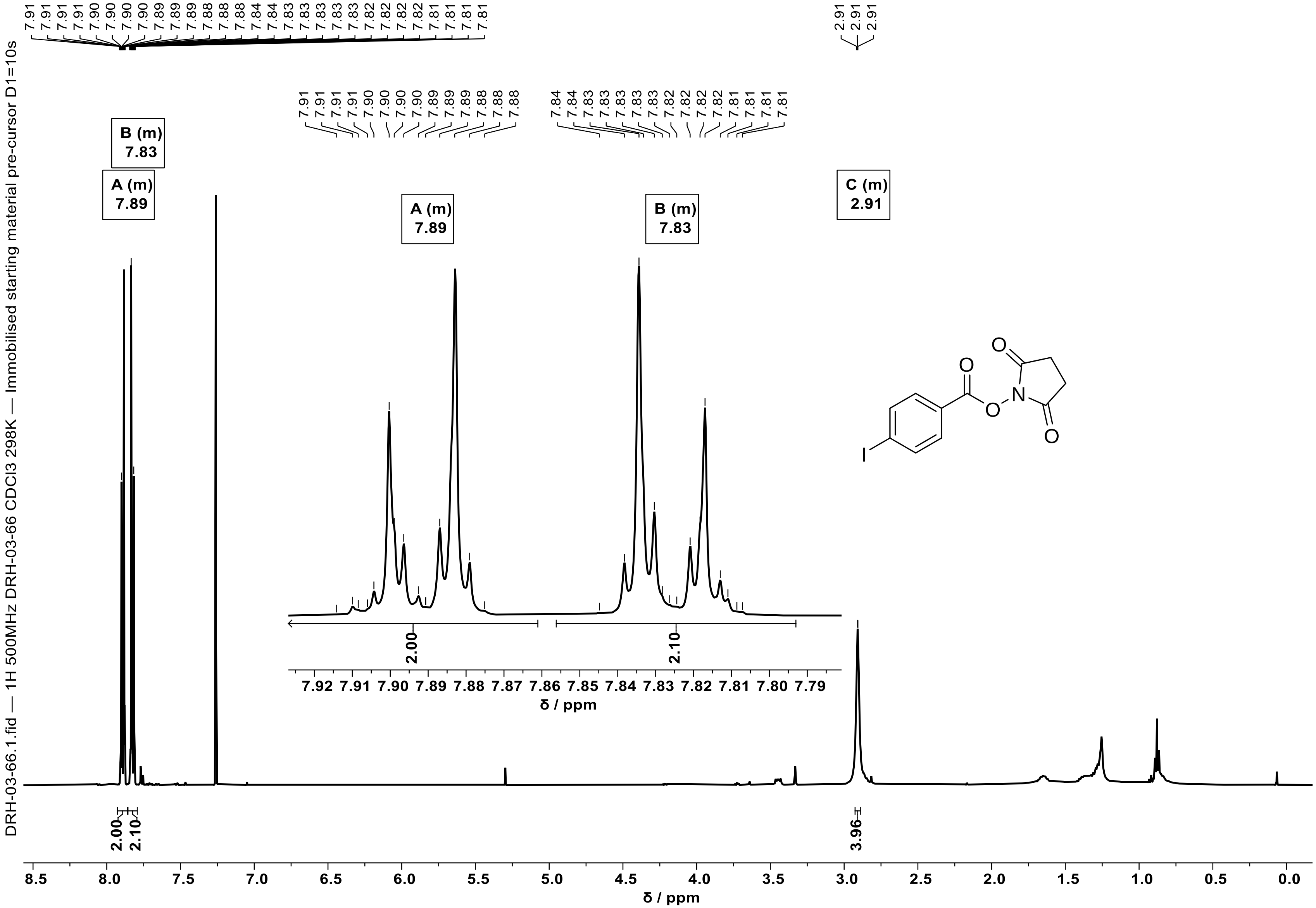

Figure 105: ¹H NMR (500 MHz, CDCl$_3$, 16 scans, 10 s relaxation delay, 298 K) spectrum of the 3-phase test resin precursor SI46. Lab book ref. DRH-03-66

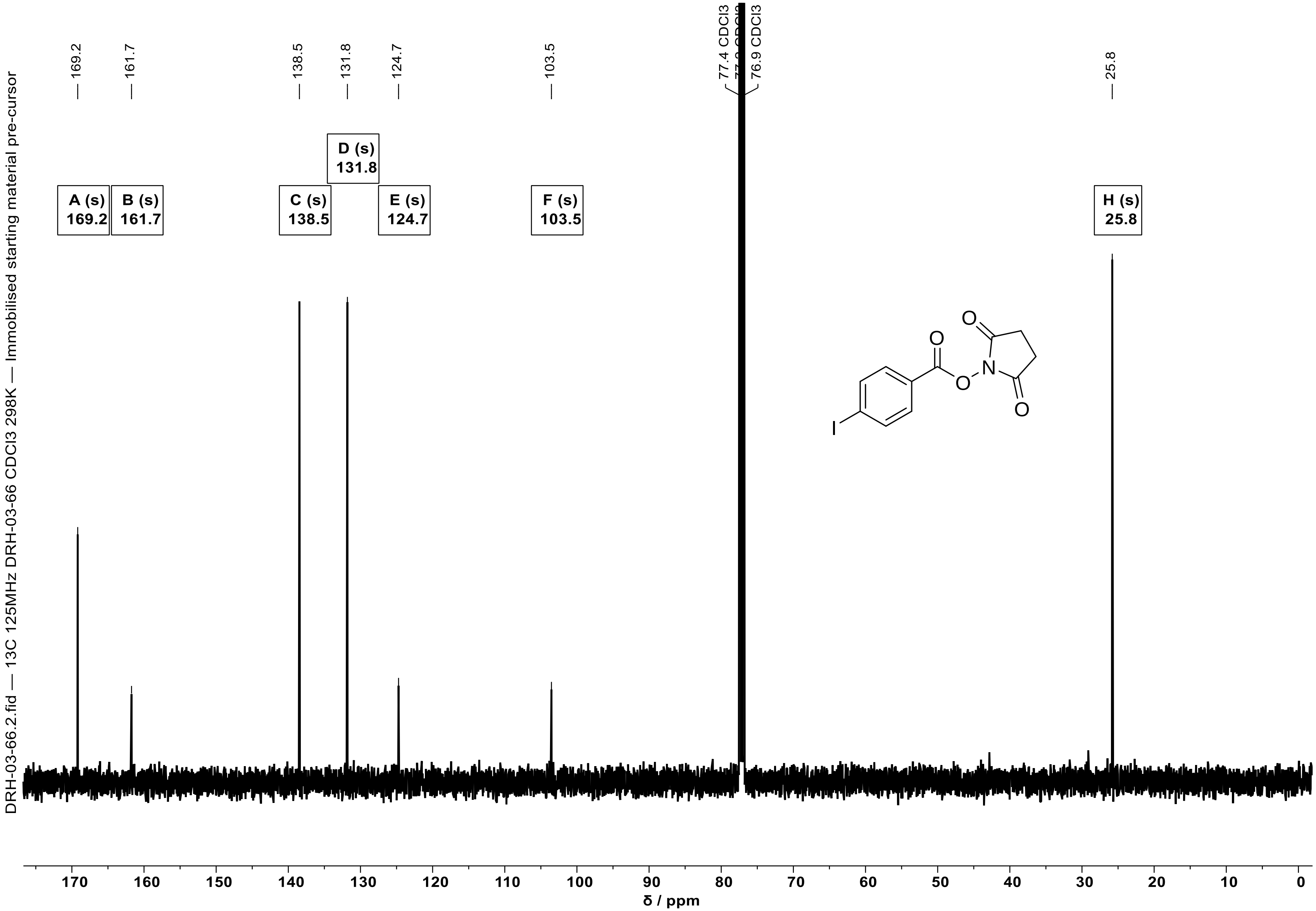

Figure 106: ^{13}C NMR (126 MHz, C*D*Cl$_3$, 128 scans, 298 K) spectrum of the 3-phase test resin precursor SI46. Lab book ref. DRH-03-66

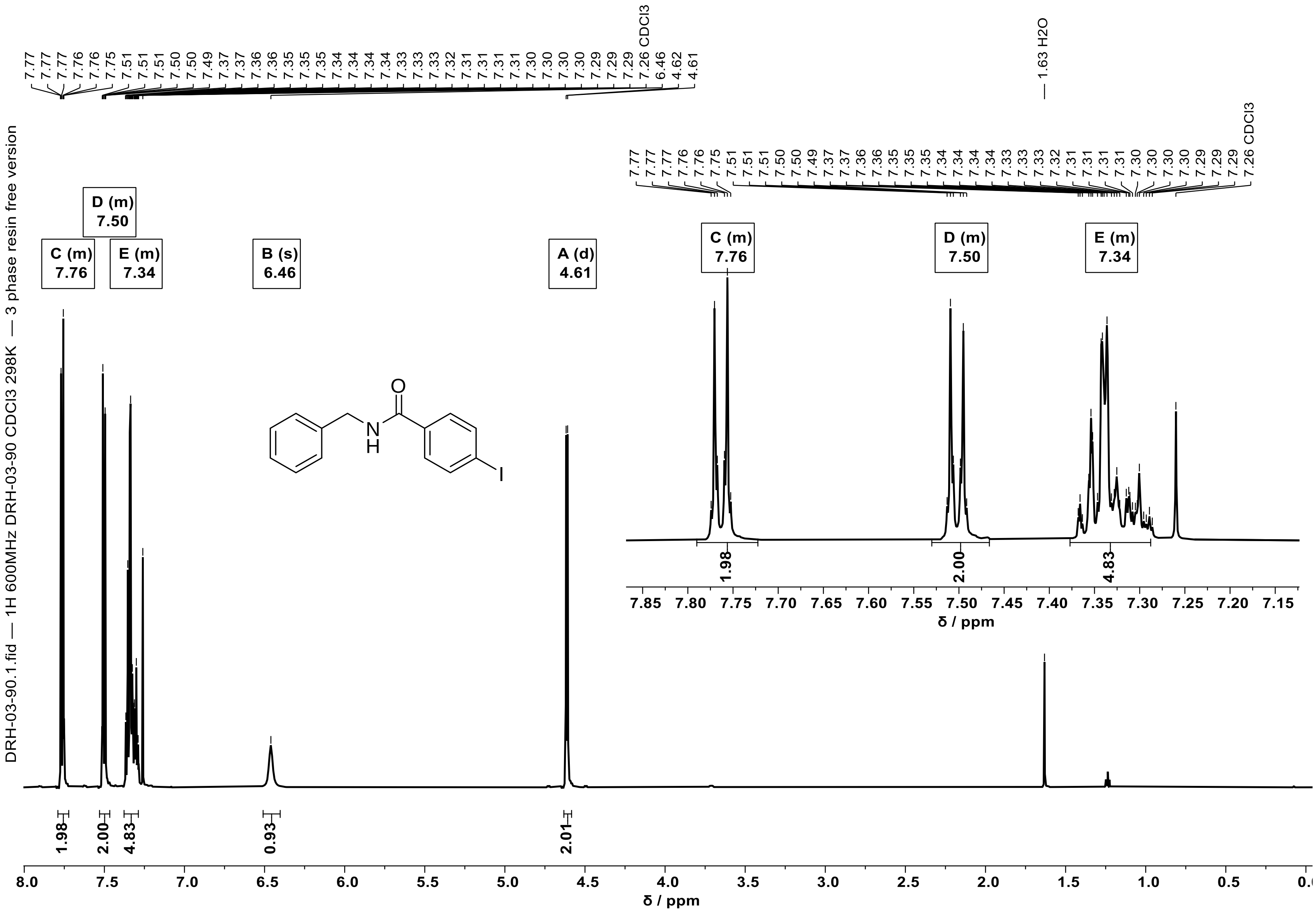

Figure 107: ^{1}H NMR (600 MHz, C*D*Cl$_3$, 16 scans, 298 K) spectrum of the 3-phase test resin (unbound version) 29. Lab book ref. DRH-03-90

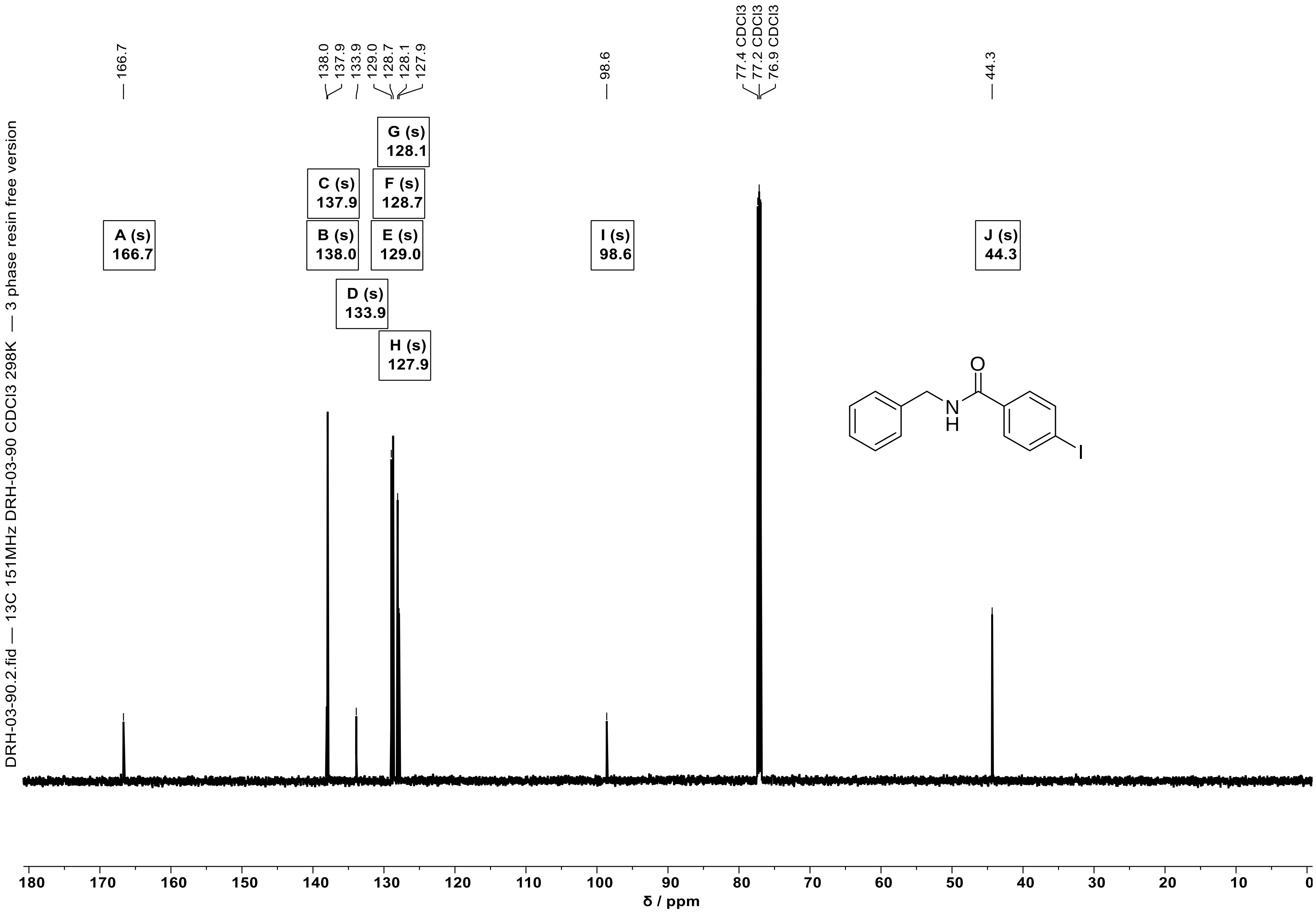

Figure 108: ^{13}C NMR (151 MHz, C*D*Cl$_3$, 128 scans, 298 K) spectrum of the 3-phase test resin (unbound version) 29. Lab book ref. DRH-03-90

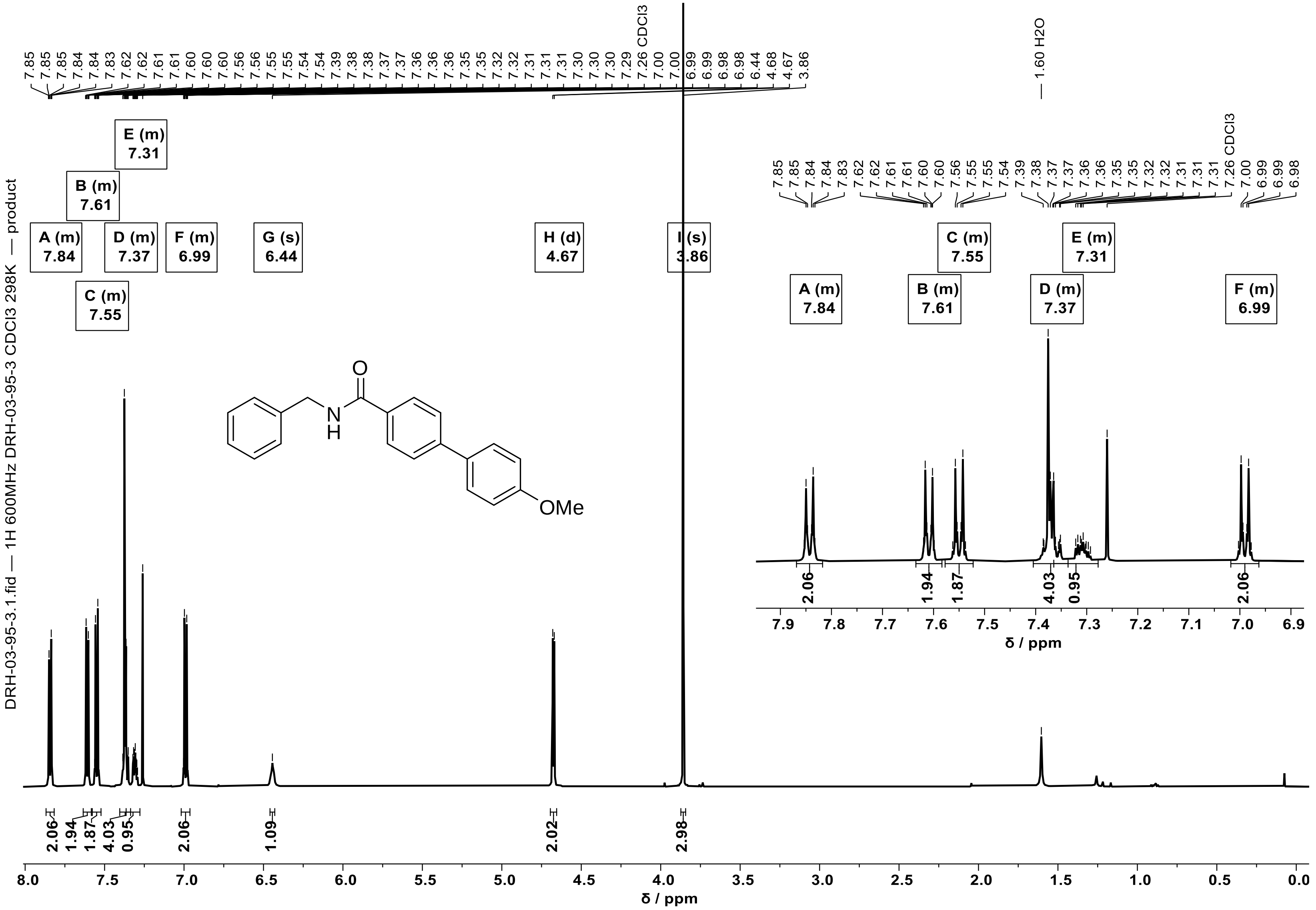

Figure 109: ^{1}H NMR (600 MHz, CDCl$_3$, 32 scans, 298 K) spectrum of the 3-phase test resin cross-coupled product (unbound version) 30. Lab book ref. DRH-03-95-3

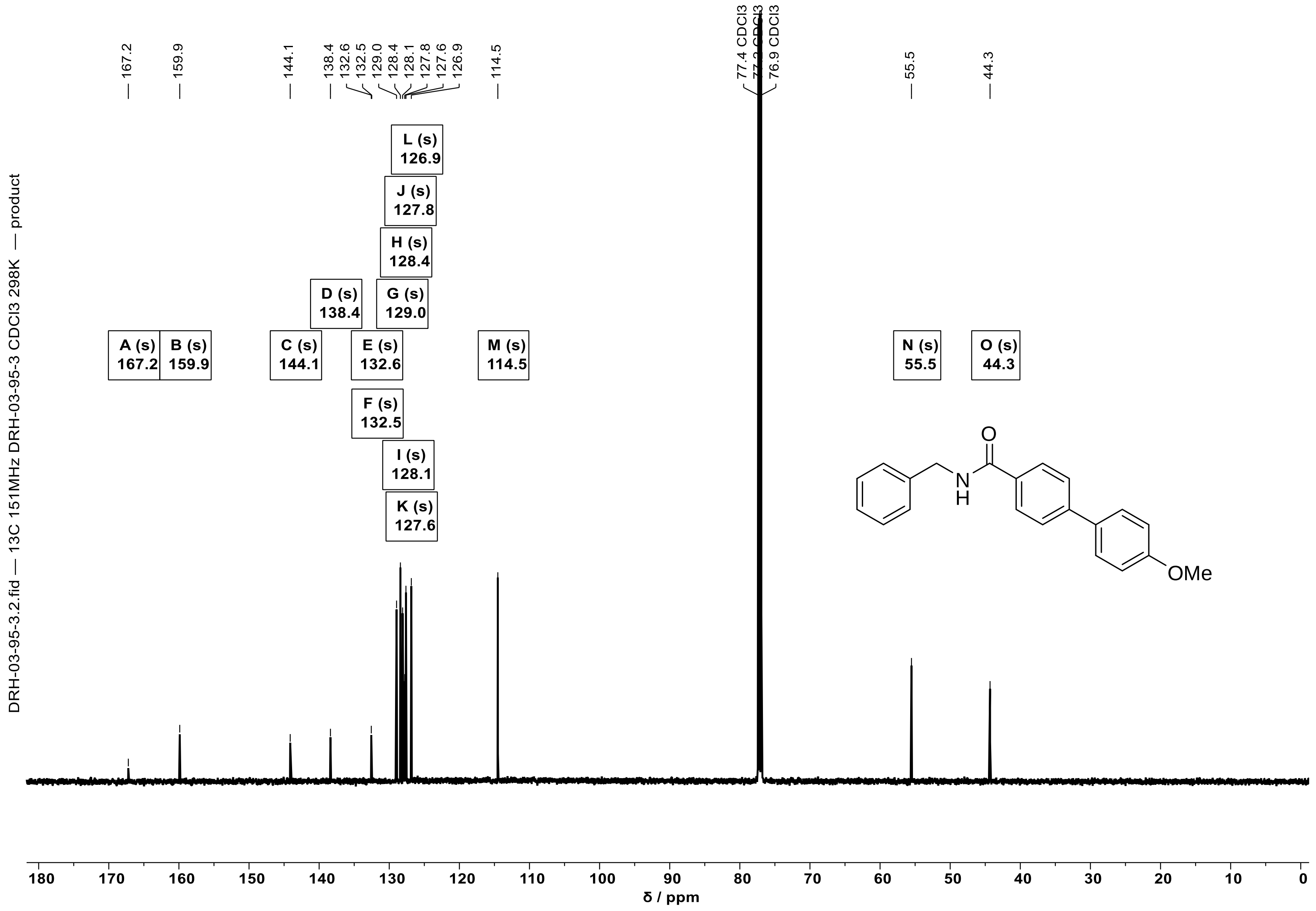

Figure 110: ^{13}C NMR (151 MHz, C*D*Cl$_3$, 512 scans, 298 K) spectrum of the 3-phase test resin cross-coupled product (unbound version) 30. Lab book ref. DRH-03-95-3

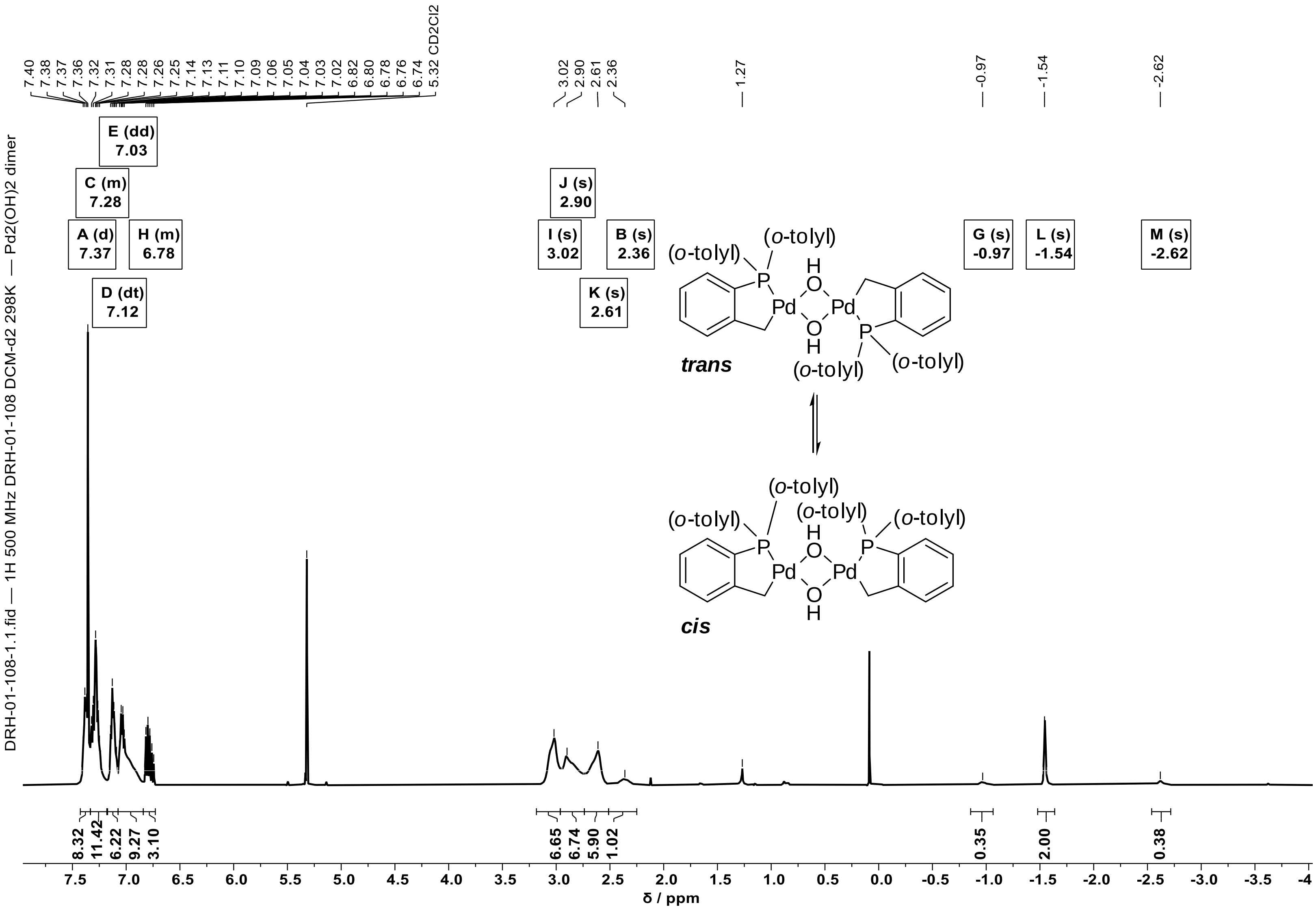

Figure 111: ^{1}H NMR (500 MHz, DCM-d_2, 32 scans, 298 K) spectrum of the [Pd(C^P)(μ_2-OH)]$_2$ palladacycle 2. Lab book ref. DRH-01-108

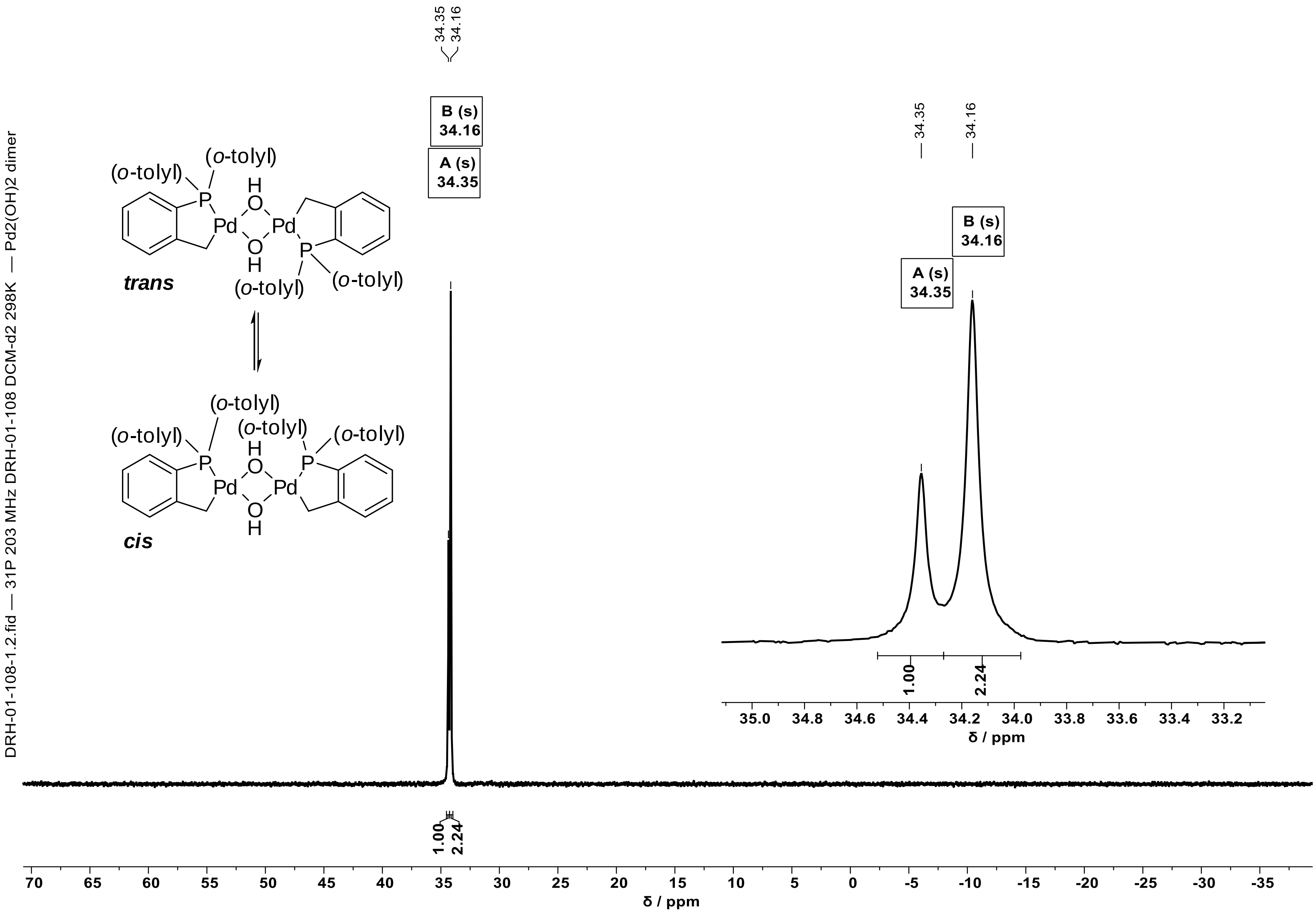

Figure 112: ^{31}P **NMR (203 MHz, DCM-*d*$_2$, 128 scans, 298 K) spectrum of the [Pd(C^P)(μ_2-OH)]$_2$ palladacycle 2. Cis/trans isomers are present, giving two signals. Lab book ref. DRH-01-108**

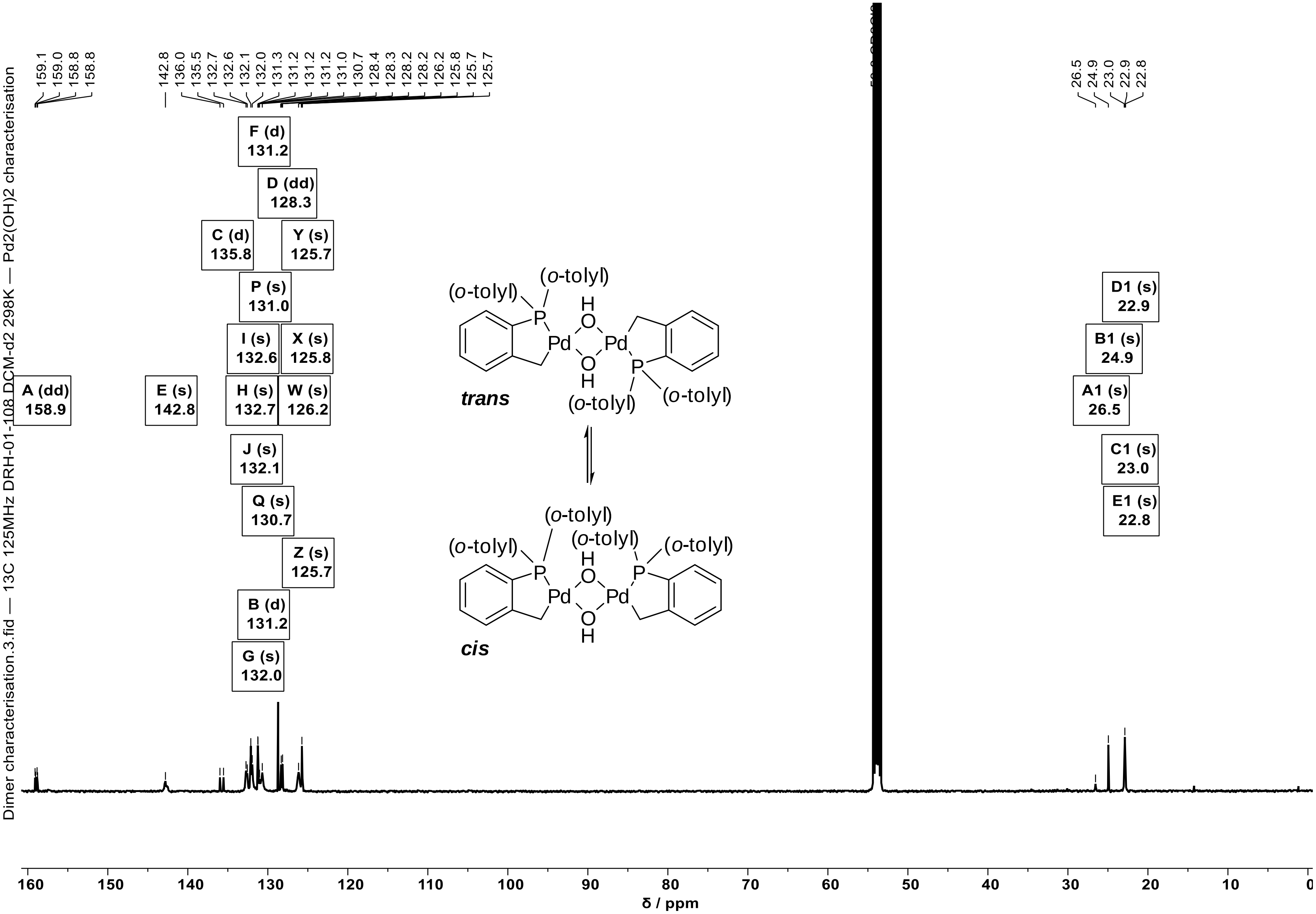

Figure 113: ^{13}C NMR (126 MHz, DCM-d_2, 49732 scans, 298 K) spectrum of the [Pd(C^P)(μ_2-OH)]$_2$ palladacycle 2. Lab book ref. DRH-01-108

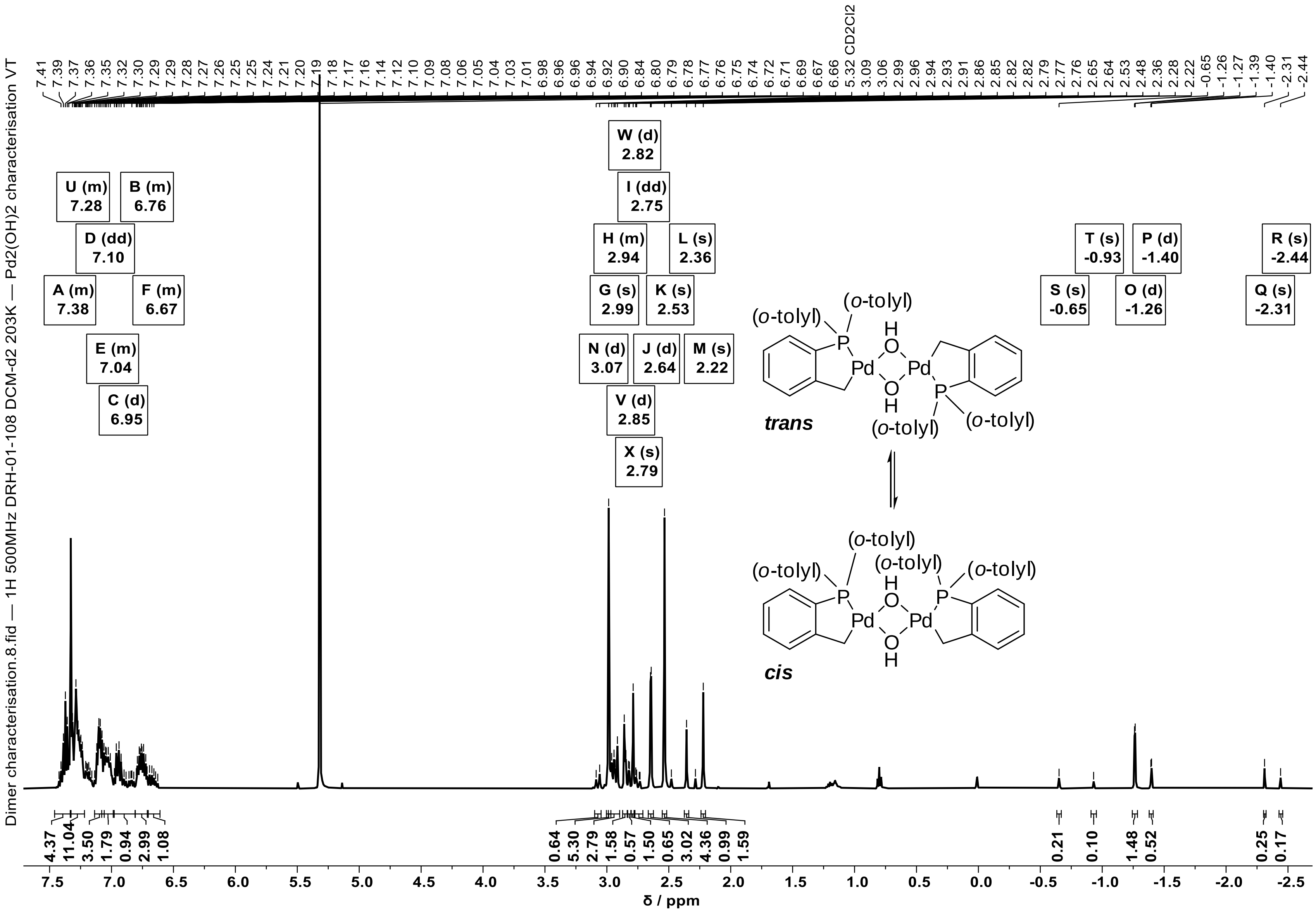

Figure 114: ^{1}H NMR (500 MHz, DCM-d_2, 64 scans, 203 K) spectrum of the $[Pd(C^P)(\mu_2\text{-}OH)]_2$ palladacycle 2. Lab book ref. DRH-01-108

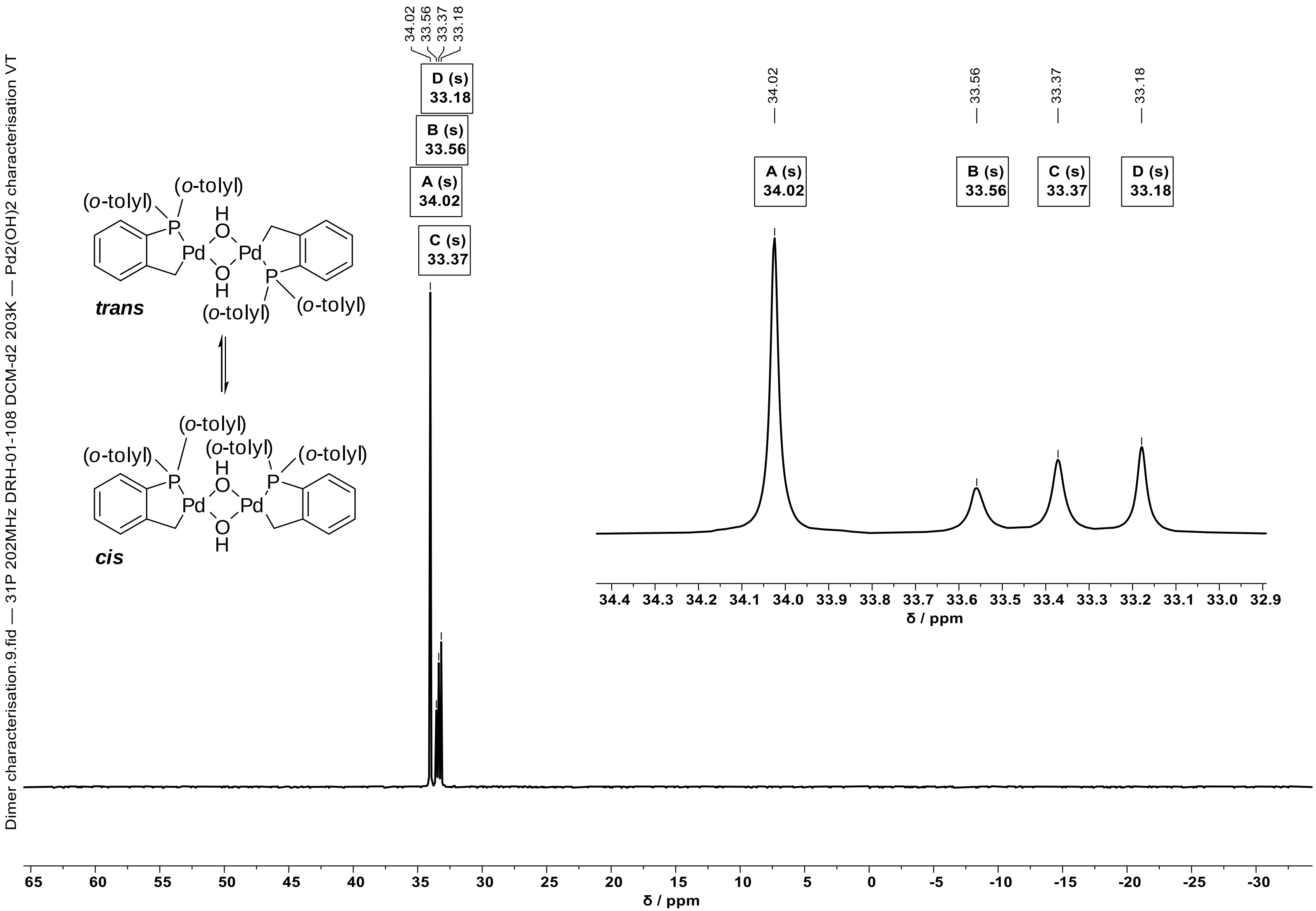

Figure 115: ^{31}P NMR (203 MHz, DCM-*d*$_2$, 256 scans, 203 K) spectrum of the [Pd(C^P)(μ_2-OH)]$_2$ palladacycle 2. Lab book ref. DRH-01-108

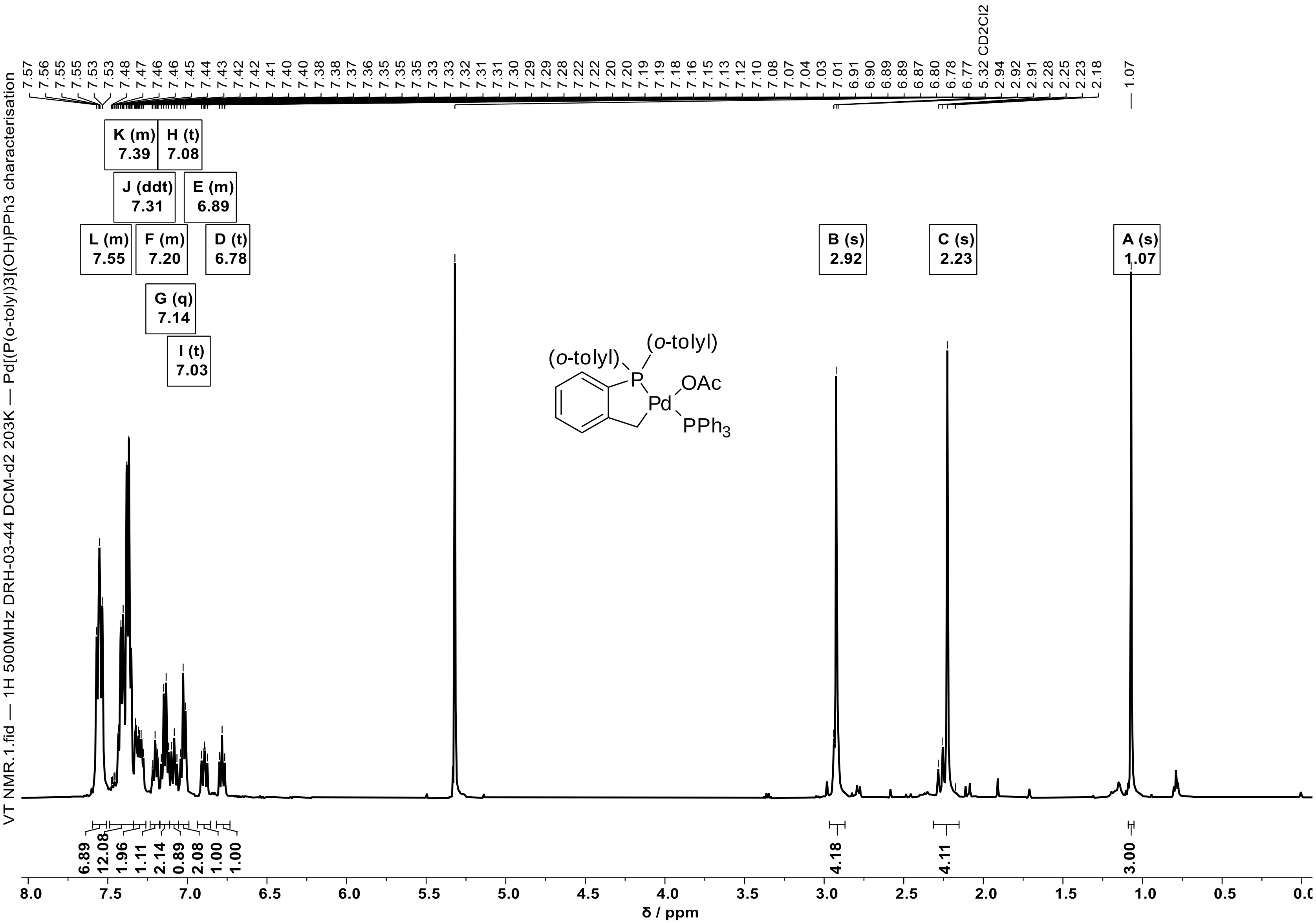

Figure 116: ¹H NMR (500 MHz, DCM-d_2, 32 scans, 203 K) spectrum of the Pd(C^P)(OAc)(PPh_3)] palladacyclic monomer SI43. Lab book ref. DRH-03-44

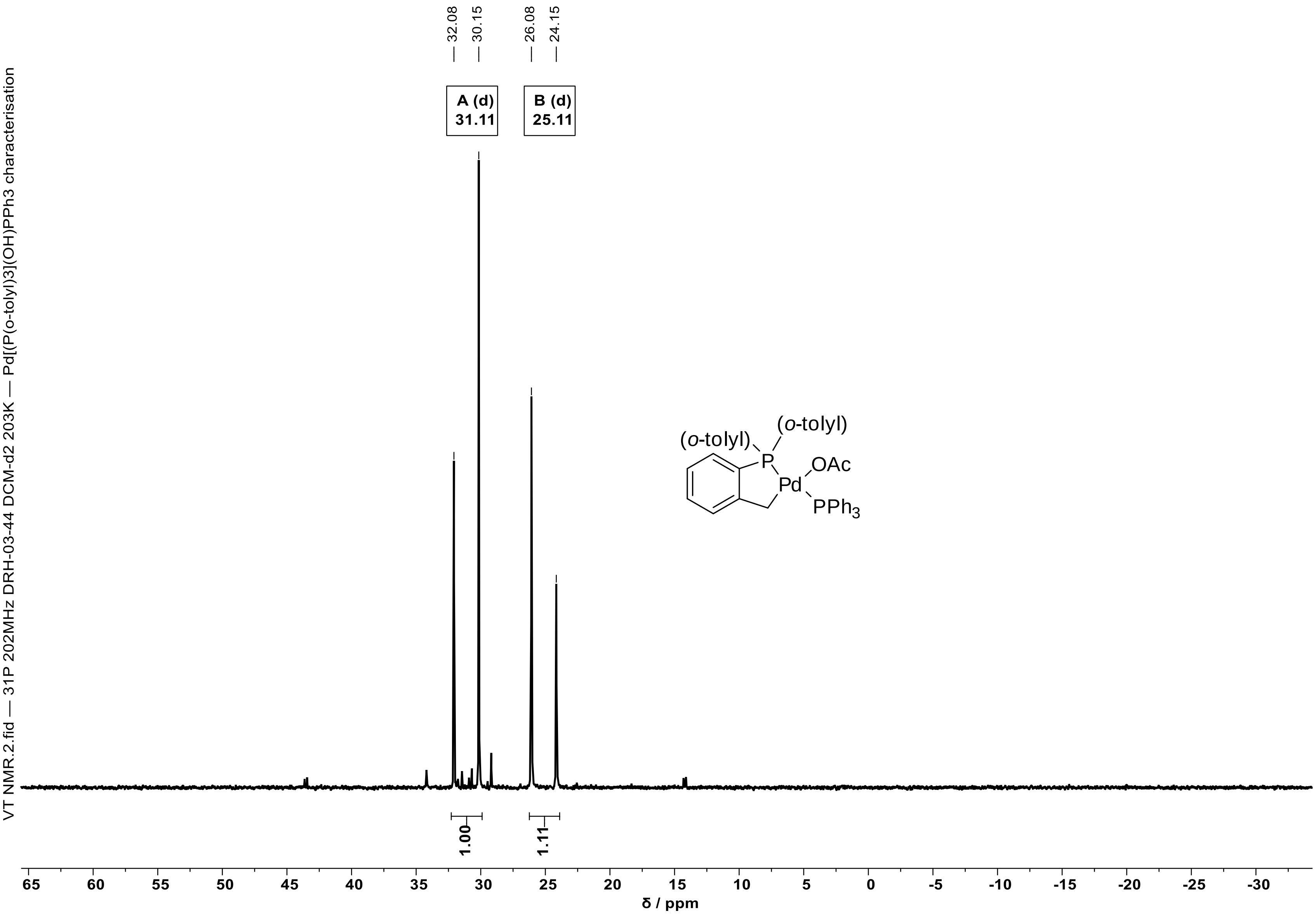

Figure 117: ^{31}P NMR (203 MHz, DCM-*d*$_2$, 128 scans, 203 K) spectrum of the Pd(C^P)(OAc)(PPh_3)] palladacyclic monomer SI43. Lab book ref. DRH-03-44

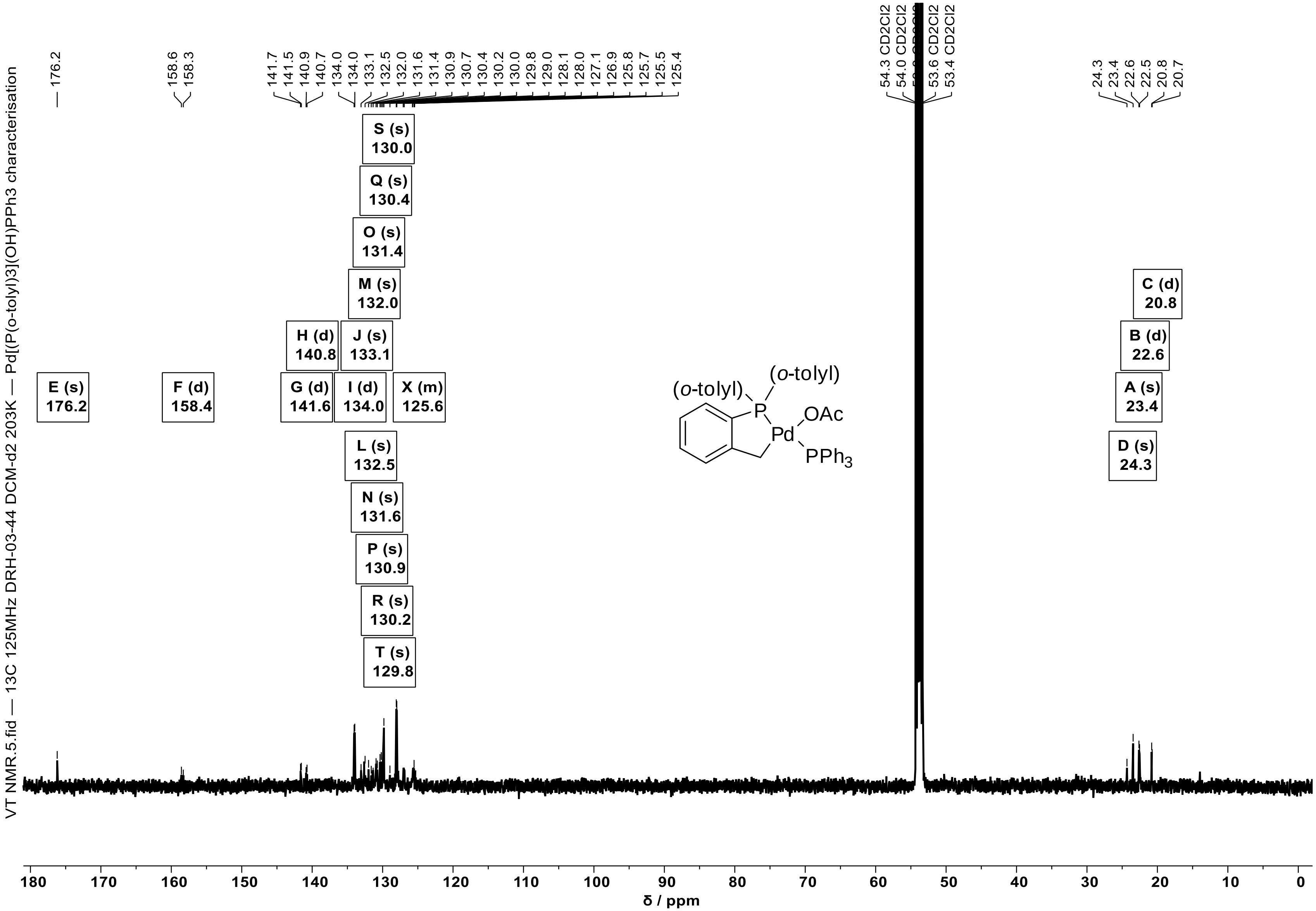

Figure 118: ^{13}**C NMR (126 MHz, DCM-*d*$_2$, 1256 scans, 203 K) spectrum of the Pd(C^P)(OAc)(PPh$_3$)] palladacyclic monomer SI43. Lab book ref. DRH-03-44**

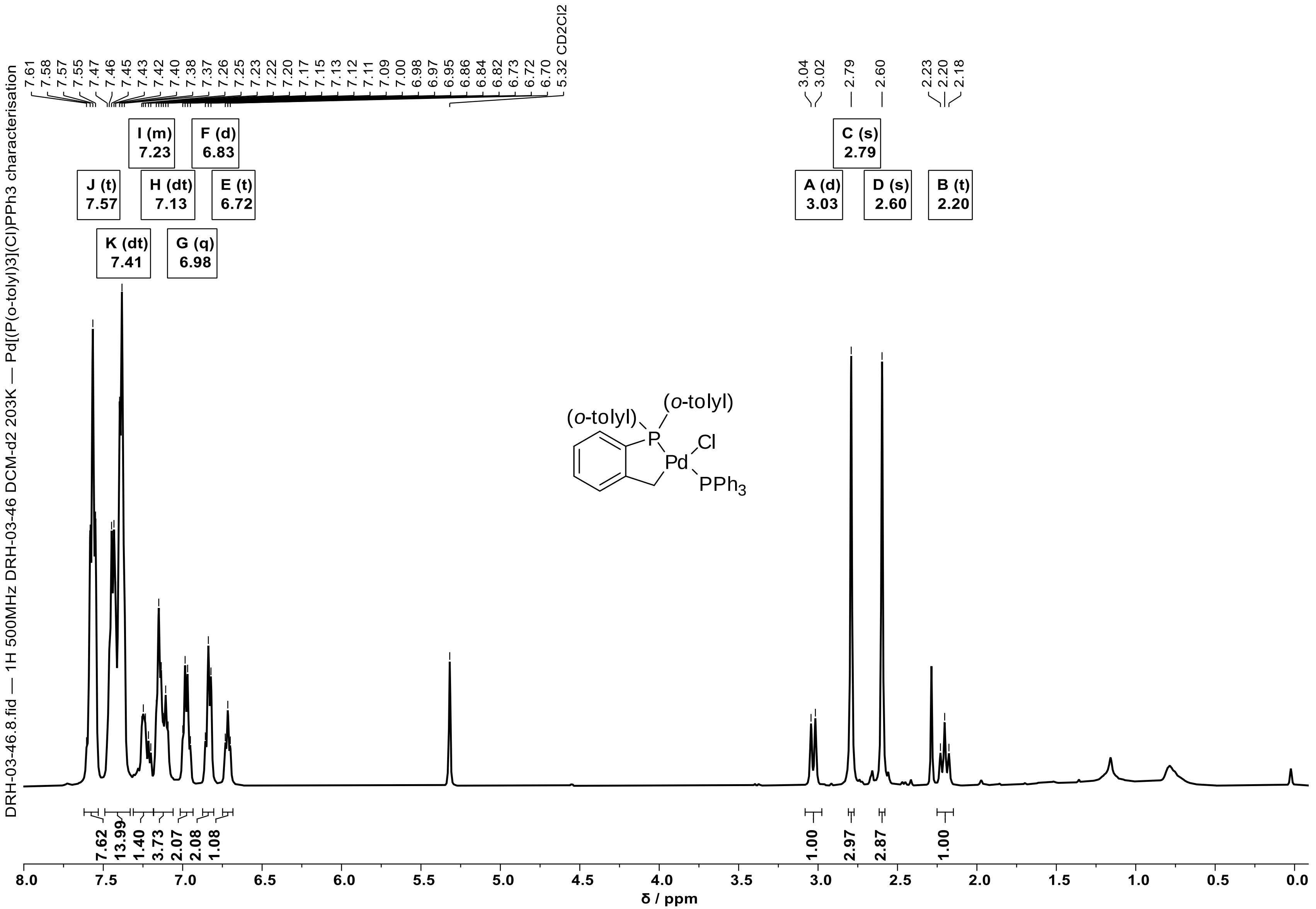

Figure 119: ^{1}H NMR (500 MHz, DCM-d_2, 32 scans, 203 K) spectrum of the Pd(C^P)(Cl)(PPh_3)] palladacyclic monomer SI44. Lab book ref. DRH-03-46

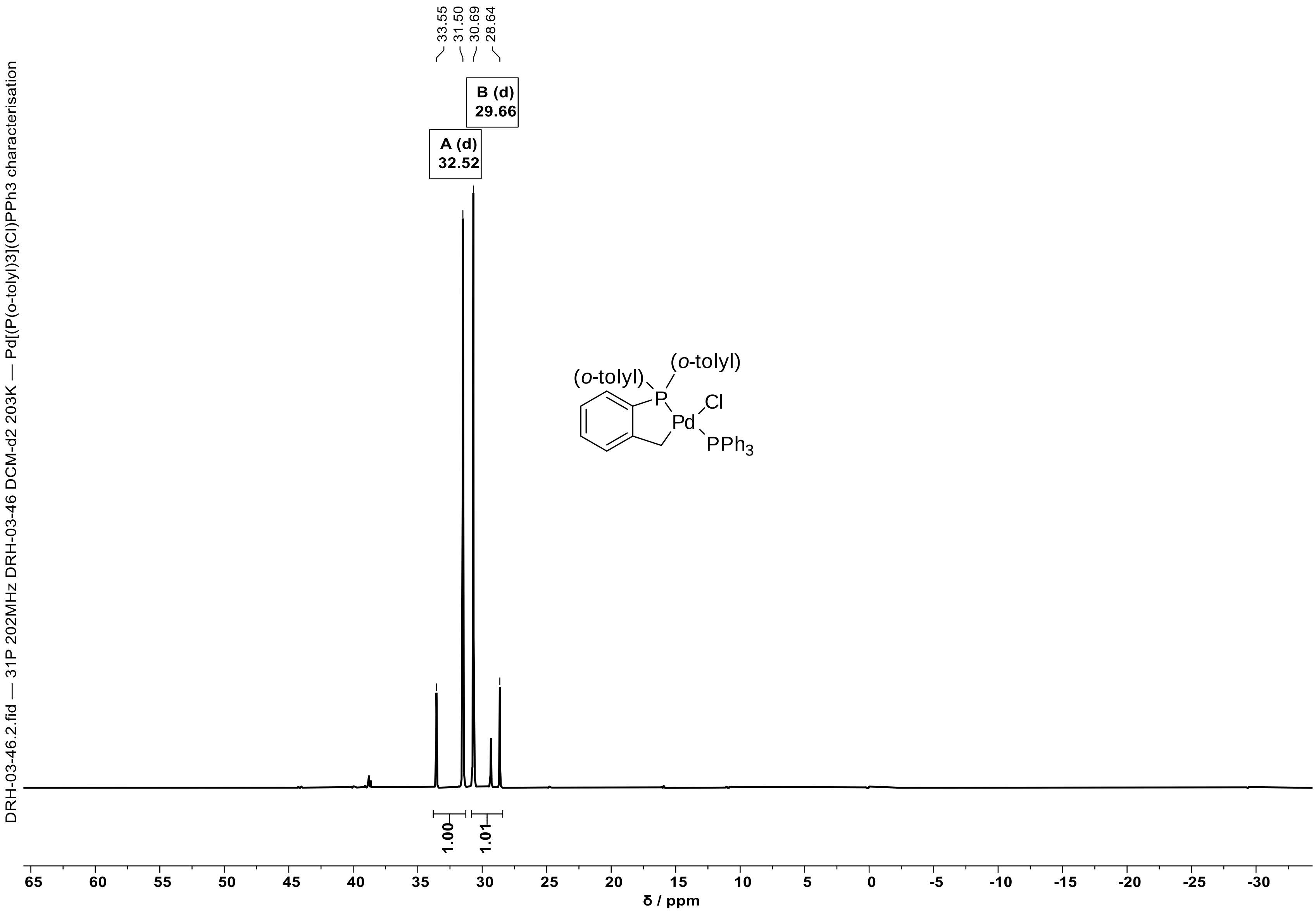

Figure 120: ^{31}P NMR (203 MHz, DCM-*d*$_2$, 64 scans, 203 K) spectrum of the Pd(C^P)(Cl)(PPh$_3$)] palladacyclic monomer SI44. Lab book ref. DRH-03-46

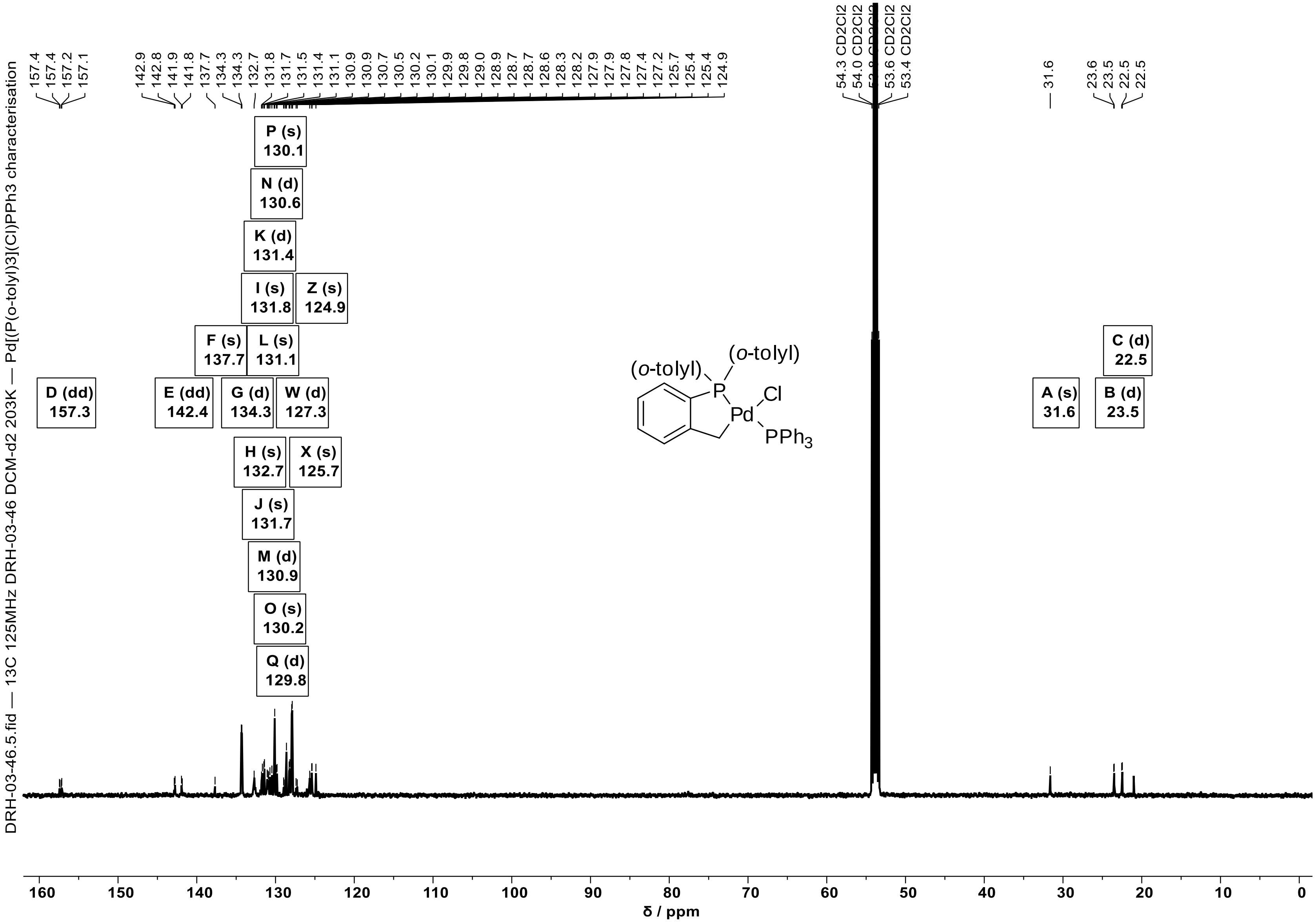

Figure 121: ^{13}C NMR (126 MHz, DCM-d_2, 512 scans, 203 K) spectrum of the Pd(C^P)(Cl)(PPh$_3$)] palladacyclic monomer SI44. Lab book ref. DRH-03-46

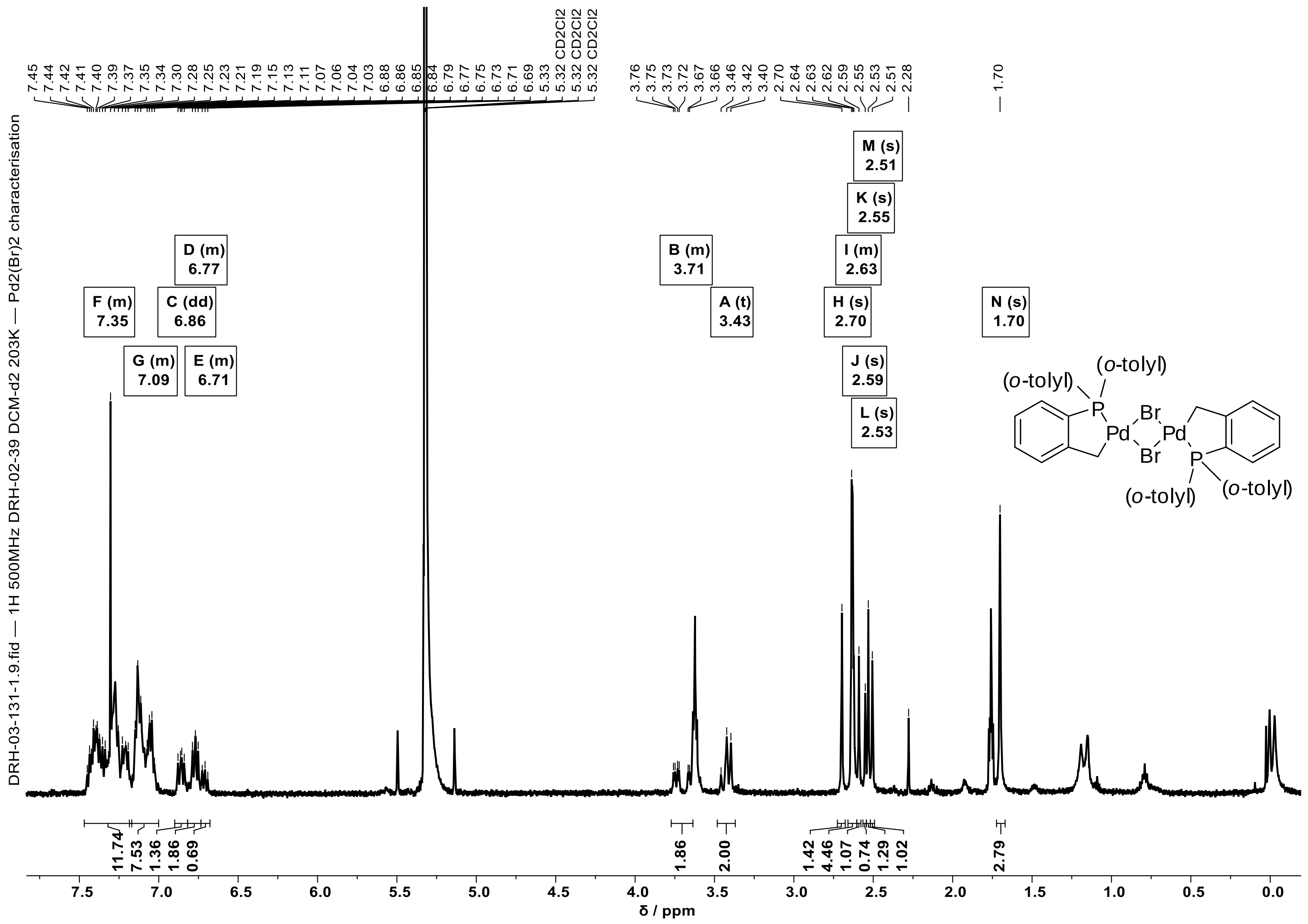

Figure 122: ^{1}H NMR (500 MHz, DCM-d_2, 128 scans, 203 K) spectrum of the [Pd(C^P)(μ_2-Br)]$_2$ palladacycle 35. Residual solvent signals (THF, grease) below 1.5 ppm have not been integrated. Lab book ref. DRH-02-39

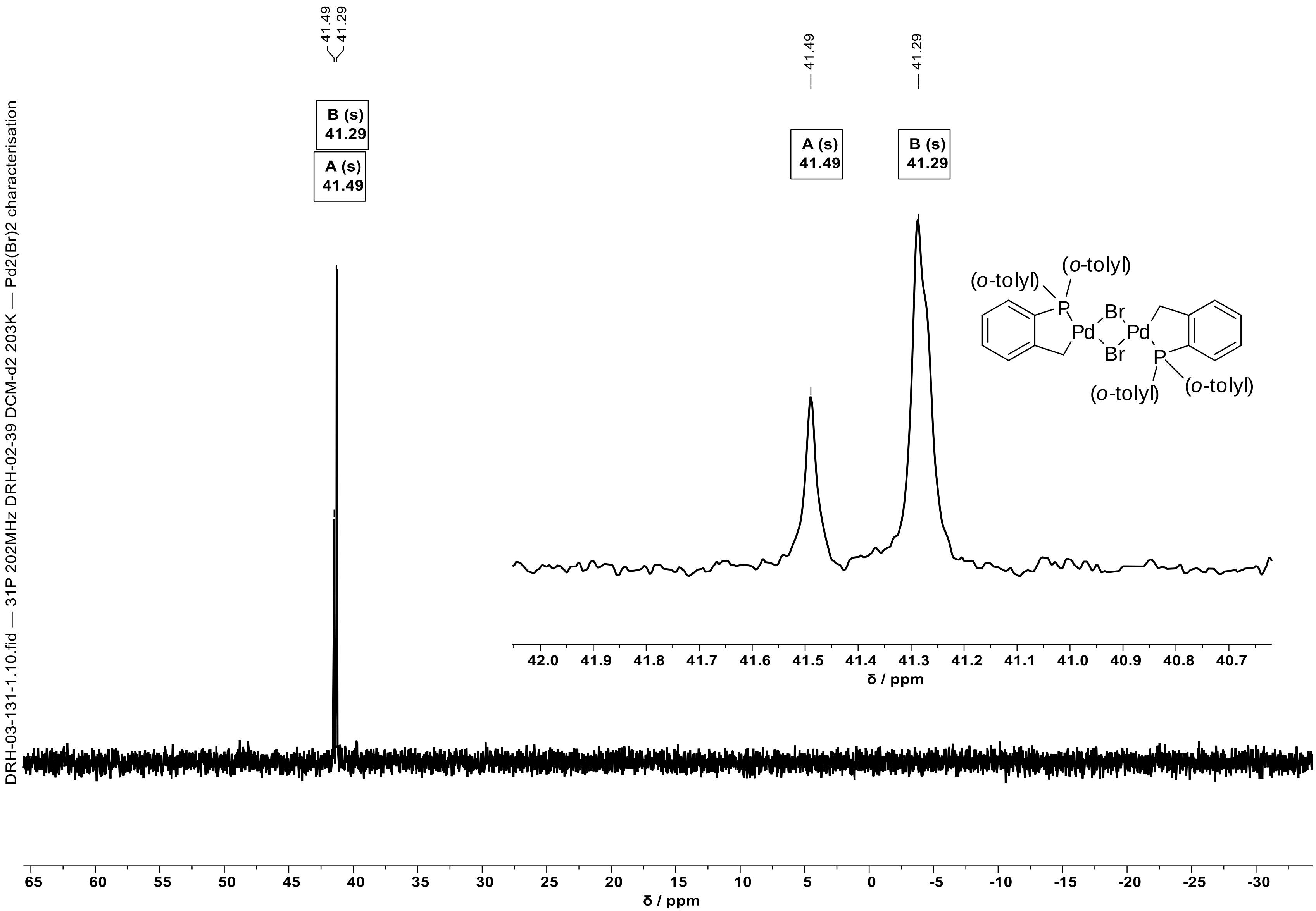

Figure 123: ^{31}P NMR (202 MHz, DCM-d_2, 256 scans, 203 K) spectrum of the [Pd(C^P)(μ_2-Br)]$_2$ palladacycle 35. Lab book ref. DRH-02-39

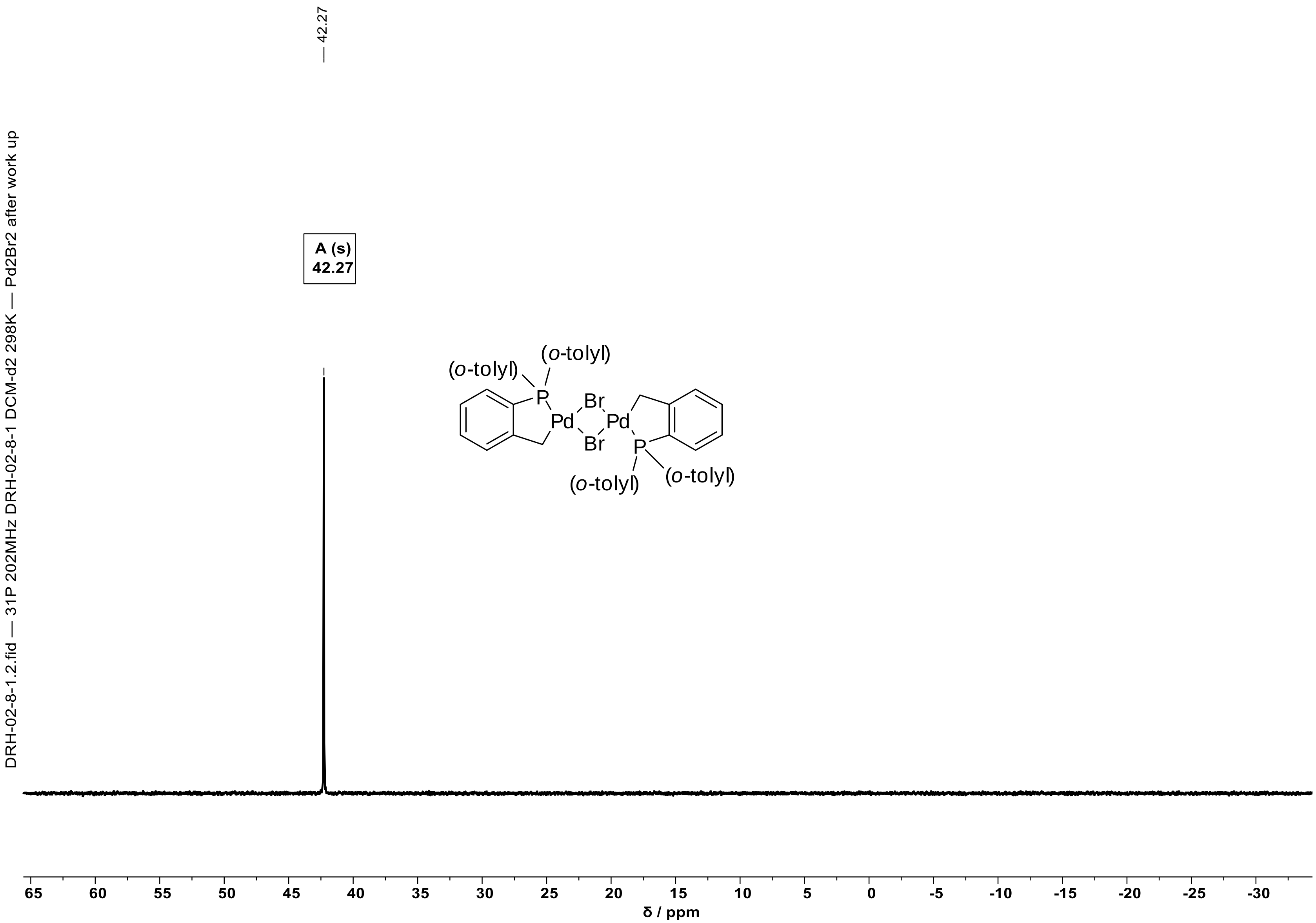

Figure 124: ^{31}P NMR (202 MHz, DCM-d_2, 128 scans, 203 K) spectrum of the [Pd(C^P)(μ_2-Br)]$_2$ palladacycle 35. Lab book ref. DRH-02-8

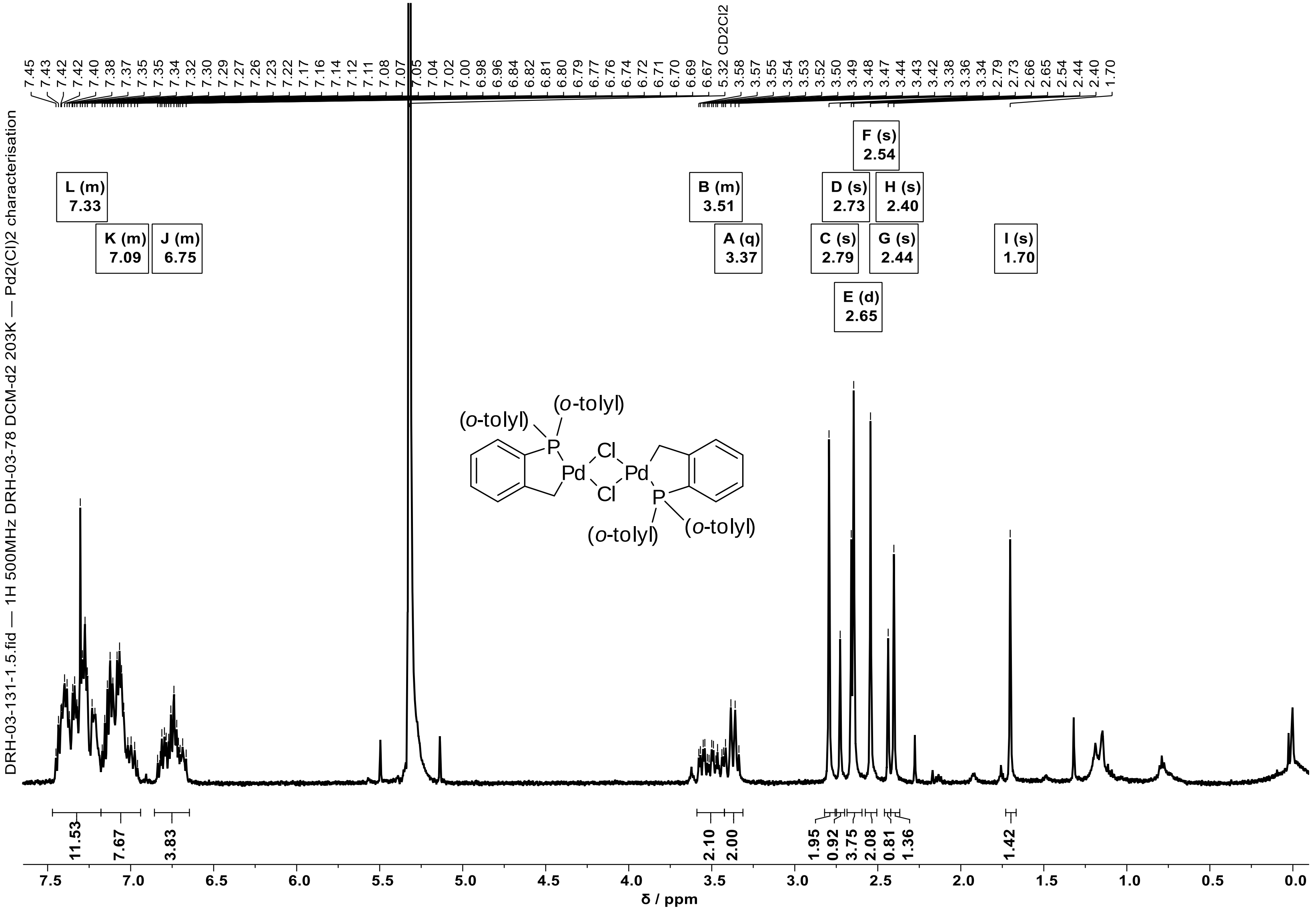

Figure 125: ^{1}H NMR (500 MHz, DCM-d_2, 128 scans, 203 K) spectrum of the $[Pd(C^\wedge P)(\mu_2\text{-}Cl)]_2$ palladacycle 17. Residual solvent signals (most likely water and grease) below 1.5 ppm have not been integrated. Lab book ref. DRH-03-78

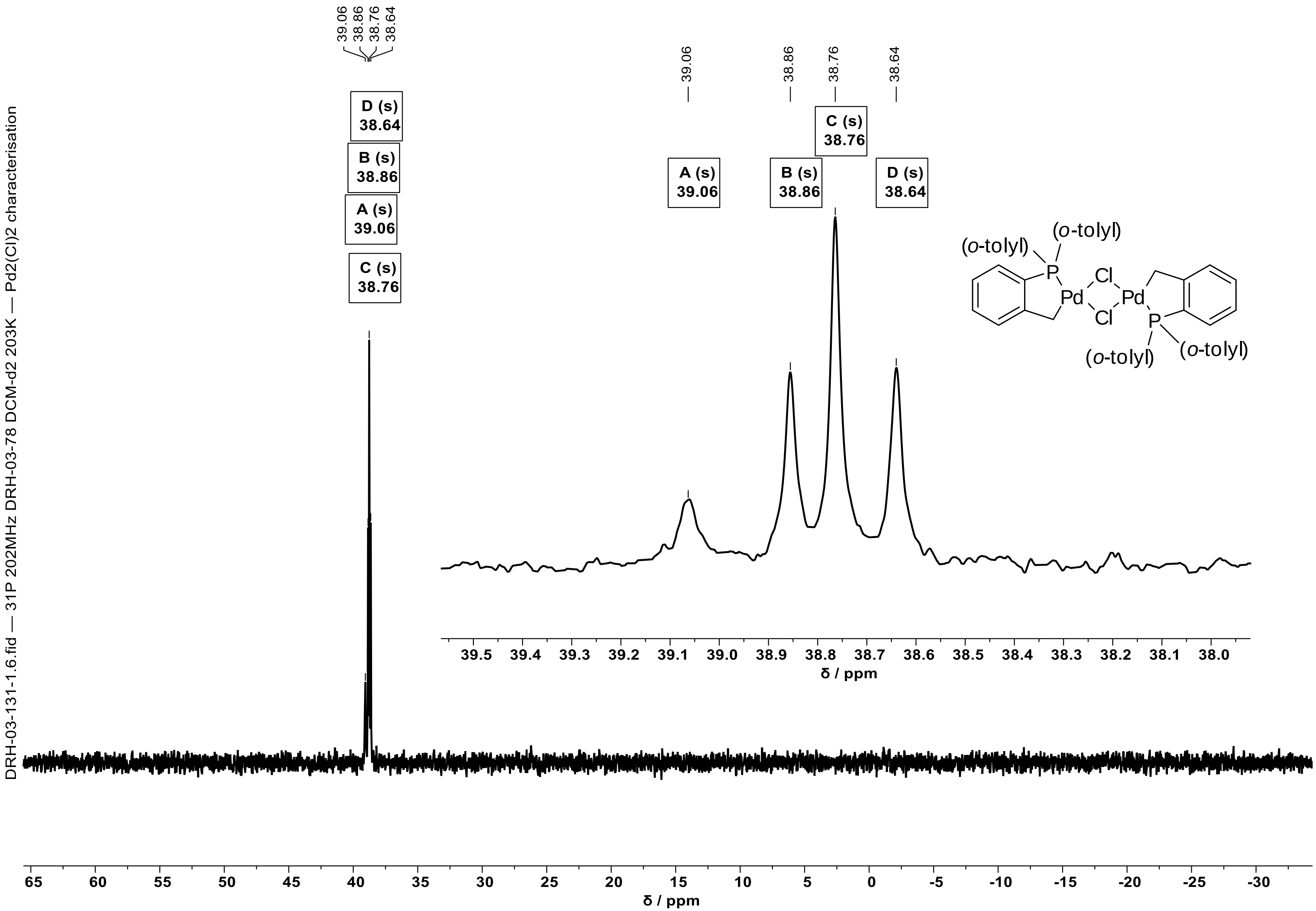

Figure 126: ^{31}P NMR (203 MHz, DCM-d_2, 128 scans, 203 K) spectrum of the [Pd(C^P)(μ_2-Cl)]$_2$ palladacycle 17. Lab book ref. DRH-03-78

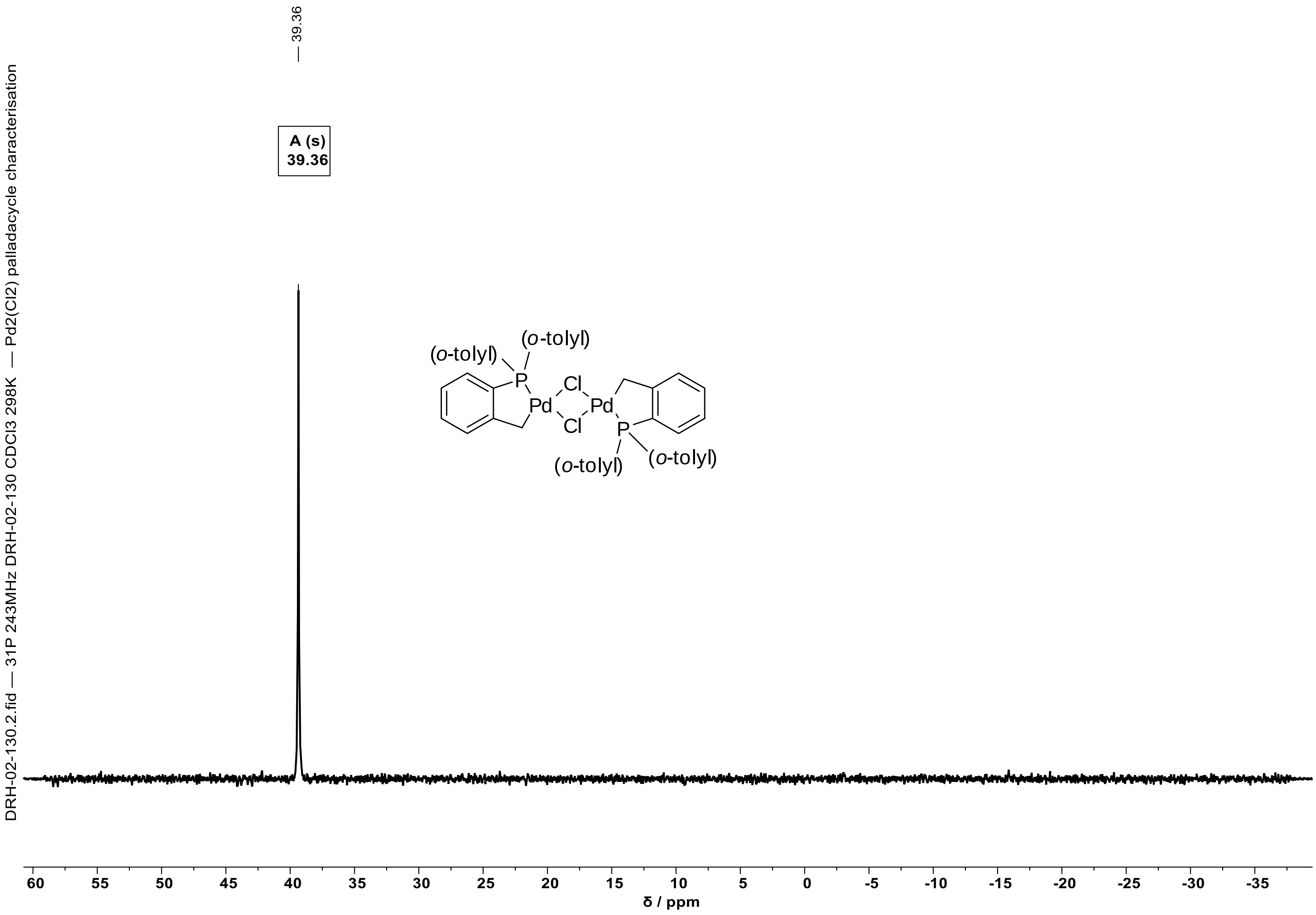

Figure 127: ^{31}P NMR (243 MHz, C*D*Cl_3, 128 scans, 298 K) spectrum of the [Pd(C^P)(μ_2-Cl)]$_2$ palladacycle 17. Lab book ref. DRH-02-130

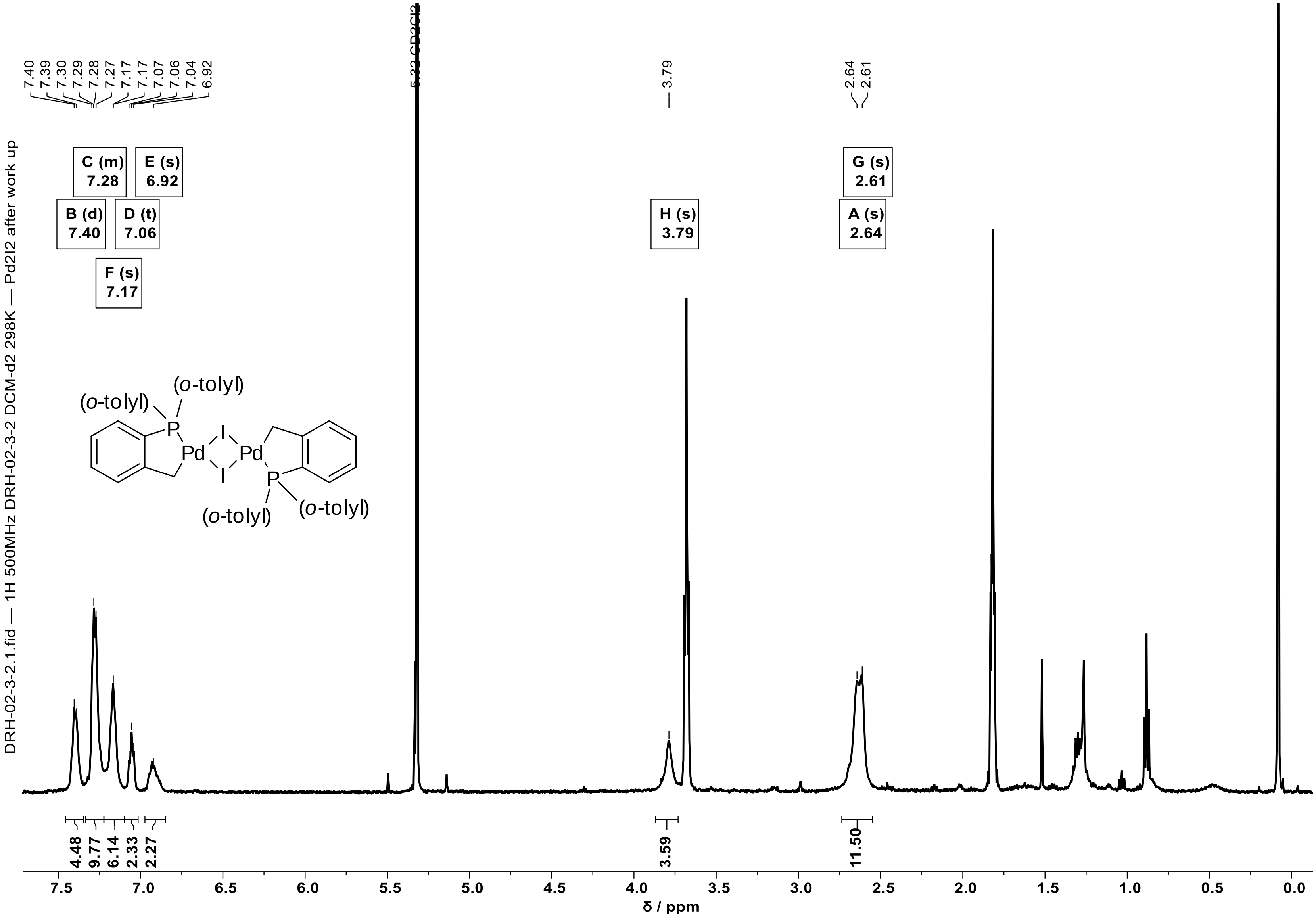

Figure 128: ^{1}H NMR (500 MHz, DCM-d_2, 64 scans, 298 K) spectrum of the [Pd(C^P)(μ_2-I)]$_2$ palladacycle 32. Residual solvent signals (THF and grease) have not been integrated. Lab book ref. DRH-02-3

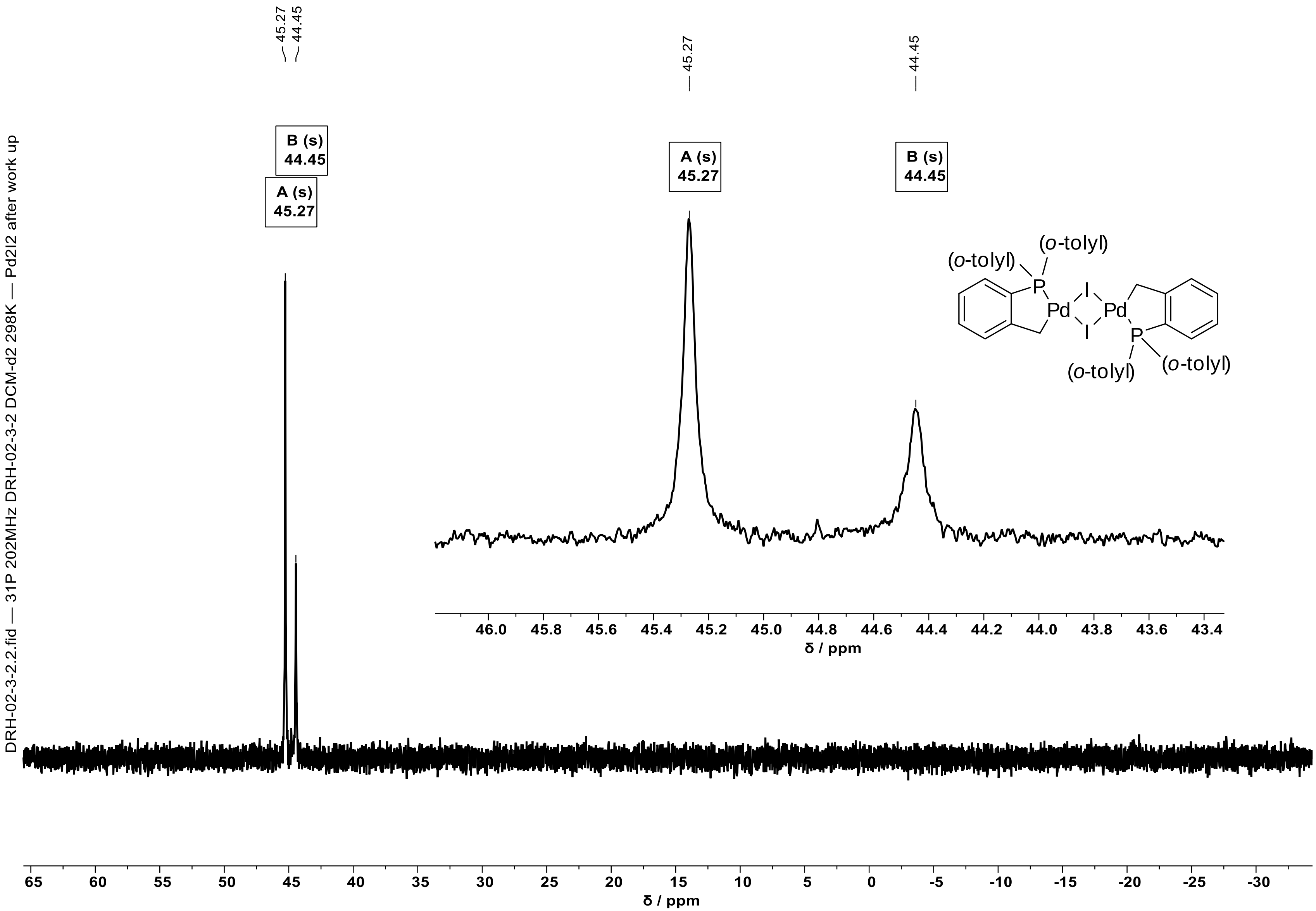

Figure 129: ^{31}P NMR (203 MHz, DCM-d_2, 256 scans, 298 K) spectrum of the [Pd(C^P)(μ_2-I)]$_2$ palladacycle 32. Lab book ref. DRH-02-3

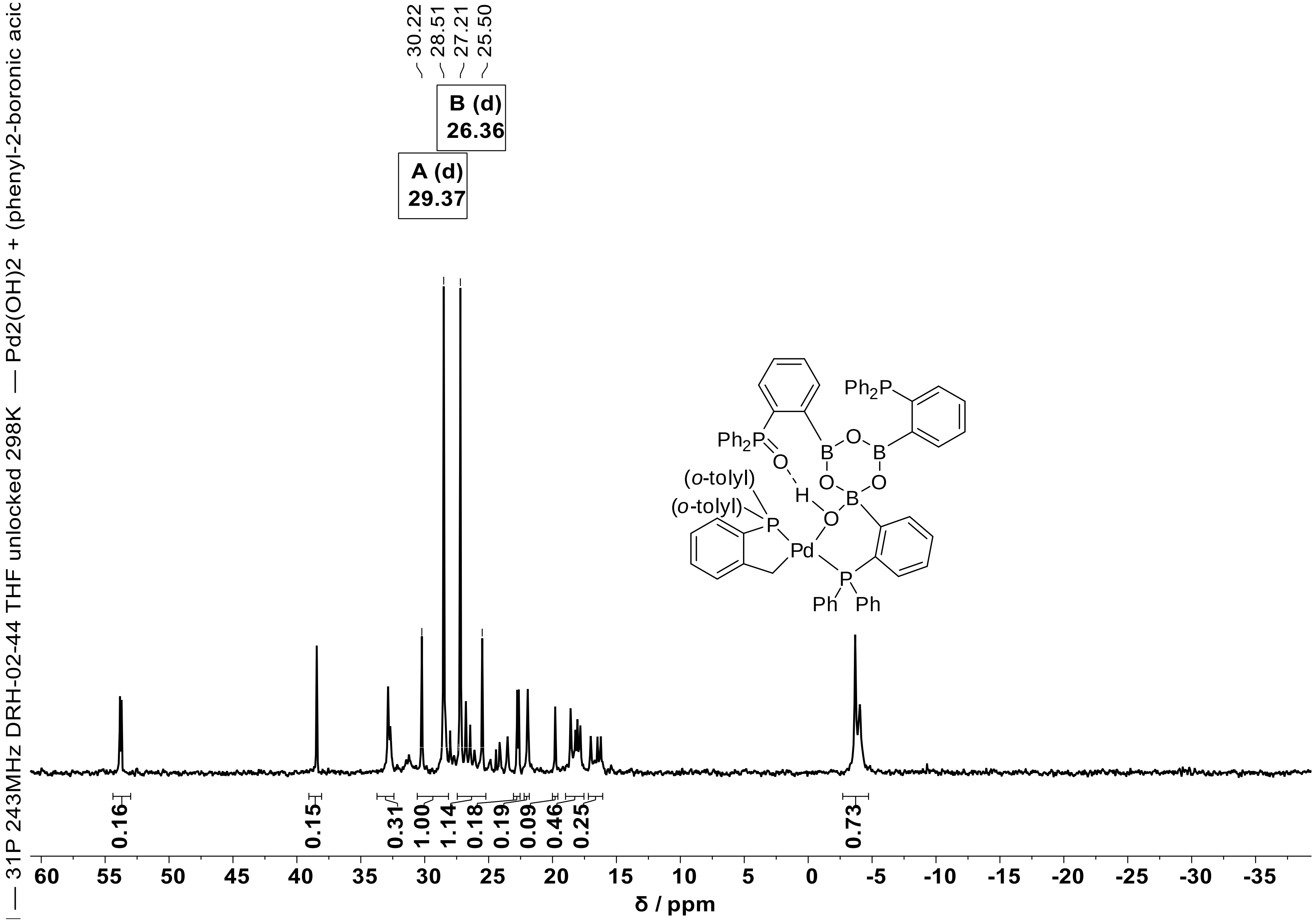

Figure 130: **^{31}P NMR (243 MHz, THF-unlocked, 128 scans, 298 K) spectrum of the Pd-OH boronic acid phosphine complex 21. Lab book ref. DRH-02-44**

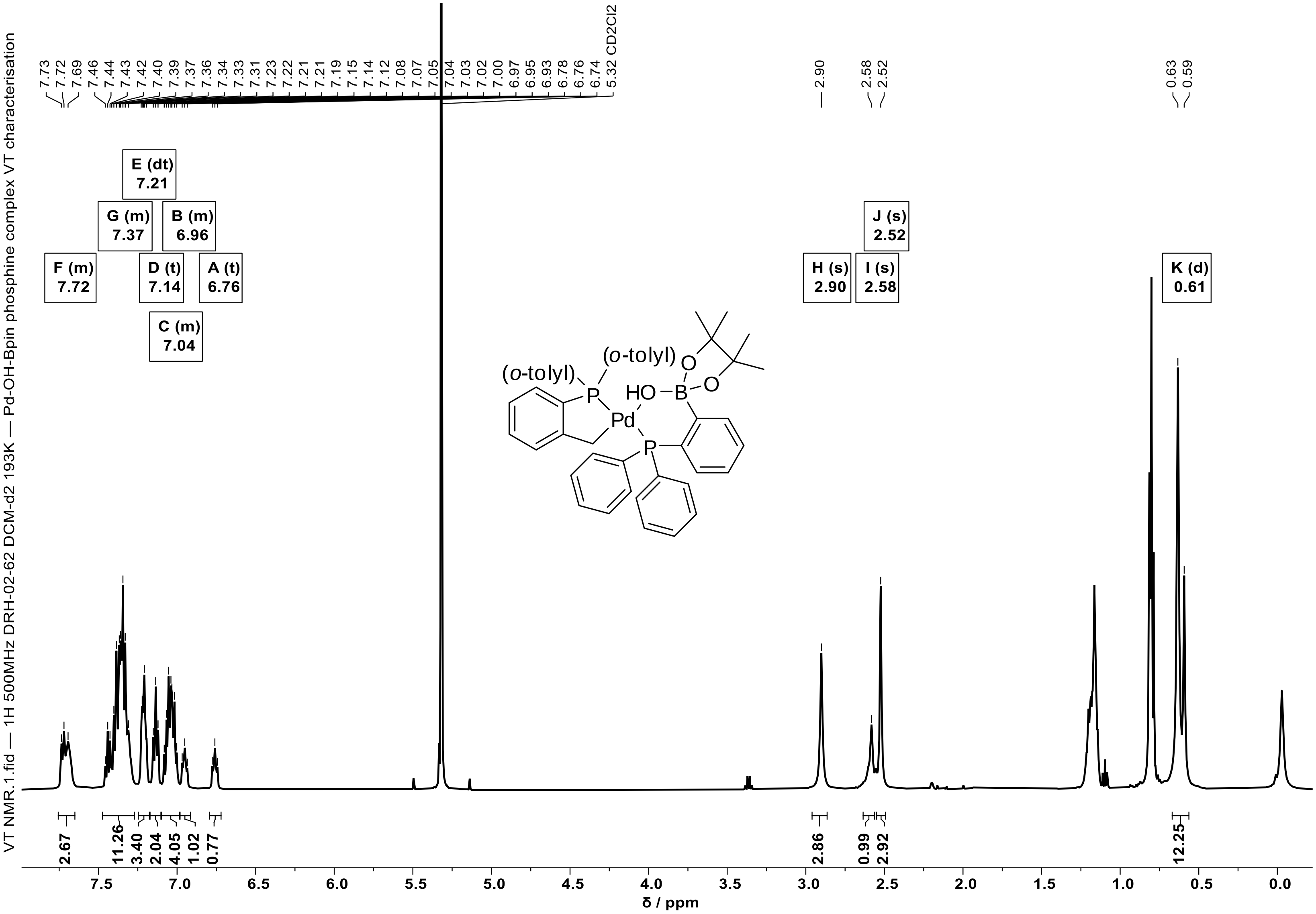

Figure 131: ^{1}H NMR (500 MHz, DCM-d_2, 32 scans, 193 K) spectrum of the phosphine stabilized Pd-OH-Bpin complex 23. Excess pinacol boronic acid was present and was not integrated. Lab book ref. DRH-02-62

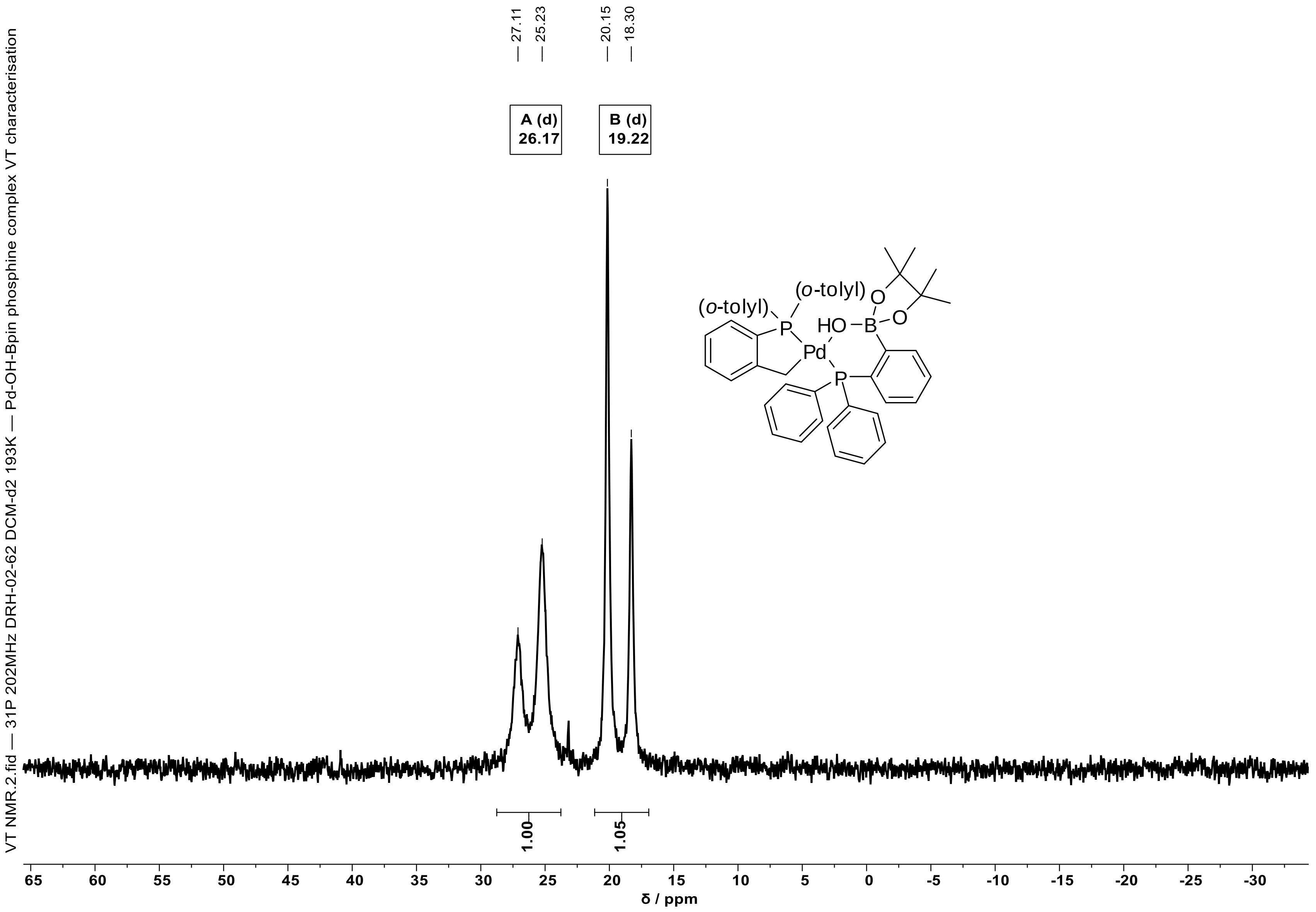

Figure 132: ^{31}P NMR (203 MHz, DCM-d_2, 128 scans, 193 K) spectrum of the phosphine stabilized Pd-OH-Bpin complex 23. Lab book ref. DRH-02-62

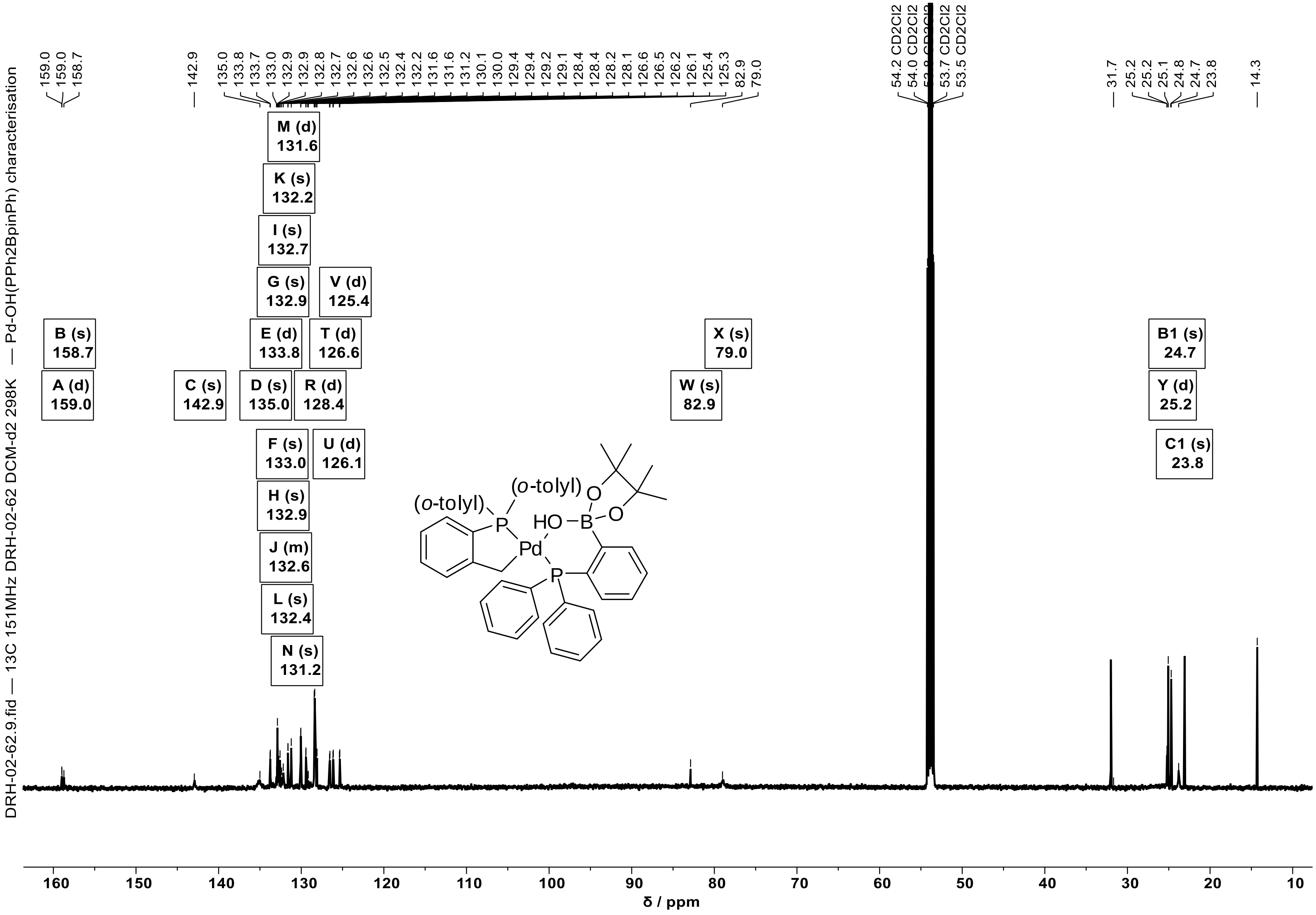

Figure 133: ^{13}**C NMR (151 MHz, DCM-*d*$_2$, 2048 scans, 298 K) spectrum of the phosphine stabilized Pd-OH-Bpin complex 23. Lab book ref. DRH-02-62**

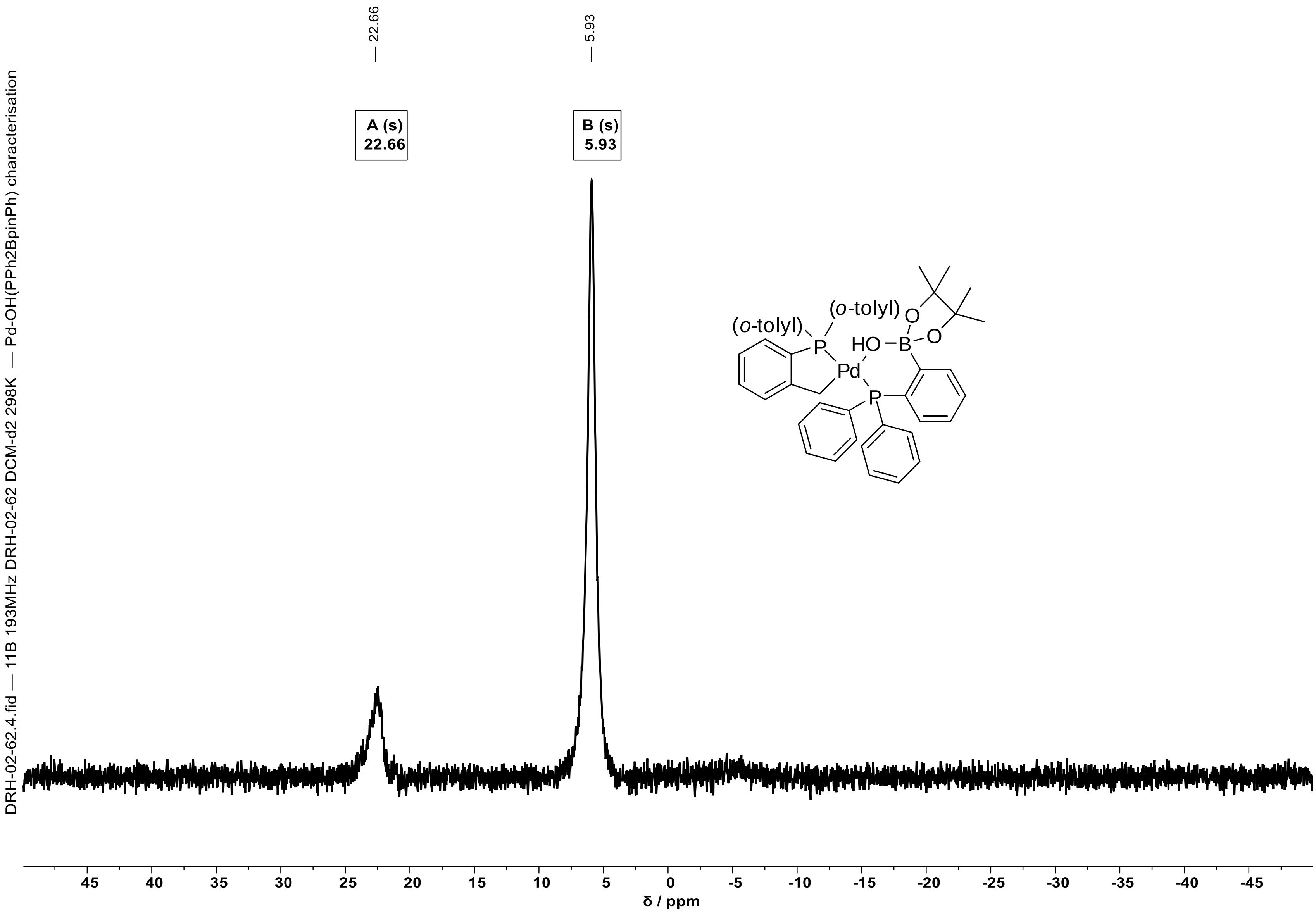

Figure 134: ^{11}B NMR (193 MHz, DCM-d_2, 32 scans, 298 K) spectrum of the phosphine stabilized Pd-OH-Bpin complex 23. Lab book ref. DRH-02-62

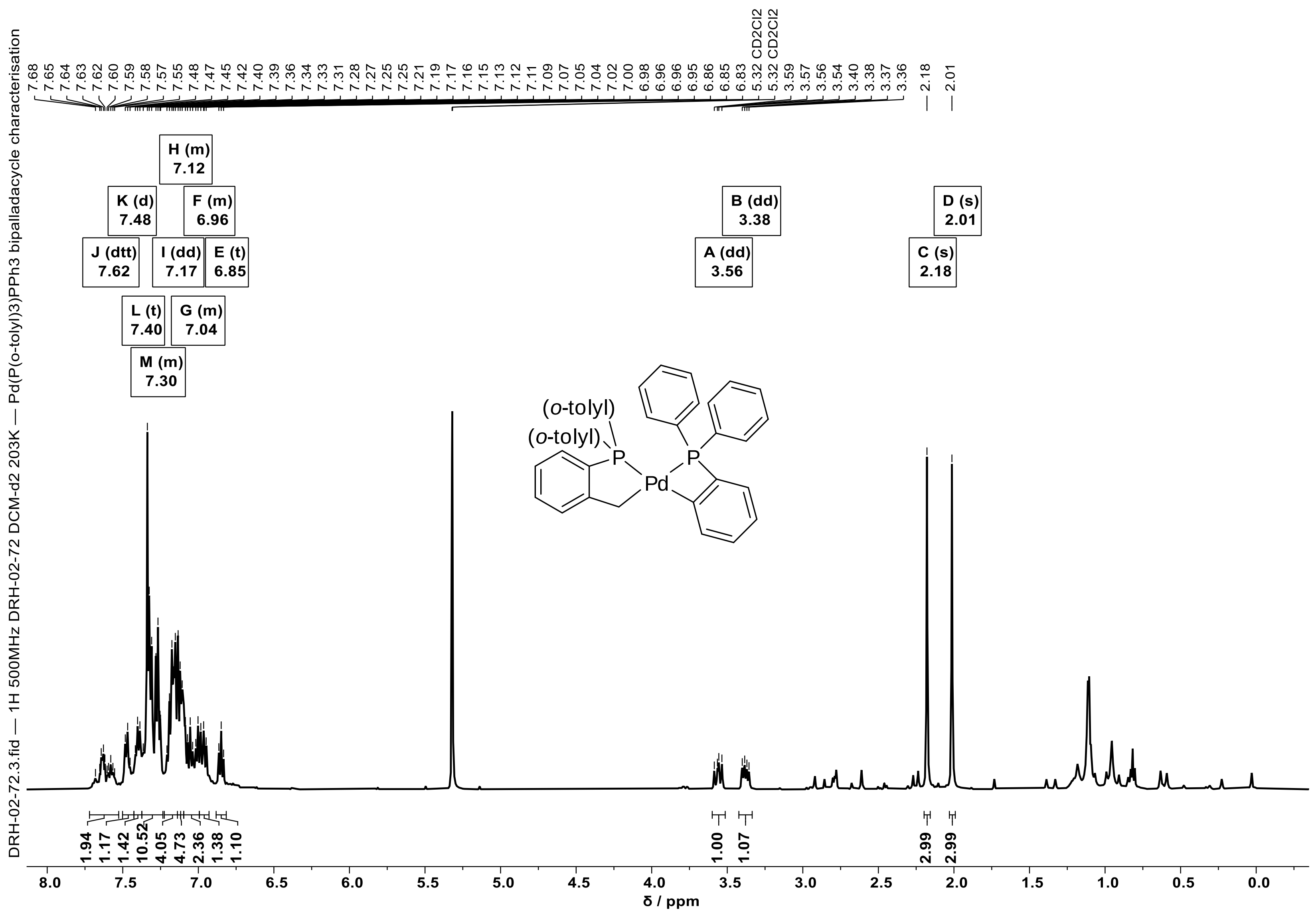

Figure 135: **^{1}H NMR (500 MHz, DCM-d_2, 32 scans, 203 K) spectrum of the bipalladacyclic Pd complex 25. Small amounts of residual pinacolboronic acid and residual solvent were present (below 1.5 ppm) and not integrated. Lab book ref. DRH-02-72**

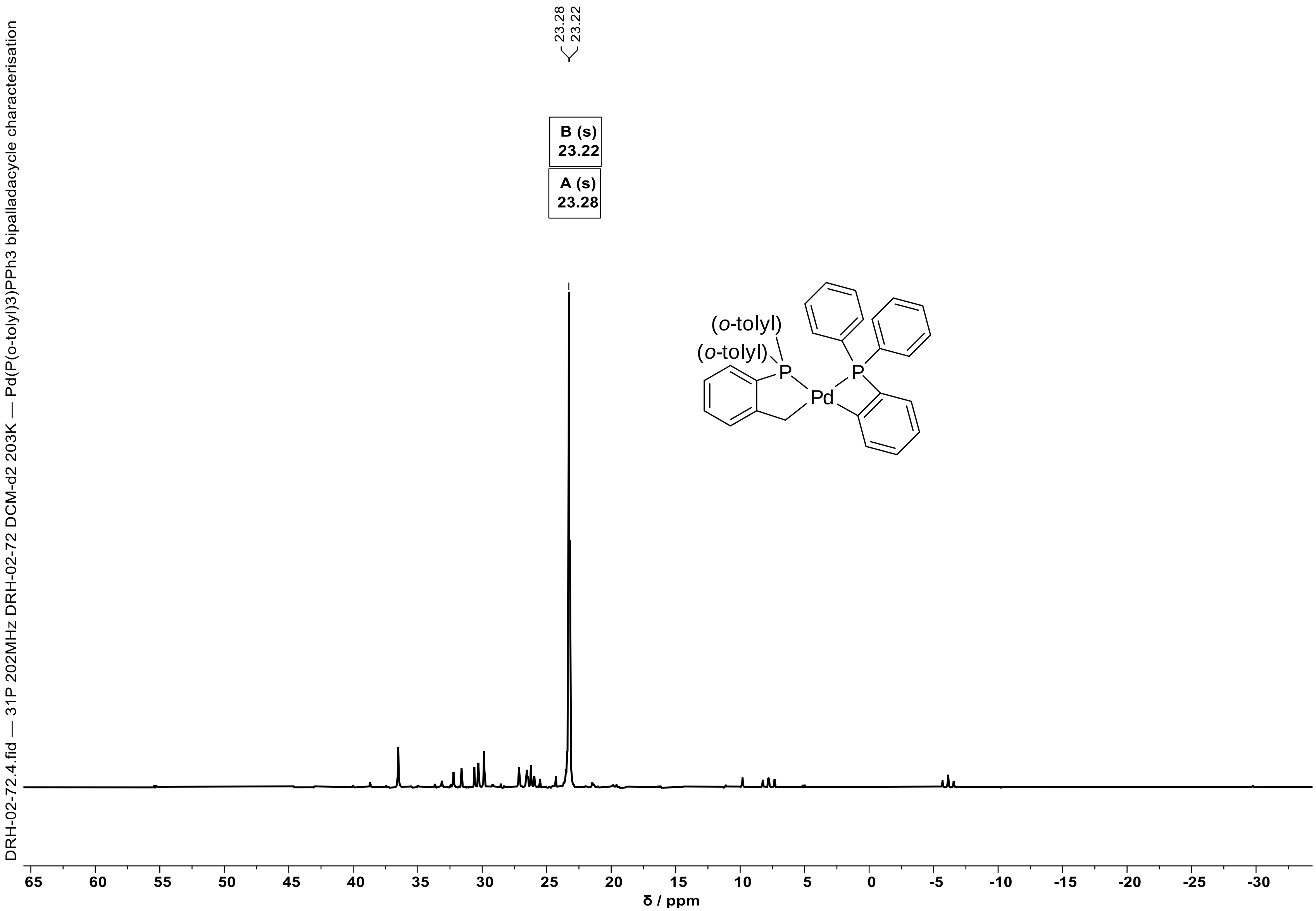

Figure 136: ^{31}P NMR (203 MHz, DCM-d_2, 128 scans, 203 K) spectrum of the bipalladacyclic Pd complex 25. Lab book ref. DRH-02-72

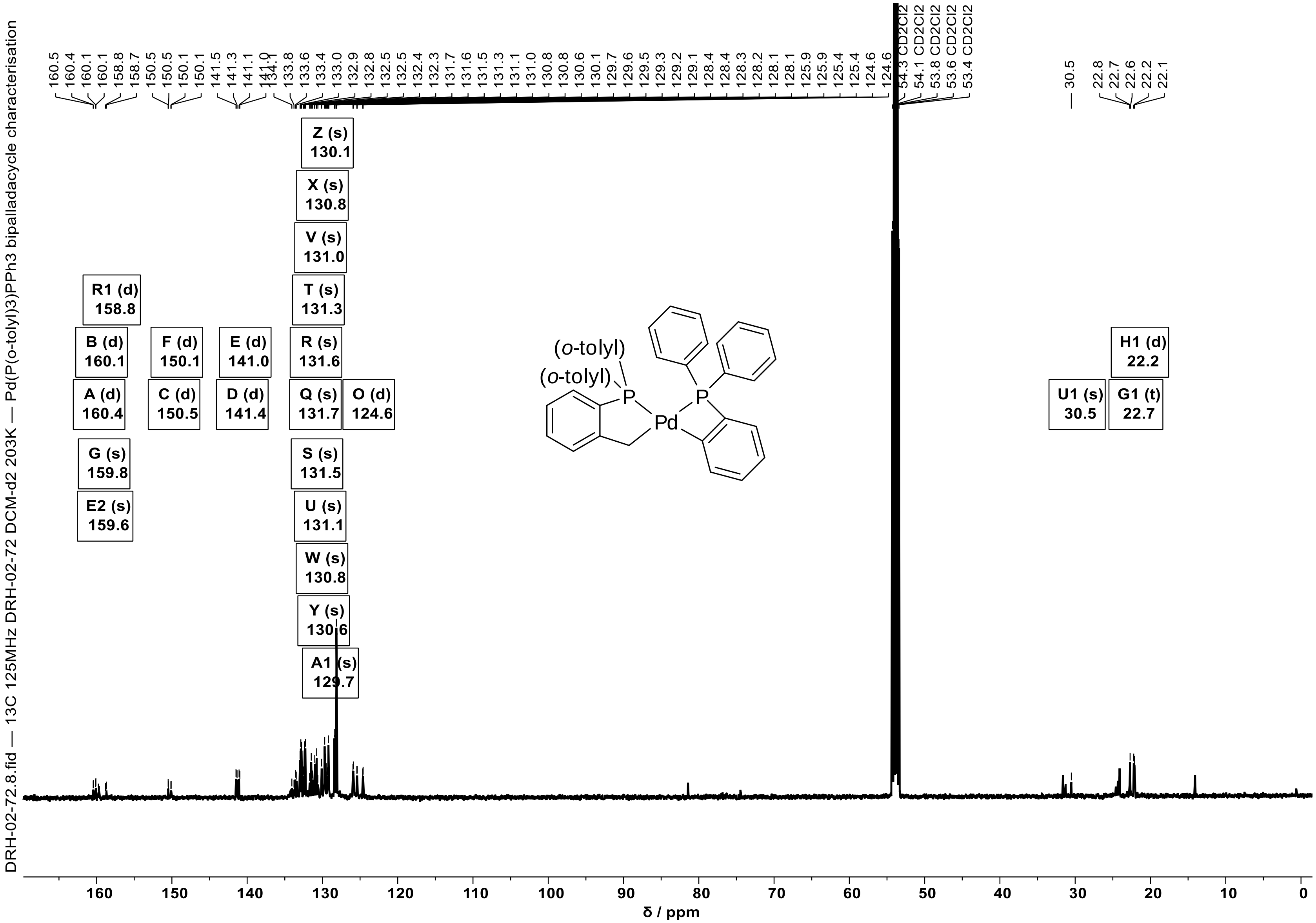

Figure 137: ^{31}C NMR (126 MHz, DCM-d_2, 1024 scans, 203 K) spectrum of the bipalladacyclic Pd complex 25. Lab book ref. DRH-02-72

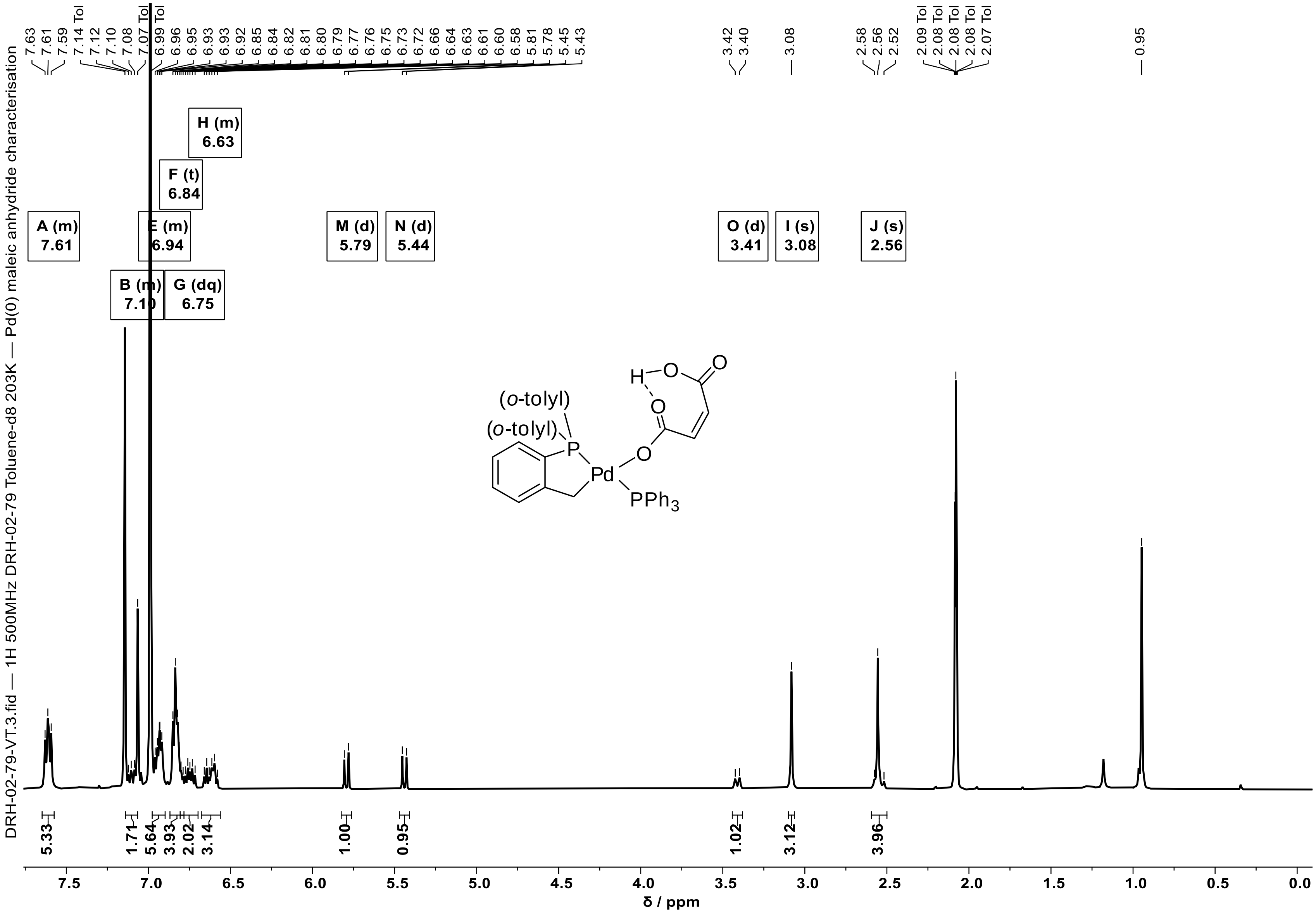

Figure 138: ^{1}H NMR (500 MHz, Toluene-d_8, 32 scans, 203 K) spectrum of the maleic acid Pd complex 27. Lab book ref. DRH-02-79

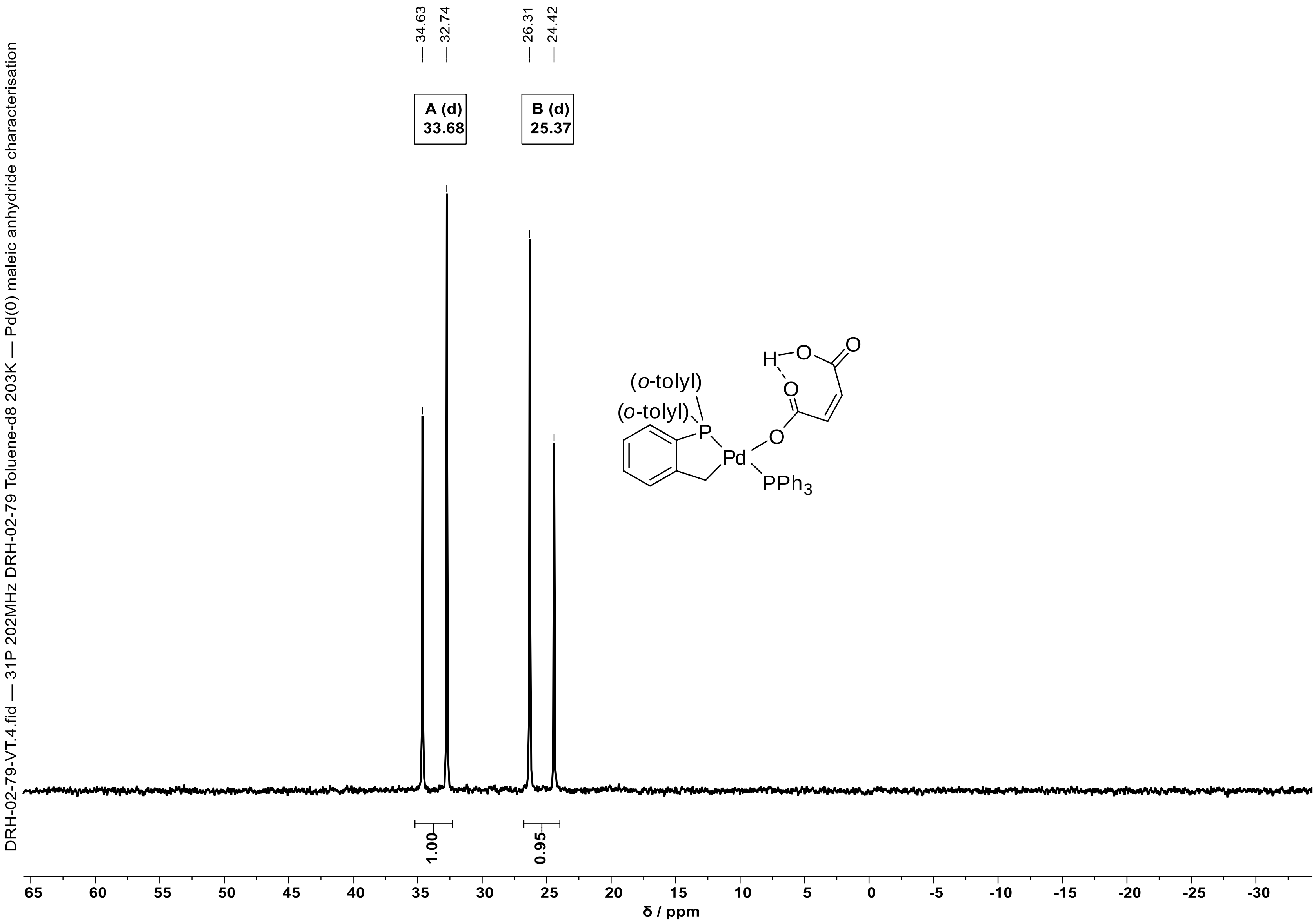

Figure 139: ^{31}P NMR (203 MHz, Toluene-d_8, 128 scans, 203 K) spectrum of the maleic acid Pd complex 27. Lab book ref. DRH-02-79

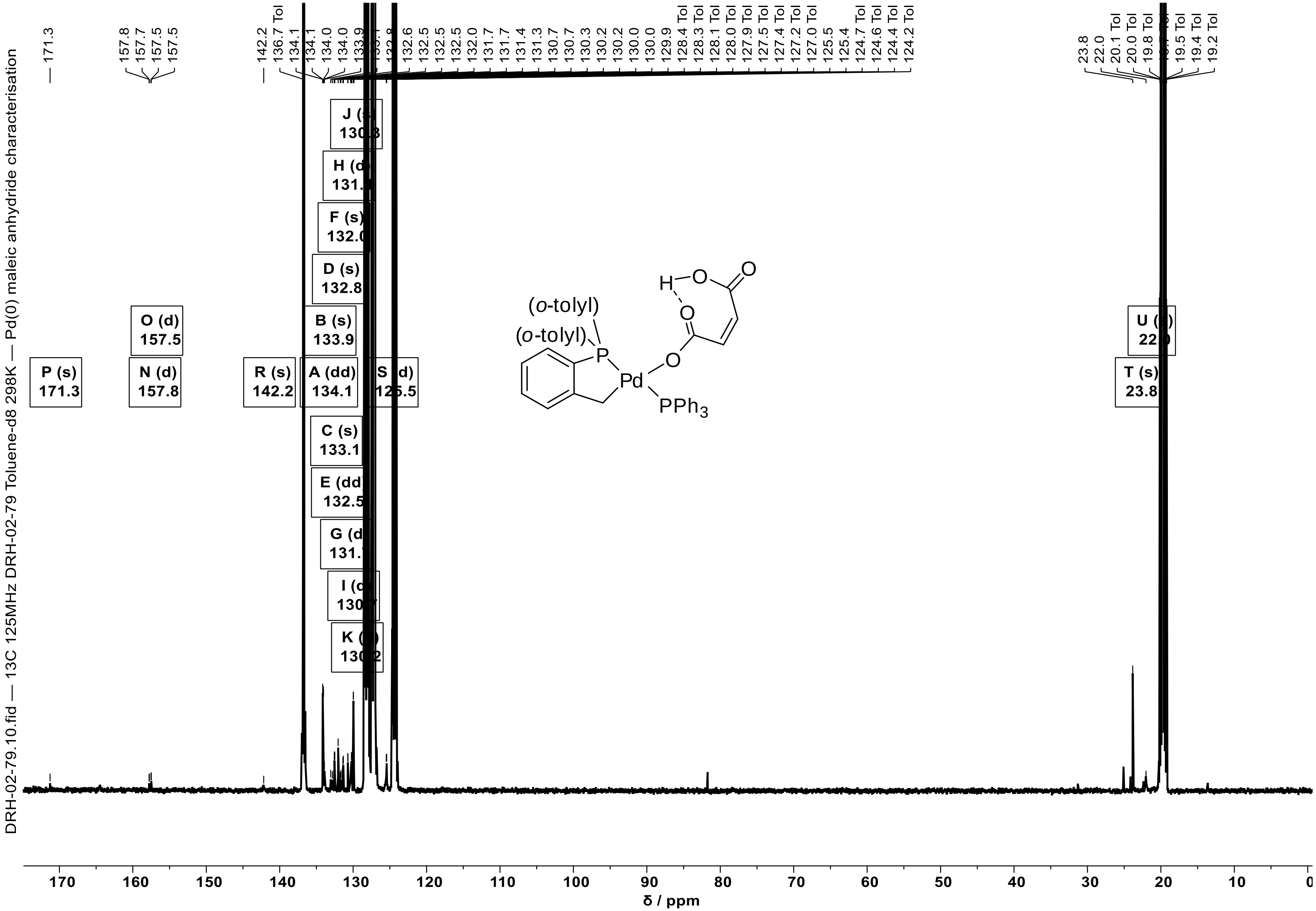

Figure 140: ^{13}C NMR (126 MHz, Toluene-d_8, 19052 scans, 298 K) spectrum of the maleic acid Pd complex 27. Lab book ref. DRH-02-79

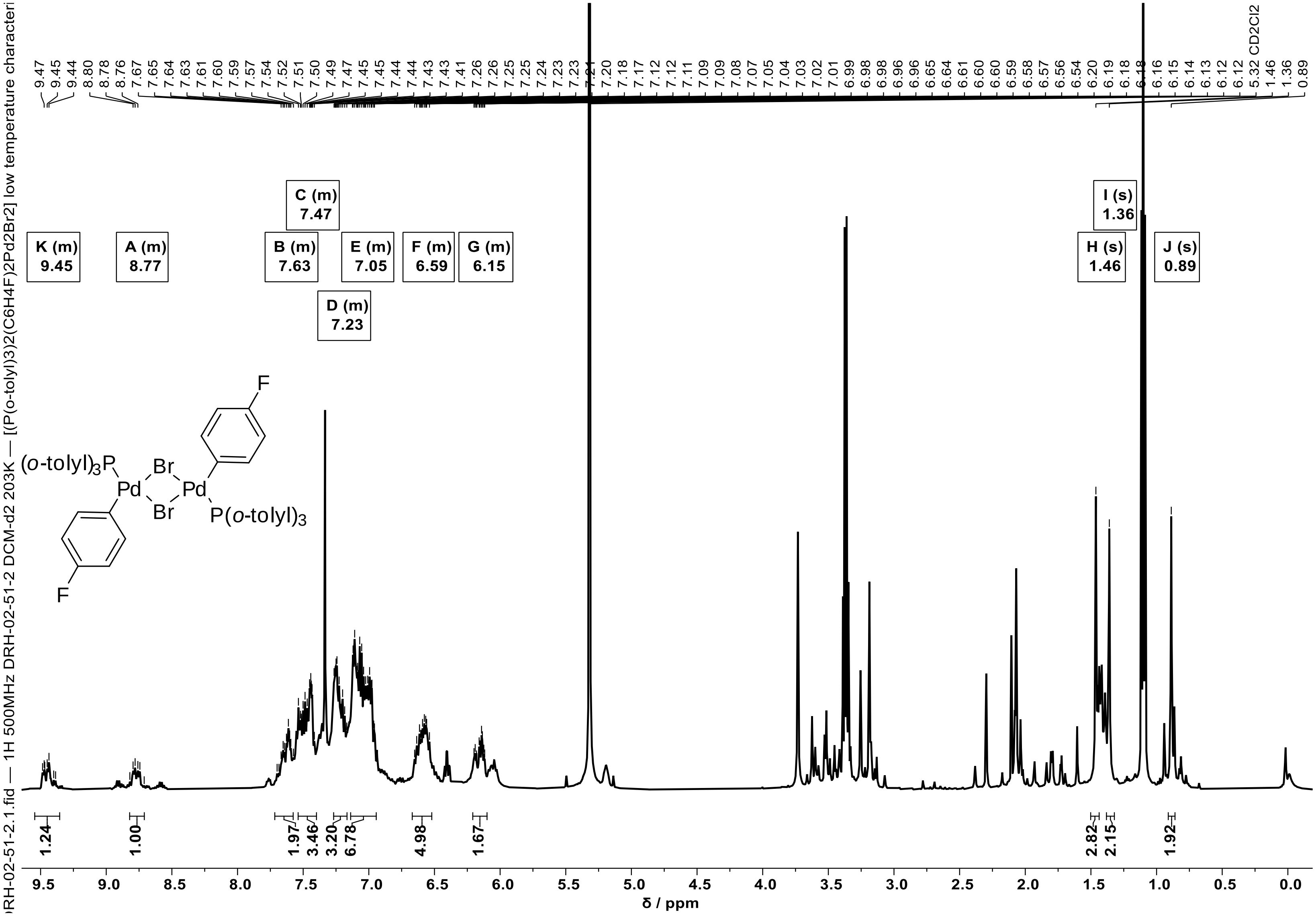

Figure 141: ^{1}H NMR (500 MHz, DCM-d_2, 64 scans, 203 K) spectrum of the $Pd_2(\mu_2\text{-}Br)_2(C_6H_4F)_2(P(o\text{-tolyl})_3)_2$ oxidative addition complex SI45. Lab book ref. DRH-02-50-1, DRH-02-51-2

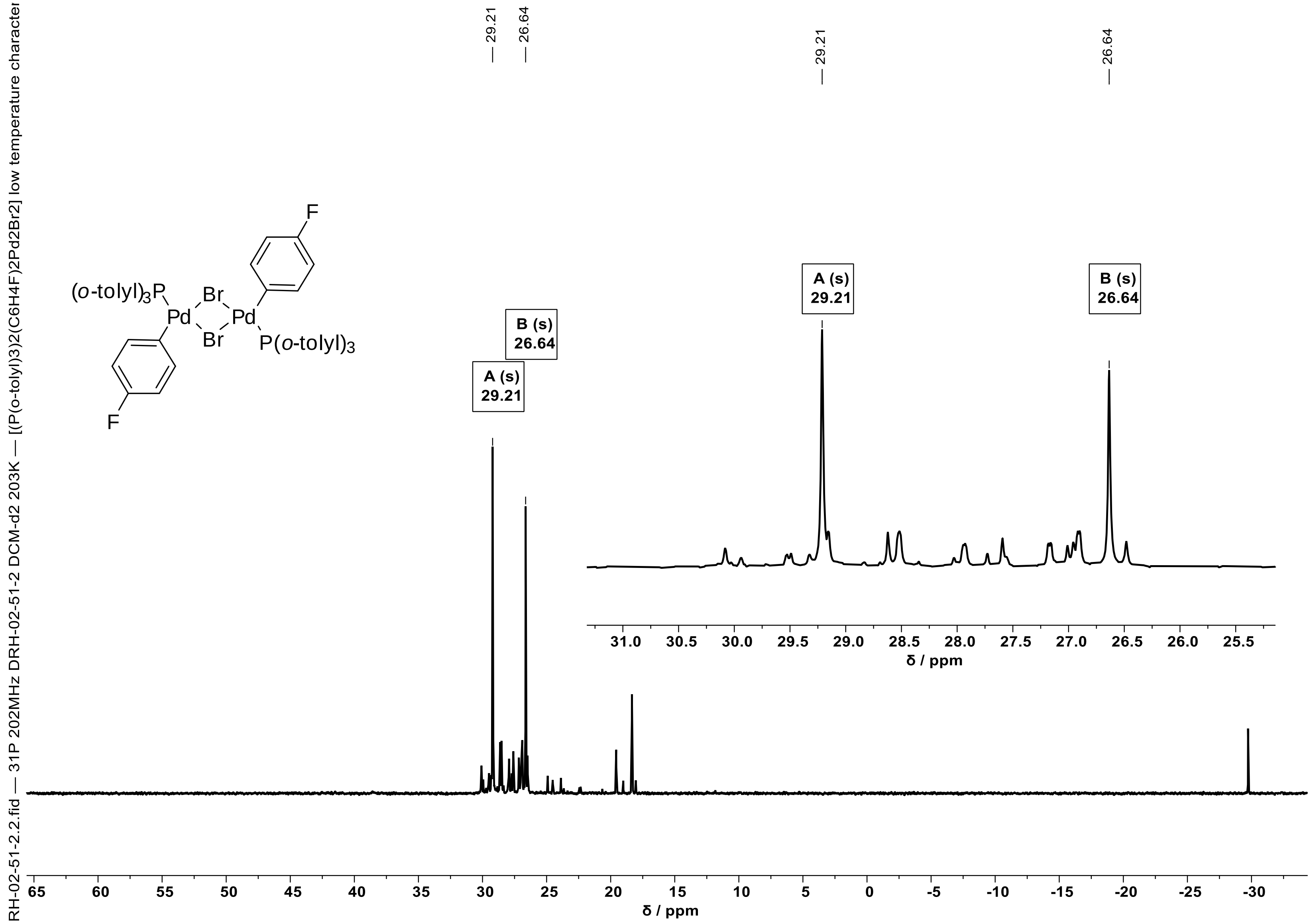

Figure 142: ^{31}P NMR (203 MHz, DCM-d_2, 128 scans, 203 K) spectrum of the $Pd_2(\mu_2\text{-}Br)_2(C_6H_4F)_2(P(\textit{o}\text{-tolyl})_3)_2$ oxidative addition complex SI45. Only the relevant ^{31}P environments (-*cis* and -*trans* isomers) have been identified. Lab book ref. DRH-02-50-1, DRH-02-51-2

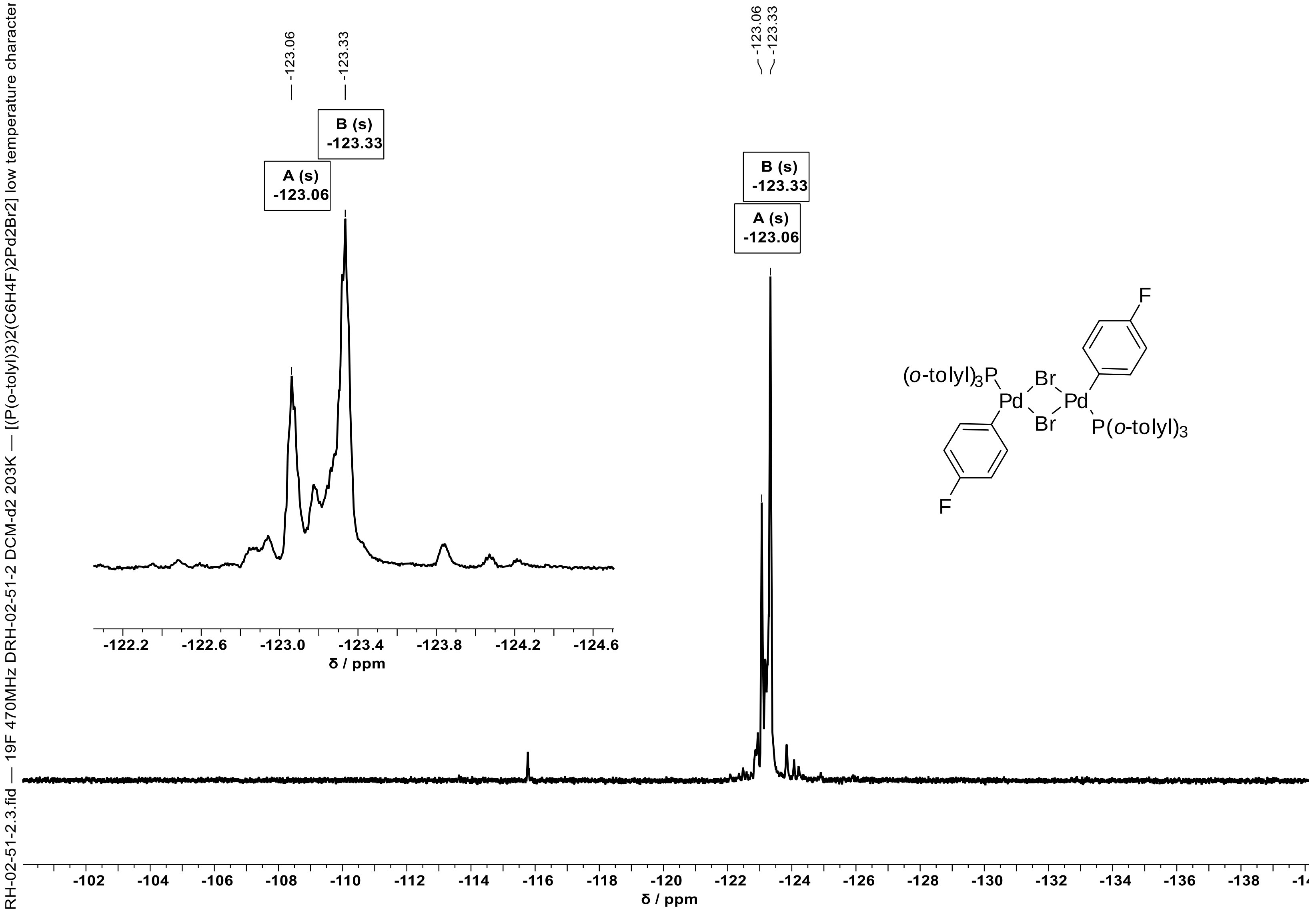

Figure 143: ^{19}F NMR (471 MHz, DCM-d_2, 128 scans, 203 K) spectrum of the $Pd_2(\mu_2\text{-}Br)_2(C_6H_4F)_2(P(o\text{-tolyl})_3)_2$ oxidative addition complex SI45. Lab book ref. DRH-02-50-1, DRH-02-51-2

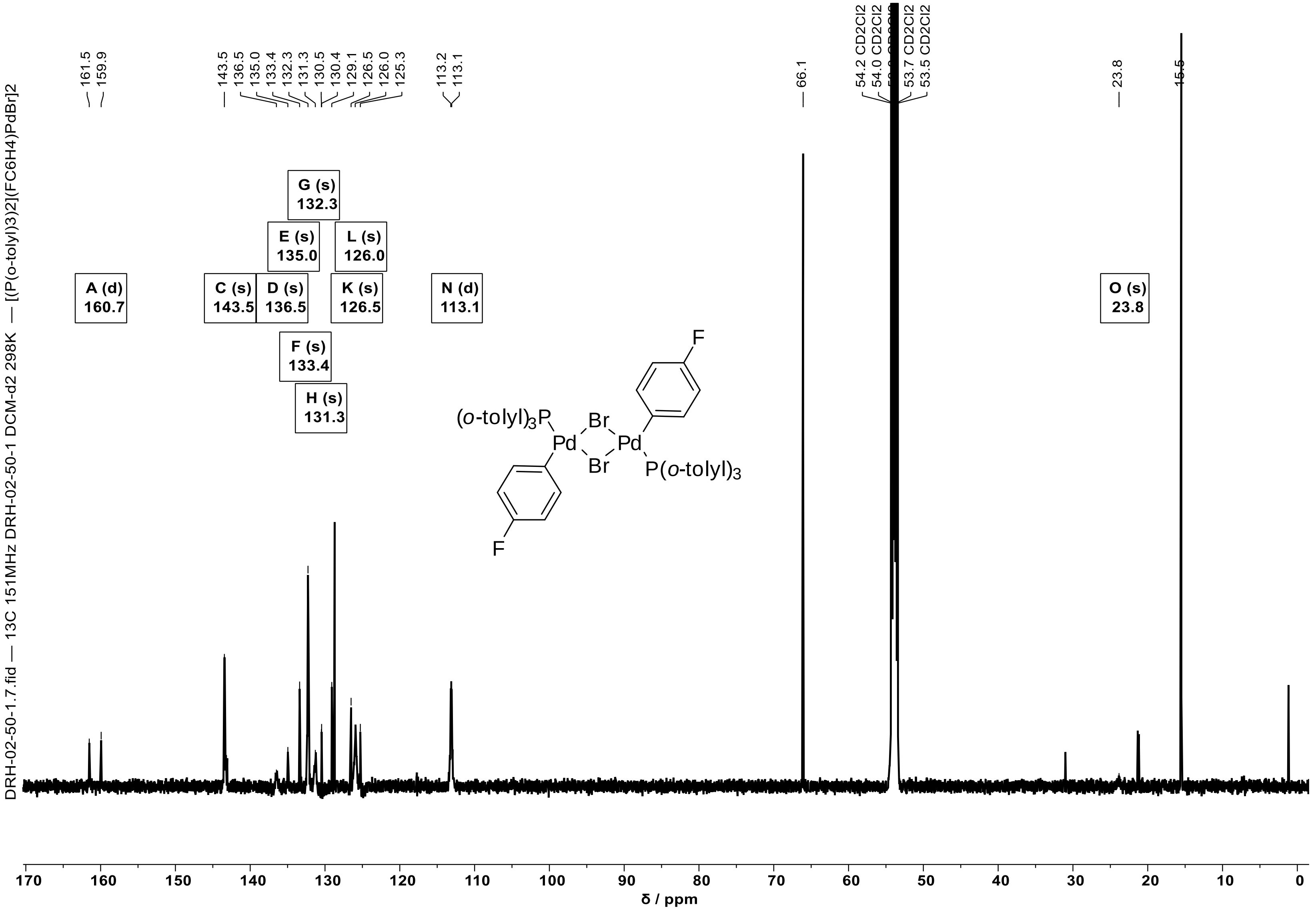

Figure 144: ^{13}C NMR (151 MHz, DCM-d_2, 16384 scans, 298 K) spectrum of the $Pd_2(\mu_2\text{-}Br)_2(C_6H_4F)_2(P(o\text{-tolyl})_3)_2$ oxidative addition complex SI45. Lab book ref. DRH-02-50-1, DRH-02-51-2

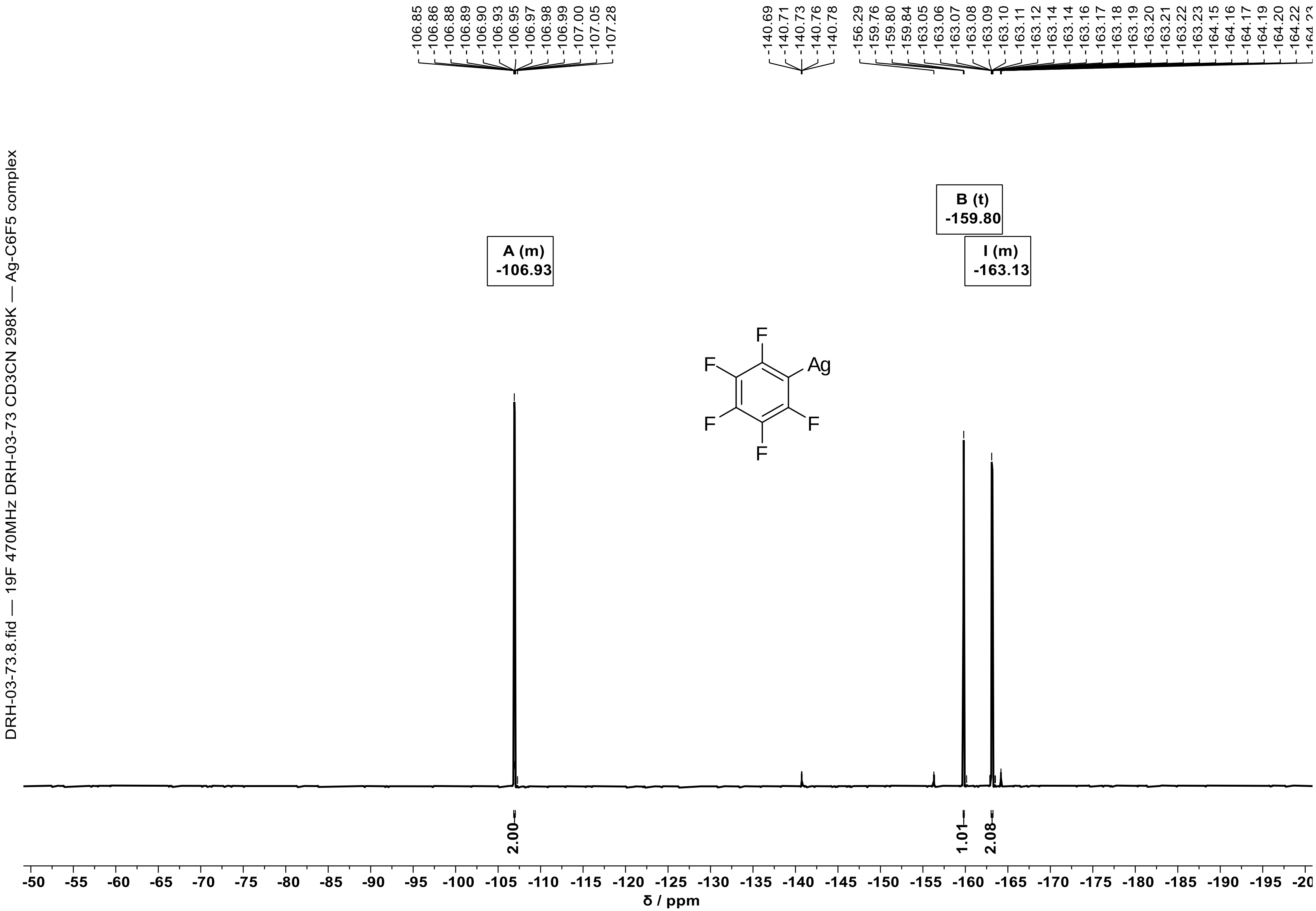

Figure 145: ^{19}**F NMR (470 MHz, Acetonitrile-*d*$_3$, 32 scans, 298 K) spectrum of the Ag-C_6F_5 complex 18. Lab book ref. DRH-03-73**

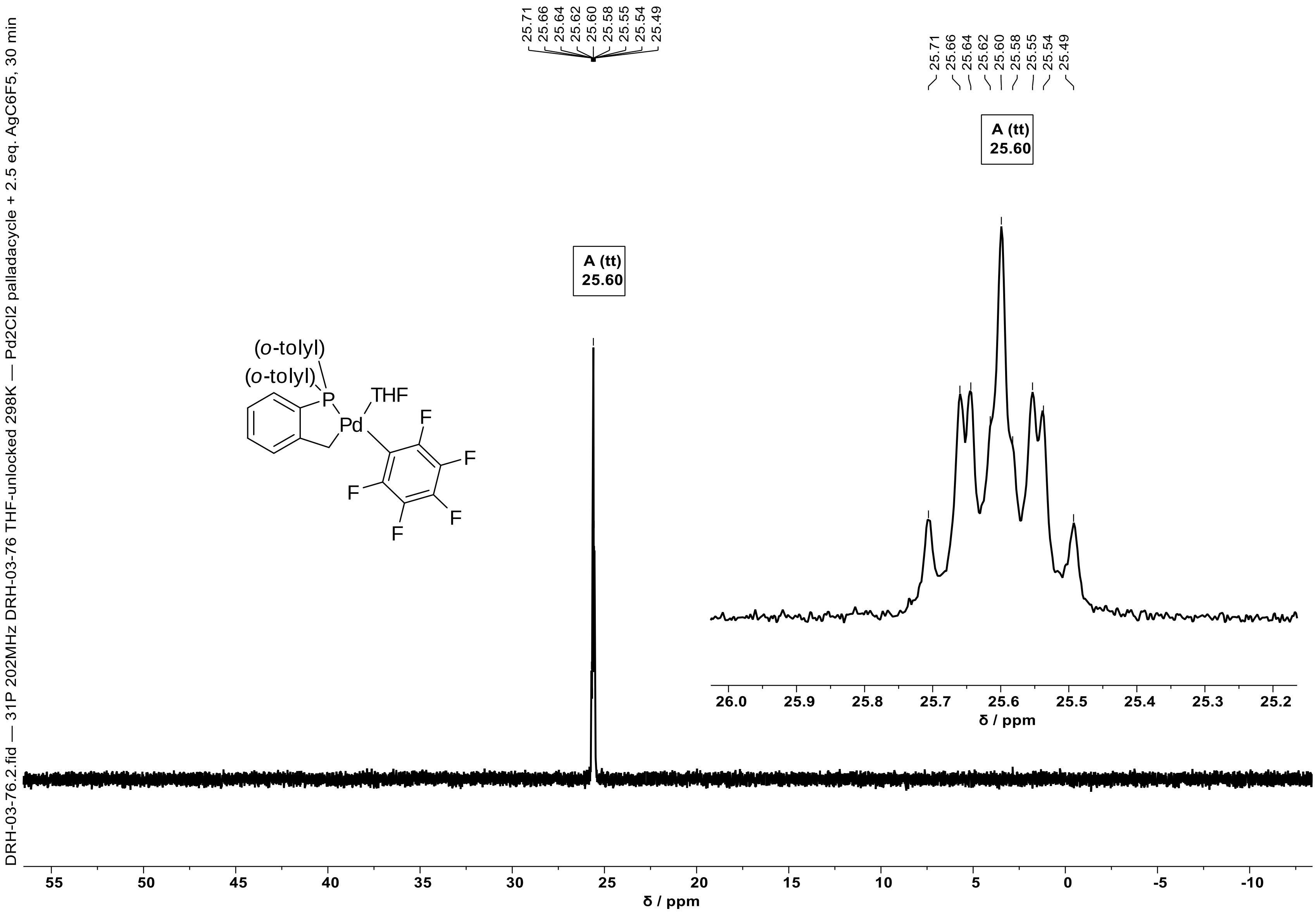

Figure 146: ^{31}**P NMR (203 MHz, THF-unlocked, 128 scans, 298 K) spectrum of the [Pd(C^P)(Ar^{F})] monomer complex 16a-m. Lab book ref. DRH-03-76**

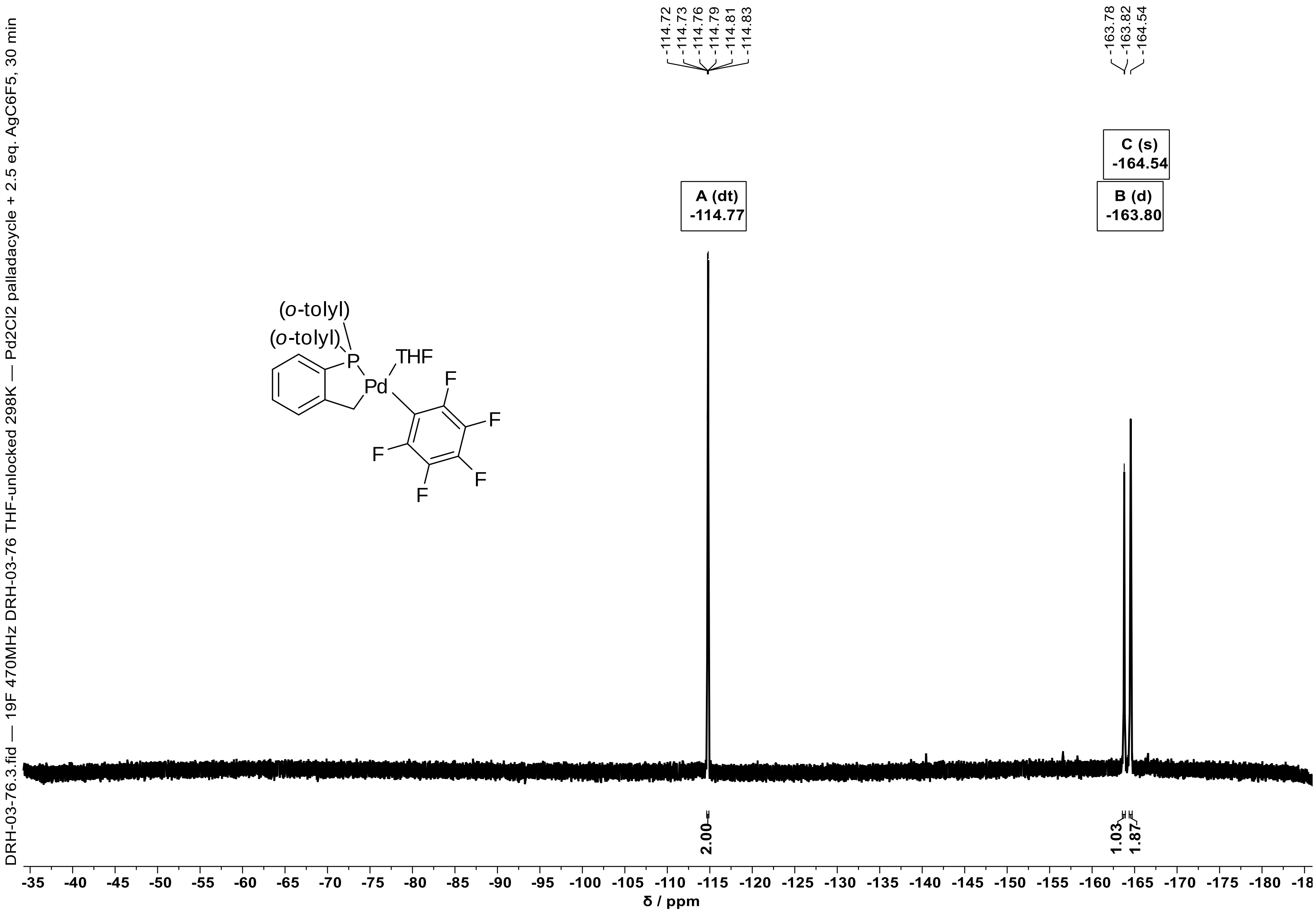

Figure 147: ^{19}F NMR (470 MHz, THF-unlocked, 128 scans, 298 K) spectrum of [Pd(C^P)(Ar^F)] monomer complex 16a-m. Lab book ref. DRH-03-76

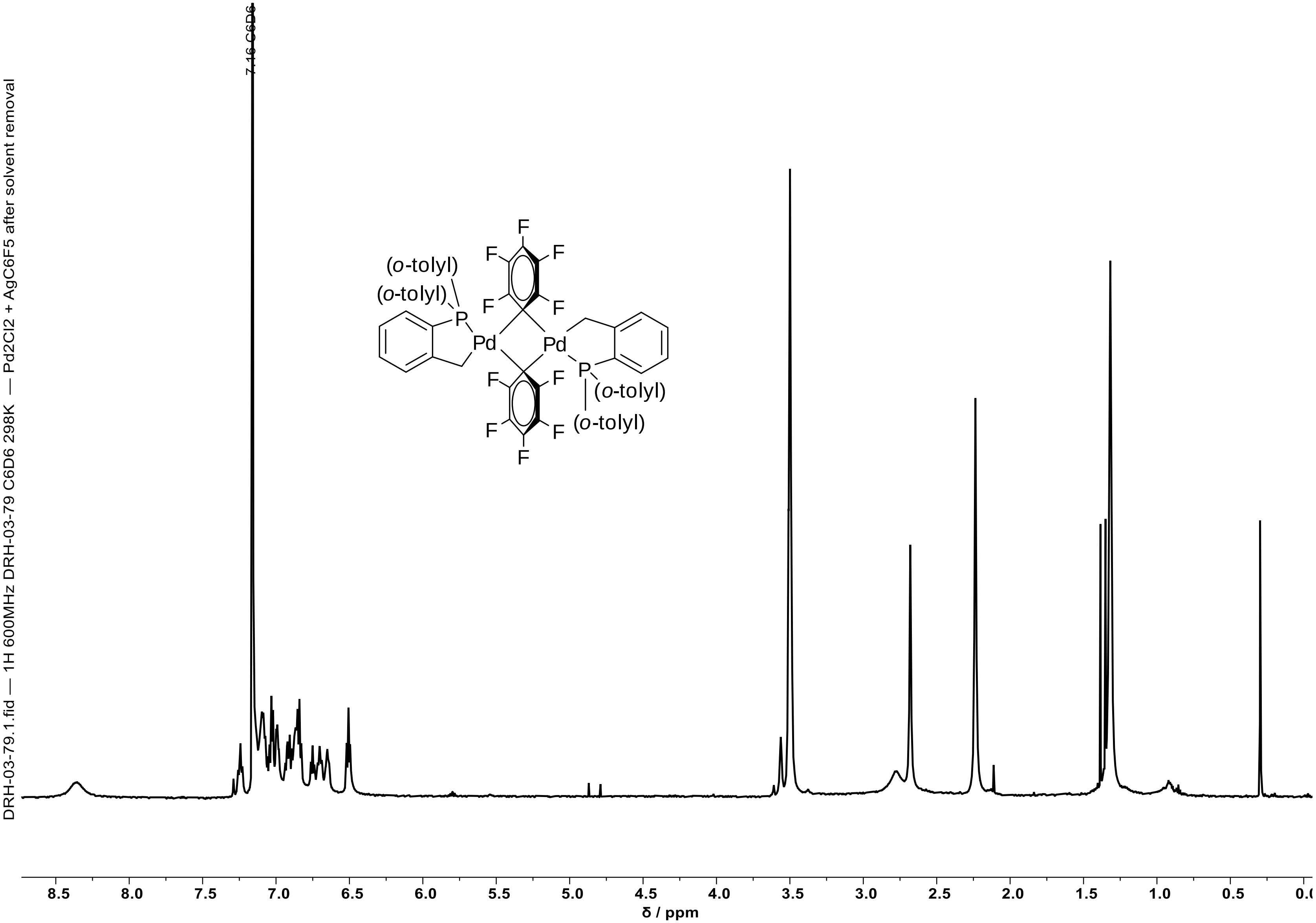

Figure 148: ^{1}H NMR (600 MHz, Benzene-d_6, 32 scans, 298 K) spectrum of the [Pd(P^C)(μ_2-Ar^F)]$_2$ palladacycle 16a. Lab book ref. DRH-03-79

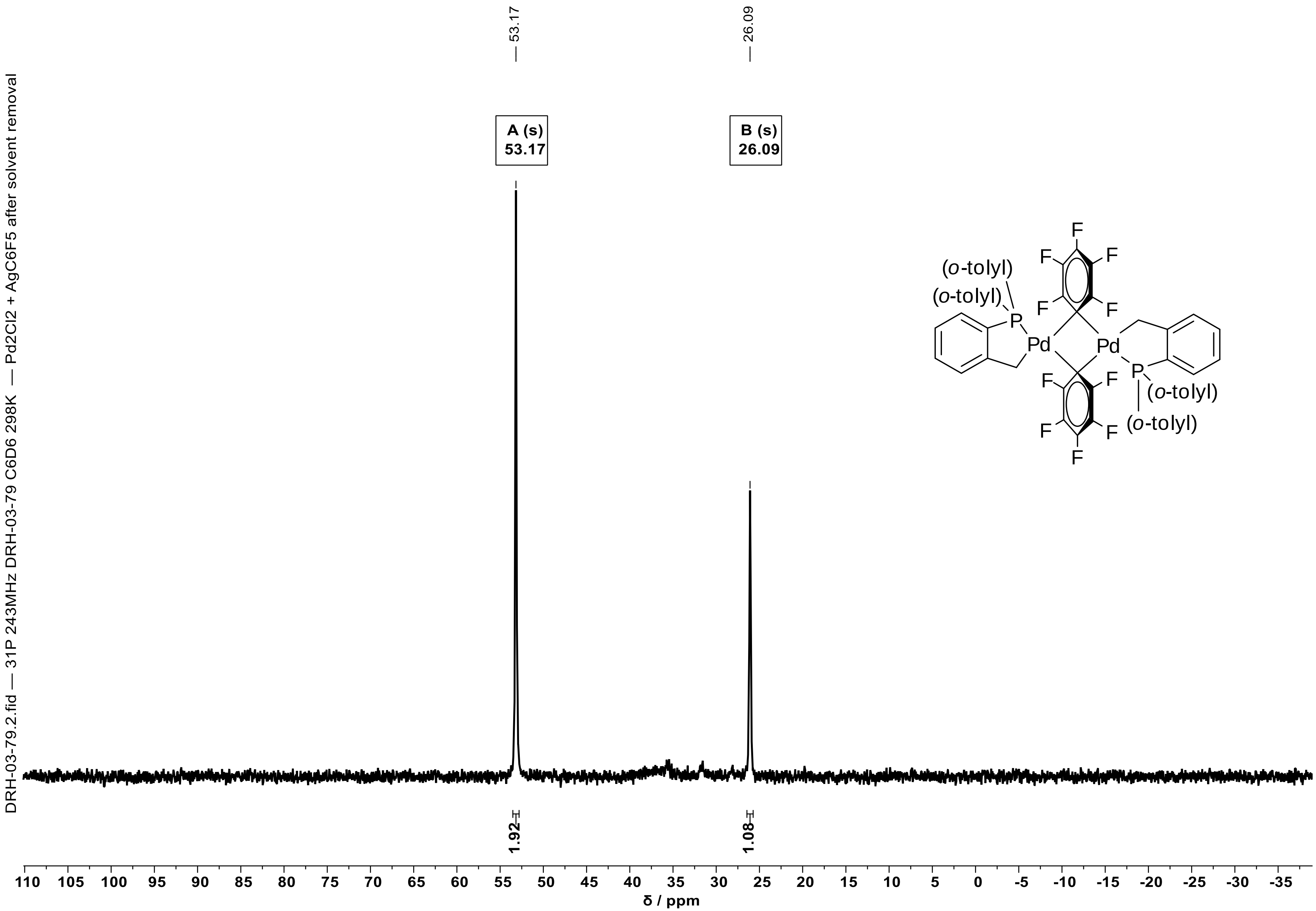

Figure 149: ^{31}P NMR (243 MHz, Benzene-d_6, 128 scans, 298 K) spectrum of the [Pd(P^C)(μ_2-Ar^F)]$_2$ palladacycle 16a. Lab book ref. DRH-03-79

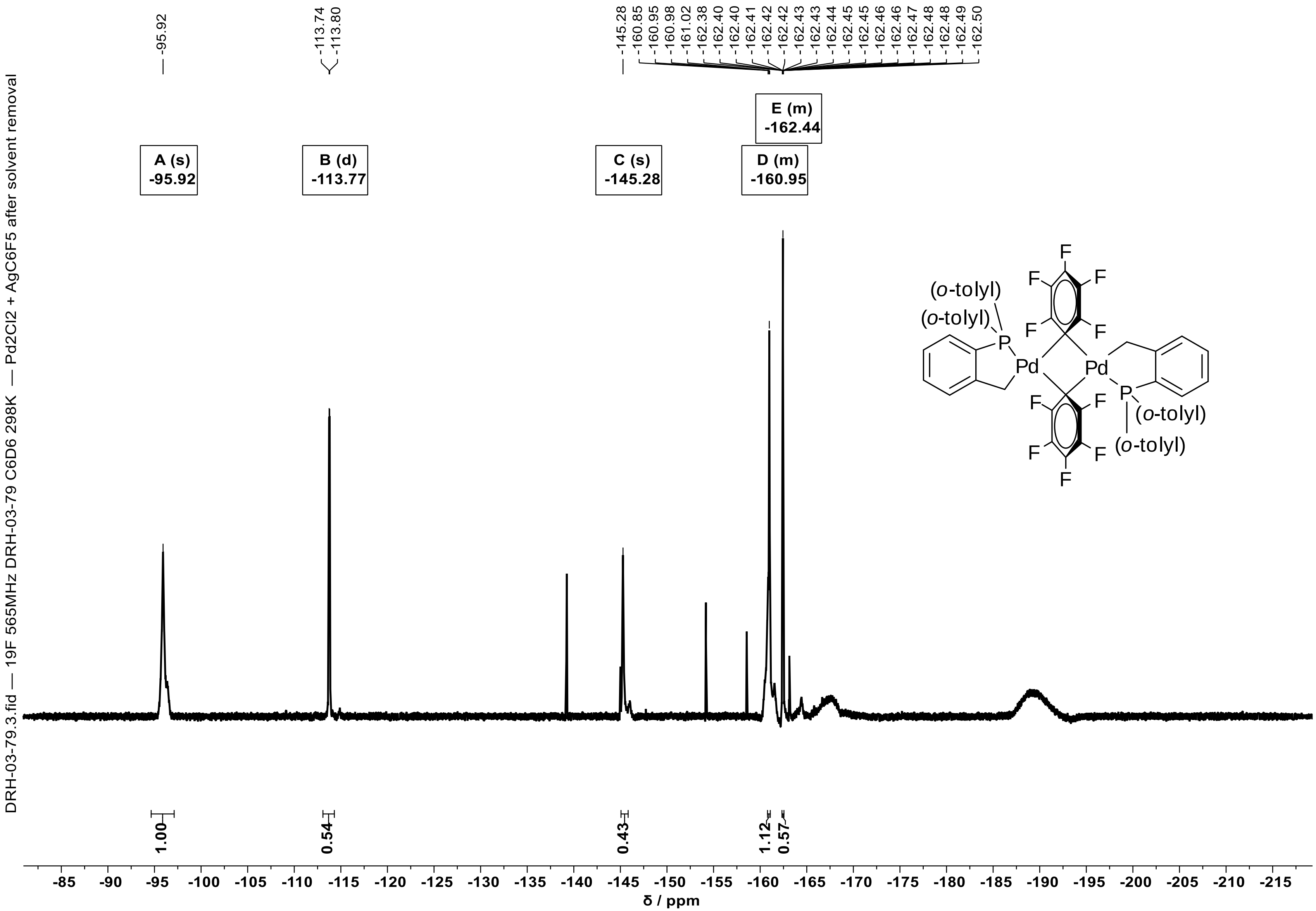

Figure 150: **^{19}F NMR (565 MHz, Benzene-d_6, 128 scans, 298 K) spectrum of the [Pd(P^C)(μ_2-Ar^F)]$_2$ palladacycle 16a. Lab book ref. DRH-03-79**

10. Density Functional Theory (DFT) Calculations

10.1 DFT Methodology and SPE Calculations for Proposed $Pd^{(II)}/Pd^{(IV)}$ Cross-Coupling Mechanism

Unless stated otherwise, all calculations were performed at the DFT level using the B3LYP functional[20–22] with a superfine integration grid for all atoms in the Gaussian16(revision A.03) suite of programs.[23] Geometry optimization and frequency calculations were performed using the def2-SVP basis set in the gas phase for all structures. Single-point energy (SPE) calculations were performed at the B3LYP/def2-TZVPP level of theory,[24,25] with the SMD continuum model[26] (solvent = dichloromethane). The D3 version of Grimme's dispersion corrections with Becke-Johnson damping[27] were used for the energy calculations. Unless stated otherwise, all calculations were carried out at 298.15 K and 1.00 Atm pressure. The nature of stationary points was verified by frequency calculations, with minima having zero imaginary frequencies, and transition states having only one imaginary frequency.

Dispersion corrections were only applied to the SPE calculations and not to the geometry optimizations. This is due to palladacycles such as **2** and **35** puckering around the Pd-X-Pd bridge, indicating an over-correction in the calculations with respect to the crystal structure geometries. As such, dispersion corrections at both optimization and SPE would lead to inaccurate calculations.

Table 27: Single point energies (SPE) of optimized structures. Optimization was carried out at the B3LYP/def2svp (gas phase) level of theory. SPE calculations <Functional>/def2tzvpp, SMD (dichloromethane) solvent correction, empirical dispersion = GD3BJ.

	Single Point Energy (SPE) / Hartree			
**Molecule**	**B3LYP**	**PBE0**	**LC-ωPBE**	**B3PW91**
**2**	-2716.020089	-2713.24227	-2714.294325	-2715.304705
**4**	-523.064633	-522.407354	-522.681152	-522.848702
**37**	-1881.078197	-1879.033227	-1879.83835	-1880.507693
**TS1**	-1881.063254	-1879.018175	-1879.821605	-1880.494374
**38**	-1881.072316	-1879.027884	-1879.83168	-1880.504523
**TS2**	-1881.057003	-1879.015496	-1879.812656	-1880.489598
**39**	-1881.080492	-1879.032785	-1879.831887	-1880.508674
**Boric acid**	-252.632002	-252.341642	-252.471546	-252.524757
**13a**	-1628.435146	-1626.679088	-1627.343769	-1627.96986
**3**	-2905.233521	-2904.419031	-2904.60293	-2905.086433
**TS3**	-4533.646344	-4531.085943	-4531.912507	-4533.039179
**36**	-4533.684276	-4531.119668	-4531.957001	-4533.07833
**TS4**	-4533.679957	-4531.115626	-4531.954117	-4533.074998
**5**	-677.39887	-676.549339	-676.887263	-677.129223
**35a**	-3856.357907	-3854.634368	-3855.153055	-3856.017654
**35**	-7712.760608	-7709.308144	-7710.354954	-7712.081801

Table 28: Reaction energies (ΔG) from single point energy calculations

		Energy (ΔG) from SPE / kcal mol^{-1}			
**Reaction coordinate**	**Reaction**	**B3LYP**	**PBE0**	**LC-ωPBE**	**B3PW91**
1	½ 2 + 4 -->37	-2.2085	-2.97311	-6.29731	-4.16568
2	37 -->TS1	9.376777	9.445175	10.50754	8.357712
3	TS1 --> 38	-5.68643	-6.09243	-6.32209	-6.36853
4	38 --> TS2	9.608953	7.773507	11.93762	9.365482
5	TS2 --> 39	-14.7394	-10.8489	-12.0675	-11.9702
6	39 --> 13a + boric acid	8.3734	7.564549	10.39898	8.82081
7	13a + 3 --> TS3	14.00775	7.640477	21.45558	10.73909
8	TS3 --> 36	-23.8024	-21.1625	-27.9201	-24.5674
9	36 --> TS4	2.710185	2.536367	1.809719	2.09084
10	TS4 --> 35a + 5	-48.2048	-42.721	-54.0914	-45.1043
11	35a --> ½ 35	-14.0542	-12.3643	-15.3249	-14.5872

Table 29: Energetic span (δE) between lowest energy reaction coordinate (0 – 6) and reaction coordinate 7 (TS3)

Functional	Energetic span δE / kcal mol^{-1}
B3LYP	22.38115
PBEPBE	15.48238
LC-wPBE	35.91011
B3PW91	19.5599

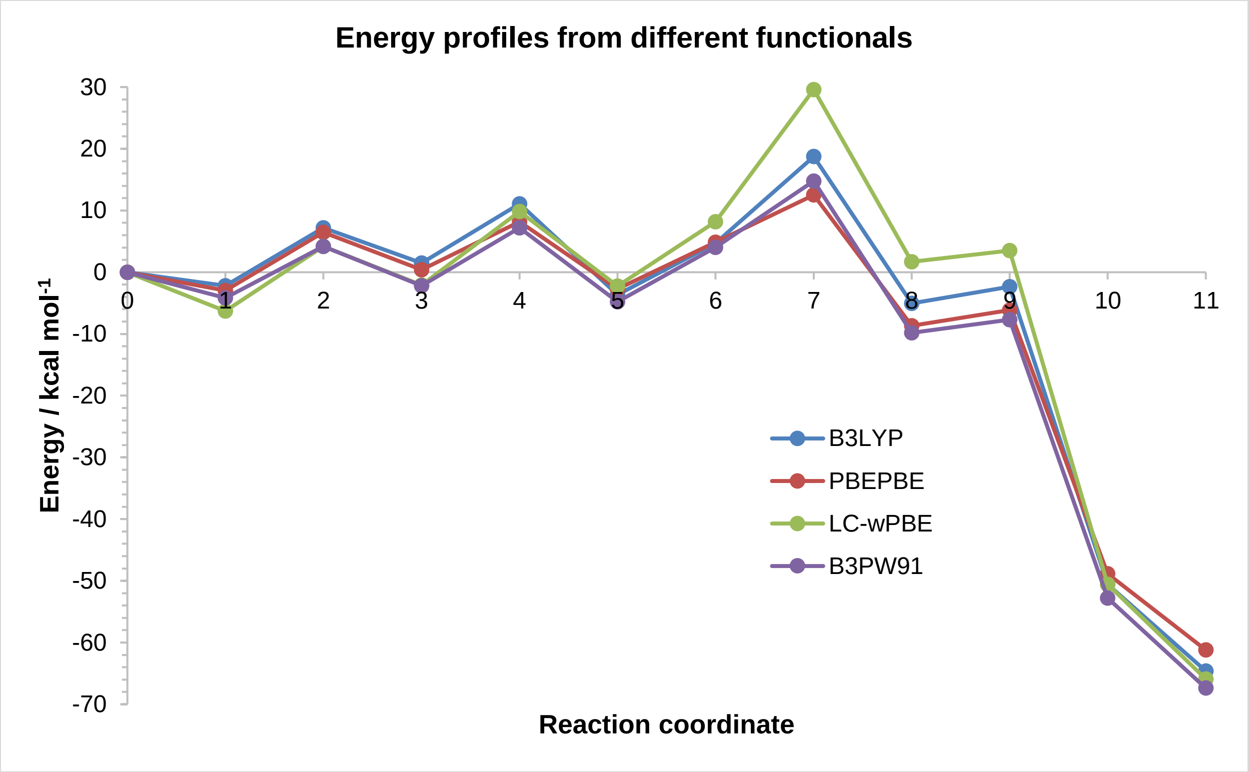

Figure 151: Calculated reaction energies for the proposed pathway (kcal mol^{-1}) from SPE calculations with different functionals.

10.2 Stabilization of Complex 13a and 36 by different ligands

The proposed complex **13a** contains a vacant coordination site prior to oxidative addition for the proposed $Pd^{(II)}$/$Pd^{(IV)}$ mechanism. As shown in Figure 9 in the manuscript, boric acid can weakly coordinate this site (**39**) and needs to be dissociated. However, it is possible for other molecules to coordinate in place of boric acid, and the coordination energies of DCM, H_2O, NMP and THF have been calculated (Table 31). Although all calculated molecules bind more strongly to **13a** than boric acid, and hence raise the energetic span by 3 – 7 kcal mol^{-1} (for B3LYP), it should be noted that under the stoichiometric reaction conditions that this is modelling, only trace water is present (along with DCM solvent), and no THF/NMP. As the implicit solvent model for the DFT calculations is DCM, **39_DCM** is effectively using explicit solvent modelling, which lowers the energy of the structure relative to the other modelled complexes. As such, the energies calculated here may not be representative of the true energy levels. However, the calculations do demonstrate that under catalytic conditions, NMP and THF do bind strongly and would likely displace boric acid, hence raising the energy barrier to oxidative addition.

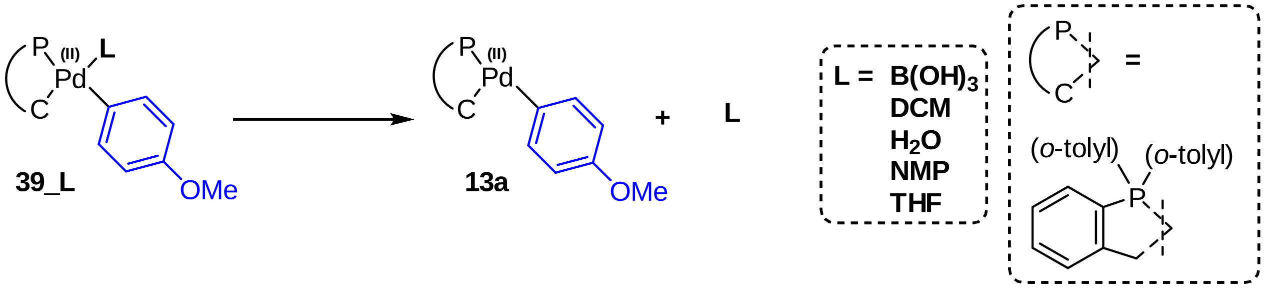

Table 30: Single point energies (SPE) of optimized structures for the binding of ligands L to 13a. Optimization was carried out at the B3LYP/def2svp (gas phase) level of theory. SPE calculations <Functional>/def2tzvpp, SMD (dichloromethane) solvent correction, empirical dispersion = GD3BJ.

	**Single Point Energy (SPE) / Hartree**	
**Molecule**	**B3LYP**	**PBE0**
**39**	-1881.080492	-1879.032785
**Boric acid**	-252.632002	-252.341642
**13a**	-1628.435146	-1626.679088
**39_DCM**	-2588.245063	-2586.026044
**DCM**	-959.792156	-959.330028
**39_H_2O**	-1704.927462	-1703.086044
**H_2O**	-76.474283	-76.387475
**39_NMP**	-1954.572805	-1952.394706
**NMP**	-326.112628	-325.691993
**39_THF**	-1861.028526	-1858.958795
**THF**	-232.568188	-232.256598

Table 31: Reaction energies (ΔG) from single point energy calculations for the dissociation of 39_L to 13a and L.

	**Energy (ΔG) from SPE / kcal mol^{-1}**	
**Reaction**	**B3LYP**	**PBE0**
39 --> 13a + boric acid	8.3734	7.564549
39_DCM --> 13a + DCM	11.14508	10.62237
39_H_2O--> 13a + H_2O	11.31576	12.22439
39_NMP --> 13a + NMP	15.70703	14.82476
39_THF --> 13a + THF	15.80806	14.50097
39 --> 13a + boric acid	8.3734	7.564549

To demonstrate the stabilization of explicit solvent modelling on other key structures, Pd$^{(IV)}$ intermediate **36** (which is modelled as a 5-coordinate molecule with a vacant coordination site) was optimized with the same ligand set. All ligands stabilize the Pd$^{(IV)}$ intermediate **36** to a similar extent as **13a**, even when not directly coordinated to the metal center (DCM and THF). It was not feasible to model the transition states **TS3** and **TS4** with explicit solvent, but it is expected that the energy of these structures would be stabilized

by a similar amount (approx.. 10 kcal mol^{-1}). As such, it is anticipated that solvent participation in the key oxidative addition step should not significantly affect the energetic span of the process.

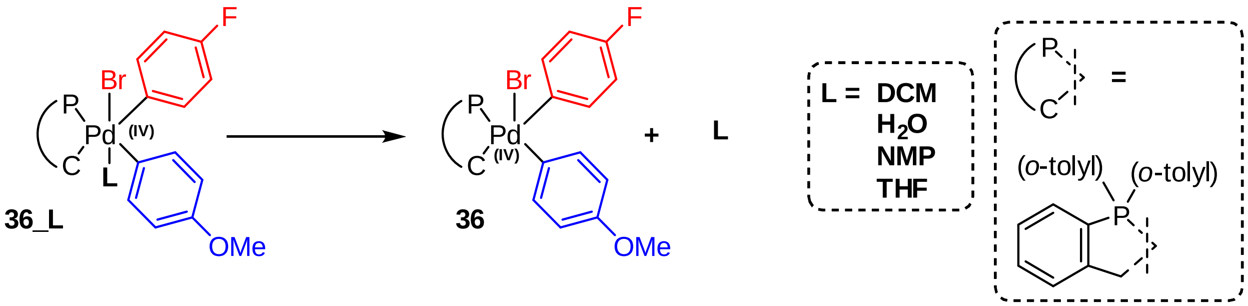

Table 32: Single point energies (SPE) of optimized structures for the binding of ligands L to 36. Optimization was carried out at the B3LYP/def2svp (gas phase) level of theory. SPE calculations <Functional>/def2tzvpp, SMD (dichloromethane) solvent correction, empirical dispersion = GD3BJ.

	Single Point Energy (SPE) / Hartree	
**Molecule**	**B3LYP**	**PBE0**
**36**	-4533.684276	-4531.119668
**36_DCM**	-5493.490798	-5490.459446
**36_H_2O**	-4610.179221	-4607.525803
**36_NMP**	-4859.818464	-4856.826955
**36_THF**	-4766.265556	-4763.385716

Table 33: Reaction energies (ΔG) from single point energy calculations for the dissociation of 36_L to 36 and L. SPE for solvents are taken from Table 30.

	Energy (ΔG) from SPE / kcal mol^{-1}	
**Reaction**	**B3LYP**	**PBE0**
36_DCM --> 36 + DCM	9.01471	6.11815
36_H_2O --> 36 + H_2O	12.9655	11.7092
36_NMP --> 36 + NMP	13.529	9.59703
36_THF --> 36 + THF	8.21527	5.9299

11. References

(1) Liu, C.; Meng, G.; Szostak, M. N-Acylsaccharins as Amide-Based Arylating Reagents via Chemoselective N–C Cleavage: Pd-Catalyzed Decarbonylative Heck Reaction. *J. Org. Chem.* **2016**, *81* (23), 12023–12030. https://doi.org/10.1021/acs.joc.6b02294.

(2) Manna, K.; Jana, R. Palladium-Catalyzed Cross-Electrophile Coupling between Aryl Diazonium Salt and Aryl Iodide/Diaryliodonium Salt in H2O–EtOH. *Org. Lett.* **2023**, *25* (2), 341–346. https://doi.org/10.1021/acs.orglett.2c03932.

(3) Olding, A.; Ho, C. C.; Canty, A. J.; Lucas, N. T.; Horne, J.; Bissember, A. C. Synthesis of Arylpalladium(II) Boronates: Confirming the Structure and Chemical Competence of Pre-Transmetalation Intermediates in the Suzuki–Miyaura Reaction. *Angew. Chem. Int. Ed.* **2021**, *60* (27), 14897–14901. https://doi.org/10.1002/anie.202104802.

(4) Fukuda, K.; Iwasawa, N.; Takaya, J. Ruthenium-Catalyzed Ortho C−H Borylation of Arylphosphines. *Angew. Chem. Int. Ed.* **2019**, *58* (9), 2850–2853. https://doi.org/10.1002/anie.201813278.

(5) Paul, F.; Patt, J.; Hartwig, J. F. Structural Characterization and Simple Synthesis of {Pd[P(o-Tol)3]2}. Spectroscopic Study and Structural Characterization of the Dimeric Palladium(II) Complexes Obtained by Oxidative Addition of Aryl Bromides and Their Reactivity with Amines. *Organometallics* **1995**, *14* (6), 3030–3039. https://doi.org/10.1021/om00006a053.

(6) Shell, T. A.; Mohler, D. L. Selective Targeting of DNA for Cleavage within DNA–Histone Assemblies by a Spermine–[CpW(CO)3Ph]2 Conjugate. *Org. Biomol. Chem.* **2005**, *3* (17), 3091–3093. https://doi.org/10.1039/B504248H.

(7) Dubois, N.; Glynn, D.; McInally, T.; Rhodes, B.; Woodward, S.; Irvine, D. J.; Dodds, C. On DABAL-Me3 Promoted Formation of Amides. *Tetrahedron* **2013**, *69* (46), 9890–9897. https://doi.org/10.1016/j.tet.2013.08.062.

(8) Athavan, G.; Tanner, T. F. N.; Whitwood, A. C.; Fairlamb, I. J. S.; Perutz, R. N. Direct Evidence for Competitive C–H Activation by a Well-Defined Silver XPhos Complex in Palladium-Catalyzed C–H Functionalization. *Organometallics* **2022**. https://doi.org/10.1021/acs.organomet.2c00063.

(9) Albéniz, A. C.; Espinet, P.; López-Cimas, O.; Martín-Ruiz, B. Dimeric Palladium Complexes with Bridging Aryl Groups: When Are They Stable? *Chem. Eur. J.* **2005**, *11* (1), 242–252. https://doi.org/10.1002/chem.200400612.

(10) Hyotanishi, M.; Isomura, Y.; Yamamoto, H.; Kawasaki, H.; Obora, Y. Surfactant-Free Synthesis of Palladium Nanoclusters for Their Use in Catalytic Cross-Coupling Reactions. *Chem. Commun.* **2011**, *47* (20), 5750. https://doi.org/10.1039/c1cc11487e.

(11) Burés, J.; Larrosa, I. Organic Reaction Mechanism Classification Using Machine Learning. *Nature* **2023**, *613* (7945), 689–695. https://doi.org/10.1038/s41586-022-05639-4.

(12) Thomas, A. A.; Denmark, S. E. Pre-Transmetalation Intermediates in the Suzuki-Miyaura Reaction Revealed: The Missing Link. *Science* **2016**, *352* (6283), 329–332. https://doi.org/10.1126/SCIENCE.AAD6981.

(13) Jordan, A.; Hall, C. G. J.; Thorp, L. R.; Sneddon, H. F. Replacement of Less-Preferred Dipolar Aprotic and Ethereal Solvents in Synthetic Organic Chemistry with More Sustainable Alternatives. *Chem. Rev.* **2022**, *122* (6), 6749–6794. https://doi.org/10.1021/acs.chemrev.1c00672.

(14) M. Heravi, M.; Ghavidel, M.; Mohammadkhani, L. Beyond a Solvent: Triple Roles of Dimethylformamide in Organic Chemistry. *RSC Advances* **2018**, *8* (49), 27832–27862. https://doi.org/10.1039/C8RA04985H.

(15) Baumann, C. G.; De Ornellas, S.; Reeds, J. P.; Storr, T. E.; Williams, T. J.; Fairlamb, I. J. S. Formation and Propagation of Well-Defined Pd Nanoparticles (PdNPs) during C-H Bond Functionalization of Heteroarenes: Are Nanoparticles a Moribund Form of Pd or an Active Catalytic Species? *Tetrahedron* **2014**, *70* (36), 6174–6187. https://doi.org/10.1016/j.tet.2014.06.002.

(16) CrysAlisPro. CrysAlisPro, Oxford Diffraction Ltd. Version 1.171.34.41. p Oxford Diffraction Ltd. Version 1.171.34.41.

(17) Empirical absorption correction using spherical harmonics implemented in SCALE3 ABSPACK scaling algorithm within CrysAlisPro software. Scale3. Empirical absorption correction using spherical harmonics, implemented in SCALE3 ABSPACK scaling algorithm within CrysAlisPro software, p Oxford Diffraction Ltd. Version 1.171.34.40.

(18) Dolomanov, O. V.; Bourhis, L. J.; Gildea, R. J.; Howard, J. A. K.; Puschmann, H. OLEX2: A Complete Structure Solution, Refinement and Analysis Program. *J. Appl. Crystallogr.* **2009**, *42* (2), 339–341. https://doi.org/10.1107/S0021889808042726.

(19) Sheldrick, G. M. SHELXT - Integrated Space-Group and Crystal-Structure Determination. *Acta Crystallogr.* **2015**, *71* (1), 3–8. https://doi.org/10.1107/S2053273314026370.

(20) Lee, C.; Yang, W.; Parr, R. G. Development of the Colle-Salvetti Correlation-Energy Formula into a Functional of the Electron Density. *Phys. Rev. B* **1988**, *37* (2), 785–789. https://doi.org/10.1103/PhysRevB.37.785.

(21) Becke, A. D. Density-Functional Thermochemistry. III. The Role of Exact Exchange. *J. Chem. Phys.* **1993**, *98* (7), 5648–5652. https://doi.org/10.1063/1.464913.

(22) Stephens, P. J.; Devlin, F. J.; Chabalowski, C. F.; Frisch, M. J. Ab Initio Calculation of Vibrational Absorption and Circular Dichroism Spectra Using Density Functional Force Fields. *The Journal of Physical Chemistry* **1994**, *98* (45), 11623–11627. https://doi.org/10.1021/j100096a001.

(23) Frisch, M. J.; Trucks, G. W.; Schlegel, H. B.; Scuseria, G. E.; Robb, M. A.; Cheeseman, J. R.; Scalmani, G.; Barone, V.; Mennucci, B.; Petersson, G. A.; Nakatsuji, H.; Caricato, M.; Li, X.; Hratchian, H. P.; Izmaylov, A. F.; Bloino, J.; Zheng, G.; Sonnenberg, J. L.; Hada, M.; Ehara, M.; Toyota, K.; Fukuda, R.; Hasegawa, J.; Ishida, M.; Nakajima, T.; Honda, Y.; Kitao, O.; Nakai, H.; Vreven, T.; Montgomery Jr., J. A.; Peralta, J. E.; Ogliaro, F.; Bearpark, M.; Heyd, J. J.; Brothers, E.; Kudin, K. N.; Staroverov, V. N.; Kobayashi, R.; Normand, J.; Raghavachari, K.; Rendell, A.; Burant, J. C.; Iyengar, S. S.; Tomasi, J.; Cossi, M.; Rega, N.; Millam, J. M.; Klene, M.; Knox, J. E.; Cross, J. B.; Bakken, V.; Adamo, C.; Jaramillo, J.; Gomperts, R.; Stratmann, R. E.; Yazyev, O.; Austin, A. J.; Cammi, R.; Pomelli, C.; Ochterski, J. W.; Martin, R. L.; Morokuma, K.; Zakrzewski, V. G.; Voth, G. A.; Salvador, P.; Dannenberg, J. J.; Dapprich, S.; Daniels, A. D.; Farkas, O.; Foresman, J. B.; Ortiz, J. V.; Cioslowski, J.; Fox, D. J. *Gaussian16 (Revision A.03), Gaussian Inc. Wallingford CT*; 2016.

(24) Weigend, F. Accurate Coulomb-Fitting Basis Sets for H to Rn. *Phys. Chem. Chem. Phys.* **2006**, *8* (9), 1057–1065. https://doi.org/10.1039/b515623h.

(25) Weigend, F.; Ahlrichs, R. Balanced Basis Sets of Split Valence, Triple Zeta Valence and Quadruple Zeta Valence Quality for H to Rn: Design and Assessment of Accuracy. *Phys. Chem. Chem. Phys.* **2005**, *7* (18), 3297–3305. https://doi.org/10.1039/b508541a.

(26) Marenich, A. V.; Cramer, C. J.; Truhlar, D. G. Universal Solvation Model Based on Solute Electron Density and on a Continuum Model of the Solvent Defined by the Bulk Dielectric Constant and Atomic Surface Tensions. *J. Phys. Chem. B* **2009**, *113* (18), 6378–6396. https://doi.org/10.1021/jp810292n.

(27) Grimme, S.; Ehrlich, S.; Goerigk, L. Effect of the Damping Function in Dispersion Corrected Density Functional Theory. *J. Comput. Chem.* **2011**, *32* (7), 1456–1465. https://doi.org/10.1002/jcc.21759.
